# Assembly and comparative analysis of the complete mitochondrial and chloroplast genome of *Cyperus stoloniferus* (Cyperaceae), a coastal plant possessing saline-alkali tolerance

**DOI:** 10.1186/s12870-024-05333-9

**Published:** 2024-07-03

**Authors:** Xiaorong Miao, Wenwen Yang, Donghai Li, Aiqin Wang, Juanyun Li, Xu Deng, Longfei He, Junqi Niu

**Affiliations:** 1 College of Agriculture, Guangxi University, Nanning, 530004 China; 2 Guangxi Key Laboratory of Agricultural Resources Chemistry and Biotechnology, Agricultural College, Yulin Normal University, Yulin, 537000 China

**Keywords:** *Cyperus stoloniferus*, mtDNA, cpDNA, Comparative analysis, Systematic evolution

## Abstract

**Background:**

*Cyperus stoloniferus* is an important species in coastal ecosystems and possesses economic and ecological value. To elucidate the structural characteristics, variation, and evolution of the organelle genome of *C. stoloniferus*, we sequenced, assembled, and compared its mitochondrial and chloroplast genomes.

**Results:**

We assembled the mitochondrial and chloroplast genomes of *C. stoloniferus*. The total length of the mitochondrial genome (mtDNA) was 927,413 bp, with a GC content of 40.59%. It consists of two circular DNAs, including 37 protein-coding genes (PCGs), 22 tRNAs, and five rRNAs. The length of the chloroplast genome (cpDNA) was 186,204 bp, containing 93 PCGs, 40 tRNAs, and 8 rRNAs. The mtDNA and cpDNA contained 81 and 129 tandem repeats, respectively, and 346 and 1,170 dispersed repeats, respectively, both of which have 270 simple sequence repeats. The third high-frequency codon (RSCU > 1) in the organellar genome tended to end at A or U, whereas the low-frequency codon (RSCU < 1) tended to end at G or C. The RNA editing sites of the PCGs were relatively few, with only 9 and 23 sites in the mtDNA and cpDNA, respectively. A total of 28 mitochondrial plastid DNAs (MTPTs) in the mtDNA were derived from cpDNA, including three complete *trnT-GGU*, *trnH-GUG*, and *trnS-GCU*. Phylogeny and collinearity indicated that the relationship between *C. stoloniferus* and *C. rotundus* are closest. The mitochondrial *rns* gene exhibited the greatest nucleotide variability, whereas the chloroplast gene with the greatest nucleotide variability was *infA*. Most PCGs in the organellar genome are negatively selected and highly evolutionarily conserved. Only six mitochondrial genes and two chloroplast genes exhibited Ka/Ks > 1; in particular, *atp9*, *atp6*, and *rps7* may have undergone potential positive selection.

**Conclusion:**

We assembled and validated the mtDNA of *C. stoloniferus*, which contains a 15,034 bp reverse complementary sequence. The organelle genome sequence of *C. stoloniferus* provides valuable genomic resources for species identification, evolution, and comparative genomic research in Cyperaceae.

**Supplementary Information:**

The online version contains supplementary material available at 10.1186/s12870-024-05333-9.

## Background

*Cyperus stoloniferus* Retz., a perennial herbaceous plant belonging to the Cyperaceae family (sedges), grows primarily on coastal sand dunes and beaches. It is predominantly found in coastal areas of China, Japan, and Southeast Asia (https://www.gbif.org/species/2714571). *C. stoloniferus* exhibits thick and narrow rhizomes, creeping and interlocking growth, and high population density and is an important sand-fixing plant along the coastline [1]. *C. stoloniferus* is also an important species in coastal ecosystems with potential economic and ecological value and has been included in the Germplasm Resources of Halophytes in China (http://www.grhc.sdnu.edu.cn/info/1008/1374.htm). *C. stoloniferus* is an important medicinal plant used to treat menstrual disorders, dysmenorrhea, stomach pain, and inflammation [2, 3], and it was included in the IUCN Red List of Threatened Species in 2010. Although it has been added to this list, it is not actually threatened at this stage, so it is one of the least concerned species [4]. Currently, relatively little research has been conducted examining *C. stoloniferus*, and this greatly limits our understanding of its evolutionary characteristics and utilization.

Cyperaceae is the third largest monocotyledonous plant family with over 5,500 species. Based on morphological characteristics, such as flowers, inflorescences, spikelets, and embryos, they can be divided into 90 genera and play key roles in wetlands and alpine ecosystems [5, 6]. In recent years, based on partial nuclear DNA and plastid genes (*matK*, *rbcL*, *rps*16, etc.), studies have shown that certain species with similar morphologies may belong to different genera, whereas those with significant morphological differences may belong to the same genus. This has caused confusion in regard to species identification and sparked controversy in the taxonomy of Cyperaceae [7–9]. Therefore, more comprehensive explorations should be conducted such as HybSeq bait and targeted sequencing combined with traditional classification, to establish more accurate and reliable classification systems [10, 11].

Cyperaceae species possess adaptive characteristics such as C4 photosynthesis, dispersed centromeres, and multiple origins of holocentric chromosomes, making them ideal for studying evolutionary biology [8, 12, 13]. Plants possess three relatively independent genomes: nuclear, chloroplast (Chloroplast DNA, cpDNA), and mitochondrial (mitochondrial DNA, mtDNA). Chloroplast and mitochondrial genomes are often referred to as organelle genomes. As of 20 April 2024 the nuclear genomes of only 12 species of Cyperaceae have been reported, including seven genera of *Carex*, three genera of *Rhynchospora*, and one genus each of *Cyperus* and *Bolboschoenus* (https://www.plabipd.de/plant_genomes_pa.ep). In the NCBI database, there are over 40 complete cpDNAs of plants belonging to the family Cyperaceae, whereas mtDNAs have only been published for *C. rotundus*, *C. esculentus* [14], and *Carex breviculimis* [15]. Compared to the nuclear genome, plant organelle genomes are highly conserved, evolve rapidly, and exhibit maternal inheritance. They provide an ideal tool for tracing the origin, phylogeny, and molecular ecology [16–18]. Due to the lack of genomic data, Cyperaceae has not yet been systematically classified based on the complete organelle genome, leading to uncertainty regarding the evolutionary relationships of *C. stoloniferus*.

Therefore, we used next-generation sequencing (NGS) and third-generation sequencing (TGS) to assemble the organellar genome of *C. stoloniferus*. The structural characteristics, gene composition, repeat sequences, codon preferences, RNA editing, and sequence transfer of the mitochondrial and chloroplast genomes were compared and analysed, along with genomic collinearity, gene nucleotide diversity, and selection pressure of related species. A phylogenetic tree was constructed using the shared mitochondrial and chloroplast genes, thus providing valuable genomic resources for the classification, population genetics, and evolution of *C. stoloniferus*.

## Results

### Assembly validation, structural characteristics and gene composition of organelle genomes in *C. stoloniferus*

**Fig. 1:**
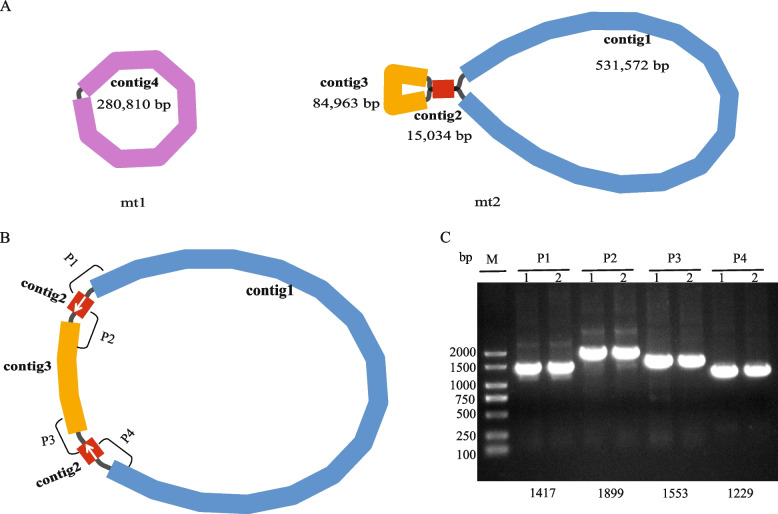
Contig assembly and PCR amplification detection of *C. stoloniferus* mtDNA. **A** The mtDNA consists of two independent circular DNAs, including mt1 and mt2. **B** mt2 consists of four contigs, among which contig2 is a 15,034bp reverse repeating direction. The counterclockwise arrow indicates a forward repeating sequence, while the clockwise arrow indicates a reverse repeating sequence. **C** Agarose gel electrophoresis of PCR amplification products of four overlapping regions (P1, P2, P3 and P4). 1 and 2 represent PCR amplification products of different DNA templates

Based on Nanopore and Illumina sequencing data and referring to the organellar genome of *C. esculentus*, we assembled the mtDNA and cpDNA of *C. stoloniferus*. Visualization results using Bandage software [19] demonstrated that mtDNA possessed two discrete DNA termed mtDNA 1 (mt1) and mtDNA 2 (mt2), respectively (Fig. 1A). mt1 is composed of only contig4 (280,810 bp) and can form a circular DNA. However, mt2 consisted of contig1 (531,572 bp), contig2 (15,034 bp), and contig3 (84,963 bp), where contig2 has overlapping regions at both ends of the sequence with contig1 and contig3, respectively. Based on this observation, we propose a possible assembly arrangement of mt2: contig1 + contig2 ( +) + contig3 + contig2 (-), where contig2 ( +) and contig2 (-) are a reverse complementary sequence (Fig. 1B). To confirm this assembly hypothesis, we designed four pairs of PCR primers for PCR amplification and Sanger sequencing of the four overlapping regions (P1, P2, P3, and P4). The four contig binding sites of mt2, PCR primers, and PCR conditions are listed in Table S1. The 1% agarose gel electrophoresis band of the PCR amplification product was consistent with the expected size (Fig. 1C and Fig. S1), and Sanger sequencing confirmed the validity of this contig combination (Fig. S2). The above results demonstrate that mt2 exhibits only one conformation; that is, the primary circular DNA is composed of contig1, contig2 (+), contig3, and contig2 (-).

**Fig. 2:**
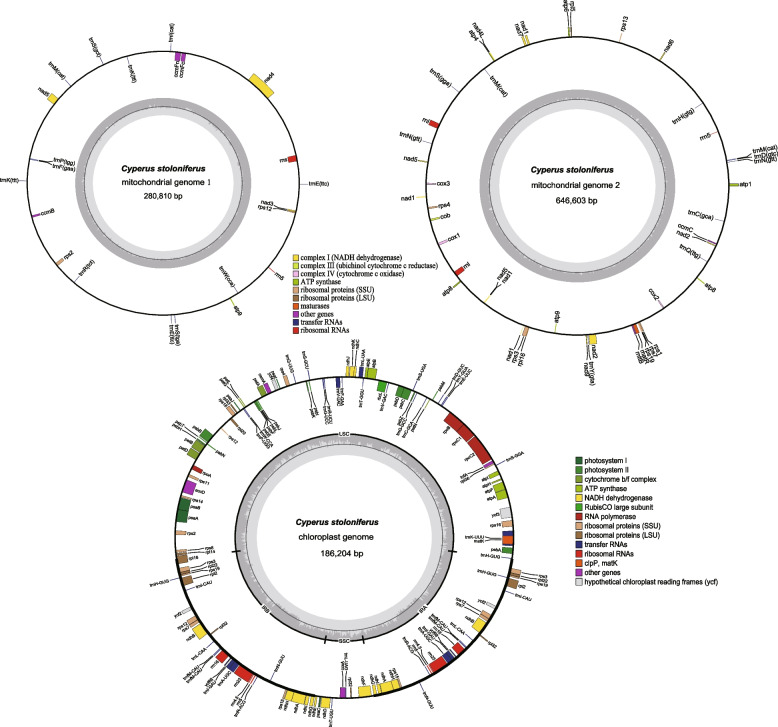
Circular DNA map of the organelle genome in *C. stoloniferus*. Different functional groups of genes are represented by different colors

The lengths of mt1 and mt2 were 280,810 and 646,603 bp, respectively, with a GC content of 40.59% (Fig. 2 and Table 1). A total of 37 protein-coding genes (PCGs), 22 tRNAs, and five rRNAs were annotated in the mtDNA of *C. stoloniferus*. *TrnE-TTC*, *trnK-TTT*, *trnM-CAT*, and a*tp8* each possessed two copies and lacked tRNAs for transporting alanine (A), valine (V), leucine (L), or threonine (T) (Table 2).

**Table 1:** The size, base content, and gene composition of organelle genomes in *C. stoloniferus*

Genome	Size (bp)	A%	T%	G%	C%	PCG	rRNA	tRNA	Accession number
mt1	280,810	29.61	29.80	20.41	20.19	9	2	12	MZ930067
mt2	646,603	29.89	29.54	20.45	20.14	28	3	10	MZ930068
cpDNA	186,204	33.20	33.61	16.23	16.96	93	8	40	MZ895087

**Table 2:** Gene composition of mtDNA in *C. stoloniferus*

Group of genes	mt1	mt2
Complex I	*nad3*, nad4^*^, nad5^*^	nad1^*^, nad2^*^, *nad4L*, *nad6*, nad5^*^, *nad7*, *nad9*
Complex III		*cob*
Complex IV		*cox1*, *cox2*, *cox3*
ATP synthase	*atp9*	*atp1*, *atp4*, *atp6*, *atp8*^(2)^, *atp9*
Ribusomal protein large subunit (LSU)		*rpl5*, *rpl16*, *rps19*, *rps7*
Ribosomal protein small subunit (SSU)	*rps2*, *rps12*	*rps1*, rps3^*^, *rps4*, *rps13*
Maturases		*matR*
Other genes	*ccmB*, *ccmFc*, *ccmFN*	*ccmC*, *mttB*
Ribosomal RNA (rRNA)	*rns*, *rrn5*	*rnl*^(2)^, *rrn5*
Transfer RNA (tRNA)	*trnE-TTC*^(2)^, *trnF-GAA*, *trnI-CAT*, *trnK-TTT*^(2)^, *trnM-CAT*, *trnP-TGG*, *trnR-TCT*, *trnS-GCT*, *trnS-TGA*, *trnW-CCA*	*trnC-GCA*, *trnD-GTC*, *trnH-GTG*, *trnM-CAT*^(2)^, *trnN-GTT*, *trnN-GTT*, *trnQ-TTG*, *trnS-GGA*, *trnY-GTA*

The superscript numbers in parentheses represent gene copy numbers, and ^*^indicates that the gene contains intron

The length of the *C. stoloniferus* cpDNA was 186,204 bp, and the GC content was 33.19%. It possessed a typical tetrad circular structure with two reverse repeat sequence regions (IRs), a large single-copy region (LSC), and a small single-copy region (SSC) with lengths of 74,842 (GC, 37.33%), 101,039 (GC, 30.93%), and 10,323 bp (GC, 25.13%), respectively. A total of 141 genes were annotated, including 93 PCGs, 8 rRNAs, and 40 tRNAs (Table S2). Among these, 24 genes possessed two copies, *rpl32* and *trnH-GUG* possessed three copies, and *trnfM-CAU* possessed four copies. The total lengths of the mtDNA and cpDNA coding sequences were 42,632 and 79,714 bp, respectively, accounting for 4.60% and 42.81% of their genomes. Non-coding sequences accounted for 95.04% and 57.09% of the total sequences, respectively (Table S3). This is similar to the proportion of non-coding sequences in the mtDNA of *C. esculentus* (95.36%) [14].

In the organelle genome of *C. stoloniferus*, 23 genes possessed introns: 18 genes had one intron, *ycf3* had two, and *nad4* had four. Simultaneously, trans-splicing was observed in *nad1*, *nad2*, *nad5*, and *rps12* (Table S4). Exons trans-splicing are derived from different pre-mRNAs, and evidence of trans-splicing introns in these genes has been reported in *Nymphaea* [20].

### Organelle genome repeat sequences

**Fig. 3:**
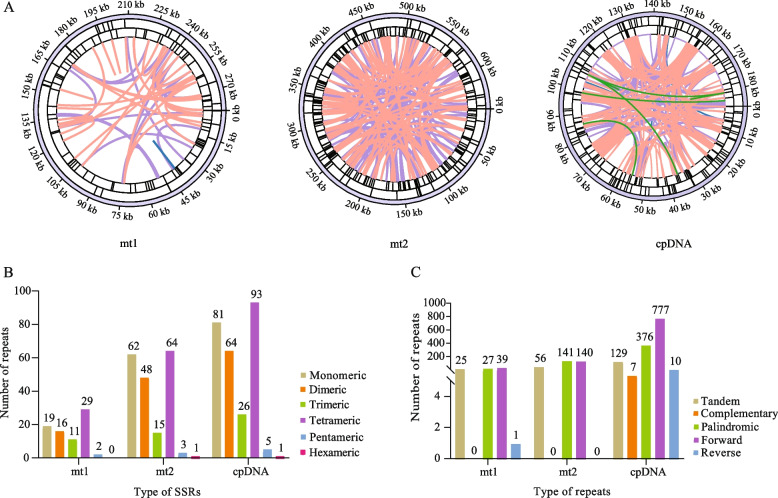
The repetitive sequence of the organelle genome in *C. stoloniferus*. **A** The short lines in the outer, middle, and inner circles represent the positions of SSRs, tandem repeats, and dispersed repeats in the organelle genome, respectively. **B** The types and quantities of SSRs. **C** The types and quantities of tandem repeats and dispersed repeats

Repetitive sequences not only play an important role in maintaining the advanced structure of the genome, but also play a crucial role in driving evolution, inducing variations, and regulating gene expression [21, 22]. Therefore, we analysed the dispersed repeats, microsatellites, and tandem repeats of the *C. stoloniferus* organelle genomes (Fig. 3A). Microsatellites, also known as simple sequence repeats (SSRs), are DNA fragments composed of short sequence repeat units with length of 1–6 base pairs distributed throughout the entire genome [23]. In this study, 270, 77, and 193 SSRs were detected in cpDNA, mt1, and mt2, respectively (Fig. 3B). The SSRs of mtDNA and cpDNA were primarily tetranucleotide repeats with the lowest number of hexanucleotide repeats. There were 29, 64, and 93 tetranucleotide repeats in mt1, mt2, and cpDNA, respectively, accounting for 37.66%, 33.16%, and 48.19% of the total number of SSRs in the genome (Tables S5-S7).

In total, 25, 56, and 129 tandem repeats were identified in mt1, mt2, and cpDNA, respectively (Fig. 3C, Tables S8-S10). The cpDNA detected 1,170 dispersed repeats, including 777 forward, 376 reverse, 7 complementary, and 10 palindromic repeats. The mt1 and mt2 contained 66 and 280 dispersed repeats, respectively. Among them, mt1 did not possess complementary repeats, while mt2 did not possess complementary and reverse repeats (Fig. 3C, Tables S11-S13). These dispersed repeats ranged from 30 to 15,034 bp. The total lengths of the cpDNA, mt1, and mt2 dispersed repeat sequences were 105,087, 2,622, and 28,346 bp, accounting for 56.44%, 0.93%, and 4.38% of the genome, respectively. These rich repetitive sequences provide important data for screening molecular markers for studying the genetic diversity of *C. stoloniferus*.

### Gene codon preference

**Fig. 4:**
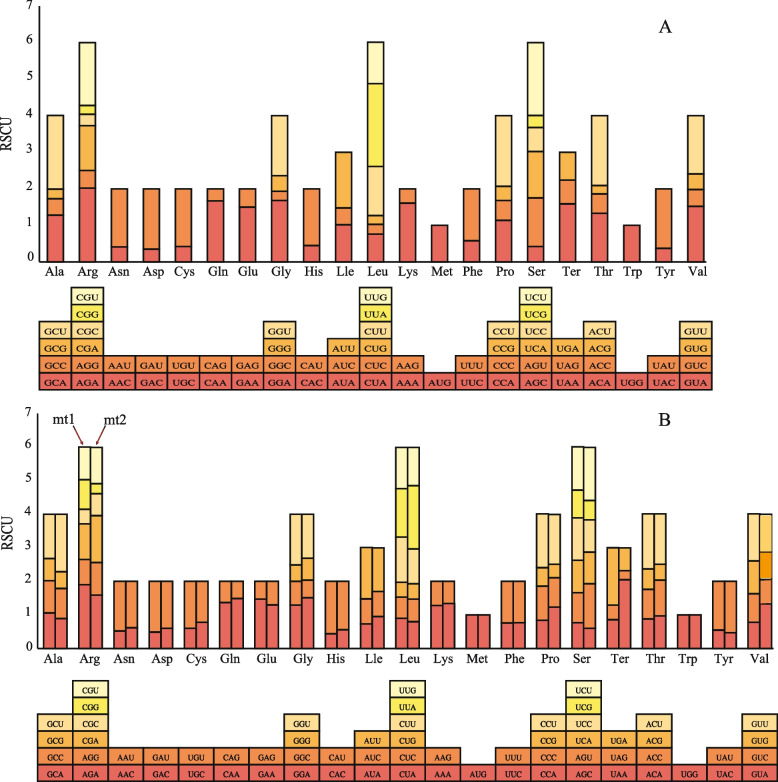
Relative synonymous codon usage (RSCU) of the organelle genome in *C. stoloniferus*. **A** RSCU analysis of cpDNA. **B** RSCU analysis of mtDNA. The color of the histogram is the same as the codon’s color

Codon preference refers to the difference in the frequency of use of degenerate codons by organisms during the translation process and the formation of a set of commonly used codons that have adapted to it during evolution, which is of great significance for gene expression [24]. Codon preference can be represented by the relative synonymous codon usage (RSCU), with RSCU values ranging from 0 to 2, where RSCU = 1 represents the expected usage frequency, RSCU < 1 indicates that the codon usage frequency is lower than the expected value, and RSCU > 1 indicates that the codon usage frequency is higher than the expected value [25]. At RSCU > 1, mt1, mt2, and cpDNA contained 26, 28, and 31 codons, respectively (Fig. 4), indicating that the organelle genes of *C. stoloniferus* prefer to use these codons. Among these high-frequency codons (RSCU > 1), the third codon position was A or U, accounting for 94.63% and 97.35% of mitochondrial and chloroplast codons, respectively. In low-frequency codons (RSCU < 1), the third codon position was G or C, accounting for 76.86% and 93.41% of the mitochondrial and chloroplast codons, respectively. This is a common characteristic of codon bias in terrestrial plant organelle genomes [26].

The most frequently used synonymous codons for mt1, mt2, and cpDNA in *C. stoloniferus* were UGA (Ter: RSCU = 1.71), UAA (Ter: RSCU = 2.05), and UUA (Leu: RSCU = 2.27), respectively. The least frequently used synonymous codons were UAG (Ter: RSCU = 0.43), UAG (Ter: RSCU = 0.27), and CUG (Leu: RSCU = 0.24), with AUG (Met) having an RSCU of = 1 (Table S14). The most frequently used codons for mtDNA and cpDNA were UUU and AUU with 445 and 815 codons, respectively. The termination codon of mt1 tended to be UGA, whereas that of mt2 and cpDNA tended to be UAA. The codon-related parameters of the organelle genome, including ENC, CAI, GC1, GC2, GC3, T3s, C3s, A3s, and G3s, are detailed in Table S15.

### RNA editing

**Fig. 5:**
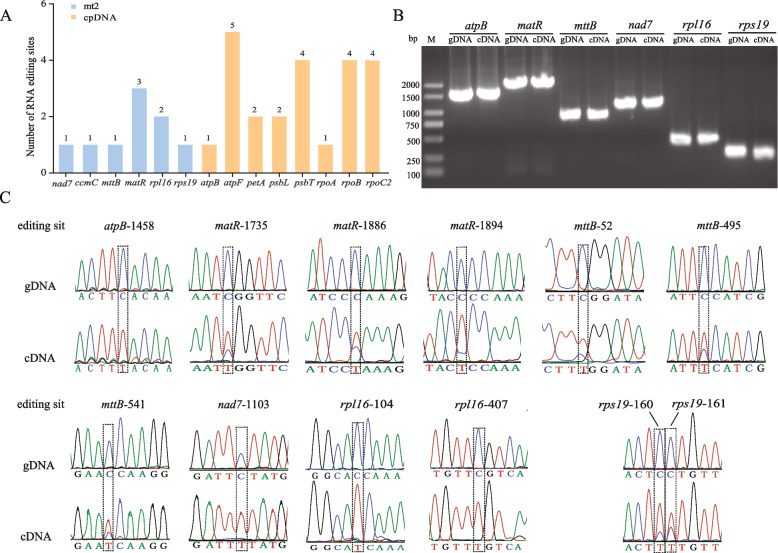
Prediction and validation of RNA editing sites in the organelle genome PCGs of C. stoloniferus. **A** RNA editing site prediction. **B** PCR amplification product electrophoresis detection. **C** Comparison of gDNA and cDNA editing sites. The RNA editing site is named "gene name", and "-" connects the position of RNA editing nucleotides in the coding region

RNA editing is the phenomenon of base insertion, deletion, or alteration that occurs during DNA transcription to form RNA in the mitochondria, chloroplasts, and nuclei [27]. By mapping transcriptome data to mtDNA and cpDNA, nine and 23 RNA editing sites were identified in the mitochondrial and chloroplast genes of *C. stoloniferus*, respectively (Fig. 5A). Six genes were detected in mtDNA that may have undergone RNA editing, including *ccmC*, *matR*, *mttB*, *nad7*, *rpl16*, and *rps19*; however, they were not detected in mt1. There are eight genes in cpDNA: *atpB*, *atpF*, *petA*, *psbL*, *psbT*, *rpoA*, *rpoB*, and *rpoC2*. Eight codons were converted to leucine, and accounted for 27% of the RNA editing sites, indicating the highest tendency for RNA editing to convert to leucine. In the mitochondria and chloroplasts, 88.89% and 78.26% were identified above the first two bases of the codon, respectively, thereby altering the corresponding amino acids (Table S16). All mitochondrial RNA editing sites were C-U-edited, whereas chloroplast C-U-edited sites accounted for 30.43% of the total. RNA editing may form termination codons, ultimately leading to premature termination of chloroplast *atpF*, *psbT*, and *rpoC2* translation. After RNA editing, 55.56% of the hydrophilic amino acids in the mitochondria were converted into hydrophobic amino acids compared with only 13.04% in the chloroplasts. Meanwhile, 30.43% of the hydrophilic amino acids in the chloroplasts were converted into other hydrophilic amino acids; however, this did not occur in the mitochondria (Table S17).

To evaluate the accuracy of predicting RNA editing sites, PCR amplification was performed using gDNA and cDNA as templates (Fig. 5B), and the Sanger sequencing results were compared (Fig. 5C and Supplementary File 1). Six genes were validated: *atpB*, *matR*, *mttB*, *nad7*, *rpl16*, and *rps19*. Among these, *atpB*, *matR*, *nad7*, and *rpl16* were consistent with the predicted results; however, no editing sites were detected for six chloroplast genes or one mitochondrial gene. In addition to *mttB*-52, two new editing sites, *mttB*-483 and *mttB*-541, were found in *mttB*, whereas *rps19* generated a new editing site *rps19*-161 on the same codon. The different sampling periods of *C. stoloniferus* leaves may be an important reason for the inconsistent RNA editing sites.

### Plastid DNA transfer

**Fig. 6:**
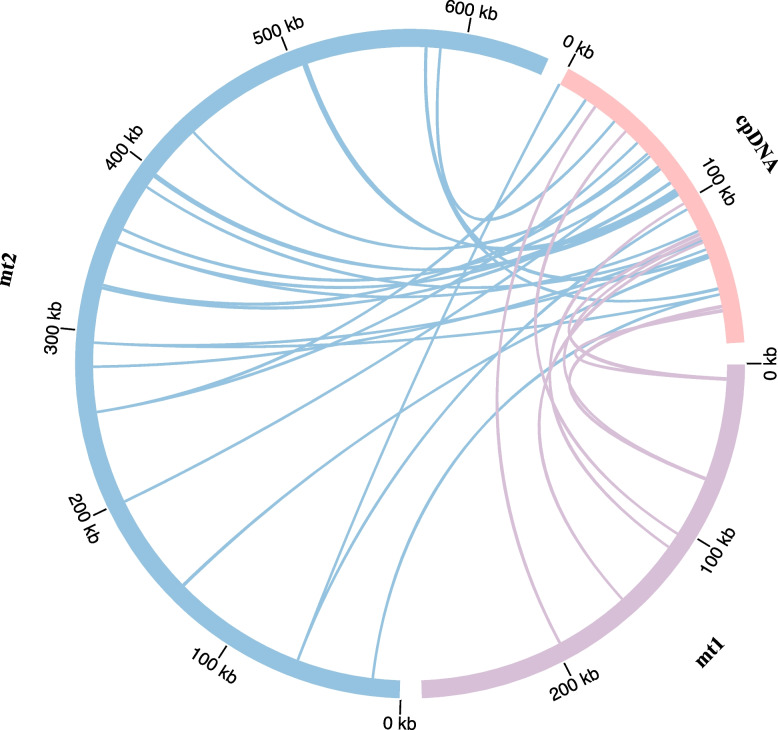
Transfer events of plastid DNA to the mitochondrial genome in *C. stoloniferus*. The outer arc represents mt1, mt2, and cpDNA, while the inner arc represents the corresponding transfer MTPTs

mtDNA generally contains sequences derived from plastid DNA, known as mitochondrial plastid DNA (MTPT) [28]. Based on nucleotide sequence similarity, 28 MTPTs were identified in the mtDNA of *C. stoloniferus* which possibly originated from cpDNA, with lengths ranging from 36 to 1,464 bp (Fig. 6). The total lengths of the MTPTs was 10,186 bp, accounting for 5.47% of the cpDNA. Among these MTPTs, 19 were chloroplast genes (most of which were gene fragments) such as *accD*, *atpA*, *ndhA*, *ndhH*, *rpoC1*, *rps12*, *rps15*, *rrn16*, and *rrn23*. Among these, only three genes were complete: *trnT-GGU*, *trnH-GUG*, and *trnS-GCU* (Table S18).

mt1 and mt2 possessed seven and 21 MTPTs, respectively, with a total length of 8,710 bp, which accounted for 0.94% of the mtDNA. Surprisingly, the chloroplast *trnT-GGU* was transferred to mtDNA and transformed into *trnM-CAT*, indicating that base mutations may occur during sequence transfer. Additionally, some small fragment sequences derived from chloroplasts were subsets of larger fragment sequences or appeared multiple times in mtDNA, indicating that these fragments may have undergone multiple independent transfer integrations, replications, and recombinations within the mtDNA after transfer integration [29].

### Phylogenetic analysis

**Fig. 7:**
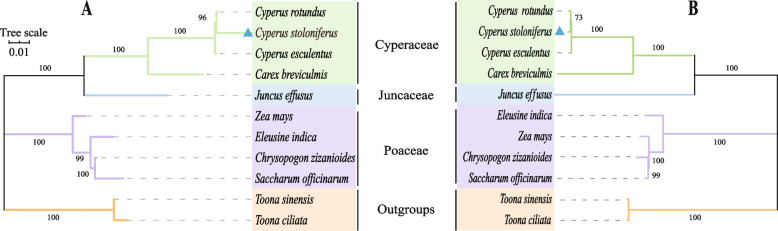
The phylogenetic relationship between *C. stoloniferus* and 10 other species. **A** and (**B**) are phylogenetic trees constructed based on genes shared by 27 mitochondria (Table S19) and 68 chloroplasts (Table S20), respectively. Colors represent plants of the same family

To identify the phylogenetic status of *C. stoloniferus*, *Toona ciliata* and *T. sinensis* from Meliaceae were used as outgroups. Based on the shared genes of the mitochondria and chloroplasts, we used the maximum likelihood (ML) method to analyse the evolutionary relationships of nine closely related species. A phylogenetic tree constructed from the 27 mitochondrial PCGs shared by the 11 plant species is shown in Fig. 7A. The results indicated that, in Cyperaceae, the closest relative to *C. stoloniferus* was *C. rotundus*, followed by *C. esculentus*. The most distant species was *C. brevicullis*. The phylogenetic tree constructed from the 68 chloroplast PCGs (Fig. 7B) indicated that the overall structures of the two phylogenetic trees were the same, thus further confirming the evolutionary relationships of these four sedge plants.

Phylogenetic analysis also indicated that Cyperaceae was closely related to Juncaceae, whereas Poaceae was more distant. Further research determined that the mitochondria in Juncaceae possess *rps10* and *rps14* which are absent in Cyperaceae and Poaceae (Fig. S3A and Table S19). In Cyperaceae and Juncaceae, chloroplasts lacked *clpP* and *ycf15*, whereas in Cyperaceae, *rpl23* was missing, but there were two *ycf68* genes (Fig. S3B and Table S20). The occurrence of loss, addition, and replication events of organelle-functional genes in the same family is consistent with the results of phylogenetic clustering [30].

### Collinearity of organelle genome in *Cyperaceae*

Analysis of the regions collinear with organelle genomes in the four sedge plants revealed numerous homologous collinear fragments. There were 62, 60, and 47 collinearity blocks with mtDNA lengths of greater than 5,000 bp between *C. stoloniferus* and *C. rotundus*, *C. stoloniferus* and *C. esculentus*, *C. esculentus* and *C. breviculis*, respectively (Table S21). There were eight, 14, and six collinearity blocks with cpDNA lengths of greater than 5,000 bp between *C. stoloniferus* and *C. rotundus*, *C. stoloniferus* and *C. esculentus*, *C. esculentus* and *C. breviculis*, respectively. However, the eight collinear blocks between *C. stoloniferus* and *C. rotundus* were > 10,000 bp long, whereas those between *C. esculentus* and *C. breviculis* were less than 10,000 bp (Table S22). Additionally, *C. stoloniferus* and *C. rotundus* were the longest among all collinearity blocks, with 53,854 (Supplementary file 2) and 47,814 bp, respectively, thus indicating that the closer the species relationship, the longer the collinearity block.

**Fig. 8:**
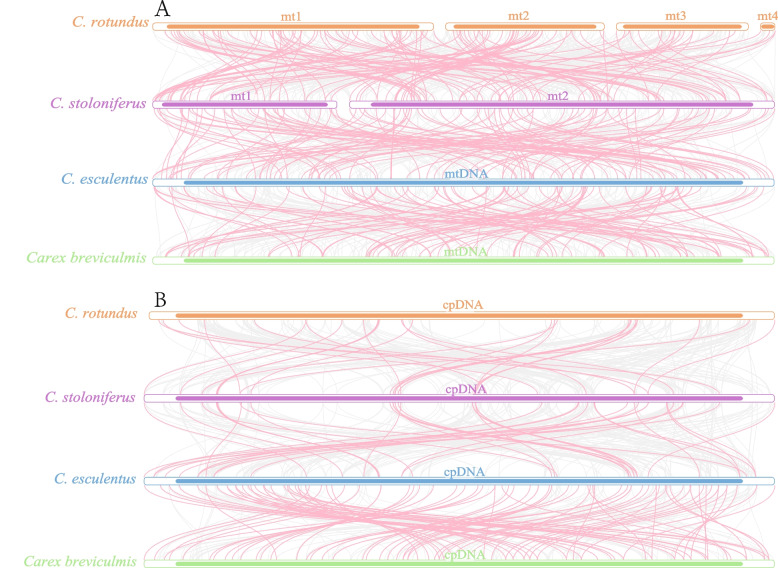
The collinear blocks between the organelle genomes of four species in Cyperaceae. **A** and (**B**) are collinear blocks of mtDNA and cpDNA, respectively. The bar chart represents the organelle genome, and the arc represents the homologous sequence of adjacent species. The red highlighted area represents homologous segments with lengths greater than 500 bp and an E values of 0, while the gray area represents homologous fragments with lengths less than 500 bp and an E values greater than 0

Meanwhile, there were differences in the collinear block arrangement positions of mtDNA (or cpDNA) in Cyperaceae, indicating that compared to closely related species, the organellar genome of *C. stoloniferus* has undergone extensive genomic rearrangement (Fig. 8). In addition, certain regions of mtDNA and cpDNA in *C. stoloniferus* do not share homology with those of other species, indicating that they existed only in the organellar genome of *C. stoloniferus*.

### Nucleotide diversity

**Fig. 9:**
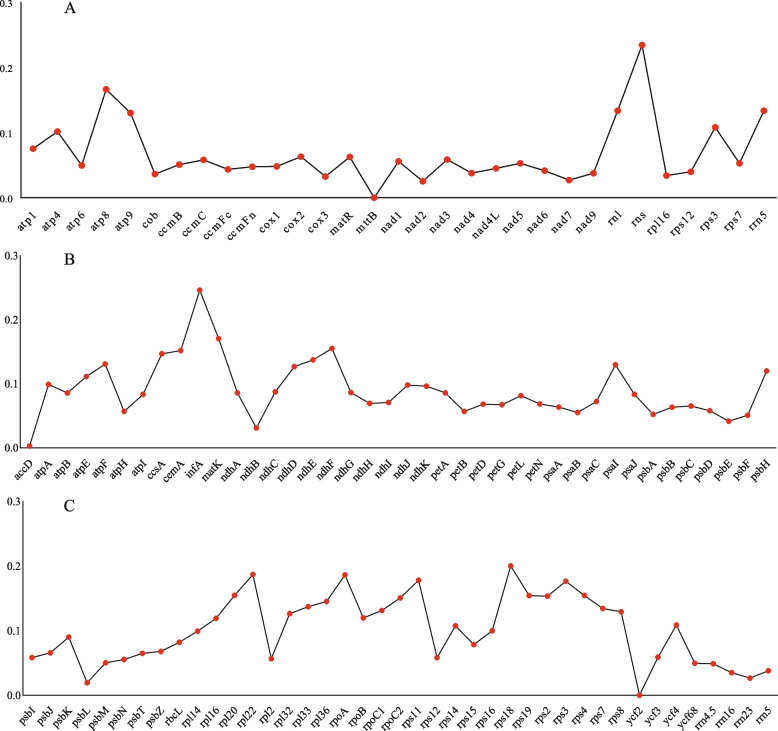
Nucleotide diversity of genes in organelle genomes of 9 closely related species. **A** The Pi value of mitochondrial genes. **B** and (**C**) indicate the Pi values of chloroplast genes

Nucleotide diversity (Pi) can be used to evaluate genetic differences in nucleotide sequences between different species and populations, and regions with high variability can be selected as potential molecular markers for populations [31]. Pi analysis of organelle genes was conducted on nine closely related plants, and the results indicated that the mitochondrial gene with the highest variability was *rns* (Pi = 0.23425). This was followed by *atp8* (Pi = 0.1664) and *mttB* (Pi = 0) (Fig. 9A and Table S23). In the mitochondrial PCGs, only seven genes exhibited Pi > 0.10, whereas the remaining 24 genes possessed Pi values ranging from 0 to 0.07535, indicating that the nucleotide sequences of most of mitochondria genes in *C. stoloniferus* were highly conserved.

The Pi values of chloroplast PCGs ranged from 0 to 0.24609, with 51 genes less than 0.10 (Fig. 9B, C, and Table S23). Among them, *infA* (Pi = 0.24609) exhibited the greatest variability, and this was followed by *rps18* (Pi = 0.20028), *rpl22* (Pi = 0.18676), and *rpoA* (Pi = 0.18614) that also exhibited greater variability. In contrast, the most conserved genes were *accD* (Pi = 0.00293) and *ycf2* (Pi = 0). Additionally, the Pi values of the four chloroplast rRNA genes were less than 0.05, whereas those of the three mitochondrial rRNA genes were greater than 0.108, indicating that the nucleotide sequence of the chloroplast rRNA gene in *C. stoloniferus* was more conserved than that of the mitochondrial.

### Ka/Ks analysis of PCGs

**Fig. 10:**
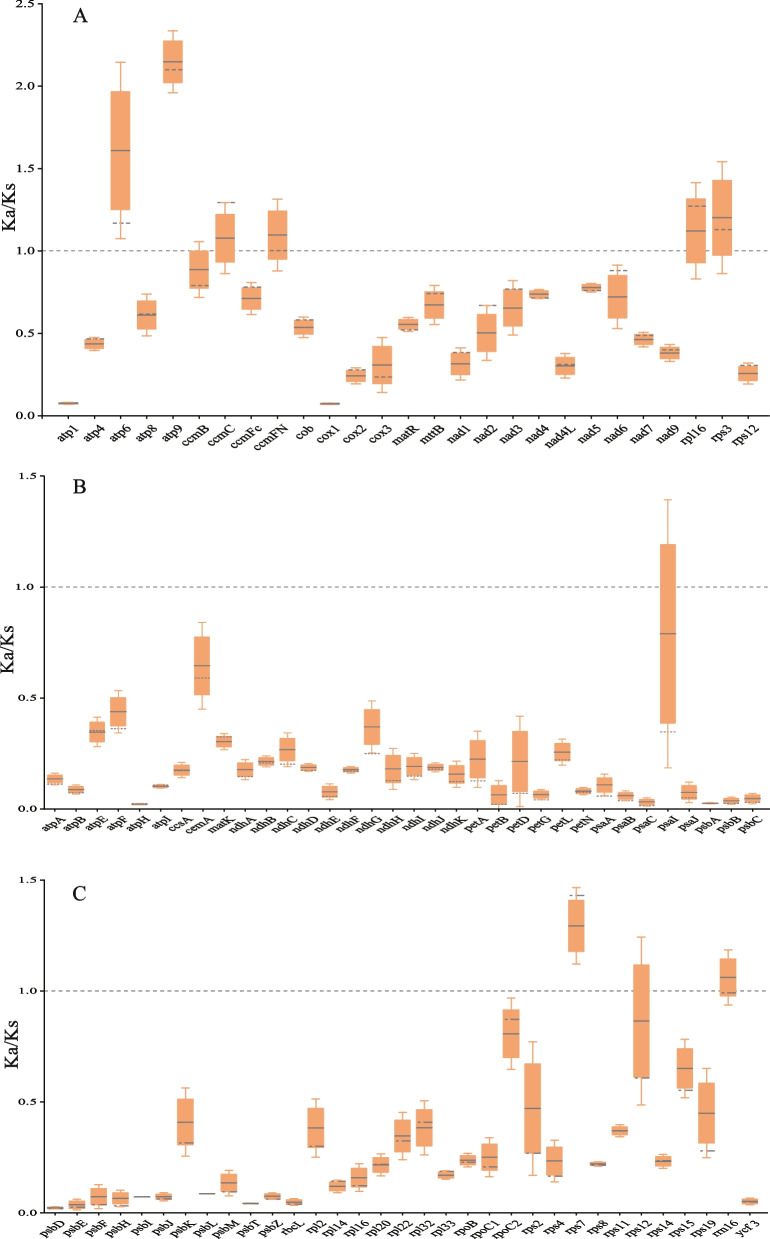
Ka/Ks analysis of PCGs in the organelle genomes of 9 closely related species. **A** The Ka/Ks of mitochondrial PCGs. **B** and (**C**) indicate the Ka/Ks of chloroplast PCGs. The black solid lines on the box plot represents the average value, and the dashed lines represents the middle value

Ka/Ks (also known as dN/dS) represents the ratio of the nonsynonymous substitution rate (Ka) to the synonymous substitution rate (Ks), which is used to measure protein selection pressure in the evolutionary process of different species [32]. When Ka/Ks > 1, genes underwent positive selection. When Ka/Ks = 1, genes underwent neutral evolution. When Ka/Ks < 1, genes were subjected to negative or purifying selection [33]. To evaluate selection pressure on PCGs in closely related plants of *C. stoloniferus*, we calculated the Ka/Ks values of 27 mitochondrial and 68 chloroplast genes. The results are presented in Fig. 10A, in which 21 mitochondrial PCGs exhibited Ka/Ks < 1, particularly *atp1* (Ka/Ks = 0.0746) and *cox1* (Ka/Ks = 0.07223) (Table S24), indicating that these genes have undergone purification selection and have relatively stable protein functions. In contrast, the average Ka/Ks values of *atp6*, *atp9*, *ccmC*, *ccmFN*, *rpl16*, and *rps3* were > 1, and *atp9* (Ka/Ks = 2.15) and *atp6* (Ka/Ks = 1.61) were strongly and positively selected. Compared with mitochondrial genes, the average Ka/Ks values of *rps7* and *rrn16* in chloroplasts were greater than 1, whereas the Ka/Ks values of the remaining 66 genes were less than 1 (Fig. 10B and C), indicating that most PCGs in chloroplasts exhibited negative selection and were highly conserved during evolution.

## Discussion

### Structural characteristics of plant organelle genome

With the rapid development of sequencing and assembly technologies in recent years, the number of high-quality organellar genome assemblies has rapidly increased. Currently, 10,123 cpDNA and 585 mtDNA sequences have been obtained from plants [34]. The structure and genetic composition of cpDNA are highly conserved. However, owing to their relatively unique genetic backgrounds and evolutionary histories, there are differences in the size of cpDNA among different species, which generally range in length from 107 to 218 kb [35]. cpDNA sizes in the *C. stoloniferus*, *C. rotundus* [36], and *C.esculentus* [37] in the genus *Cyperus* are approximately 186 kb, and their GC content is extremely similar, ranging from 33.19% to 33.26%.

Compared to cpDNA, plant mtDNA is generally larger and more complex, with not only single circular DNA, polycyclic DNA [38], and linear DNA [39], but also possibly DNA with a complex structure [40, 41]. Species such as *Camellia sinensis* [42], *Coptis deltoidei* [43], *Fallopia multiflora* [44], and *Prunella vulgaris* [45] possess two circular DNA in their mtDNA, whereas buckwheat possesses 10 [46] and *Amorphophallus albus* possesses 19 [47]. This study also confirmed that the mtDNA of *C. stoloniferus* possesses two circular DNA, whereas *C. esculentus* with a closer genetic relationship, possesses only one [14] and *C. breviculmis* with a further genetic relationship, may exhibit four different conformations [15]. Most plant mtDNAs exhibit a circular structure containing the entire genome sequence, and some homologous sequences may undergo recombination, ultimately resulting in the formation of small circular structures. Small circular, linear, and primary circular DNA may coexist in plant mtDNA [39].

### Genomic evolution

Most higher plants exhibit little or no homologous recombination in their cpDNA, and their gene composition and nucleotide sequences are conserved [48]. However, mtDNA also exhibits a highly conserved and different evolutionary rate compared to that of nuclear genes, and this can provide a large amount of classification information and can be used for the classification and identification of closely related species [49, 50]. The spikelets of Poaceae and Cyperaceae were similar. Chromosomes and pollen morphology indicate a close relationship between Cyperaceae and Juncaceae, while phylogenetic analysis suggests that Cyperaceae possesses a closer genetic relationship with Juncaceae and a farther relationship with Poaceae [51, 52]. This study also demonstrated this evolutionary relationship by constructing a phylogenetic tree based on shared genes between mitochondria and chloroplasts.

*C. rotundus* and *C. esculentus* began to differentiate approximately 5.6 million years ago, although they share very similar morphological characteristics, growth habits, habitats, and growth and development processes [53]. However, the results of the systematic evolution indicated that the phylogenetic relationship between *C. rotundus* and *C. stoloniferus* was the closest, followed by *C. esculentus*. This is the first study to elucidate the evolutionary relationships among these three sedge species. The maximum collinear region of mtDNA in Cyperaceae is approximately 53.85 kb, whereas that of cpDNA is approximately 47.81 kb. Collinear regions were observed in both *C. stoloniferus* and *C. rotundus*, further indicating a close genetic relationship. However, species with distant genetic relationships also possess smaller collinear blocks, which may be due to the highly dynamic structure of plant organelle genomes that are still evolving [54].

A large number of tandem repeats, dispersed repeats, and SSRs were detected in both the mtDNA and cpDNA of *C. stoloniferus*. Concurrently, genes such as *rns*, *atp8*, *infA*, *rps18*, *rpl22*, and *rpoA* exhibited high Pi values. These are not only important sources of information for developing populations and evolutionary analysis markers but also play an important role in genome plasticity and adaptive evolution [55]. Additionally, certain noncoding regions of cpDNA exhibit relatively high nucleotide substitution rates that are not only suitable for reconstructing phylogenetic relationships between species but also for studying phylogenetic geography within species [56, 57]. Therefore, the organellar genome contributes to genetic diversity, and lineage geography research focused on *C. stoloniferus* helps to trace the historical origins of existing distribution patterns and elucidates the impact of geological changes on the evolution of *C. stoloniferus*.

### Genomic sequence transfer

During plant evolution, mtDNA has undergone significant changes in gene sequence, genome structure, and sequence transfer from other organelles [58]. Plasmid DNA fragments are commonly transferred to mtDNA, and this frequent DNA transfer can be traced back to the common ancestor of gymnosperms and angiosperms approximately 300 MYA [28]. As evolution progresses, cpDNA gradually decreases, whereas mtDNA gradually expands because of frequent DNA exchange with the nucleus and chloroplast genome [59]. This study also determined that the mtDNA of *C. stoloniferus* is 0.927 Mb, and this is 4.98-fold larger than that of cpDNA. Plasmid transfer DNA fragments are randomly dispersed in cpDNA, with a total length of 3.19 kp in *C. esculentus* [38], 5.67 kp in *C. breviculimis* [15], and 10.19 kp in *C. stoloniferus*. It is currently the longest known plant plasmid in the family Cyperaceae and transfers plastid DNA to mtDNA.

Horizontal gene transfer (HGT) has been proposed relative to vertical gene transfer (parental transfer to offspring), and this overcomes the limitations of genetic relationships and makes gene flow more complex [60]. Due to the lack of data regarding the nuclear genome of *C. stoloniferus*, further research is required to determine if DNA transfer occurs between the nuclear genome and the mitochondrial and chloroplast genomes.

### RNA editing

Plant organelle gene expression involves many different co-transcriptional or post transcriptional nucleic acid modifications, including 5'and 3' RNA processing, cis- and trans-splicing, and RNA editing [27]. RNA editing involves the production of RNA products that differ from the DNA templates and can alter genetic information at the mRNA level. The transformation of C to U in plant mtDNA and cpDNA is the primary type of RNA editing [61]. The mitochondrial genes of *C. stoloniferus* belonged to the C-U editing type, whereas the chloroplast genes accounted for 30.43% of the total. RNA editing not only leads to changes in the encoded amino acids but may also generate termination codons, ultimately leading to premature termination of the translation process [62]. In the organellar genome of *C. stoloniferus*, this phenomenon may occur in *atpF*, *psbT*, and *rpoC2*.

The number of RNA editing sites in organellar genomes varies among species, with many gains and losses at the editing sites [63]. In terrestrial plants, the number of RNA-editing sites ranges from zero to several hundred. The number of editing sites decreases with plant evolution, and editing events occur more frequently in early differentiated plants than they do in late-differentiated plants, thus indicating that RNA editing may occur simultaneously in early differentiated plants of different branches and incur significant losses during the evolutionary process [64]. The loss of a large number of RNA-editing sites in the organelle genome of *Welwitschia mirabilis* cells may also be caused by reverse transcription processing, and a few retained editing sites may also exist in genes with lower expression levels [65]. This study also observed that the organellar of *C. stoloniferus* possess fewer editing sites, and this could be important for future research examining RNA editing in Cyperaceae.

### Gene selection pressure

A long-standing issue in evolutionary biology is how natural selection and environmental pressures shape plant genome structures. The Ka/Ks of most genes in the organelle genome of *C. stoloniferus* were less than 1, as nonsynonymous substitutions generally produce harmful traits, and only in a few cases can they lead to evolutionary advantages. This is consistent with the results of previous studies [33, 66]. This study determined that the Ka/Ks values of *atp6*, *atp9*, and *rps7* were > 1, thus indicating that these genes have undergone positive selection and are rapidly evolving, and this may be related to the adaptation of *C. stoloniferus* coastal environments. Mitochondria are the primary sites for generating the energy required for cellular activity in plants. *atp6* and *atp9* are located on the inner mitochondrial membrane and are important components of the ATP synthase complex [67]. They are potential drivers of mtDNA evolution and are often used in CMS breeding [56, 68]. Similar to the growth environment of *C. stoloniferus*, it has been observed in mangroves that only the *rps7* gene is positively selected [69]. However, whether these genes were selected under environmental stress to produce new functions for adaptation to coastal environments requires further investigation.

## Conclusion

In this study, using the Illumina and Nanopore sequencing platforms, we assembled for the first time the mitochondrial and chloroplast genomes of *C. stoloniferus*, and this is also the fourth complete mtDNA of Cyperaceae. PCR amplification and Sanger sequencing confirmed that the mtDNA of *C. stoloniferus* possessed two circular DNAs, among which mt2 possessed a 15,034 bp reverse complementary sequence, thus confirming the authenticity of the complex genomic structure of Cyperaceae. Furthermore, a comparative analysis was conducted to examine the gene composition, repeat sequences, codon preference, RNA editing, and nucleotide diversity of the organellar genome of *C. stoloniferus*. A total of 28 MTPTs were observed to originate from cpDNA with a length of 8,710 bp and accounting for 0.94% of the mtDNA, including three complete *trnT-GGU*, *trnH-GUG*, and *trnS-GCU*. The selection pressure results indicated that mitochondrial *atp6*, *atp9*, *ccmC*, *ccmFN*, *rpl16*, and *rps3* and chloroplast *rps7* and *rrn16* have undergone potential positive selection, thus revealing that these genes may play a role in the adaptation of *C. stoloniferus* to coastal environments. Genomic evolution and collinearity analyses indicated that the genetic relationship between *C. stoloniferus* and *C. rotundus* is the closest. These results will help researchers understand the characteristics of organellar genomes in Cyperaceae and lay the foundation for further elucidation of the evolutionary relationships of Cyperaceae.

## Methods

### Plant materials and DNA extracting

*C. stoloniferus* was collected from the coast of Jiangshan Town, Fangchenggang City, Guangxi Province, China (108° 33'E and 21° 68'N), by Li Donghai and identified by Professor Wang Aiqin from Guangxi University. It is currently stored in the Characteristic Plant Herbarium of Southeast Guangxi, Yulin Normal University, under plant specimen number LM202118. Using young leaves of *C. stoloniferus*, an improved CTAB method was used to extract the total DNA [70]. A NanoDrop spectrophotometre and agarose gel electrophoresis were used to assess the DNA purity, concentration, and integrity.

### Genomic sequencing, assembly, and annotation

A Nextera XT DNA Library Preparation Kit (Illumina Inc., San Diego, CA, USA) was used to construct DNA library with an average length of 350 bp. Sequencing was performed on the Illumina NovaSeq 6000 platform to generate 11.52 Gb of raw sequence data. After using NGS QC toolkit v2.3.3 [71] to remove adapter sequences and low-quality reads, we obtained 11.45 Gb in 38.16 million high-quality clean short-reads (Table S25). High-quality reads were assembled into cpDNA using the de novo assembler SPAdes v3.11.0 [72]. Finally, based on the cpDNA of *C. esculentus* (NCBI reference sequence: MW542207), the chloroplast genome of *C. stoloniferus* was annotated using PGA [73].

A long fragment DNA library was constructed using the SQK-LSK109 linker kit, and high-throughput sequencing was performed using Oxford Nanopore technology, generating a total of 13.44 Gb of raw sequencing data. After filtering and re-editing the raw reads using NanoFit and NanoPlot in Nanopack [74], a total of 12.73 Gb of clean long-reads with an average length of 9,342 bp was obtained (Table S25). The adapter sequence was trimmed using Porechop v0.2.1 [75], and a rough but computationally efficient assembly was obtained using Miniasm [76]. The assembly was then polished using Racon [77]. Referring to the mtDNA of *C. esculentus* (MW542206), potential homologous contigs were obtained using Bandage v0.8.1 [19]. Align the Nanopore reads with *C. stoloniferus* assembly draft using Minimup2 [78], and then segregated aligned reads and reassembly using Flye [79] and Canu [80], respectively. The final genome sequence was obtained by short-read polishing using Pilon [81]. The mitochondrial genome was annotated using Mitofy (http://dogma.ccbb.utexas.edu/mitof) and MFannot (https://github.com/BFL-lab/Mfanno) databases. After annotation, the Se-quin files were output, manually corrected, and submitted to the NCBI database.

### Identification of repetitive sequences

SSRs were detected using the online MISA software (https://webblast.ipk-gatersleben.de/misa), with parameter settings following Xia's method [41]. Tandem repeat sequences were identified using the online Tandem Repeat Finder software (https://tandem.bu.edu/trf) with default parameters [82]. Using online Repeater software (https://bibiserv.cebitec.uni-bielefeld.de/reputer) for dispersed repeats identification, the parameter settings refer to Xia's method [41] for analyzing the number of forward repeats, reverse repeats, complementary repeats, and palindrome repeats.

### Identification of codon preference

Using online software on the SHYCloud platform (http://www.jshycloud.net/), extracted PCGs from the chloroplast and mitochondrial genomes of *C. stoloniferus*. CodonW v1.4.2 software (https://sourceforge.net/projects/codonw) was used to analyse RSCU, T3s, C3s, A3s, G3s, CAI, CBI, and ENC of the PCGs codons. Online Cusp software (http://emboss.toulouse.inra.fr/cgi-bin/emboss/cusp) was used to analyse the GC content of the first, second, and third codons (GC1, GC2, and GC3, respectively).

### Identification of RNA editing

TopHat2 software was used to map the raw transcriptome data of *C. stoloniferus* to the organelle genome [83]. We used REDITools software to detect potential RNA editing sites in PCG with parameter settings of coverage ≥ 5, frequency ≥ 0.1, and p-value ≤ 0.5 [84]. Tablet v1.17.08.17 was used to analyse the BAM files and manually identify and remove false-positive RNA editing events [85]. To further verify the accuracy of the RNA editing sites, PCR primers (Table S26) were designed on both sides of the gene-editing site. The gDNA and cDNA synthesised from leaf RNA using random primers were used as templates for PCR amplification, and the PCR products were subjected to Sanger sequencing. RNA editing events were analysed by comparing the sequence differences between the gDNA and cDNA.

### Identification of plastid transfer sequence

Online BLAST software was used to perform homology comparisons between the cpDNA and mtDNA of *C. stoloniferus* with the parameter settings of word size 7 and E value = 1e^−5^. We analysed the homologous sequence regions and identified the sequence length, quantity, and gene types of MTPT, focusing only on sequences exceeding 35 bp and containing gene transfer sequences. Advanced Circos in the TBtools software was used to draw chloroplast and mitochondrial DNA sequence transfer maps [86, 87].

### Construction of phylogenetic tree

The mtDNA and cpDNA sequences of closely related species were downloaded from the NCBI website (https://www.ncbi.nlm.nih.gov), and 27 and 68 PCGs shared by the mtDNA and cpDNA of 11 species, respectively, were identified (Tables S19 and S20). Based on the amino acid sequences of the shared genes, the ML method of the MEGA11 software [88] was used to construct phylogenetic trees with a bootstrap value of 1,000 and an evolutionary model of GTR + I + G.

### Comparative analysis of organelle genomes in closely related species

Using BLAST software, the mtDNA and cpDNA of the four sedge plants were compared pairwise with the parameter E value of = 1e^−10^. Homologous sequences with length greater than 40 bp were screened, and the multiple synteny plot of TBtools software was used to visualize genomic collinearity regions. Using the YN model of KaKs Calculator v2.0 [32], we calculated the Ka/Ks values of the PCGs in the organelle genomes of nine closely related species through pairwise comparison. Ka or Ks values of zero, were not included in the statistics analysis. Using Mafft software for gene nucleotide sequence multiple alignment, DnaSP v5.10 software [89] was used to calculate the Pi value of the gene and visualize the calculation results as a box plot using Origin2019.

### Supplementary Information

Supplementary Material 1.

Supplementary Material 2.

Supplementary Material 3.

Supplementary Material 4.

Supplementary Material 5.

Supplementary Material 6.

## Data Availability

The raw sequencing data and assembly sequences of *C. stoloniferus* were deposited in NCBI with accession numbers PRJNA759403, SRR15684162, SRR15684161, MZ930067, MZ930068 and MZ895087, respectively. The SRA numbers corresponding to the raw transcriptome data of *C. stoloniferus* leaves were SRR27501691, SRR27501692, and SRR27501693.

## Abbreviations

cpDNA: Chloroplast genome
mtDNA: Mitochondrial genome
MTPT: Mitochondrial plastid sequence
PCG: Protein coding gene
RSCU: Relative synonymous codon usage
SSR: Simple sequence repeat
