# Supplementary material for: Assembly and comparative analysis of the complete mitochondrial and chloroplast genome of Cyperus stoloniferus (Cyperaceae), a coastal plant possessing saline-alkali tolerance
Source: BMC Plant Biol. 2024 Jul 3;24:628. doi: 10.1186/s12870-024-05333-9 (PMC11220973; doi:10.1186/s12870-024-05333-9)

**Figure S1** The original uncut electropherogram. (A) garose gel electrophoresis of PCR amplification products of four overlapping regions (P1, P2, P3 and P4). (B) Using gDNA and cDNA as templates, the organelle gene PCR amplification products of *C. stoloniferus* were detected by agarose gel electrophoresis.

**A**


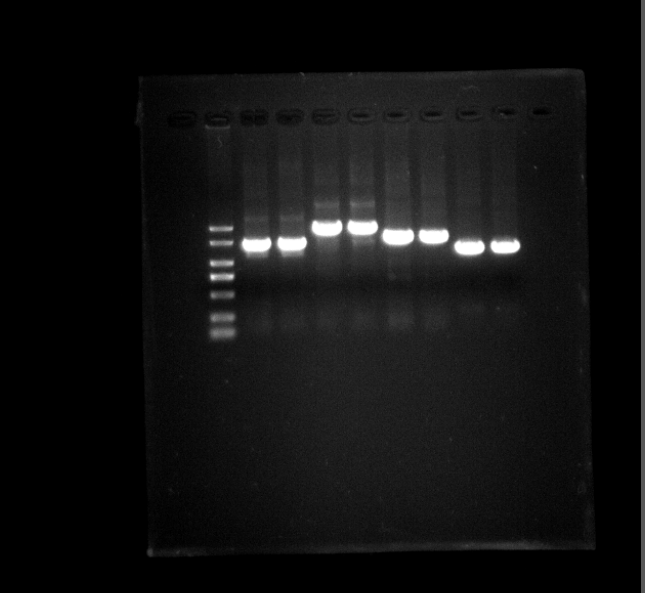


**B**


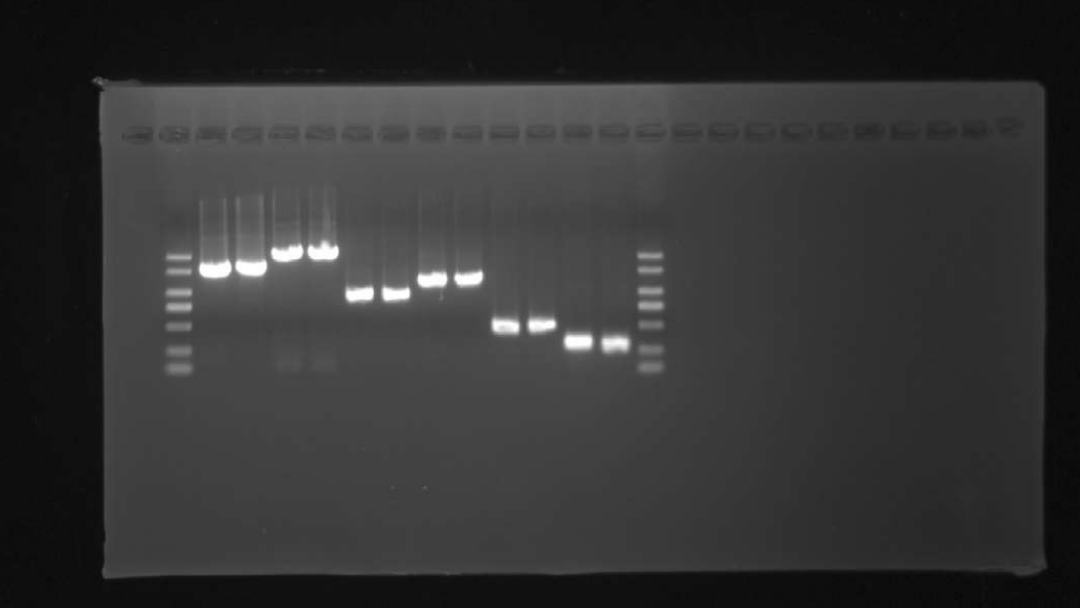

Supplement: Supplementary file 2 — Supplementary Material 2. [file 12870_2024_5333_MOESM2_ESM.docx]
