# Supplementary material for: Assembly and comparative analysis of the complete mitochondrial and chloroplast genome of Cyperus stoloniferus (Cyperaceae), a coastal plant possessing saline-alkali tolerance
Source: BMC Plant Biol. 2024 Jul 3;24:628. doi: 10.1186/s12870-024-05333-9 (PMC11220973; doi:10.1186/s12870-024-05333-9)
Supplement: Supplementary file 3 — Supplementary Material 3. [file 12870_2024_5333_MOESM3_ESM.doc]

**Figure S2** Partial sanger sequencing results of four overlapping regions in mt2

P1, P2, P3, and P4 represent the overlapping areas of contig1+contig2, contig2+contig3, contig3+contig2, and contig2+contig1, respectively. The red arrow indicates the connection point of two contigs. The PCR amplification products are directly sent to BGI for sequencing, and the sequencing primers are forward and reverse primers with overlapping regions.


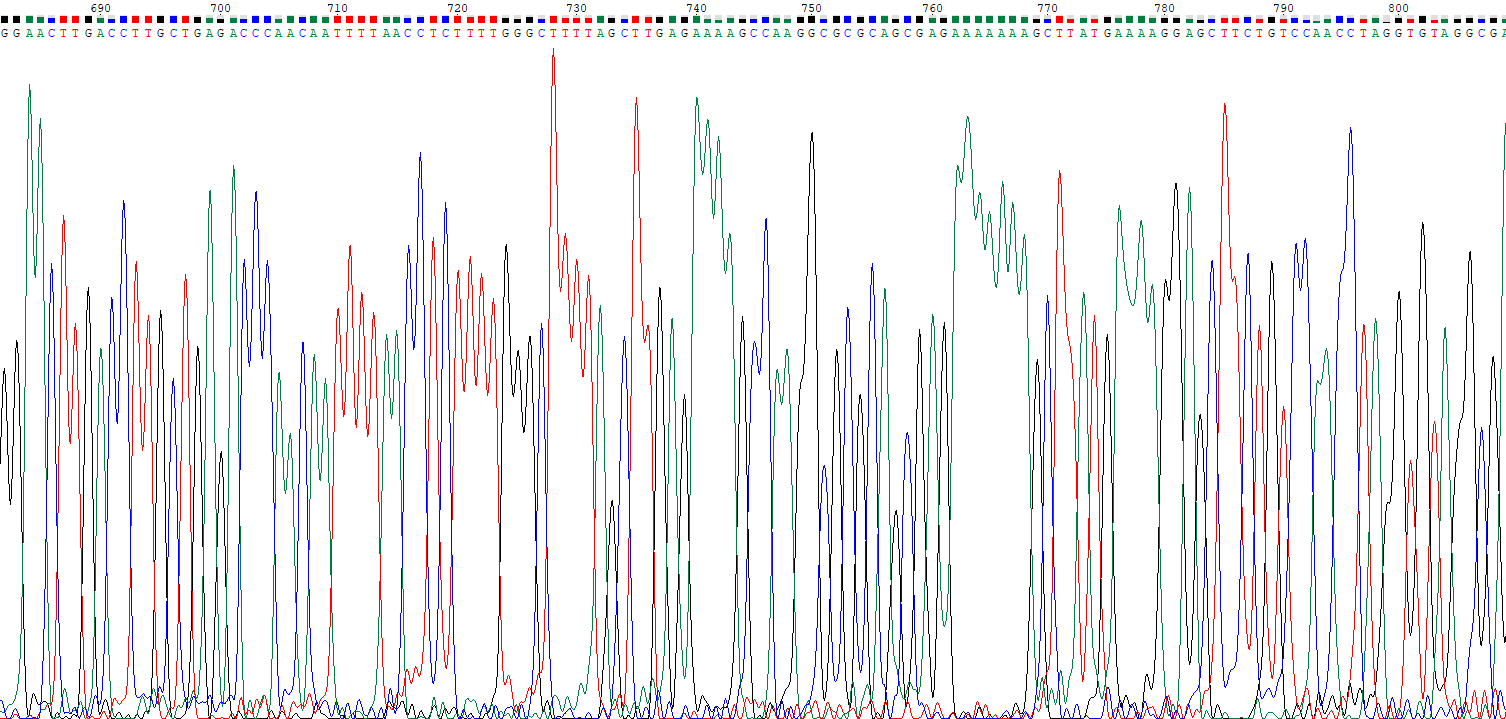


P1: Peak plot of forward primer sequencing results, with connecting sites located between the 746th and 747th bases


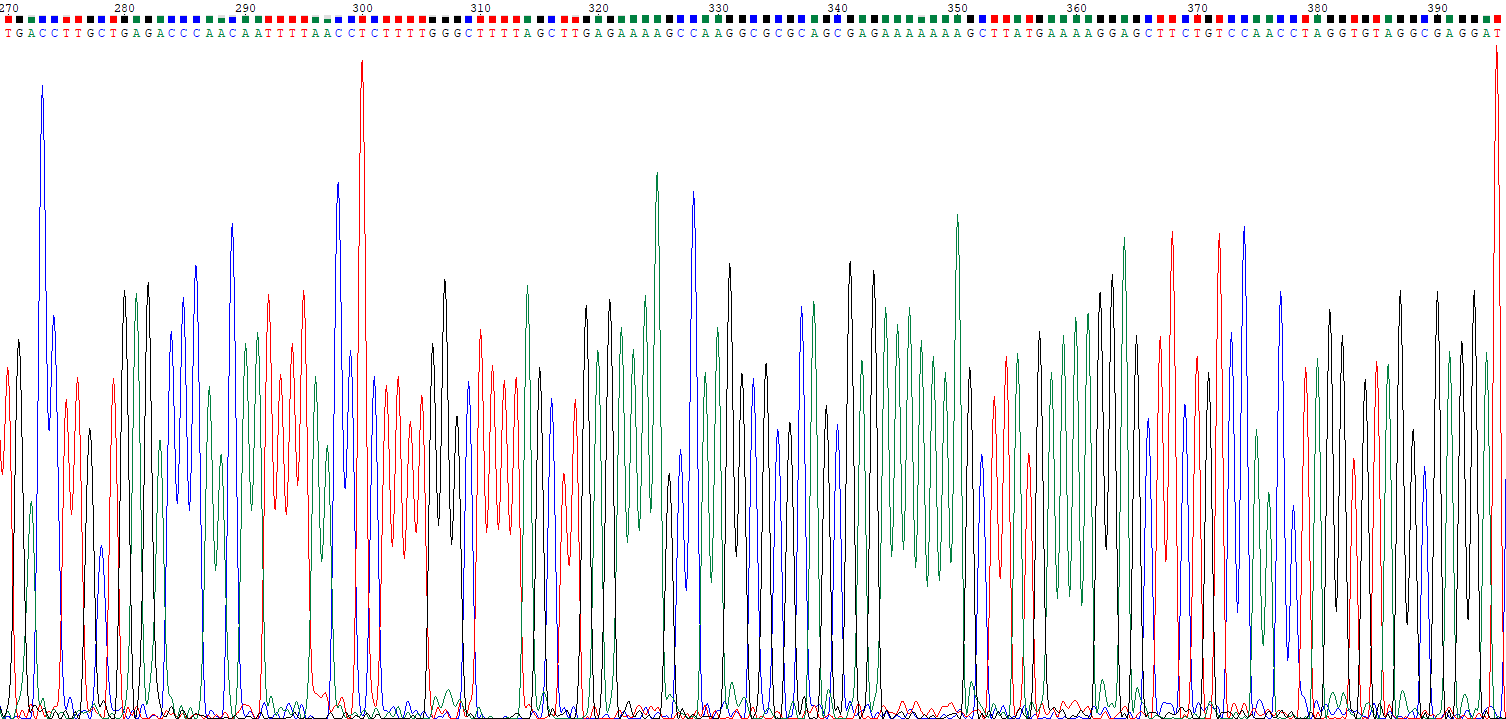


P1: Peak plot of reverse primer sequencing results, with connecting sites located between the 328th and 329th bases


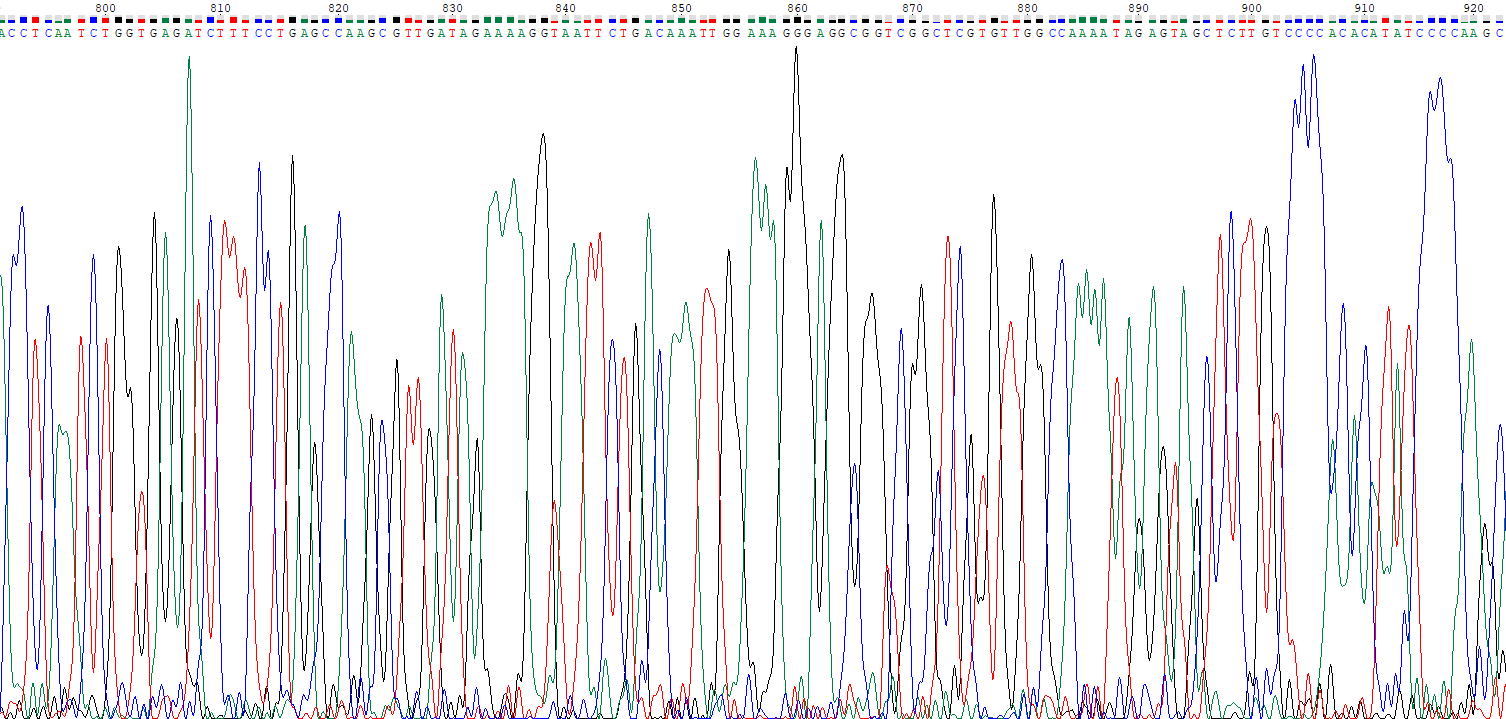


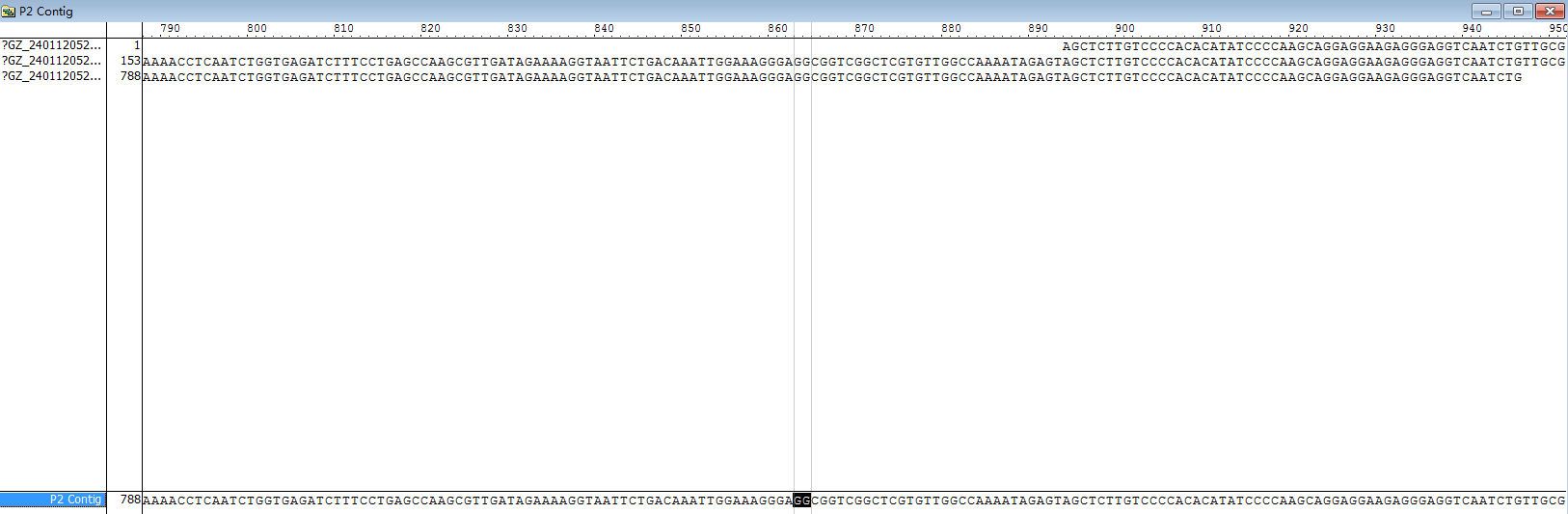


P2: Peak plot of forward primer sequencing results, with connecting sites located between the 863th and 864th bases


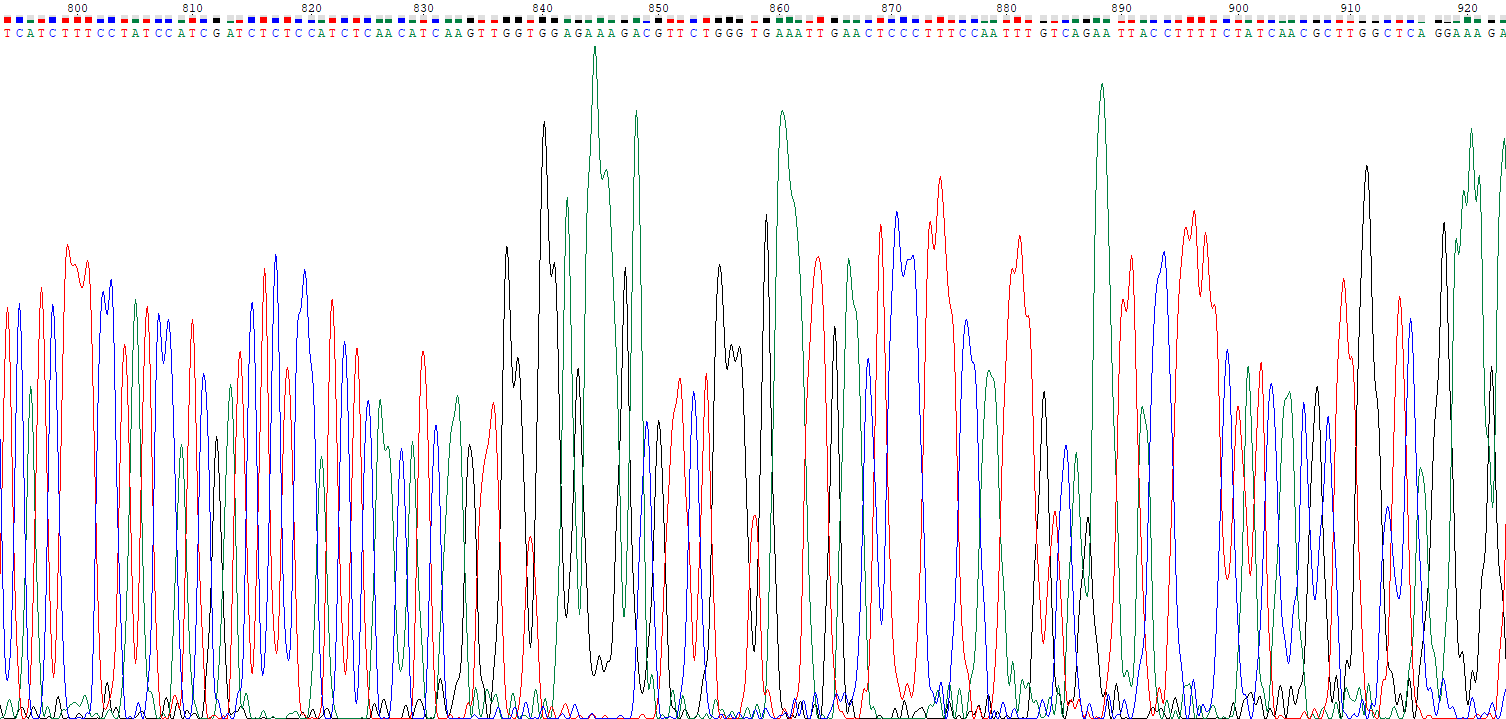


P3: Peak plot of forward primer sequencing results, with connecting sites located between the 867th and 868th bases


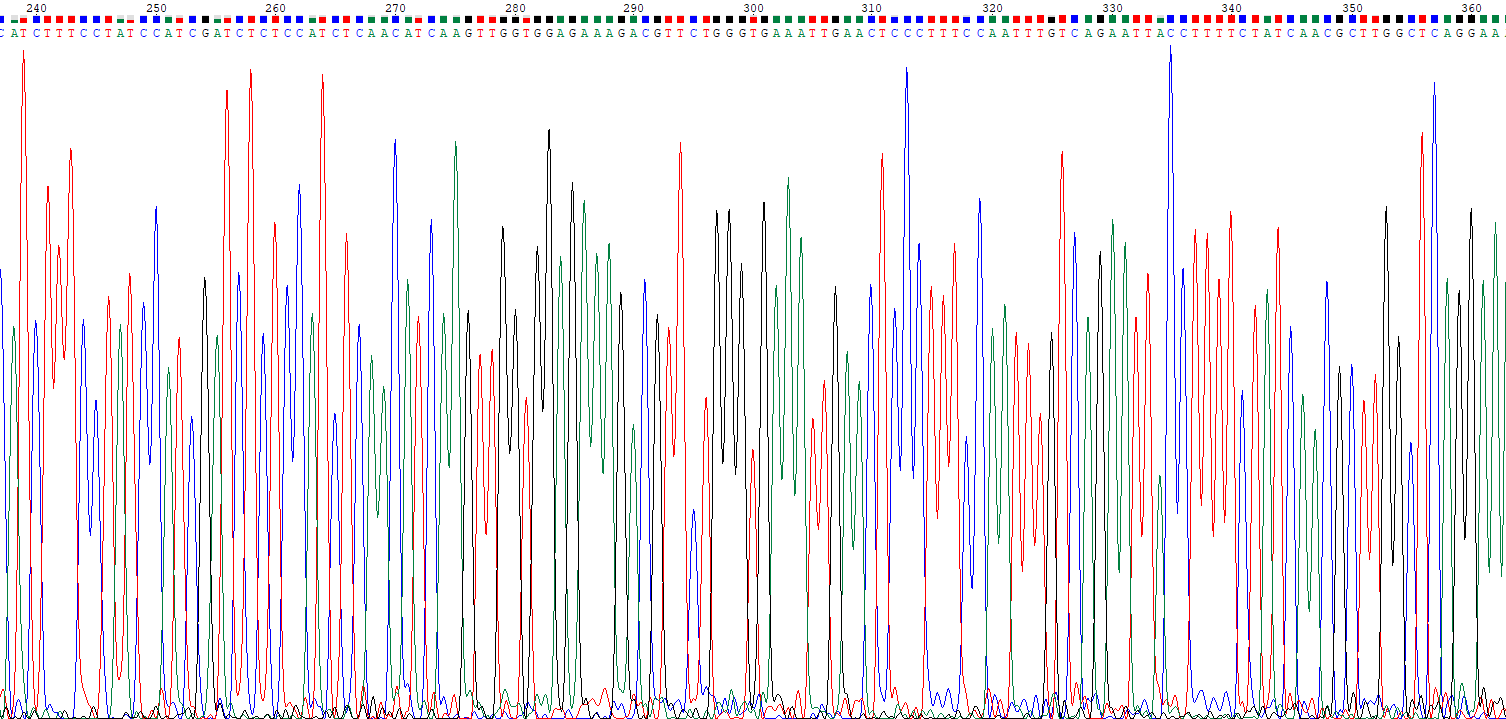


P3: Peak plot of reverse primer sequencing results, with connecting sites located between the 309th and 310th bases


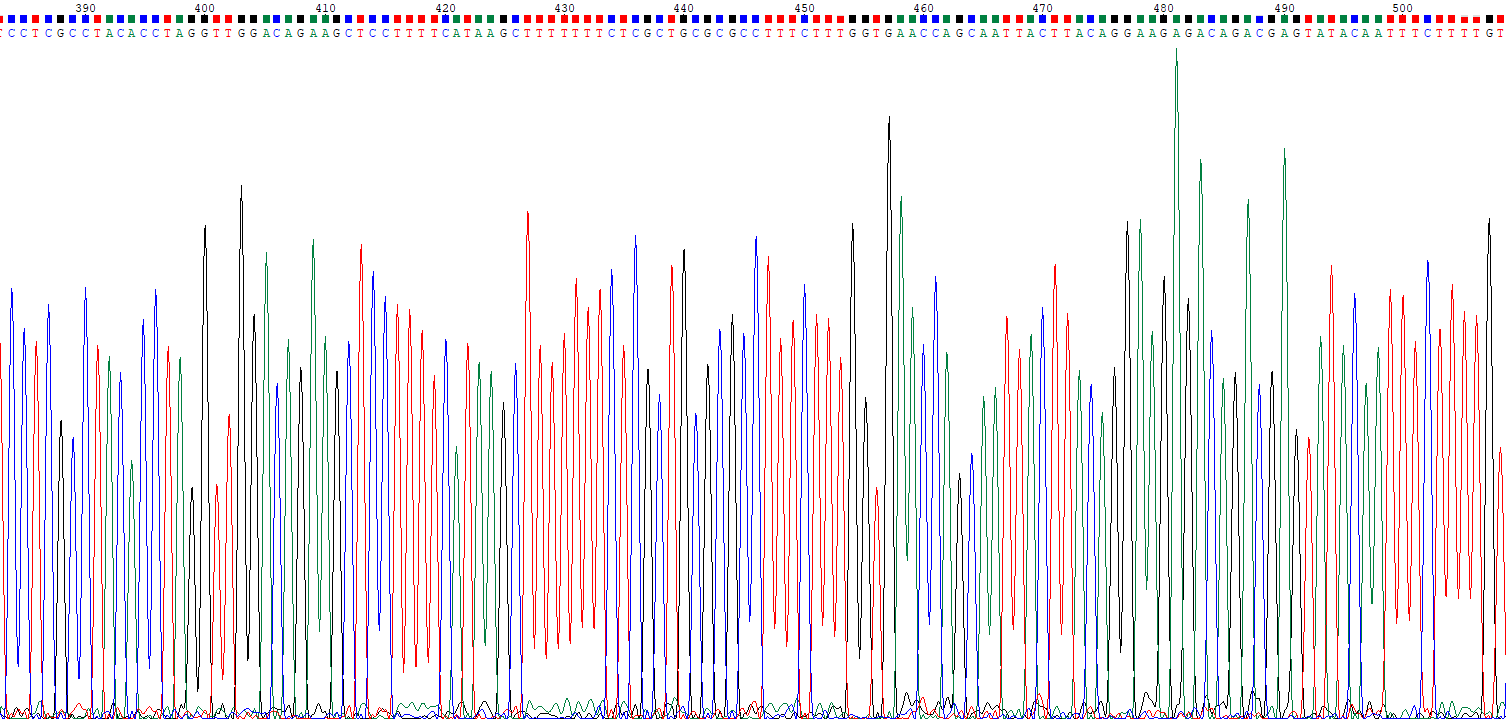


P4: Peak plot of forward primer sequencing results, with connecting sites located between the 448th and 449th bases


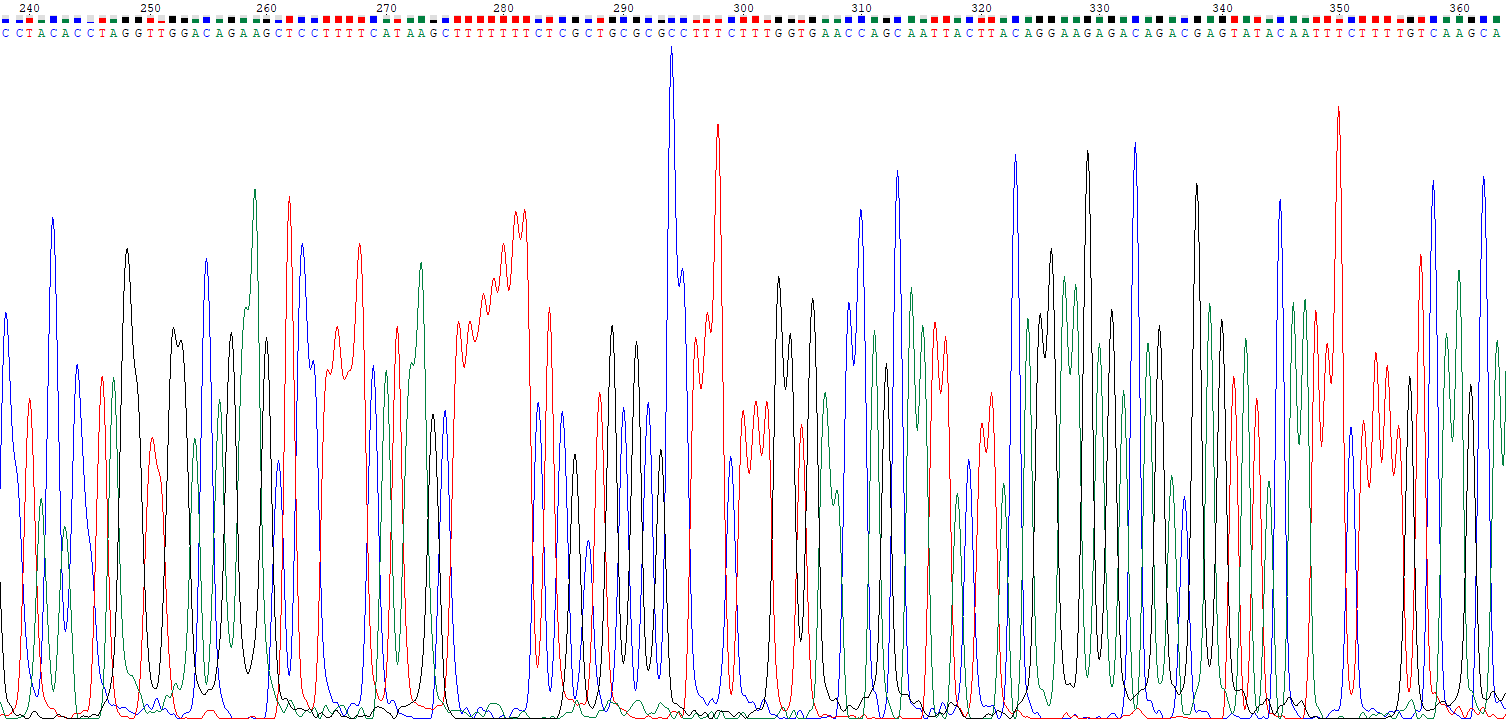


P4: Peak plot of reverse primer sequencing results, with connecting sites located between the 297th and 298th bases
