# Supplementary material for: Assembly and comparative analysis of the complete mitochondrial and chloroplast genome of Cyperus stoloniferus (Cyperaceae), a coastal plant possessing saline-alkali tolerance
Source: BMC Plant Biol. 2024 Jul 3;24:628. doi: 10.1186/s12870-024-05333-9 (PMC11220973; doi:10.1186/s12870-024-05333-9)

**Figure S3** Comparison of loss, gain, and duplication events of PCGs in chloroplast and mitochondrial genomes of 11 plant species. (A) the number of mitochondrial PCGs. (B) the number of chloroplast PCGs. Only one gene was not displayed in 11 plant species. Meanwhile, the mitochondrial *orf* gene is only abundant in maize and *Chrysopogon zizanioides*, so the *orf* gene is also partially displayed.


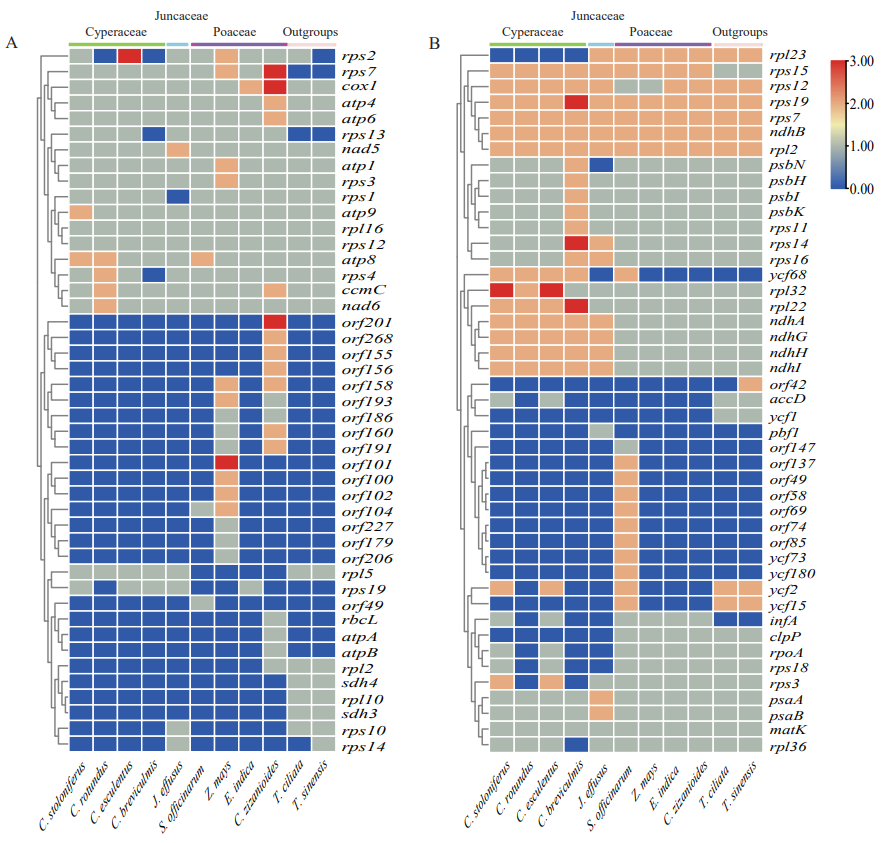

Supplement: Supplementary file 4 — Supplementary Material 4. [file 12870_2024_5333_MOESM4_ESM.docx]
