# Supplementary material for: Assembly and comparative analysis of the complete mitochondrial and chloroplast genome of Cyperus stoloniferus (Cyperaceae), a coastal plant possessing saline-alkali tolerance
Source: BMC Plant Biol. 2024 Jul 3;24:628. doi: 10.1186/s12870-024-05333-9 (PMC11220973; doi:10.1186/s12870-024-05333-9)
Supplement: Supplementary file 5 — Supplementary Material 5. [file 12870_2024_5333_MOESM5_ESM.docx]

**Supplementary file 1** Comparison of gDNA and cDNA editing sites in the organelle genome PCGs of *C. stoloniferus* based on Sanger sequencing*.* The black arrow represents gene RNA editing sites

*atpB*


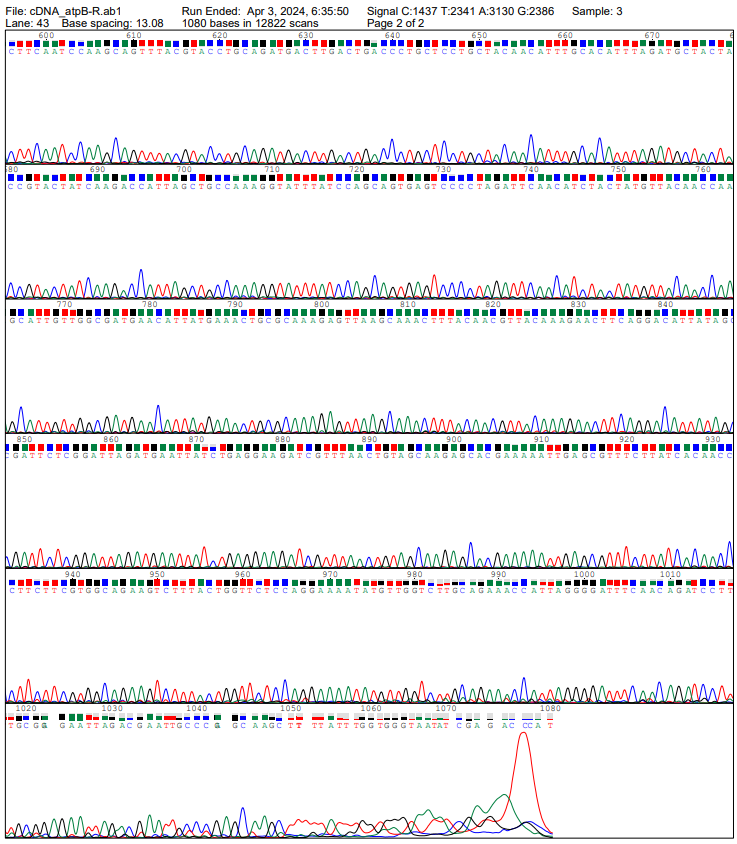


*atpB*-1458


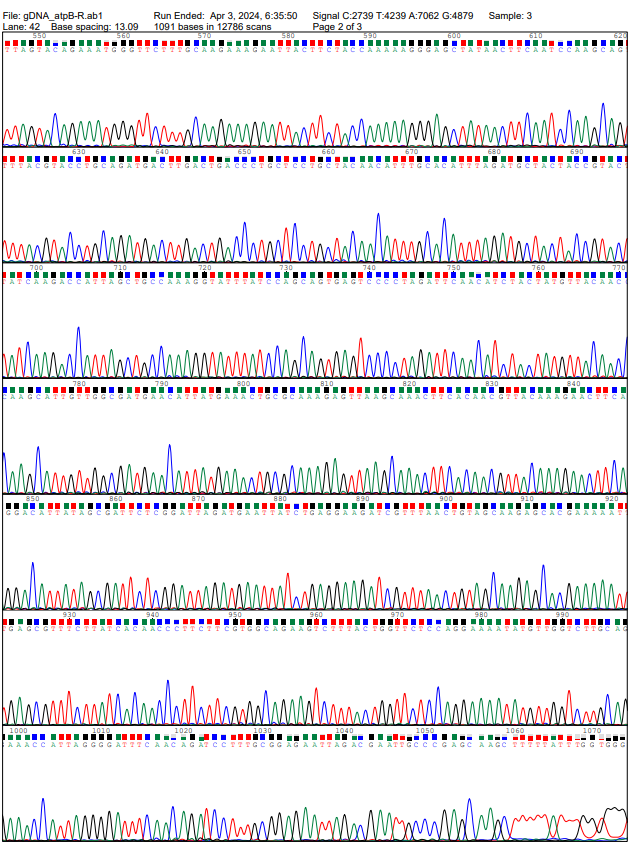


*atpB*-1458

*matR*


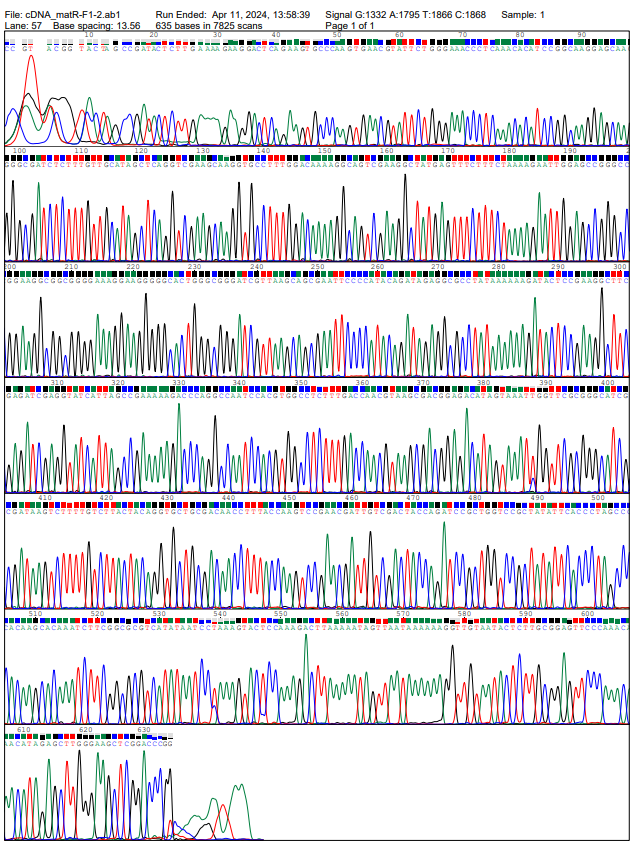


*matR*-1894

*matR*-1886

*matR*-1735


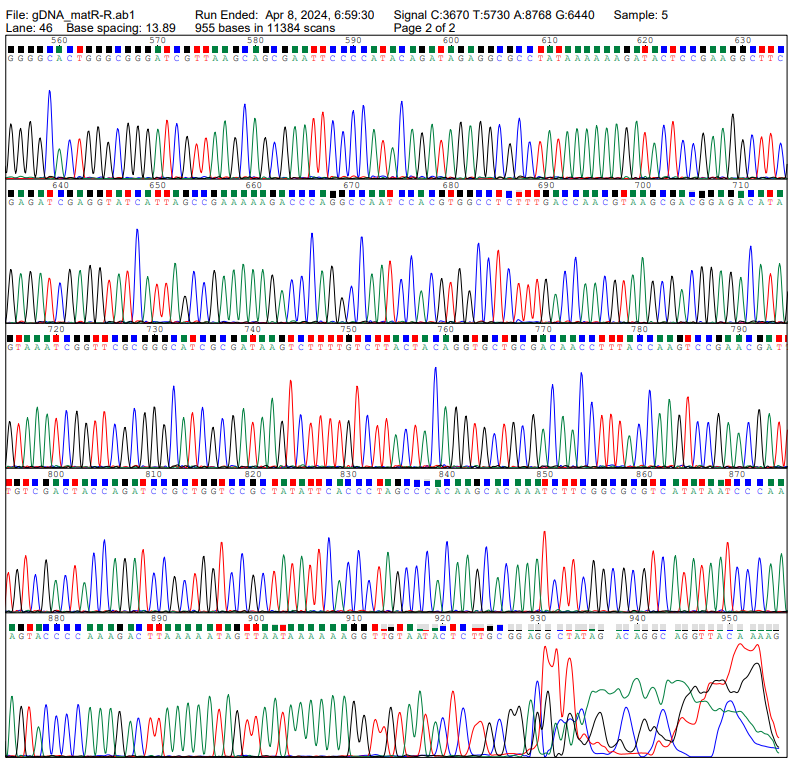


*matR*-1894

*matR*-1886

*matR*-1735

*mttB*


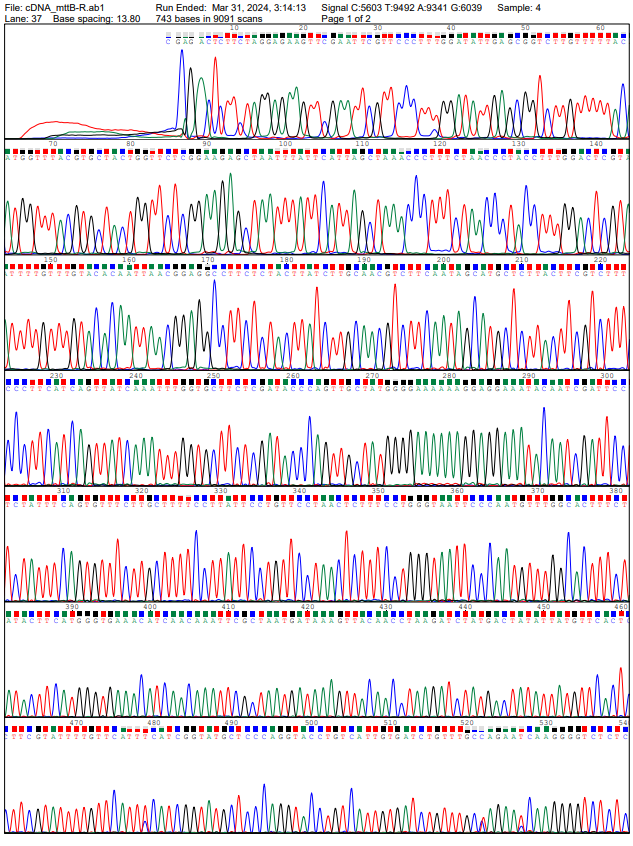


*mttB*-541

-1735

*mttB-*495

-1735

*mttB*-52

-1735


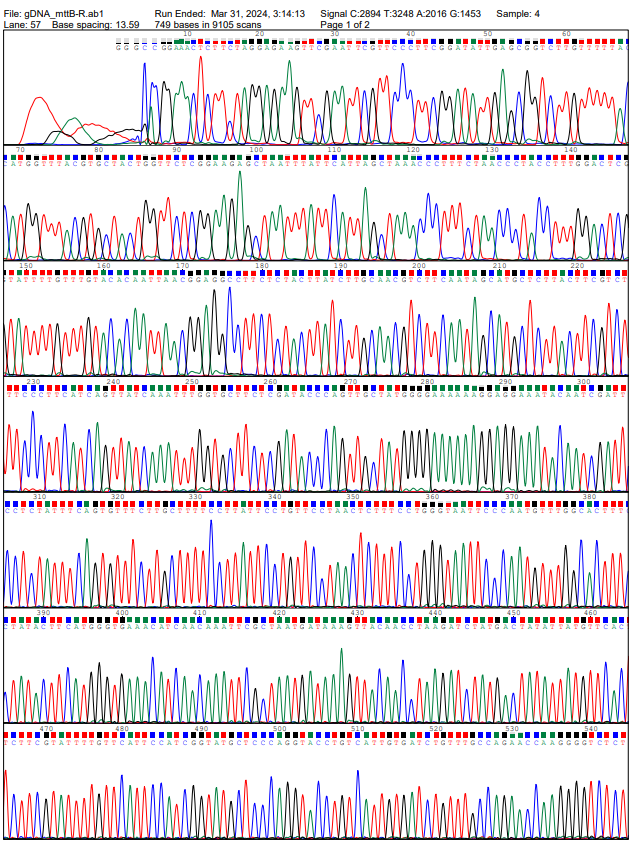


*mttB-*541

-1735

*mttB-*495

-1735

*mttB-*52

-1735

*nad7*


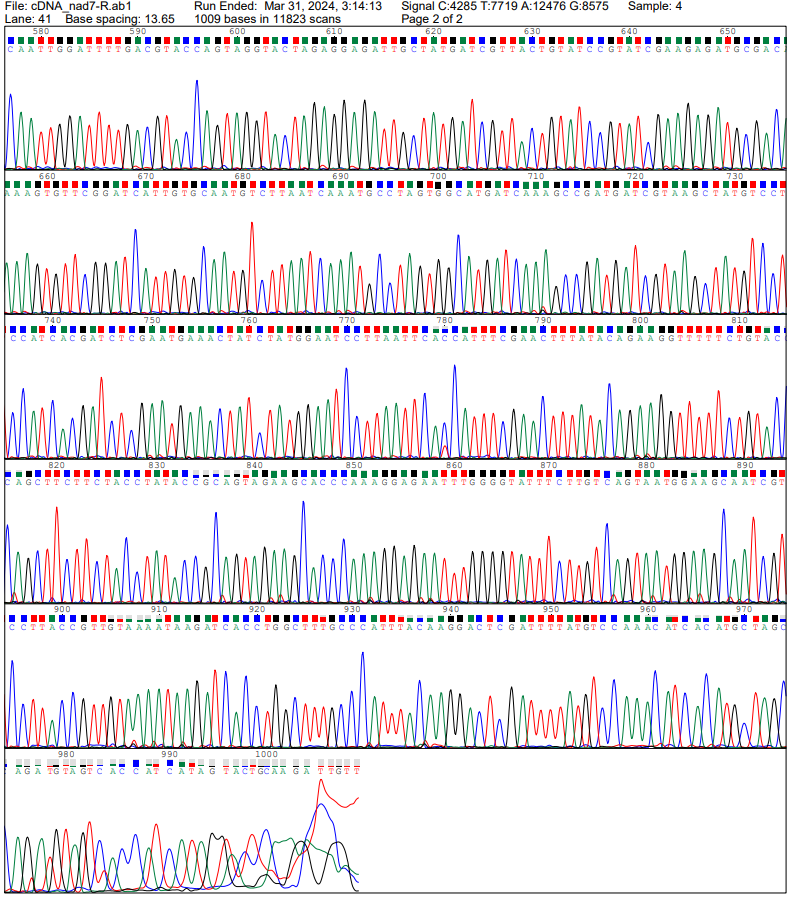


*nad7-*1103

-1735


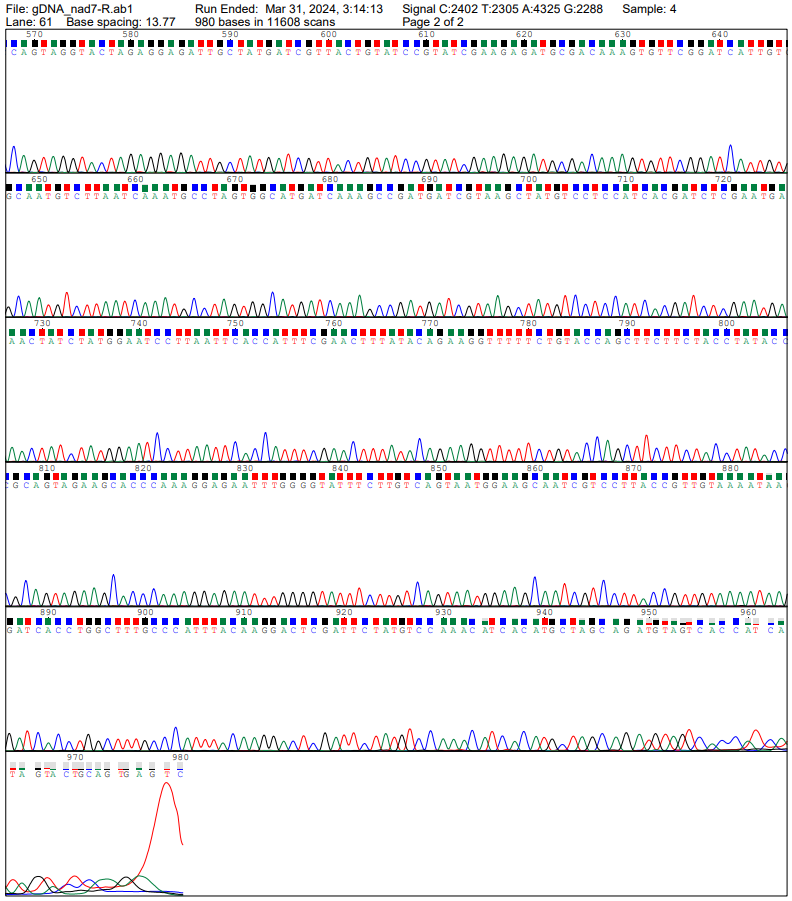


*nad7-*1103

-1735

*rpl16*


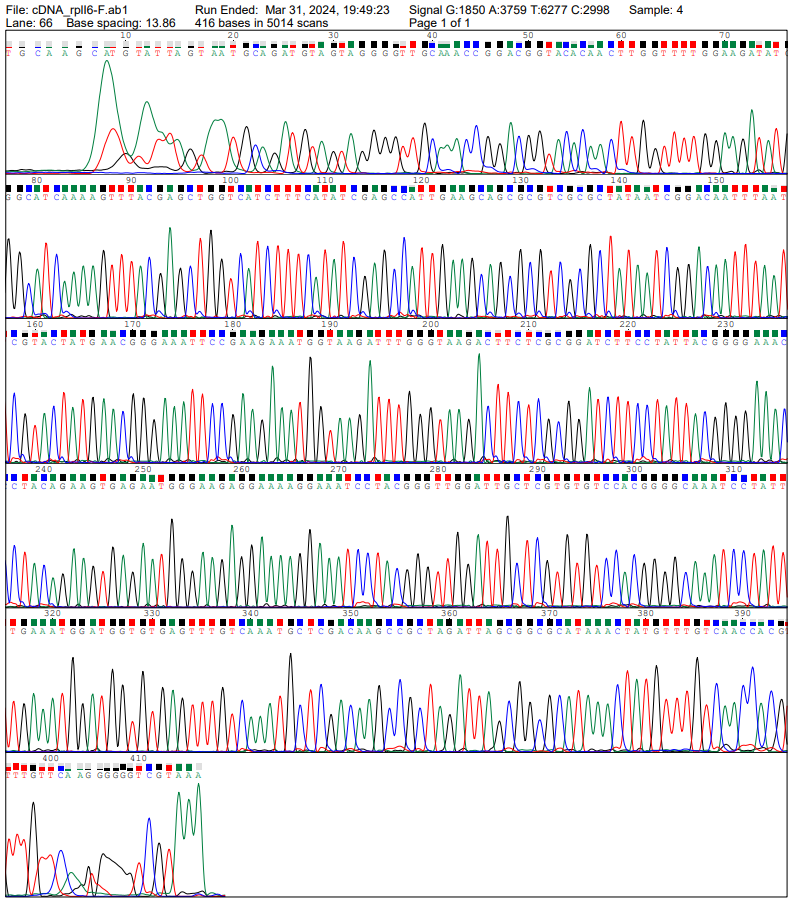


*rpl16-*1043

-1735

*rpl16-*4073

-1735


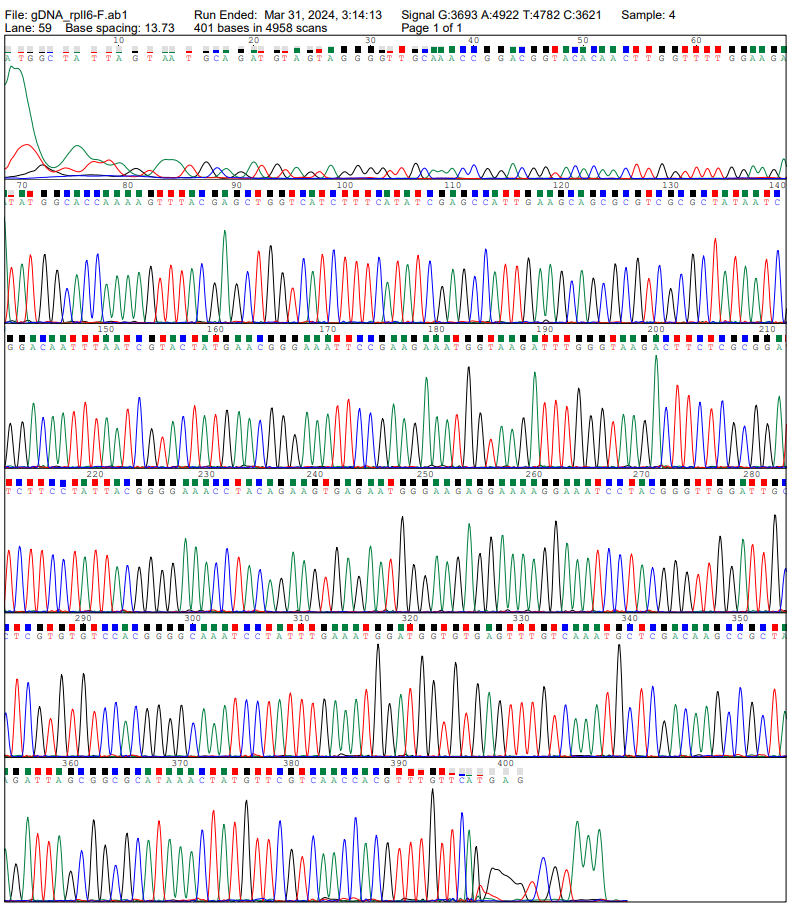


*rpl16-*4073

-1735

*rpl16-*1043

-1735

*rps19*


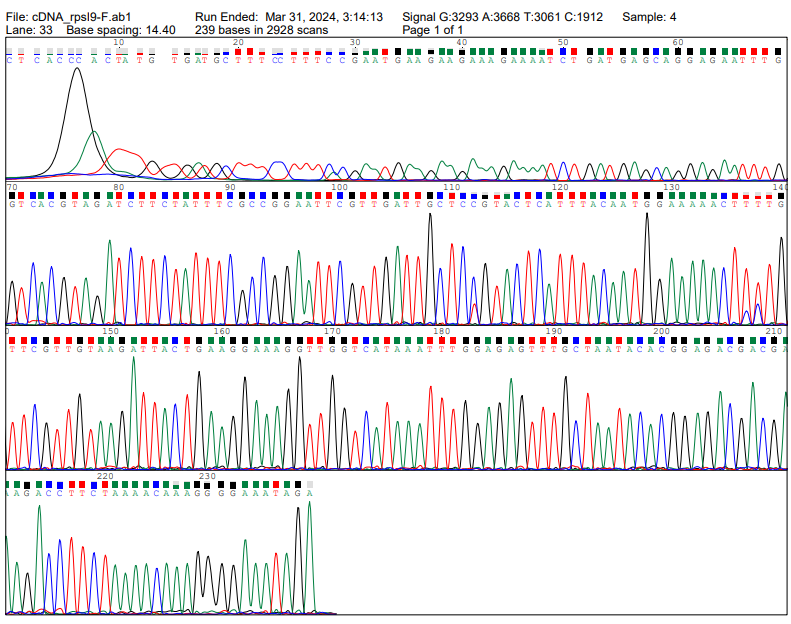


*rps19-*1603

-1735

*rps19-*1613

-1735


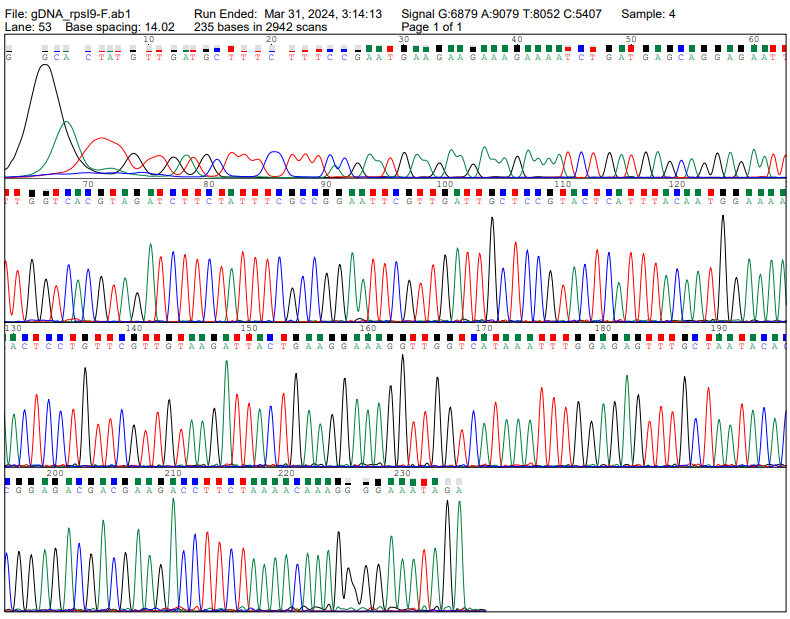


*rps19-*1603

-1735

*rps19-*1613

-1735
