# Supplementary material for: Assembly and comparative analysis of the complete mitochondrial and chloroplast genome of Cyperus stoloniferus (Cyperaceae), a coastal plant possessing saline-alkali tolerance
Source: BMC Plant Biol. 2024 Jul 3;24:628. doi: 10.1186/s12870-024-05333-9 (PMC11220973; doi:10.1186/s12870-024-05333-9)
Supplement: Supplementary file 6 — Supplementary Material 6. [file 12870_2024_5333_MOESM6_ESM.docx]

**Supplementary file 2** Blast alignment results of homologous sequences between the *C. stoloniferus* mt2 and *C. rotundus* mt1 genomes

>Query: *Cyperus stolonifer* mt2, complete sequence Query ID: MZ930068.1 Length: 646603

> Sbjct : *Cyperus rotundus* mt1, complete genome Sequence ID: OK001344.1 Length: 706916

Range 1: 188274 to 242085

Score:98615 bits(53402), Expect:0.0,

Identities:53713/53854(99%), Gaps:57/53854(0%), Strand: Plus/Plus

Query 477617 CAAGGCGCGAAGCGACAAAGGTTGAAGTCCTAGTAAACCACTTAAAAATAAAAACAACTC 477676

||||||||||||||||||||||||||||||||||||||||||| ||||| ||||||||||

Sbjct 188274 CAAGGCGCGAAGCGACAAAGGTTGAAGTCCTAGTAAACCACTTCAAAATCAAAACAACTC 188333

Query 477677 TAACAAGTTCCAAATTTCTTACTTTAAGAGTTAAGACCATGGAAACCAAGCAAATTATGT 477736

||||||||||||||||||||||||||||||||||||||||||||||||||||||||||||

Sbjct 188334 TAACAAGTTCCAAATTTCTTACTTTAAGAGTTAAGACCATGGAAACCAAGCAAATTATGT 188393

Query 477737 TATGTaaaaataaaagaaaaaagggttgcgaaaaaagtgaaaCATACCTGAACCAGGCAC 477796

||||||||||||||||||||||||||||||||||||||||||||||||||||||||||||

Sbjct 188394 TATGTAAAAATAAAAGAAAAAAGGGTTGCGAAAAAAGTGAAACATACCTGAACCAGGCAC 188453

Query 477797 TTTTCGGACTCTATACATGTTGGTGAGATCTGACATTTTTCTTTCAACCCTTAGCGGCCT 477856

||||||||||||||||||||||||||||||||||||||||||||||||||||||||||||

Sbjct 188454 TTTTCGGACTCTATACATGTTGGTGAGATCTGACATTTTTCTTTCAACCCTTAGCGGCCT 188513

Query 477857 GTTGCGTAAGCCTCTGTAACCTCCGACTGTTCAACATGAGTCGATTCTTTCTCCACCTAA 477916

||||||||||||||||||||||||||||||||||||||||||||||||||||||||||||

Sbjct 188514 GTTGCGTAAGCCTCTGTAACCTCCGACTGTTCAACATGAGTCGATTCTTTCTCCACCTAA 188573

Query 477917 TGTCCTGCCTGAAAACCTAATATTCACACAACAAATACTATACGTGTAGGGATTGGGGGT 477976

||||||||||||||||||||||||||||||||||||||||||||||||||||||||||||

Sbjct 188574 TGTCCTGCCTGAAAACCTAATATTCACACAACAAATACTATACGTGTAGGGATTGGGGGT 188633

Query 477977 GATCTGCAACGGTACTCGCCTGCCGGAGGGGTTTAGCTCATTATACGAGGGCGAAGCTGT 478036

|||||||||||||||| |||||||||||||||||||||||||||||||||||||||||||

Sbjct 188634 GATCTGCAACGGTACTTGCCTGCCGGAGGGGTTTAGCTCATTATACGAGGGCGAAGCTGT 188693

Query 478037 ATACGTTCTTCAAAGTTAAGAATGAAGTTCATTAACGGGTCATTATGCGAAGAAGCAAAG 478096

||||||||||||||||||||||||||||||||||||||||||||||||||||||||||||

Sbjct 188694 ATACGTTCTTCAAAGTTAAGAATGAAGTTCATTAACGGGTCATTATGCGAAGAAGCAAAG 188753

Query 478097 GAAGATCGTATAACGCCGCTGCTGCCCTAGCCTCTCTATAAGAAGTTCTGTCAACATAGT 478156

||||||||||||||||||||||||||||||||||||||||||||||||||||||||||||

Sbjct 188754 GAAGATCGTATAACGCCGCTGCTGCCCTAGCCTCTCTATAAGAAGTTCTGTCAACATAGT 188813

Query 478157 TAAAACCAGTAGTAGATACCAAACAAACTCCAAACACACGGTACTAATTTTCCTGGCTAA 478216

||||||||||||||||||||||||||||||||||||||||||||||||||||||||||||

Sbjct 188814 TAAAACCAGTAGTAGATACCAAACAAACTCCAAACACACGGTACTAATTTTCCTGGCTAA 188873

Query 478217 TGTCGGGTCCTCACCCCTACCCATATGTAATGGAAGAAAGCCCTGGCTTCGGTCAGCTAA 478276

||||||||||||||||||||||||||||||||||||||||||||||||||||||||||||

Sbjct 188874 TGTCGGGTCCTCACCCCTACCCATATGTAATGGAAGAAAGCCCTGGCTTCGGTCAGCTAA 188933

Query 478277 GATTCTTGCTCCCTTGCTTCGATCGATTCAATAGCAATGAAATTCATCCAAACATGCCGT 478336

||||||||||||||||||||||||||||||||||||||||||||||||||||||||||||

Sbjct 188934 GATTCTTGCTCCCTTGCTTCGATCGATTCAATAGCAATGAAATTCATCCAAACATGCCGT 188993

Query 478337 AAATCAAGAGCTGATTTCACCCGAGTCTGTACATTCGCGCGAAATCCCCTTACTTTAAGA 478396

||||||||||||||||||||||||||||||||||||||||||||||||||||||||||||

Sbjct 188994 AAATCAAGAGCTGATTTCACCCGAGTCTGTACATTCGCGCGAAATCCCCTTACTTTAAGA 189053

Query 478397 TAGTAAAAAAGCCCTTATCCTGCTGCCATGATAAATGAGAAAGTAAATAAATTAAGCAGA 478456

|||| |||||||||||||||||||||||||||||||||||||||||||||||||||||||

Sbjct 189054 TAGTCAAAAAGCCCTTATCCTGCTGCCATGATAAATGAGAAAGTAAATAAATTAAGCAGA 189113

Query 478457 AAATTCCTTTTACTTGCTAATCTAGCATCAATTCAATTTAGAGTATGGGCATTCCAGGTC 478516

||||||||||||||||||||||||||||||||||||||||||||||||||||||||||||

Sbjct 189114 AAATTCCTTTTACTTGCTAATCTAGCATCAATTCAATTTAGAGTATGGGCATTCCAGGTC 189173

Query 478517 AAAGATAGTAAAGTACTCCATCAAAACGGGCACCGCTCCCCTTATCTTCAACGACAATCT 478576

||||||||||||||||||||||||||||||||||||||||||||||||||||||||||||

Sbjct 189174 AAAGATAGTAAAGTACTCCATCAAAACGGGCACCGCTCCCCTTATCTTCAACGACAATCT 189233

Query 478577 CGAAAGGATAAAGGAAATGTGAAAGCCACTACAGTACTACTAACTAATAAGACTCGATAG 478636

||||||||||||||||||||||||||||||||||||||||||||||||||||||||||||

Sbjct 189234 CGAAAGGATAAAGGAAATGTGAAAGCCACTACAGTACTACTAACTAATAAGACTCGATAG 189293

Query 478637 ACTGCTTAAACTCCCTTTTTAATTCGGGATAGAAGATAggggggggAATAGGTCTCACCA 478696

||||||||||||||||||||||||||||||||||||||||||||||||||||||||||||

Sbjct 189294 ACTGCTTAAACTCCCTTTTTAATTCGGGATAGAAGATAGGGGGGGGAATAGGTCTCACCA 189353

Query 478697 CTCAATCTTTCATAATATCTCGGGGttttttcctttttCATTTCTTAGCGAGGATTTAAC 478756

||||||||||||||||||||||||||||||||||||||||||||||||||||||||||||

Sbjct 189354 CTCAATCTTTCATAATATCTCGGGGTTTTTTCCTTTTTCATTTCTTAGCGAGGATTTAAC 189413

Query 478757 ACACCAACCAATAGACTGCGAGGAGAGATGGCCTGCTCTACTTGACTATGGAGCGAGCGA 478816

||||||||||||||||||||||||||||||||||||||||||||||||||||||||||||

Sbjct 189414 ACACCAACCAATAGACTGCGAGGAGAGATGGCCTGCTCTACTTGACTATGGAGCGAGCGA 189473

Query 478817 AGGGGTCAATCTCTTTCTTCATAGTCTATAATTACCTCGGTTCATCGATATAATCCCGGG 478876

||||||||||||||||||||||||||||||||||||||||||||||||||||||||||||

Sbjct 189474 AGGGGTCAATCTCTTTCTTCATAGTCTATAATTACCTCGGTTCATCGATATAATCCCGGG 189533

Query 478877 AGTTAATTGAAAGGAACATTGTTTATGAAGTGGAAAGAATTACCACCTGCCTTTCTTTGA 478936

||||||||||||||||||||||||||||||||||||||||||||||||||||||||||||

Sbjct 189534 AGTTAATTGAAAGGAACATTGTTTATGAAGTGGAAAGAATTACCACCTGCCTTTCTTTGA 189593

Query 478937 CTACCTAGATATAGTAAGTAGTTCATTTCCTCTGTAGTCTAGTGAGTTTATTTGTGCGAA 478996

||||||||||||||||||||||||||||||||||||||||||||||||||||||||||||

Sbjct 189594 CTACCTAGATATAGTAAGTAGTTCATTTCCTCTGTAGTCTAGTGAGTTTATTTGTGCGAA 189653

Query 478997 ATAGATTGATTTAGCATTGAACTCTCATTTGTCTTTCTTTTTCCCTTGCTCGGATTTCCC 479056

||||||||||||||||||||||||||||||||||||||||||||||||||||||||||||

Sbjct 189654 ATAGATTGATTTAGCATTGAACTCTCATTTGTCTTTCTTTTTCCCTTGCTCGGATTTCCC 189713

Query 479057 AGAGACTAGAACATATTGATTTCCTTCAAGGCGCGGGCGGGCATAGAATATCTATAATTA 479116

||||||||||||||||||||||||||||||||||||||||||||||||||||||||||||

Sbjct 189714 AGAGACTAGAACATATTGATTTCCTTCAAGGCGCGGGCGGGCATAGAATATCTATAATTA 189773

Query 479117 CCAGGGCGGGCTTCCCGAATAGAGTATAAAGATTTGGCTCATACATACCTTGATTGAAAC 479176

||||||||||||||||||||||||||||||||||||||||||||||||||||||||||||

Sbjct 189774 CCAGGGCGGGCTTCCCGAATAGAGTATAAAGATTTGGCTCATACATACCTTGATTGAAAC 189833

Query 479177 TTTGTGAATTCAACTCTTTTGTTGTAGAAAATTTTACCTGCCTGCAGAGGAGGGAGATAT 479236

||||||||||||||||||||||||||||||||||||||||||||||||||||||||||||

Sbjct 189834 TTTGTGAATTCAACTCTTTTGTTGTAGAAAATTTTACCTGCCTGCAGAGGAGGGAGATAT 189893

Query 479237 CTCCCAGAAGTAGCTCTGTTACAAGATTTATATACACCCCCTCCCTGGAAAAGACCACCG 479296

||||||||||||||||||||||||||||||||||||||||||||||||||||||||||||

Sbjct 189894 CTCCCAGAAGTAGCTCTGTTACAAGATTTATATACACCCCCTCCCTGGAAAAGACCACCG 189953

Query 479297 GATTTACTTACTTCGGCATATTTTATTATATATATTGGTTGGAGTGTAGTGTGTGCATTT 479356

||||||||||||||||||||||||||||||||||||||||||||||||||||||||||||

Sbjct 189954 GATTTACTTACTTCGGCATATTTTATTATATATATTGGTTGGAGTGTAGTGTGTGCATTT 190013

Query 479357 TCCttttttttttAGTTGTGCTGCGTGCACTTACCAATAAGTAAGTGTAGGGCCCACCCA 479416

||||||||||||||||||||||||||||||||||||||||||||||||||||||||||||

Sbjct 190014 TCCTTTTTTTTTTAGTTGTGCTGCGTGCACTTACCAATAAGTAAGTGTAGGGCCCACCCA 190073

Query 479417 AGTAGTGATTAGCACTTCATAAGAGGAAGGCCATGGATGTACATACATAAAAGTGACTCT 479476

||||||||||||||||||||||||||||||||||||||||||||||||||||||||||||

Sbjct 190074 AGTAGTGATTAGCACTTCATAAGAGGAAGGCCATGGATGTACATACATAAAAGTGACTCT 190133

Query 479477 TTCCTAGTTAAGATAAACTTATTTAGGTAAGGGGAAAGACTGCAGTATGCGAATTTACTT 479536

||||||||||||||||||||||||||||||||||||||||||||||||||||||||||||

Sbjct 190134 TTCCTAGTTAAGATAAACTTATTTAGGTAAGGGGAAAGACTGCAGTATGCGAATTTACTT 190193

Query 479537 TCGGCAACTTTTTAATTGGAAACTCATATCCCTTGTCTGACAGCTTGCTTTACCCCGAAT 479596

||||||||||||||||||||||||||||||||||||||||||||||||||||||||||||

Sbjct 190194 TCGGCAACTTTTTAATTGGAAACTCATATCCCTTGTCTGACAGCTTGCTTTACCCCGAAT 190253

Query 479597 TGGTATGGAATTACGAATTACTCTACTTGTGGTTCATTTGCTCTTATAGTAAGGGAGGGA 479656

||||||||||||||||||||||||||||||||||||||||||||||||||||||||||||

Sbjct 190254 TGGTATGGAATTACGAATTACTCTACTTGTGGTTCATTTGCTCTTATAGTAAGGGAGGGA 190313

Query 479657 GTTACTAGACTTATAAAAGGAAGAAAAAGAACAGTTGCGTTGAGCGGTGAGCGCTTGGGG 479716

||||||||||||||||||||||||||||||||||||||||||||||||||||||||||||

Sbjct 190314 GTTACTAGACTTATAAAAGGAAGAAAAAGAACAGTTGCGTTGAGCGGTGAGCGCTTGGGG 190373

Query 479717 TTGAAGTATCGTGCTACCTGGCGAATGGGCAGCACAAGACCTAGGTAGAAGAAGAGAGaa 479776

||||||||||||||||||||||||||||||||||||||||||||||||||||||||||||

Sbjct 190374 TTGAAGTATCGTGCTACCTGGCGAATGGGCAGCACAAGACCTAGGTAGAAGAAGAGAGAA 190433

Query 479777 aaaaaGTTTGACGCGTTGTCGAGATTCAGAGACATGGAGAATATGGGATTTGGGATTGAC 479836

||||||||||||||||||||||||||||||||||||||||||||||||||||||||||||

Sbjct 190434 AAAAAGTTTGACGCGTTGTCGAGATTCAGAGACATGGAGAATATGGGATTTGGGATTGAC 190493

Query 479837 GTCGGAGGTGGTTCGTTGGATTCGGTTTGCGGGCAAGAGAAGACATGCGGAGAACGAGAC 479896

||||||||||||||||||||||||||||||||||||||||||||||||||||||||||||

Sbjct 190494 GTCGGAGGTGGTTCGTTGGATTCGGTTTGCGGGCAAGAGAAGACATGCGGAGAACGAGAC 190553

Query 479897 TCGCTCAAAGCTTACCAAAGGCACCCGCCTCGCATTCTCTCTACTCAGCTGACATCAGCA 479956

||||||||||||||||||||||||||||||||||||||||||||||||||||||||||||

Sbjct 190554 TCGCTCAAAGCTTACCAAAGGCACCCGCCTCGCATTCTCTCTACTCAGCTGACATCAGCA 190613

Query 479957 CCTCAGGAATCCCCTATTCAATCCCTGCAAACTTCAGCACCTATCTCAAGATCTGACTCA 480016

||||||||||||||||||||||||||||||||||||||||||||||||||||||||||||

Sbjct 190614 CCTCAGGAATCCCCTATTCAATCCCTGCAAACTTCAGCACCTATCTCAAGATCTGACTCA 190673

Query 480017 AGAATGACGAACTCAGATAATGCTGACGAGTATGCAATGCTTCGCTTCGTCTCTAACAAC 480076

||||||||||||||||||||||||||||||||||||||||||||||||||||||||||||

Sbjct 190674 AGAATGACGAACTCAGATAATGCTGACGAGTATGCAATGCTTCGCTTCGTCTCTAACAAC 190733

Query 480077 GGTAACCGCGACCTTCAAGAAACTCTCAATAGGAGTGTTGTAATCACCGATGAGCGGAGA 480136

||||||||||||||||||||||||||||||||||||||||||||||||||||||||||||

Sbjct 190734 GGTAACCGCGACCTTCAAGAAACTCTCAATAGGAGTGTTGTAATCACCGATGAGCGGAGA 190793

Query 480137 ATGGGGGCACTATACATCCAAGCACATTTCACAAGGGTGTTCCCGATCTCAGGAGGTTTC 480196

||||||||||||||||||||||||||||||||||||||||||||||||||||||||||||

Sbjct 190794 ATGGGGGCACTATACATCCAAGCACATTTCACAAGGGTGTTCCCGATCTCAGGAGGTTTC 190853

Query 480197 CAATGGATAGCTCGATCCATTCCTAACAACCGATTCTTGATAGATCCACCGAACGAACCC 480256

|||||||||||||||||||||||||||||||||||||||| |||||||||||||||||||

Sbjct 190854 CAATGGATAGCTCGATCCATTCCTAACAACCGATTCTTGAGAGATCCACCGAACGAACCC 190913

Query 480257 TGAGTGGAGAGCAACTGCGCTTCAACATGGATTCATCATCATGGGAGGGATACGATTCCC 480316

||||||||||||||||||||||||||||||||||||||||||||||||||||||||||||

Sbjct 190914 TGAGTGGAGAGCAACTGCGCTTCAACATGGATTCATCATCATGGGAGGGATACGATTCCC 190973

Query 480317 TATAGCTGAATAAAATAAAATCTCCAACTCCACGATGTCAGACAGAGGCCATAGACCTAT 480376

||||||||||||||||||||||||||||||||||||||||||||||||||||||||||||

Sbjct 190974 TATAGCTGAATAAAATAAAATCTCCAACTCCACGATGTCAGACAGAGGCCATAGACCTAT 191033

Query 480377 TCCTCTGTGGATACTGATAAGAGGTTTGTTTACCCTTTCGCTTTTTCAAAGAAGTAGAGT 480436

||||||||||||||||||||||||||||||||||||||||||||||||||||||||||||

Sbjct 191034 TCCTCTGTGGATACTGATAAGAGGTTTGTTTACCCTTTCGCTTTTTCAAAGAAGTAGAGT 191093

Query 480437 TCAGGAAAATAGCAGATGATCTGGGAAGGGAAATATTACTGGATGTGGATCCTAGATCTG 480496

||||||||||||||||||||||||||||||||||||||||||||||||||||||||||||

Sbjct 191094 TCAGGAAAATAGCAGATGATCTGGGAAGGGAAATATTACTGGATGTGGATCCTAGATCTG 191153

Query 480497 GCAATCATCTGGATTTTTCTTACTTAAGAATGAAGGTAGGGGTATGTGATAGGGACATAA 480556

||||||||||||||||||||||||||||||||||||||||||||||||||||||||||||

Sbjct 191154 GCAATCATCTGGATTTTTCTTACTTAAGAATGAAGGTAGGGGTATGTGATAGGGACATAA 191213

Query 480557 TACCTCCTTTTAGTAAGCTTCAATTTTAGGAGGAAACCGGAGATTTCATGTTCTATACAC 480616

||||||||||||||||||||||||||||||||||||||||||||||||||||||||||||

Sbjct 191214 TACCTCCTTTTAGTAAGCTTCAATTTTAGGAGGAAACCGGAGATTTCATGTTCTATACAC 191273

Query 480617 TGTTTTTTCAAATGGAGGATGAACAACCTAACCCATTTGAGTAGCAAGAGGATGGTCCTG 480676

||||||||||||||||||||||||||||||||||||||||||||||||||||||||||||

Sbjct 191274 TGTTTTTTCAAATGGAGGATGAACAACCTAACCCATTTGAGTAGCAAGAGGATGGTCCTG 191333

Query 480677 ATGGCCATGATCTGGGCCCTGGGGACAAGGTAATTCCAGACAAGACAAAGGTCCTAGAGG 480736

||||||||||||||||||||||||||||||||||||||||||||||||||||||||||||

Sbjct 191334 ATGGCCATGATCTGGGCCCTGGGGACAAGGTAATTCCAGACAAGACAAAGGTCCTAGAGG 191393

Query 480737 TGCTGGAAGTAGTCCTGGAGGAGACATTGTAGGGAAGAATGTAGGAGGCTTAGGAGACTC 480796

||||||||||||||||||||||||||||||||||||||||||||||||||||||||||||

Sbjct 191394 TGCTGGAAGTAGTCCTGGAGGAGACATTGTAGGGAAGAATGTAGGAGGCTTAGGAGACTC 191453

Query 480797 AAGTGGTTCAGGTGGTTCAGGTGGAAATACAAGAGGTTCAGGTGGTTCTGTTGCTGGTGC 480856

||||||||||||||||||||||||||||||||||||||||||||||||||||||||||||

Sbjct 191454 AAGTGGTTCAGGTGGTTCAGGTGGAAATACAAGAGGTTCAGGTGGTTCTGTTGCTGGTGC 191513

Query 480857 TTCCAGGAACACTATAGACAAAGGTAGTGGTAATGGGAAGGGAGTTAGAGGTGATATAGA 480916

||||||||||||||||||||||||||||||||||||||||||||||||||||||||||||

Sbjct 191514 TTCCAGGAACACTATAGACAAAGGTAGTGGTAATGGGAAGGGAGTTAGAGGTGATATAGA 191573

Query 480917 AGGTGGTAATTTAAAAGGTGCTGAAGGAAAATCCTCTGGCACTAAAGTTGACATGAAAGC 480976

||||||||||||||||||||||||||||||||||||||||||||||||||||||||||||

Sbjct 191574 AGGTGGTAATTTAAAAGGTGCTGAAGGAAAATCCTCTGGCACTAAAGTTGACATGAAAGC 191633

Query 480977 TGTAAATGAGAACCTGCATCCTGATCCCCTTCTCTCTCCAGCAAAAGAAACTGAAGTTGA 481036

|||||| |||||||||||||||||||||||||||||||||||||||||||||||||||||

Sbjct 191634 TGTAAAAGAGAACCTGCATCCTGATCCCCTTCTCTCTCCAGCAAAAGAAACTGAAGTTGA 191693

Query 481037 GCAAGAAACTATTGAAATTGAGGAGATTCCATACTCTTCACCAAAGTTTAAGTCTTCTGG 481096

||||||||||||||||||||||||||||||||||||||||||||||||||||||||||||

Sbjct 191694 GCAAGAAACTATTGAAATTGAGGAGATTCCATACTCTTCACCAAAGTTTAAGTCTTCTGG 191753

Query 481097 CAAGAGGAAGATAGAAGAGATGCCCAAAAACAAGAATCTGGCAATTATTACTTACTCTGG 481156

||||||||||||||||||||||||||||||||||||||||||||||||||||||||||||

Sbjct 191754 CAAGAGGAAGATAGAAGAGATGCCCAAAAACAAGAATCTGGCAATTATTACTTACTCTGG 191813

Query 481157 TCCTGACCAGACATTAGTTTTTCAAGAGAATTCGATGCAGCAAGGGGCTGGGGAGGCTGA 481216

||||||||||||||||||||||||||||||||||||||||||||||||||||||||||||

Sbjct 191814 TCCTGACCAGACATTAGTTTTTCAAGAGAATTCGATGCAGCAAGGGGCTGGGGAGGCTGA 191873

Query 481217 GAAGGAAGTTGAGGATTTGAATGGAGAAGTGGAGTCCACTCAGGAGATACTGGAGGTCCC 481276

||||||||||||||||||||||||||||||||||||||||||||||||||||||||||||

Sbjct 191874 GAAGGAAGTTGAGGATTTGAATGGAGAAGTGGAGTCCACTCAGGAGATACTGGAGGTCCC 191933

Query 481277 TCCTCTTAATCAAGCTCCTCCAGAATCAAGACAGAGTACAAGGCGCGCAGCGGGAAAGAG 481336

||||||||||||||||||||||||||||||||||||||||||||||||||||||||||||

Sbjct 191934 TCCTCTTAATCAAGCTCCTCCAGAATCAAGACAGAGTACAAGGCGCGCAGCGGGAAAGAG 191993

Query 481337 GCCCATTCAGTTCTCCTCAGGAGCACCAGTCCAGGGCCCACCTCAGAACAGAATTCCTCT 481396

||||||||||||||||||||||||||||||||||||||||||||||||||||||||||||

Sbjct 191994 GCCCATTCAGTTCTCCTCAGGAGCACCAGTCCAGGGCCCACCTCAGAACAGAATTCCTCT 192053

Query 481397 TCTTAGGGCAGGTATAGATTCTTTAATTAATGATTTTCCTTATCCAGAATATACAGATGA 481456

||||||||||||||||||||||||||||||||||||||||||||||||||||||||||||

Sbjct 192054 TCTTAGGGCAGGTATAGATTCTTTAATTAATGATTTTCCTTATCCAGAATATACAGATGA 192113

Query 481457 TGAATTAGTTAATGTGTTTACTCGGAATGGTTTCTCTCTAGGTACTGATGACAAAACTAG 481516

||||||||||||||||||||||||||||||||||||||||||||||||||||||||||||

Sbjct 192114 TGAATTAGTTAATGTGTTTACTCGGAATGGTTTCTCTCTAGGTACTGATGACAAAACTAG 192173

Query 481517 ATTAAAAGTAATTCAACACTTTAGGTCTTATACAAAACAGAAGTTAACTTATGTCCTTCA 481576

||||||||||||||||||||||||||||||||||||||||||||||||||||||||||||

Sbjct 192174 ATTAAAAGTAATTCAACACTTTAGGTCTTATACAAAACAGAAGTTAACTTATGTCCTTCA 192233

Query 481577 AGGTATCTTAGATAAATTAGATAGTTCCTCTAGTAATTCACTTGTAGATTTAAGCACTTC 481636

||||||||||||||||||||||||||||||||||||||||||||||||||||||||||||

Sbjct 192234 AGGTATCTTAGATAAATTAGATAGTTCCTCTAGTAATTCACTTGTAGATTTAAGCACTTC 192293

Query 481637 AGTGCTGCCTTTGAGAGCTCTTAAGCATAAATGAATATAATCAGTTGGAATGTTAGAGGA 481696

||||||||||||||||||||||||||||||||||||||||||||||||||||||||||||

Sbjct 192294 AGTGCTGCCTTTGAGAGCTCTTAAGCATAAATGAATATAATCAGTTGGAATGTTAGAGGA 192353

Query 481697 CTGGGTAAACCAGCCGAGAAGAAGTCTATTTGCCCGGAAGAAATGTTCAAGCATGAGCCC 481756

||||||||||||||||||||||||||||||||||||||||||||||||||||||||||||

Sbjct 192354 CTGGGTAAACCAGCCGAGAAGAAGTCTATTTGCCCGGAAGAAATGTTCAAGCATGAGCCC 192413

Query 481757 GCAGCCTTAACACGTGAACTCCTGAGAATCGTATTCCTATTGTGTAGGTCCAAGCCAGAA 481816

||||||||||||||||||||||||||||||||||||||||||||||||||||||||||||

Sbjct 192414 GCAGCCTTAACACGTGAACTCCTGAGAATCGTATTCCTATTGTGTAGGTCCAAGCCAGAA 192473

Query 481817 CGTTTCACCATAGATGATGTCGAAGTGTTCTCCATTCTCAAGCATTTGAAACTCGGCACT 481876

||||||||||||||||||||||||||||||||||||||||||||||||||||||||||||

Sbjct 192474 CGTTTCACCATAGATGATGTCGAAGTGTTCTCCATTCTCAAGCATTTGAAACTCGGCACT 192533

Query 481877 TACGTATTCGAGCCTATGCACCGATCCTTCCGAAACCCAACAAGCCTGGTAAATTTCGCC 481936

||||||||||||||||||||||||||||||||||||||||||||||||||||||||||||

Sbjct 192534 TACGTATTCGAGCCTATGCACCGATCCTTCCGAAACCCAACAAGCCTGGTAAATTTCGCC 192593

Query 481937 CTATCACGCAGCCCCACGAGGCGGATCGACTGGTACTCAACGCCATGGCTCTCGTACTTA 481996

||||||||||||||||||||||||||||||||||||||||||||||||||||||||||||

Sbjct 192594 CTATCACGCAGCCCCACGAGGCGGATCGACTGGTACTCAACGCCATGGCTCTCGTACTTA 192653

Query 481997 GGGATGCACTGGAAGCAGTCTTTCTTCAAGCGTCACATGGATTCCGGGTCGGGCGTGGAA 482056

||||||||||||||||||||||||||||||||||||||||||||||||||||||||||||

Sbjct 192654 GGGATGCACTGGAAGCAGTCTTTCTTCAAGCGTCACATGGATTCCGGGTCGGGCGTGGAA 192713

Query 482057 CGAAAAGTTTCTTGAAGGAGGTTCAGGGCTGGCCTCCAATGGATTACCTAGTTCAGTGCG 482116

||||||||||||||||||||||||||||||||||||||||||||||||||||||||||||

Sbjct 192714 CGAAAAGTTTCTTGAAGGAGGTTCAGGGCTGGCCTCCAATGGATTACCTAGTTCAGTGCG 192773

Query 482117 ATGTGGTTAATTGTTTCGATAGCATTAGACACGATCTGCTTCTCCCGCTTCTTGGGGAAT 482176

||||||||||||||||||||||||||||||||||||||||||||||||||||||||||||

Sbjct 192774 ATGTGGTTAATTGTTTCGATAGCATTAGACACGATCTGCTTCTCCCGCTTCTTGGGGAAT 192833

Query 482177 GTTTGTTCCGGGGGAATCTTTACTTCGTGAAGCTCATCCACGCCTTCCTGACCACCAACA 482236

||||||||||||||||||||||||||||||||||||||||||||||||||||||||||||

Sbjct 192834 GTTTGTTCCGGGGGAATCTTTACTTCGTGAAGCTCATCCACGCCTTCCTGACCACCAACA 192893

Query 482237 TCTATGACAAGAACGGCAGAAACTATGCTTGCCGGGAGGTGGGTATTTCTCAAGGAAGTC 482296

||||||||||||||||||||||||||||||||||||||||||||||||||||||||||||

Sbjct 192894 TCTATGACAAGAACGGCAGAAACTATGCTTGCCGGGAGGTGGGTATTTCTCAAGGAAGTC 192953

Query 482297 CGATCTCACCAGTGCTCATGAAAAGTTATGGAAAAGTTTTAATAGAAAGAAAAGATCCTC 482356

|||||||||||||||||||||||||||||||||||||||||||| |||||||||||||||

Sbjct 192954 CGATCTCACCAGTGCTCATGAAAAGTTATGGAAAAGTTTTAATATAAAGAAAAGATCCTC 193013

Query 482357 ACTTTATTATACTAAAAGTTACATTAAATAAAATAAGTTATTTACATGAAAAGTGTCTCA 482416

||||||||||||||||||||||||||||||||||||||||||||||||||||||||||||

Sbjct 193014 ACTTTATTATACTAAAAGTTACATTAAATAAAATAAGTTATTTACATGAAAAGTGTCTCA 193073

Query 482417 TCAGCCGTTATCCCCTTGATAAATTTCCTACGGGCAAGTTCACTCTTTCTGCTGCCGAGG 482476

||||||||||||||||||||||||||||||||||||||||||||||||||||||||||||

Sbjct 193074 TCAGCCGTTATCCCCTTGATAAATTTCCTACGGGCAAGTTCACTCTTTCTGCTGCCGAGG 193133

Query 482477 AAAGAAGTCGAATTCGGGAAGGCTCAGCTTTGGTGGTATATCCTGACTTATAAAAGTAGT 482536

||||||||||||||||||||||||||||||||||||||||||||||||||||||||||||

Sbjct 193134 AAAGAAGTCGAATTCGGGAAGGCTCAGCTTTGGTGGTATATCCTGACTTATAAAAGTAGT 193193

Query 482537 GttttttttCTATTTTCCAAAGTAAAAGTCCTAGATTGAGGAAATAATATATATGGTAGA 482596

| ||||||||||||||||||||||||||||||||||||||||||||||||||||||||||

Sbjct 193194 G-TTTTTTTCTATTTTCCAAAGTAAAAGTCCTAGATTGAGGAAATAATATATATGGTAGA 193252

Query 482597 ATCCCCGAATCATTAGGTTTTCTTTATCTTTACTCACTGCACTTGAAGAACAAAAGTCTC 482656

||||||||||||||||||||||||||||||||||||||||||||||||||||||||||||

Sbjct 193253 ATCCCCGAATCATTAGGTTTTCTTTATCTTTACTCACTGCACTTGAAGAACAAAAGTCTC 193312

Query 482657 GCGGGACCCTTCATACTTAGTAAATTGCAGCCtttttttttCGAGTTTATCTTGGGGAGA 482716

|||||||||||||||||||||||||||||||| |||||||||||||||||||||||||||

Sbjct 193313 GCGGGACCCTTCATACTTAGTAAATTGCAGCC-TTTTTTTTCGAGTTTATCTTGGGGAGA 193371

Query 482717 ACCAATTTACTGTAAACATACCAGAATGGGTTGCTCAGAATCGTTAAAACTTTATGATTT 482776

||||||||||||||||||||||||||||||||||||||||||||||||||||||||||||

Sbjct 193372 ACCAATTTACTGTAAACATACCAGAATGGGTTGCTCAGAATCGTTAAAACTTTATGATTT 193431

Query 482777 GGGGTAAATTCACATTCAGCTATCACGCCTAACTCTGCTTCAAGTGCTATATTGTTTTAT 482836

|||| |||||||||||||||||||||||||||||||||||||||||||||||||||||||

Sbjct 193432 GGGGGAAATTCACATTCAGCTATCACGCCTAACTCTGCTTCAAGTGCTATATTGTTTTAT 193491

Query 482837 TATAATAAAACACTACTGGTGTCTTAGTCGTAGCCGCTTGCTGCATAGATAGCTGTGAGG 482896

||||||||||||||||||||||||||||||||||||||||||||||||||||||||||||

Sbjct 193492 TATAATAAAACACTACTGGTGTCTTAGTCGTAGCCGCTTGCTGCATAGATAGCTGTGAGG 193551

Query 482897 AAGGGAGGTAAGTAAGTTCTTGCACTAGTCTGGGAGGGACATGGATCGAATCGGAATGCT 482956

||||||||||||||||||||||||||||||||||||||||||||||||||||||||||||

Sbjct 193552 AAGGGAGGTAAGTAAGTTCTTGCACTAGTCTGGGAGGGACATGGATCGAATCGGAATGCT 193611

Query 482957 AGCTTGGAATAAATCATTCTCCGCAACCTTCTTTCAGTCAGAGTGGCAAGCATGCTTTCG 483016

||||||||||||||||||||||||||||||||||||||||||||||||||||||||||||

Sbjct 193612 AGCTTGGAATAAATCATTCTCCGCAACCTTCTTTCAGTCAGAGTGGCAAGCATGCTTTCG 193671

Query 483017 AGTATCAGTAGGACGTGCATAAATGCAATTTCTTTTTAAAAAATATACATTATTTAATAA 483076

||||||||||||||||||||||||||||||||||||||||||||||||||||||||||||

Sbjct 193672 AGTATCAGTAGGACGTGCATAAATGCAATTTCTTTTTAAAAAATATACATTATTTAATAA 193731

Query 483077 TAAATGATATATCATTTAATAGTTCGACTTCCGTTACTTCGATAGAATAGAGTTATAAGA 483136

||||||||||| |||||||||||||||||||||||||||||||||||||||||||||||

Sbjct 193732 TAAATGATATAGAATTTAATAGTTCGACTTCCGTTACTTCGATAGAATAGAGTTATAAGA 193791

Query 483137 GCTGGAGGATAGGCACGAAGTGTTTTCTTGCTTGTTTTGGTTTTGACTTTCTTGGTTGCC 483196

||||||||||||||||||||||||||||||||||||||||||||||||||||||||||||

Sbjct 193792 GCTGGAGGATAGGCACGAAGTGTTTTCTTGCTTGTTTTGGTTTTGACTTTCTTGGTTGCC 193851

Query 483197 CAGCATGCATATAAGAGTATCCCCTTTCAGGGAGTTTTGCCCGTCCTTAAAAAGCGTCTA 483256

||||||||||||||||||||||||||||||||||||||||||||||||||||||||||||

Sbjct 193852 CAGCATGCATATAAGAGTATCCCCTTTCAGGGAGTTTTGCCCGTCCTTAAAAAGCGTCTA 193911

Query 483257 GAAAGAAAGGGCCGAAGGACGCGCAGCGGTTGAAGATCTATTCCTTTGAACGAGCTAAAC 483316

||||||||||||||||||||||||||||||||||||||||||||||||||||||||||||

Sbjct 193912 GAAAGAAAGGGCCGAAGGACGCGCAGCGGTTGAAGATCTATTCCTTTGAACGAGCTAAAC 193971

Query 483317 CACTCTTTTTAATTAAGTAGTACCACTGCTTTGCCCGAAGCTCTCATTCTACAGTTAAGG 483376

||||||||||||||||||||||||||||||||||||||||||||||||||||||||||||

Sbjct 193972 CACTCTTTTTAATTAAGTAGTACCACTGCTTTGCCCGAAGCTCTCATTCTACAGTTAAGG 194031

Query 483377 TTTCAGCGGGATCCGTTCTATCAGGATCTACAACTTTTATGCTCCCGGGAGCCCGATGCC 483436

||||||||||||||||||||||||||||||||||||||||||||||||||||||||||||

Sbjct 194032 TTTCAGCGGGATCCGTTCTATCAGGATCTACAACTTTTATGCTCCCGGGAGCCCGATGCC 194091

Query 483437 ATCAGTCAACTAACAGGTTAGCAAGCCTACCAACGAAGCACTCATCCCATTCAAAGCTAC 483496

||||||||||||||||||||||||||||||||||||||||||||||||||||||||||||

Sbjct 194092 ATCAGTCAACTAACAGGTTAGCAAGCCTACCAACGAAGCACTCATCCCATTCAAAGCTAC 194151

Query 483497 GTTTATCACGCCGAAGGCGGGTGGTGGTATACAAAGGAGGTCGTAAGTTAATCTCATTTT 483556

||||||||||||||||||||||||||||||||||||||||||||||||||||||||||||

Sbjct 194152 GTTTATCACGCCGAAGGCGGGTGGTGGTATACAAAGGAGGTCGTAAGTTAATCTCATTTT 194211

Query 483557 CCACTAGTGGGTGCGGGCTTCCTCTCCATAATAACTAACCACTCCATTAAGCCAATTAAT 483616

||||||||||||||||||||||||||||||||||||||||||||||||||||||||||||

Sbjct 194212 CCACTAGTGGGTGCGGGCTTCCTCTCCATAATAACTAACCACTCCATTAAGCCAATTAAT 194271

Query 483617 TTGGGTATTTCCTGCATATTCTACGGACGGATCATCTTCGGACGTATACAGACCCAATAT 483676

||||||||||||||||||||||||||||||||||||||||||||||||||||||||||||

Sbjct 194272 TTGGGTATTTCCTGCATATTCTACGGACGGATCATCTTCGGACGTATACAGACCCAATAT 194331

Query 483677 AGGTTCTTTCTTTATACCCTGAAGTGCCTGGGGAGTTCTTCTTATATTAACATCTAAAAG 483736

||||||||||||||||||||||||||||||||||||||| ||||||||| ||||||||||

Sbjct 194332 AGGTTCTTTCTTTATACCCTGAAGTGCCTGGGGAGTTCTGCTTATATTACCATCTAAAAG 194391

Query 483737 TAAGTCCCTCTTTCCAGCATGCGAGTTTACAAACAATATTGAGGATAGAGAAGAGTGCCC 483796

|||||||||||||||||||||||||||||||||||||||||||||||||||||||||||

Sbjct 194392 GAAGTCCCTCTTTCCAGCATGCGAGTTTACAAACAATATTGAGGATAGAGAAGAGTGCCC 194451

Query 483797 TTTTCATTCCTTTTTCAAGATAGAGGTCAATGCATCCAAGGCAGCAAAGGAAAGTCTTCT 483856

||||||||||||||||||||||||||||||||||||||||||||||||||||||||||||

Sbjct 194452 TTTTCATTCCTTTTTCAAGATAGAGGTCAATGCATCCAAGGCAGCAAAGGAAAGTCTTCT 194511

Query 483857 TTTTGCTCAGGCTTTACTCCTTCCTCTGCTTACAGTCGAGCGGCTTCGCACGCGCCTCAT 483916

||||||||||||||||||||||||||||||||||||||||||||||||||||||||||||

Sbjct 194512 TTTTGCTCAGGCTTTACTCCTTCCTCTGCTTACAGTCGAGCGGCTTCGCACGCGCCTCAT 194571

Query 483917 CTGCCAGGAAAACGAGAATTTATGGTTCTGGTCTCTTTTGAATTGCATTTGCCACCGAAA 483976

||||||||||||||||||||||||||||||||||||||||||||||||||||||||||||

Sbjct 194572 CTGCCAGGAAAACGAGAATTTATGGTTCTGGTCTCTTTTGAATTGCATTTGCCACCGAAA 194631

Query 483977 GTTCAtttttttGAAATGGTTTGTCCGAAAGAAAGttttttttAGCTTTGGGGAGTAAAT 484036

||||||||||||||||||||||||||||||||||||||||||||||||||||||||||||

Sbjct 194632 GTTCATTTTTTTGAAATGGTTTGTCCGAAAGAAAGTTTTTTTTAGCTTTGGGGAGTAAAT 194691

Query 484037 TAGTTACAATTAGGTGCGTTTGGTGATAGAGTTAATTCGTGGGTGATGGCCGGGTCCATA 484096

||||||||||||||||||||||||||||||||||||||||||||||||||||||||||||

Sbjct 194692 TAGTTACAATTAGGTGCGTTTGGTGATAGAGTTAATTCGTGGGTGATGGCCGGGTCCATA 194751

Query 484097 AGCAAATAATCAAAAACGTCCTTGCCGCTGCGCGCCTAGACTTCGGTTAGCCCTTAAGAT 484156

||||||||||||||||||||||||||||||||||||||||||||||||||||||||||||

Sbjct 194752 AGCAAATAATCAAAAACGTCCTTGCCGCTGCGCGCCTAGACTTCGGTTAGCCCTTAAGAT 194811

Query 484157 TGGAATAAATAAGT-----TTCTATGAGATTAAGCCTCTGACTTCGCCTCACAGTGATTT 484211

|||||||||||||| |||||||||||||||||||||||||||||||||||||||||

Sbjct 194812 TGGAATAAATAAGTTTCTATTCTATGAGATTAAGCCTCTGACTTCGCCTCACAGTGATTT 194871

Query 484212 GCTTTTTCTCCGGATCAGCTCAACCAGTATAGGTACTGTGCCTCCGTATCCTGAATGAAA 484271

||||||||||||||||||||||||||||||||||||||||||||||||||||||||||||

Sbjct 194872 GCTTTTTCTCCGGATCAGCTCAACCAGTATAGGTACTGTGCCTCCGTATCCTGAATGAAA 194931

Query 484272 GAATCATAAGCATCCCGAACTCCAACTACTTGAATATTTACCATAAATCTTTTCTTCCCA 484331

||||||||||||||||||||||||||||||||||||||||||||||||||||||||||||

Sbjct 194932 GAATCATAAGCATCCCGAACTCCAACTACTTGAATATTTACCATAAATCTTTTCTTCCCA 194991

Query 484332 GTGGGTGAATTCTCTTATTCCAACCGGAACTTTCTGTCTTTGACGTGGGTACCTATTACT 484391

||||||||||||||||||||||||||||||||||||||||||||||||||||||||||||

Sbjct 194992 GTGGGTGAATTCTCTTATTCCAACCGGAACTTTCTGTCTTTGACGTGGGTACCTATTACT 195051

Query 484392 AATAGGGAATGTGGGCGATCCCACACGAGTGAAAACTACATGGCATAAGCTGGAGCTGTT 484451

||||||||||||||||||||||||||||||||||||||||||||||||||||||||||||

Sbjct 195052 AATAGGGAATGTGGGCGATCCCACACGAGTGAAAACTACATGGCATAAGCTGGAGCTGTT 195111

Query 484452 CTTACACATCATGATATCTCGATTTGCAATCACTTCTAGCATTAGAAATCTTGTCACTTT 484511

||||||||||||||||||||||||||||||||||||||||||||||||||||||||||||

Sbjct 195112 CTTACACATCATGATATCTCGATTTGCAATCACTTCTAGCATTAGAAATCTTGTCACTTT 195171

Query 484512 AGTTTAATCACTCTAATTTAATAAATAATTACTTTGGAATACTTACATACATAATACATA 484571

|||||||||||||||||||||||||| |||||||||||||||||||||||||||||||||

Sbjct 195172 AGTTTAATCACTCTAATTTAATAAATCATTACTTTGGAATACTTACATACATAATACATA 195231

Query 484572 AACATTTTACAAGTTTAGCATACTTTACATGCAATTATTATTACTTTAATATTCACTTTT 484631

||||||||||||||||||||||||||||||||||||||||||||||||||||||||||||

Sbjct 195232 AACATTTTACAAGTTTAGCATACTTTACATGCAATTATTATTACTTTAATATTCACTTTT 195291

Query 484632 TAAGAATAAATCTTTGAACTTGTAGCACTAATTACTGTTGAATTAAGAATACTTACTGAA 484691

||||||||||||||||||||||||||||||||||||||||||||||||||||||||||||

Sbjct 195292 TAAGAATAAATCTTTGAACTTGTAGCACTAATTACTGTTGAATTAAGAATACTTACTGAA 195351

Query 484692 ATATATGGCTAGAGGGGGATGAATAGAACTGAAATTGAATTGCATTTTTTATTTGATAAA 484751

||||||||||||||||||||||||||||||||||||||||||||||||||||||||||||

Sbjct 195352 ATATATGGCTAGAGGGGGATGAATAGAACTGAAATTGAATTGCATTTTTTATTTGATAAA 195411

Query 484752 TAGAAGTGTGACTAAAATGAAAGTCCTATACCAAAATTCTCAACCGTCAAGACTTAATAG 484811

||||||||||||||||||||||||||||||||||||||||||||||||||||||||||||

Sbjct 195412 TAGAAGTGTGACTAAAATGAAAGTCCTATACCAAAATTCTCAACCGTCAAGACTTAATAG 195471

Query 484812 AATATTCAAACTCAATAATATTATATTAATATCTCAGGAAAGAGTTCCACCTAGTGCTTA 484871

||||||||||||||||||||||||||||||||||||||||||||||||||||||||||||

Sbjct 195472 AATATTCAAACTCAATAATATTATATTAATATCTCAGGAAAGAGTTCCACCTAGTGCTTA 195531

Query 484872 ATCTGTGTTGTGCCCCTAAGCGTATTGAGCCCCAGAAGAGATCCACGTTTAGCTTTCTCA 484931

||||||||||||||||||||||||||||||||||||||||||||||||||||||||||||

Sbjct 195532 ATCTGTGTTGTGCCCCTAAGCGTATTGAGCCCCAGAAGAGATCCACGTTTAGCTTTCTCA 195591

Query 484932 TTTTCATACCTGGGAGACAAAGAAATAAATTGCTGACTGACTATTCGAATGCTTACCTCT 484991

||||||||||||||||||||||||||||||||||||||||||||||||||||||||||||

Sbjct 195592 TTTTCATACCTGGGAGACAAAGAAATAAATTGCTGACTGACTATTCGAATGCTTACCTCT 195651

Query 484992 TTAACTCCTGCATTAACTCAACCTGCGGCCTCCATCTGATTATTAGAAAAAGCCTTATAT 485051

||||||||||||||||||||||||||||||||||||||||||||||||| ||||||||||

Sbjct 195652 TTAACTCCTGCATTAACTCAACCTGCGGCCTCCATCTGATTATTAGAAAGAGCCTTATAT 195711

Query 485052 TATACTTCTAATTCATTCTTACTGTGCATTAAGTGGTAAAGAGATTGCCCTAGGCATTAA 485111

||||||||||||||||||||||||||||||||||||||||||||||||||||||||||||

Sbjct 195712 TATACTTCTAATTCATTCTTACTGTGCATTAAGTGGTAAAGAGATTGCCCTAGGCATTAA 195771

Query 485112 AATCTTCCCAAAGAAAGGCATCATAGACTGGAATATCTTAGCTTAATAGTGAAAATCAAG 485171

||||||||||||||||||||||||||||||||||||||||||||||||||||||||||||

Sbjct 195772 AATCTTCCCAAAGAAAGGCATCATAGACTGGAATATCTTAGCTTAATAGTGAAAATCAAG 195831

Query 485172 GCATAATAAAGCTTTTTGAGTTACTATGGGCTGTAACCTTCTTTCCAACAAGAGATCCCT 485231

||||||||||||||||||||||||||||||||||||||||||||||||||||||||||||

Sbjct 195832 GCATAATAAAGCTTTTTGAGTTACTATGGGCTGTAACCTTCTTTCCAACAAGAGATCCCT 195891

Query 485232 TTGGCTTCATATTCTTAATATTAGGCGCGAAGCGGTTAATAATCACATAGAAAGGTTGAA 485291

||||||||||||||||||||||||||||||||||||||||||||||||||||||||||||

Sbjct 195892 TTGGCTTCATATTCTTAATATTAGGCGCGAAGCGGTTAATAATCACATAGAAAGGTTGAA 195951

Query 485292 CTATATAAACCTCTATGGCAGGGACTAGGAGGCTGCCTTCCTACTTACTACTTGCTAGCT 485351

||||||||||||||||||||||||||||||||||||||||||||||||||||||||||||

Sbjct 195952 CTATATAAACCTCTATGGCAGGGACTAGGAGGCTGCCTTCCTACTTACTACTTGCTAGCT 196011

Query 485352 TTCCTTCAACCAAGCCTTAAACGATGGAATGGAGGGCTCTCCATCTCCGTACTAGTCATG 485411

||||||||||||||||||||||||||||||||||||||||||||||||||||||||||||

Sbjct 196012 TTCCTTCAACCAAGCCTTAAACGATGGAATGGAGGGCTCTCCATCTCCGTACTAGTCATG 196071

Query 485412 ACTCGAGAGCGAGCGGAATACTTGTAGCGAACTGGTTTCAGTCAATTGCCACAATTGCTA 485471

||||||||||||||||||||||||||||||||||||||||||||||||||||||||||||

Sbjct 196072 ACTCGAGAGCGAGCGGAATACTTGTAGCGAACTGGTTTCAGTCAATTGCCACAATTGCTA 196131

Query 485472 GATCTTTCTTTTCTCGCTCTACTTTTGTACTATTAAAGTATAGAAGAAGAACGTTCTGGA 485531

||||||||||||||||||||||||||||||||||||||||||||||||||||||||||||

Sbjct 196132 GATCTTTCTTTTCTCGCTCTACTTTTGTACTATTAAAGTATAGAAGAAGAACGTTCTGGA 196191

Query 485532 CTTAGACGGCAACAATACTTAAACAATGTATATTATTCCTTCTTCTCCTTTGACTTCGAT 485591

||||||||||||||||||||||||||||||||||||||||||||||||||||||||||||

Sbjct 196192 CTTAGACGGCAACAATACTTAAACAATGTATATTATTCCTTCTTCTCCTTTGACTTCGAT 196251

Query 485592 TCTATCTTCCTGGATAATAGTAGAGAACTACTGGCCTTGGTCTCCACGCTAGCACTGACC 485651

||||||||||||||||||||||||||||||||||||||||||||||||||||||||||||

Sbjct 196252 TCTATCTTCCTGGATAATAGTAGAGAACTACTGGCCTTGGTCTCCACGCTAGCACTGACC 196311

Query 485652 TGGTCACCCAGCACTCCTTTCCAACCAAAGGAGCTGAAAGAGTGTAGGCGAGAGGAGCGA 485711

||||||||||||||||||||||||||||||||||||||||||||||||||||||||||||

Sbjct 196312 TGGTCACCCAGCACTCCTTTCCAACCAAAGGAGCTGAAAGAGTGTAGGCGAGAGGAGCGA 196371

Query 485712 AGTGAGCCAATAAAGTAAGTCAAGAAGCTAGGCCGAGAACCAGTAGCCGAAGCTATTGAG 485771

||||||||||||||||||||||||||||||||||||||||||||||||||||||||||||

Sbjct 196372 AGTGAGCCAATAAAGTAAGTCAAGAAGCTAGGCCGAGAACCAGTAGCCGAAGCTATTGAG 196431

Query 485772 AAAATTCTAGCTCGTGATAAGATAAGAAGTACCATTCTAAAGTAAGGAGCAAAGCTATCA 485831

||||||||||||||||||||||||||||||||||||||||||||||||||||||||||||

Sbjct 196432 AAAATTCTAGCTCGTGATAAGATAAGAAGTACCATTCTAAAGTAAGGAGCAAAGCTATCA 196491

Query 485832 ACCCGAGGTCGTATAAAGCATAAATGAAATCACCGGGCGCTGCGCCCTTACATTTAAGGA 485891

||||||||||||||||||||||||||||||||||||||||||||||||||||||||||||

Sbjct 196492 ACCCGAGGTCGTATAAAGCATAAATGAAATCACCGGGCGCTGCGCCCTTACATTTAAGGA 196551

Query 485892 AGCCCTTACTCTTATATCTTCTAGATAGAAGTAAGAAAGGCCATAAACCGACTGTTGATA 485951

||||||||||||||||||||||||||||||||||||||||||||||||||||||||||||

Sbjct 196552 AGCCCTTACTCTTATATCTTCTAGATAGAAGTAAGAAAGGCCATAAACCGACTGTTGATA 196611

Query 485952 TACCTCCAAAAGATCCCCGACATGAGCATCAGGAAACAAGCCTGAAAGGCATAAATGGAG 486011

||||||||||||||||||||||||||||||||||||||||||||||||||||||||||||

Sbjct 196612 TACCTCCAAAAGATCCCCGACATGAGCATCAGGAAACAAGCCTGAAAGGCATAAATGGAG 196671

Query 486012 GTCCTTCGTAATATGCCTTACTCTAGCGAGAGACTTGTAGCGAGCTGTGAGCTGTTTTCG 486071

||||||||||||||||||||||||||||||||||||||||||||||||||||||||||||

Sbjct 196672 GTCCTTCGTAATATGCCTTACTCTAGCGAGAGACTTGTAGCGAGCTGTGAGCTGTTTTCG 196731

Query 486072 AGTGGGAGTAGAAAACTTTAATGAACATTGAGTTTGACTAGGCACGGAGCCGATTTGAAT 486131

||||||||||||||||||||||||||||||||||||||||||||||||||||||||||||

Sbjct 196732 AGTGGGAGTAGAAAACTTTAATGAACATTGAGTTTGACTAGGCACGGAGCCGATTTGAAT 196791

Query 486132 TAGTACCACTTTCTTGCCTCAACTCGAGAGACCTTTCCCCGAGCGAACAATAGCAGTTCA 486191

||||||||||||||||||||||||||||||||||||||||||||||||||||||||||||

Sbjct 196792 TAGTACCACTTTCTTGCCTCAACTCGAGAGACCTTTCCCCGAGCGAACAATAGCAGTTCA 196851

Query 486192 CCTTTCCCGGCGAAGACGATACATATTTAATACGACTGATGAGAGGAAGTGGCTATATGT 486251

||||||||||||||||||||||||||||||||||||||||||||||||||||||||||||

Sbjct 196852 CCTTTCCCGGCGAAGACGATACATATTTAATACGACTGATGAGAGGAAGTGGCTATATGT 196911

Query 486252 TGACTCTGTCTCGATGGATCTTATCTTATATATATGATTCCGGATTTGTGTTAACTTTTT 486311

||||||||||||||||||||||||||||||||||||||||||||||||||||||||||||

Sbjct 196912 TGACTCTGTCTCGATGGATCTTATCTTATATATATGATTCCGGATTTGTGTTAACTTTTT 196971

Query 486312 TGTaaaaaaaaaaGTTCTTTTCTTTTGTTATTTAGCAAATTTTTCTTCATCTGAATTGGA 486371

||||||||||||||||||||||||||||||||||||||||||||||||||||||||||||

Sbjct 196972 TGTAAAAAAAAAAGTTCTTTTCTTTTGTTATTTAGCAAATTTTTCTTCATCTGAATTGGA 197031

Query 486372 GGATCCAAAGTATTATTGGGTGACGCGTTCTTTTCGTTAACGTTTGACCTATCTTTTATT 486431

||||||||||||||||||||||||||||||||||||||||||||||||||||||||||||

Sbjct 197032 GGATCCAAAGTATTATTGGGTGACGCGTTCTTTTCGTTAACGTTTGACCTATCTTTTATT 197091

Query 486432 GTCAGTTTGTGTGCATGAGCATTGGTTGCTCGCTGTGTTTCACTTCAATGACTGTATCAT 486491

||||||||||||||||||||||||||||||||||||||||||||||||||||||||||||

Sbjct 197092 GTCAGTTTGTGTGCATGAGCATTGGTTGCTCGCTGTGTTTCACTTCAATGACTGTATCAT 197151

Query 486492 TGAGTTCTTTCTACAATTCTATGGTGGATTAAACTCGATATGAAAAACTTGCAACAGAGG 486551

||||||||||||||||||||||||||||||||||||||||||||||||||||||||||||

Sbjct 197152 TGAGTTCTTTCTACAATTCTATGGTGGATTAAACTCGATATGAAAAACTTGCAACAGAGG 197211

Query 486552 TTGTACACTGGTAGCAGGATTTCATTGCACGTCGGGGAACTATGATATTCGTTTGTGGGA 486611

||||||||||||||||||||||||||||||||||||||||||||||||||||||||||||

Sbjct 197212 TTGTACACTGGTAGCAGGATTTCATTGCACGTCGGGGAACTATGATATTCGTTTGTGGGA 197271

Query 486612 TAGAGTTTGAACAAGGAAGAGATGGCTCATAAGATAGTCGGTACGAATGCGAGTCTGATC 486671

||||||||||||||||||||||||||||||||||||||||||||||||||||||||||||

Sbjct 197272 TAGAGTTTGAACAAGGAAGAGATGGCTCATAAGATAGTCGGTACGAATGCGAGTCTGATC 197331

Query 486672 TTCATCCTGACACTCTCCCCATTGACTAGCTTTGAGAACGAGCGAGACAAGAAAGCCTAT 486731

||||||||||||||||||||||||||||||||||||||||||||||||||||||||||||

Sbjct 197332 TTCATCCTGACACTCTCCCCATTGACTAGCTTTGAGAACGAGCGAGACAAGAAAGCCTAT 197391

Query 486732 TGATTGGGTAATCCAAGTAGGGTTGGACAGCAACTCTACATTTTCTTTCTTTTCTTTTAG 486791

||||||||||||||||||||||||||||||||||||||||||||||||||||||||||||

Sbjct 197392 TGATTGGGTAATCCAAGTAGGGTTGGACAGCAACTCTACATTTTCTTTCTTTTCTTTTAG 197451

Query 486792 GCCTGGATGaaaaaaaTAAACCCCCGCCCAACTTTTTAAGTTCTATACAAATGCTCAATC 486851

||||||||||||||||||||||||||||||||||||||||||||||||||||||||||||

Sbjct 197452 GCCTGGATGAAAAAAATAAACCCCCGCCCAACTTTTTAAGTTCTATACAAATGCTCAATC 197511

Query 486852 CCATCCTACGTAGTCGTACATTTGTATGTAGAGGCTATTAGAAATTTATTTCTAACATGC 486911

||||||||||||||||||||||||||||||||||||||||||||||||||| ||||||||

Sbjct 197512 CCATCCTACGTAGTCGTACATTTGTATGTAGAGGCTATTAGAAATTTATTTATAACATGC 197571

Query 486912 AGGCACGAAGTGTCACAATTTCCACAAAATGTTTCAAACCCATAAAATCAGTTCGAATTT 486971

||||||||||||||||||||||||||||||||||||||||||||||||||||||||||||

Sbjct 197572 AGGCACGAAGTGTCACAATTTCCACAAAATGTTTCAAACCCATAAAATCAGTTCGAATTT 197631

Query 486972 TACTGCTGCCTAGTTAGAGATATGTACTGAATGAGCCTAACCATTTTGGTACATAAATTA 487031

|||||||||||||||||||||||||||||||||||||||||||||||||||||||||| |

Sbjct 197632 TACTGCTGCCTAGTTAGAGATATGTACTGAATGAGCCTAACCATTTTGGTACATAAATGA 197691

Query 487032 ATCTTGCGCTAACACTAAGACTAGGCTAAAACTTCCTCGTACAGCCTTATGTACTGGTAA 487091

||||||||||||||||||||||||||||||||||||||||||||||||||||||||||||

Sbjct 197692 ATCTTGCGCTAACACTAAGACTAGGCTAAAACTTCCTCGTACAGCCTTATGTACTGGTAA 197751

Query 487092 GTCTGGTTGAGCCCAACCAGTTCTTCATGCAGAATATTAACAACTAGGATTTCATTTCAA 487151

||||||||||||||||||||||||||||||||||||||||||||||||||||||||||||

Sbjct 197752 GTCTGGTTGAGCCCAACCAGTTCTTCATGCAGAATATTAACAACTAGGATTTCATTTCAA 197811

Query 487152 GAGAAACTAACAAATTGGTGTTTCTAGTTTATGGCCAACATGCTGAGAATAGACGTACCC 487211

||||||||||||||||||||||||||||||||||||||||||||||||||||||||||||

Sbjct 197812 GAGAAACTAACAAATTGGTGTTTCTAGTTTATGGCCAACATGCTGAGAATAGACGTACCC 197871

Query 487212 CTACAACTACTACAACTCCAACCAAAAGTGGGGATGTGAGATAATAATTAGAACCTTGCC 487271

||||||||||||||||||||||||||||||||||||||||||||||||||||||||||||

Sbjct 197872 CTACAACTACTACAACTCCAACCAAAAGTGGGGATGTGAGATAATAATTAGAACCTTGCC 197931

Query 487272 AAGATATCGCAAGACTCCGACATTTCCACAAtttttttAAGTAATTGAAAAAGGTCCCTG 487331

||||||||||||||||||||||||||||||||||||||||||||||||||||||||||||

Sbjct 197932 AAGATATCGCAAGACTCCGACATTTCCACAATTTTTTTAAGTAATTGAAAAAGGTCCCTG 197991

Query 487332 AAGTTTTGAATCTATCTCAGAGCTCTGACTGTAACTAATGCATGCTGCATGCACACAGAA 487391

||||||||||||||||||||||||||||||||||||||||||||||||||||||||||||

Sbjct 197992 AAGTTTTGAATCTATCTCAGAGCTCTGACTGTAACTAATGCATGCTGCATGCACACAGAA 198051

Query 487392 TCAGCCTTGCACCAAGTACTCTTATTCTGAGAGATGTCACAAAACCCTATAATAATTTTT 487451

|||||||||||||||||||||||||||||||||||||||||||||||||||||||||||

Sbjct 198052 TCAGCCTTGCACCAAGTACTCTTATTCTGAGAGATGTCACAAAACCCTATAATAATTTTA 198111

Query 487452 ATATTTTTAGTTAATTGCTTAGAGTTTCTATAAATAGGAATTTCCATAACTGAACATGCT 487511

||||||||||||||||||||||||||||||||||||||||||||||||||||||||||||

Sbjct 198112 ATATTTTTAGTTAATTGCTTAGAGTTTCTATAAATAGGAATTTCCATAACTGAACATGCT 198171

Query 487512 ACCGACATTTTCTAGTTACATCCAGTAGCATTGAGAAGGACCTACACAAAGACGGAATAG 487571

||||||||||||||||||||||||||||||||||||||||||||||||||||||||||||

Sbjct 198172 ACCGACATTTTCTAGTTACATCCAGTAGCATTGAGAAGGACCTACACAAAGACGGAATAG 198231

Query 487572 ATTGATGAATAAAAAGTGAAAAAGGTCGTCTTGGATAAGCCGTGGAAATAAGTGGTGGAG 487631

||||||||||||||||||||||||||||||||||||||||||||||||||||||||||||

Sbjct 198232 ATTGATGAATAAAAAGTGAAAAAGGTCGTCTTGGATAAGCCGTGGAAATAAGTGGTGGAG 198291

Query 487632 TTGTCAAATATGATAGAGCTTTGCAATCTTTAGTTAAGTTAGTGGCTTCCTTTTTGTTAG 487691

||||||||||||||||||||||||||||||||||||||||||||||||||||||||||||

Sbjct 198292 TTGTCAAATATGATAGAGCTTTGCAATCTTTAGTTAAGTTAGTGGCTTCCTTTTTGTTAG 198351

Query 487692 CAGCATGCGCGTAAGAACTTAATATTTTTACTGGACCGGATTCAAAAGAAGGTATTGGTA 487751

||||||||||||||||||||||||||||||||||||||||||||||||||||||||||||

Sbjct 198352 CAGCATGCGCGTAAGAACTTAATATTTTTACTGGACCGGATTCAAAAGAAGGTATTGGTA 198411

Query 487752 GGAAATCCATTTTTTACACGCTTTTCGTTATTAGTTTAAAAATACTCTTTGCTCTATTTA 487811

||||||||||||||||||||||||||||||||||||| ||||||||||||||||||||||

Sbjct 198412 GGAAATCCATTTTTTACACGCTTTTCGTTATTAGTTTCAAAATACTCTTTGCTCTATTTA 198471

Query 487812 AGGTGAAGGCTGTTCCTAACCTCCGTATTTTAGATCTGTTTTCAAATGGCAACTGCATGT 487871

|||||||||||||||||||||||||||||| |||||||||||||||||||||||||||||

Sbjct 198472 AGGTGAAGGCTGTTCCTAACCTCCGTATTTGAGATCTGTTTTCAAATGGCAACTGCATGT 198531

Query 487872 TGAATTCTCGGTGAAGGAAATATTTCTGACAGCTCAGTGTTTACCGTAGCCCGCTAAAGC 487931

||||||||||||||||||||||||| ||||||||||||||||||||||||||||||||||

Sbjct 198532 TGAATTCTCGGTGAAGGAAATATTTATGACAGCTCAGTGTTTACCGTAGCCCGCTAAAGC 198591

Query 487932 CCAGTTTGTGAAGTTCAATTAATATTCGTCTCATTCTCTCTCTAAGTGACTGAGGCTAAT 487991

||||||||||||||||||||||||||||||||||||||||||||||||||||||||||||

Sbjct 198592 CCAGTTTGTGAAGTTCAATTAATATTCGTCTCATTCTCTCTCTAAGTGACTGAGGCTAAT 198651

Query 487992 CGTTCTAAGGGTTGTGTGTGATTGAGTTTTGTCTAAGTATTCTGGGGACTAGTTTTGCAC 488051

||||||||||||||||||||||||||||||||||||||||||||||||||||||||||||

Sbjct 198652 CGTTCTAAGGGTTGTGTGTGATTGAGTTTTGTCTAAGTATTCTGGGGACTAGTTTTGCAC 198711

Query 488052 ATTTTCTATTGTACCTTTGGCAATAGGAAGTCTGAACCGACACCCCCCTATCCCGCCTAG 488111

||||||||||||||||||||||||||||||||||||||||||||||||||||||||||||

Sbjct 198712 ATTTTCTATTGTACCTTTGGCAATAGGAAGTCTGAACCGACACCCCCCTATCCCGCCTAG 198771

Query 488112 TTGGAATGCTGTGCTTTAGATAGATTCTTGGGTTTTTTCTTATCTTAATAGTGGGGTTCT 488171

|||||||||||||||||||||||||||||||||||||||||||||| |||||||||||||

Sbjct 198772 TTGGAATGCTGTGCTTTAGATAGATTCTTGGGTTTTTTCTTATCTTCATAGTGGGGTTCT 198831

Query 488172 ATGATATAGAATAGTAAGGATAAGATATGACACAAATACATACCAAAAGGTAAGAACATG 488231

||||||||||||||||||||||||||||||||||||||||||||||||||||||||||||

Sbjct 198832 ATGATATAGAATAGTAAGGATAAGATATGACACAAATACATACCAAAAGGTAAGAACATG 198891

Query 488232 GAACTTGCCAAGATCGTACCGCAAGCAAACAAAGATGCCTCCAGAGAATATTATCTACGG 488291

||||||||||||||||||||||||||||||||||||||||||||||||| ||||||||||

Sbjct 198892 GAACTTGCCAAGATCGTACCGCAAGCAAACAAAGATGCCTCCAGAGAATCTTATCTACGG 198951

Query 488292 GGAAAACCTCCTTGTGACAACGCTATGCTATTCCTATTTCCGATTCGCATAGTCCTCTTT 488351

||||||||||||||||||||||||||||||||||||||||||||||||||||||||||||

Sbjct 198952 GGAAAACCTCCTTGTGACAACGCTATGCTATTCCTATTTCCGATTCGCATAGTCCTCTTT 199011

Query 488352 GTTTTAATTCCTACTTGCTATTCATTCTATGGGGATGGCTGATCTTGCTTGGTCTTCACG 488411

||||||||||||||||||||||||||||||||||||||||||||||||||||||||||||

Sbjct 199012 GTTTTAATTCCTACTTGCTATTCATTCTATGGGGATGGCTGATCTTGCTTGGTCTTCACG 199071

Query 488412 CCTTGGTCTTGGGCCTCCCGCTATTCTATAAAATTCATGATTCCTTTTAGCGaaaaaaaG 488471

|||||||||||||||||||||||||||||||||||||||||||||||||| |||||||||

Sbjct 199072 CCTTGGTCTTGGGCCTCCCGCTATTCTATAAAATTCATGATTCCTTTTAGTGAAAAAAAG 199131

Query 488472 AGGAGCTATGGCCCGACAACAACGGGTGCATATCGCGTAAAGCAAGTATGAAGCTCAACT 488531

||||||||||||||||||||||||||||||||||||||||||||||||||||||||||||

Sbjct 199132 AGGAGCTATGGCCCGACAACAACGGGTGCATATCGCGTAAAGCAAGTATGAAGCTCAACT 199191

Query 488532 TTCTTCATTTCTGGGATCAATATCGAGGTCTTTCCCCCCGCACTTATTGATACTATCTGA 488591

||||||||||||||||||||||||||||||||||||||||||||||||||||||||||||

Sbjct 199192 TTCTTCATTTCTGGGATCAATATCGAGGTCTTTCCCCCCGCACTTATTGATACTATCTGA 199251

Query 488592 CCTGCCTGTCAACAAACCTTTGACCTTTACCCGCGCTTTGACACAGACTTTGAAGCTGTA 488651

||||||||||||||||||||||||||||||||||||||||||||||||||||||||||||

Sbjct 199252 CCTGCCTGTCAACAAACCTTTGACCTTTACCCGCGCTTTGACACAGACTTTGAAGCTGTA 199311

Query 488652 CAGCCTCCGGCAATAGAGTTTCtttttttGTGTGGATAAATTGCAATCAAATCGATAGAG 488711

||||||||||||||||| ||||||||||||||||||||||||||||||||||||||||||

Sbjct 199312 CAGCCTCCGGCAATAGATTTTCTTTTTTTGTGTGGATAAATTGCAATCAAATCGATAGAG 199371

Query 488712 ATTAGGTAGTGGCAGGTTTTTCAAGCCTCATGTGTGCGTAACCAGACCTTCGGTCTTAAA 488771

||||||||||||||||||||||||||||||||||||||||||||||||||||||||||||

Sbjct 199372 ATTAGGTAGTGGCAGGTTTTTCAAGCCTCATGTGTGCGTAACCAGACCTTCGGTCTTAAA 199431

Query 488772 AAGGGAGCTTCCTCTCATGAAAATAGAGATGAACAAGGGCTGACCGATCTTTAGAAGTTT 488831

||||||||||||||||||||||||||||||||||||||||||||||||||||||||||||

Sbjct 199432 AAGGGAGCTTCCTCTCATGAAAATAGAGATGAACAAGGGCTGACCGATCTTTAGAAGTTT 199491

Query 488832 GAACCCGGCGTAAGTCCTCTTTTTCTTTAATGTAACCGGGGAGCATGGGAATGTTATGAG 488891

||||||||||||||||||||||||||||||||||||||||||||||||||||||||||||

Sbjct 199492 GAACCCGGCGTAAGTCCTCTTTTTCTTTAATGTAACCGGGGAGCATGGGAATGTTATGAG 199551

Query 488892 CATAGGAATGTGTACTGCATGGTCATGTGTTCCCCACTGCACTTCTCATGTGATCGTAGC 488951

||||||||||||||||||||||||||||||||||||||||||||||||||||||||||||

Sbjct 199552 CATAGGAATGTGTACTGCATGGTCATGTGTTCCCCACTGCACTTCTCATGTGATCGTAGC 199611

Query 488952 TTACTCAGACTTACATAAGGGGGGACCCACTAATACTCTTTTAATCGCATATGTTAAGTC 489011

||||||||||||||||||||||||||||||||||||||||||||||||||||||||||||

Sbjct 199612 TTACTCAGACTTACATAAGGGGGGACCCACTAATACTCTTTTAATCGCATATGTTAAGTC 199671

Query 489012 GGCGATAGCTCTACTCTTCAGTCTGATCCAGCATCATCAAGTGGAGCAGAAGGAGGGGTC 489071

||||||||||||||||||||||||||||||||||||||||||||||||||||||||||||

Sbjct 199672 GGCGATAGCTCTACTCTTCAGTCTGATCCAGCATCATCAAGTGGAGCAGAAGGAGGGGTC 199731

Query 489072 CGCATGCCCTTTTCTCACGAGACCCTTTATGTAATTTTGAAGGTCATAGCTTTCGTCCGC 489131

||||||||||||||||||||||||||||||||||||||||||||||||||||||||||||

Sbjct 199732 CGCATGCCCTTTTCTCACGAGACCCTTTATGTAATTTTGAAGGTCATAGCTTTCGTCCGC 199791

Query 489132 GGCATGGCCTCTTTTCCGGTGGAAGGTGCAACTTGGTCTTTCTTCCTCATTGATCTCAGG 489191

||||||||||||||||||||||||||||||||||||||||||||||||||||||||||||

Sbjct 199792 GGCATGGCCTCTTTTCCGGTGGAAGGTGCAACTTGGTCTTTCTTCCTCATTGATCTCAGG 199851

Query 489192 TAGACTTTCGATTTCTTCCTCGCCCAACTTACTTACTCTATAGGGCCTCTAAGACGGAGT 489251

||||||||||||||||||||||||||||||||||||||||||||||||||||||||||||

Sbjct 199852 TAGACTTTCGATTTCTTCCTCGCCCAACTTACTTACTCTATAGGGCCTCTAAGACGGAGT 199911

Query 489252 AGTAAAATAGTAAATTCCATCAAAAATACTGGCTTATTTGAGTTTCTAGCTTTCCACTTT 489311

|||||||||| |||||||||||||||||||||||||||||||||||||||||||||||||

Sbjct 199912 AGTAAAATAGGAAATTCCATCAAAAATACTGGCTTATTTGAGTTTCTAGCTTTCCACTTT 199971

Query 489312 CCTACTTTTGCCGAATTTCCTTCCTGGGGGAAGTAAACTTCTCTCCACTTTTCTTGAGCA 489371

||||||||||||||||||||||||||||||||||||||||||||||||||||||||||||

Sbjct 199972 CCTACTTTTGCCGAATTTCCTTCCTGGGGGAAGTAAACTTCTCTCCACTTTTCTTGAGCA 200031

Query 489372 ACTTATTACTTGGCTTTTTCACTTTCTACGCTTAGCCGATGAGAGGATAAAGAGGAAATC 489431

||||||||||||||||||||||||||||||||||||||||||||||||||||||||||||

Sbjct 200032 ACTTATTACTTGGCTTTTTCACTTTCTACGCTTAGCCGATGAGAGGATAAAGAGGAAATC 200091

Query 489432 CCACCAGGAGCTCTCACCCGAAGCAATTTTTCGTGCGTGTGTAGCCCCTTCCTTAAGTTG 489491

||||||||||||||||||||||||||||||||||||||||||||||||||||||||||||

Sbjct 200092 CCACCAGGAGCTCTCACCCGAAGCAATTTTTCGTGCGTGTGTAGCCCCTTCCTTAAGTTG 200151

Query 489492 GGAAGACCAGCCAGCCAACTAAGCCGCCCAGAGTAGAGGACGCAGCCGTGAATAGGCCAG 489551

||||||||||||||||||||||||||||||||||||||||||||||||||||||||||||

Sbjct 200152 GGAAGACCAGCCAGCCAACTAAGCCGCCCAGAGTAGAGGACGCAGCCGTGAATAGGCCAG 200211

Query 489552 TAAGATCTCTTTATCCTAGCTTTATCTCTTTTGTTGGCTCTTCCACAGCTGCAGTAGGCT 489611

|||||||||||||||| |||||||||||||||||||||||||||||||||||||||||||

Sbjct 200212 TAAGATCTCTTTATCCAAGCTTTATCTCTTTTGTTGGCTCTTCCACAGCTGCAGTAGGCT 200271

Query 489612 CGTCGAATATCCGAGCTCCACTAGCATACAGTTCCACCGAATAATCATCATCCTCGTTGT 489671

||||||||||||||||||||||||||||||||||||||||||||||||||||||||||||

Sbjct 200272 CGTCGAATATCCGAGCTCCACTAGCATACAGTTCCACCGAATAATCATCATCCTCGTTGT 200331

Query 489672 CGTAGACTCCACATCGTCTATCCCCATATCCACTGGATTCTCGTTCAAAAGGTTCTGTTG 489731

||||||||||||||||||||||||||||||||||||||||||||||||||||||||||||

Sbjct 200332 CGTAGACTCCACATCGTCTATCCCCATATCCACTGGATTCTCGTTCAAAAGGTTCTGTTG 200391

Query 489732 GCACTGGTTGTCGGGCTAGAATTTTGCTCAGCGTCTTCTTTGGGAGTGTTGGTCAATTGA 489791

||||||||||||||||||||||||||||||||||||||||||||||||||||||||||||

Sbjct 200392 GCACTGGTTGTCGGGCTAGAATTTTGCTCAGCGTCTTCTTTGGGAGTGTTGGTCAATTGA 200451

Query 489792 GGAGGTCTTATTGTAACATAACCATGAGCTGACACCGGATAGTGTGCGCCTGATGCATTG 489851

||||||||||||||||||||||||||||||||||||||||||||||||||||||||||||

Sbjct 200452 GGAGGTCTTATTGTAACATAACCATGAGCTGACACCGGATAGTGTGCGCCTGATGCATTG 200511

Query 489852 TCATCATGATCTACCTCTATTTGTaaaaaaaCAACTGGGGATTTATGAACTGCCTAGTTT 489911

||||||||||||||||||||||||||| ||||||||||||||||||||||||||||||||

Sbjct 200512 TCATCATGATCTACCTCTATTTGTAAAGAAACAACTGGGGATTTATGAACTGCCTAGTTT 200571

Query 489912 GTTTGTTCTAAAAAACATATAATATATACTTTGATAGGCATGATGCTCCTTGTAAGCACT 489971

||||||||||||||||||||||||||||||||||||||||||||||||||||||||||||

Sbjct 200572 GTTTGTTCTAAAAAACATATAATATATACTTTGATAGGCATGATGCTCCTTGTAAGCACT 200631

Query 489972 GATGGATTTTTGCAGCAGGTCTTGCACAACACAAATCCATATCGACAATTTCTTTTCACA 490031

||||||||||||||||||||||||||||||||||||||||||||||||||||||||||||

Sbjct 200632 GATGGATTTTTGCAGCAGGTCTTGCACAACACAAATCCATATCGACAATTTCTTTTCACA 200691

Query 490032 CAAGCATAACTAGAACGATTCTTTCTAGAAGATAATAAAGACCTTGAGCGCACCTCGATA 490091

||||||||||||||||||||||||||||||||||||||||||||||||||||||||||||

Sbjct 200692 CAAGCATAACTAGAACGATTCTTTCTAGAAGATAATAAAGACCTTGAGCGCACCTCGATA 200751

Query 490092 ATGGATTCTTTTTCGTAGGTAGATAGTATGTCCTCCCTCGTCGCTCCATGAGAAGCAAAG 490151

||||||||||||||||||||||||||||||||||||||||||||||||||||||||||||

Sbjct 200752 ATGGATTCTTTTTCGTAGGTAGATAGTATGTCCTCCCTCGTCGCTCCATGAGAAGCAAAG 200811

Query 490152 AAAGAAGCAGCGAGCCGTCGGAGCTGACTGAATTTTATCCAAACGCTATATGCTTAACAC 490211

||||||||||||||||| ||||||||||||||||||||||||||||||||||||||||||

Sbjct 200812 AAAGAAGCAGCGAGCCGCCGGAGCTGACTGAATTTTATCCAAACGCTATATGCTTAACAC 200871

Query 490212 AAGTAAGTCGAACCTTGTGTTGGATTTTCGTTTTGACTCTAGAAAAATAAATGGAATTGT 490271

||||||||||||||||||||||||||||||||||||||||||||||||||||||||||||

Sbjct 200872 AAGTAAGTCGAACCTTGTGTTGGATTTTCGTTTTGACTCTAGAAAAATAAATGGAATTGT 200931

Query 490272 GGTTTTTGAATTCCTAAAAATGAATGAGAATTTTCACAATTGTTGCTTGCCTGGCAGGAA 490331

||||||||||||||||||||||||||||||||||||||||||||||||||||||||||||

Sbjct 200932 GGTTTTTGAATTCCTAAAAATGAATGAGAATTTTCACAATTGTTGCTTGCCTGGCAGGAA 200991

Query 490332 GAATGTGCCTGACTGAGGGACGAGAACGACTTGACGGGGGACCGAAAAGAAACATGAAAA 490391

||||||||||||||||||||||||||||||||||||||||||||||||||||||||||||

Sbjct 200992 GAATGTGCCTGACTGAGGGACGAGAACGACTTGACGGGGGACCGAAAAGAAACATGAAAA 201051

Query 490392 AATACAGGCGCGTAGCGGCGAAGCCCGCCCGGAATTTCGTCTTCCCAAAAAACCAGCAAG 490451

||||||||||||||||||||||||||||||||||||||||||||||||||||||||||||

Sbjct 201052 AATACAGGCGCGTAGCGGCGAAGCCCGCCCGGAATTTCGTCTTCCCAAAAAACCAGCAAG 201111

Query 490452 CAGTACTTCAAGAAAGATGTAGAATCGTTAGAGCAACTTTGAACTGAACTCTTTTCCAGA 490511

||||||||||||||||||||||||||||||||||||||||||||||||||||||||||||

Sbjct 201112 CAGTACTTCAAGAAAGATGTAGAATCGTTAGAGCAACTTTGAACTGAACTCTTTTCCAGA 201171

Query 490512 GAGGAACTAGGTATTTCTTTTTAGTATGGACTTTGATCTTATTATTCTATTATATTTCTT 490571

||||||||||||||||||||||||||||||||||||||||||||||||||| ||||||||

Sbjct 201172 GAGGAACTAGGTATTTCTTTTTAGTATGGACTTTGATCTTATTATTCTATTCTATTTCTT 201231

Query 490572 TCGTAGAGAGAGGGTAGCGAGTCTTTTTCGCCCTCTCCACTCCTCCTCCCATGCTTTTCT 490631

||||||||||||||||||||||||||||||||||||||||||||||||||||||||||||

Sbjct 201232 TCGTAGAGAGAGGGTAGCGAGTCTTTTTCGCCCTCTCCACTCCTCCTCCCATGCTTTTCT 201291

Query 490632 TAGTTGGACCAACCCGACGCGGCGATTTCCGACAAGTCTTTCTTCATAGAGCAAGAAGCA 490691

|||||||||||||||||||||||||||||||||||||||||||||||||||||||||||

Sbjct 201292 TAGTTGGACCAACCCGACGCGGCGATTTCCGACAAGTCTTTCTTCATAGAGCAAGAAGCG 201351

Query 490692 GaaaaaaaGCTTTAtttttttAGATTGATGGATAACCAATTCATTTTCCAATATAGTTGG 490751

|||||||||||||||||||| |||||||||||||||||||||||||||||||||||||||

Sbjct 201352 GAAAAAAAGCTTTATTTTTTGAGATTGATGGATAACCAATTCATTTTCCAATATAGTTGG 201411

Query 490752 GATATTTTACCCAAGAAATGGGTACATAAAATGAAAAGATCGGAACATGGAGATATAATT 490811

||||||||||||||||||||||||||||||||||||||||||||||||||||||||||||

Sbjct 201412 GATATTTTACCCAAGAAATGGGTACATAAAATGAAAAGATCGGAACATGGAGATATAATT 201471

Query 490812 TATACCACTACTGACTACCTATTCCCGTTATTGTGCTTTCTCAAATGGCATACCTATACA 490871

||||||||||||||||||||||||||||||||||||||||||||||||||||||||||||

Sbjct 201472 TATACCACTACTGACTACCTATTCCCGTTATTGTGCTTTCTCAAATGGCATACCTATACA 201531

Query 490872 AGGGTTCAAGTTTTGATCGATATTTGCGGAGTAGATTATCCCTCTAGAAAACGCAGATTT 490931

||||||||||||||||||||||||||||||||||||||||||||||||||||||||||||

Sbjct 201532 AGGGTTCAAGTTTTGATCGATATTTGCGGAGTAGATTATCCCTCTAGAAAACGCAGATTT 201591

Query 490932 GAAGTTGTCTATAATTTACTGAGTACTCGGTATAACTCACGCATTCGTGTACAAACAAGT 490991

||||||||||||||||||||||||||||||||||||||||||||||||||||||||||||

Sbjct 201592 GAAGTTGTCTATAATTTACTGAGTACTCGGTATAACTCACGCATTCGTGTACAAACAAGT 201651

Query 490992 GCAGACGAAGTAACACGAATATCTTCGGTAGTCAGTCTATTTCCATCAGCTGGCTGGTGG 491051

||||||||||||||||||||||||||||||||||||||||||||||||||||||||||||

Sbjct 201652 GCAGACGAAGTAACACGAATATCTTCGGTAGTCAGTCTATTTCCATCAGCTGGCTGGTGG 201711

Query 491052 GAGCGAGAAATCTGGGATATGTTTGGTCTTTCTTTCATAAATCATCCGGATTTACGCCGT 491111

||||||||||||||||||||||||||||||||||||||||||||||||||||||||||||

Sbjct 201712 GAGCGAGAAATCTGGGATATGTTTGGTCTTTCTTTCATAAATCATCCGGATTTACGCCGT 201771

Query 491112 ATATTAACAGATTATGGTTTCGAGGGTCATCCATTACGAAAAGACTTTCCTCTTAGTGGA 491171

||||||||||||||||||||||||||||||||||||||||||||||||||||||||||||

Sbjct 201772 ATATTAACAGATTATGGTTTCGAGGGTCATCCATTACGAAAAGACTTTCCTCTTAGTGGA 201831

Query 491172 TATGTGGAAGTACGCTATGATGATCCAGAAAAACGTGTGGTTTCTGAACCCATTGAGATG 491231

||||||||||||||||||||||||||||||||||||||||||||||||||||||||||||

Sbjct 201832 TATGTGGAAGTACGCTATGATGATCCAGAAAAACGTGTGGTTTCTGAACCCATTGAGATG 201891

Query 491232 ACCCAAGAATTTCGCTATTTCGATTTTGCTAGTCCTTGGGAACAGCGTAGCGACGGATAA 491291

||||||||||||||||||||||||||||||||||||||||||||||||||||||||||||

Sbjct 201892 ACCCAAGAATTTCGCTATTTCGATTTTGCTAGTCCTTGGGAACAGCGTAGCGACGGATAA 201951

Query 491292 AAAACCACATAatatatatatataAGAAGTCTAGTCCTGGGGACAAAGAAATAGGAAATG 491351

||||||||||||||||||||||||||||||||||||||||||||||||||||||||||||

Sbjct 201952 AAAACCACATAATATATATATATAAGAAGTCTAGTCCTGGGGACAAAGAAATAGGAAATG 202011

Query 491352 CTATTTGCTTTTTAAGAATAAAACTCttttttttttGAAAGAGACGACATAATTTCATTT 491411

||||||||||||||||||||||||||||||||||||||||||||||||||||||||||||

Sbjct 202012 CTATTTGCTTTTTAAGAATAAAACTCTTTTTTTTTTGAAAGAGACGACATAATTTCATTT 202071

Query 491412 AATAAGGAAGAAGTGTTATCGAACGCTACGCCCTTCCATTCTTTGACTGACGTATGGAAG 491471

||||||||||||||||||||||||||||||||||||||||||||||||||||||||||||

Sbjct 202072 AATAAGGAAGAAGTGTTATCGAACGCTACGCCCTTCCATTCTTTGACTGACGTATGGAAG 202131

Query 491472 AAGTCAAGAAAAATTCTTTCCGGGTCTTTAACTTTTCATTGATAGCAGAAGAGTACGCGC 491531

|||||||| ||||||||||||||||||||||||||||||||||||||||||||||||

Sbjct 202132 AAGTCAAG----ATTCTTTCCGGGTCTTTAACTTTTCATTGATAGCAGAAGAGTACGCGC 202187

Query 491532 GGCTAGCTCCGCCTTCAAACGCTTGCTTGCCCCTAGACCTACGTAAACTCTTGCCTACGC 491591

||||||||||||||||||||||||||||||||||||||||||||||||||||||||||||

Sbjct 202188 GGCTAGCTCCGCCTTCAAACGCTTGCTTGCCCCTAGACCTACGTAAACTCTTGCCTACGC 202247

Query 491592 TTGCAAAAGCTCAGTTTAAGTAGAAGGGCGCTAGCAAGAGGGTCACTCCTTTGATGAAGA 491651

||||||||||||||||||||||||||||||||||||||||||||||||||||||||||||

Sbjct 202248 TTGCAAAAGCTCAGTTTAAGTAGAAGGGCGCTAGCAAGAGGGTCACTCCTTTGATGAAGA 202307

Query 491652 TGACTCGCCTGGAGACTTTCGCGCTTCACTCTATGCTCGCTGGCTCCTTCATTCACTTGC 491711

||||||||||||||||||||||||||||||||||||||||||||||||||||||||||||

Sbjct 202308 TGACTCGCCTGGAGACTTTCGCGCTTCACTCTATGCTCGCTGGCTCCTTCATTCACTTGC 202367

Query 491712 TCCTTCACTCGCTGCTTCGCCAGAAGCGAGGCCTCCTCAACTTAGCTAGAAGCAACGAAG 491771

||||||||||||||||||||||||||||||||||||||||||||||||||||||||||||

Sbjct 202368 TCCTTCACTCGCTGCTTCGCCAGAAGCGAGGCCTCCTCAACTTAGCTAGAAGCAACGAAG 202427

Query 491772 GAGGCTGGGGAAGGGAAAGCCGACGATGGGGGGCTTTCTTTCGTATAAGCTTTGCCTCTT 491831

||||||||||||||||||||||||||||||||||||||||||||||||||||||||||||

Sbjct 202428 GAGGCTGGGGAAGGGAAAGCCGACGATGGGGGGCTTTCTTTCGTATAAGCTTTGCCTCTT 202487

Query 491832 GCTTGACATAAATTTTTATGAACTTACTCAATGACCTTATTTATTCTCCCACTAGCTAAT 491891

||||||||||||||||||||||||||||||||||||||||||||||||||||||||||||

Sbjct 202488 GCTTGACATAAATTTTTATGAACTTACTCAATGACCTTATTTATTCTCCCACTAGCTAAT 202547

Query 491892 CTAATAAATAGATAGGGAGTTCCATCTTCTTTTCTTCACTTCAGAACGAAGGGCACCACC 491951

||||||||||||||||||||||||||||||||||||||||||||||||||||||||||||

Sbjct 202548 CTAATAAATAGATAGGGAGTTCCATCTTCTTTTCTTCACTTCAGAACGAAGGGCACCACC 202607

Query 491952 CTTATTATTAAGGAAATCTCTCACTCAGGGGGAGCTTCCGCCCGGAAAGGAAGGAGGGGA 492011

||||||||||||||| ||||||||||||||||||||||||||||||||||||||||||||

Sbjct 202608 CTTATTATTAAGGAACTCTCTCACTCAGGGGGAGCTTCCGCCCGGAAAGGAAGGAGGGGA 202667

Query 492012 GAGTGGCCGAGTGGTCAAAAGCGGCAGACTGTAAATCTGTTGAAAGTTTTCTACGTAGGT 492071

||||||||||||||||||||||||||||||||||||||||||||||||||||||||||||

Sbjct 202668 GAGTGGCCGAGTGGTCAAAAGCGGCAGACTGTAAATCTGTTGAAAGTTTTCTACGTAGGT 202727

Query 492072 TCGAATCCTGCCTCTCCCACTTGTTTGTTGTAGACTTCATAGAATAGAAAGAAAGTAGGC 492131

||||||||||||||||||||||||||||||||||||||||||||||||||||||||||||

Sbjct 202728 TCGAATCCTGCCTCTCCCACTTGTTTGTTGTAGACTTCATAGAATAGAAAGAAAGTAGGC 202787

Query 492132 GGAAGCCGCCGGAGGGAGGGCCAACCGAGCGAAGCTCTTTC-ttttttttCCGTGCTCTT 492190

||||||||||||||||||||||||||||||||||||||||| ||||||||||||||||||

Sbjct 202788 GGAAGCCGCCGGAGGGAGGGCCAACCGAGCGAAGCTCTTTCTTTTTTTTTCCGTGCTCTT 202847

Query 492191 TCAAATGTCTCGGTTTGAGAGAGGTTGTCGCAAAAATAAGATCTCTAGAGATTTCTTCCC 492250

||||||||||||||||||| ||||||||||||||||||||||||||||||||||||||||

Sbjct 202848 TCAAATGTCTCGGTTTGAGGGAGGTTGTCGCAAAAATAAGATCTCTAGAGATTTCTTCCC 202907

Query 492251 CTTCTATATCCAATCCCAACGATACGAGAATATATAGATAGGAGCGAGTTGCTTCCGGCG 492310

||||||||||||||||||||||||||||||||||||||||||||||||||||| ||||||

Sbjct 202908 CTTCTATATCCAATCCCAACGATACGAGAATATATAGATAGGAGCGAGTTGCTCCCGGCG 202967

Query 492311 TCGGCTTGTAGTTTCTGCAGTCGGCACACACGCGCGCCCGCCATCATGCCTCCTTGGTTC 492370

||||||||||||||||||||||||| ||||||||||||||||||||||||||||||||||

Sbjct 202968 TCGGCTTGTAGTTTCTGCAGTCGGCGCACACGCGCGCCCGCCATCATGCCTCCTTGGTTC 203027

Query 492371 GTTCGGGACGAAGCCAAGCGAGACTTACGATAGACGAAGGAAGAGCGGCCGTCGCCTTTG 492430

||||||||||||||||||||||||||||||||||||||||||||||||||| ||||||||

Sbjct 203028 GTTCGGGACGAAGCCAAGCGAGACTTACGATAGACGAAGGAAGAGCGGCCGCCGCCTTTG 203087

Query 492431 AGTTTTTAATAAAACTCCAGCTTCGAAGCTGGAGTTTAGTTTAGTCCTTCAGTTTGGATT 492490

||||||||||||||||||||||||||||||||||||||||||||||||||||||||||||

Sbjct 203088 AGTTTTTAATAAAACTCCAGCTTCGAAGCTGGAGTTTAGTTTAGTCCTTCAGTTTGGATT 203147

Query 492491 TAATTAAGCAAGAAGAAGAACCCTCGCGCCTTACTTGCGCGCTCAATAACACGCAAGGGC 492550

||||||||||||||||||||||| ||||||||||||||||||||||||||||||||||||

Sbjct 203148 TAATTAAGCAAGAAGAAGAACCCCCGCGCCTTACTTGCGCGCTCAATAACACGCAAGGGC 203207

Query 492551 GaaaaaaaGAGGGAAGGAAAGTTTGATTGAGGATTGGAGAGGAGAGGTGGAATAAAAAGC 492610

||||||||||||||||||||||||||||||||||||||||||||||||||||||||||||

Sbjct 203208 GAAAAAAAGAGGGAAGGAAAGTTTGATTGAGGATTGGAGAGGAGAGGTGGAATAAAAAGC 203267

Query 492611 TCGGGATGGATGATCGAGTCTTTGTGCGAGCCGTATGCGGTGAGAGTCGCACGTACGGTA 492670

||||||||||||||||||||||||||||||||||||||||||||||||||||||||||||

Sbjct 203268 TCGGGATGGATGATCGAGTCTTTGTGCGAGCCGTATGCGGTGAGAGTCGCACGTACGGTA 203327

Query 492671 AGGAGGGGGGTTCGCGTCTGTCTATACGTGTAGTGTGGTGGTAGGGCCTACCCACCCTAT 492730

||||||||||||||||||||||||||||||||||||||||||||||||||||||||||||

Sbjct 203328 AGGAGGGGGGTTCGCGTCTGTCTATACGTGTAGTGTGGTGGTAGGGCCTACCCACCCTAT 203387

Query 492731 TTGTTCCATGATCTATGGGTCTACTGGAGCTACCCACTTCGATCAATTAGCCAAGATTTT 492790

||||||||||||||||||||||||||||||||||||||||||||||||||||||||||||

Sbjct 203388 TTGTTCCATGATCTATGGGTCTACTGGAGCTACCCACTTCGATCAATTAGCCAAGATTTT 203447

Query 492791 GACCGGATACGAAATCACTGGTGCTCCATCTAGTGGTATTTTGATGGGGATTATCTTTAT 492850

||||||||||||||||||||||||||||||||||||||||||||||||||||||||||||

Sbjct 203448 GACCGGATACGAAATCACTGGTGCTCCATCTAGTGGTATTTTGATGGGGATTATCTTTAT 203507

Query 492851 CGCTGTAGGATTCCTATTCAAGATCACTGCAGTTCCTTTTCGGGCGGCTGATGGACGGAC 492910

||||||||||||||||||||||||||||||||||||||||||||||||||||||||||||

Sbjct 203508 CGCTGTAGGATTCCTATTCAAGATCACTGCAGTTCCTTTTCGGGCGGCTGATGGACGGAC 203567

Query 492911 GGCCCCCTATAGGTAGTAGGGTAGGATGGGTGGTACCGCTCAGATTGCGGCCAATCTTCC 492970

||||||||||||||||||||||||||||||||||||||||||||||||||||||||||||

Sbjct 203568 GGCCCCCTATAGGTAGTAGGGTAGGATGGGTGGTACCGCTCAGATTGCGGCCAATCTTCC 203627

Query 492971 TAACTGCGCGCGGGCCGGGCTTAGAGCGCGTGAAACTCATCACTACCTCGTCAGAGCTAA 493030

||||||||||||||||||||||||||||||||||||||||||||||||||||||||||||

Sbjct 203628 TAACTGCGCGCGGGCCGGGCTTAGAGCGCGTGAAACTCATCACTACCTCGTCAGAGCTAA 203687

Query 493031 GAGACCATAGCATGTTACACAAAAGCGCTGCTTTCTCTGGAGTGTTTTCACACAGCTGCC 493090

||||||||||||||||||||||||||||||||||||||||||||||||||||||||||||

Sbjct 203688 GAGACCATAGCATGTTACACAAAAGCGCTGCTTTCTCTGGAGTGTTTTCACACAGCTGCC 203747

Query 493091 CGACTAGAAGAGCTACTCGCTCTGTAGTGTTGTCACACAAGATAAGCACTAGCCCGCCTG 493150

||||||||||||||||||||||||||||||||||||||||||||||||||||||||||||

Sbjct 203748 CGACTAGAAGAGCTACTCGCTCTGTAGTGTTGTCACACAAGATAAGCACTAGCCCGCCTG 203807

Query 493151 CTGGCCGGGCGAATCGAAGTTCTCTTCCGGTCAACTGTACACCCAGTCAAGTGAAAAAAC 493210

||||||||||||||||||||||||||||||||||||||||||||||||||||||||||||

Sbjct 203808 CTGGCCGGGCGAATCGAAGTTCTCTTCCGGTCAACTGTACACCCAGTCAAGTGAAAAAAC 203867

Query 493211 ACAGTGGAATCACGCAACGCACGCTGCTGGTGTGCTTCCTGCCCACGAGGAAAGAAGAGC 493270

||||||||||||||||||||||||||||||||||||||||||||||||||||||||||||

Sbjct 203868 ACAGTGGAATCACGCAACGCACGCTGCTGGTGTGCTTCCTGCCCACGAGGAAAGAAGAGC 203927

Query 493271 GACAAGGTTCAGATTTGACTGTTTGCAGCATGGGAGCTGATGACCCAAGAAATCCCTGGG 493330

||||||||||||||||||||||||||||||||||||||||||||||||||||||||||||

Sbjct 203928 GACAAGGTTCAGATTTGACTGTTTGCAGCATGGGAGCTGATGACCCAAGAAATCCCTGGG 203987

Query 493331 AAAAAAGAAAAGATATCTCGGTAACGAAAACCATAGGAGGCTGTATTGGCGAGATCCAAG 493390

||||||||||||||||||||||||||||||||||||||||||||||||||||||||||||

Sbjct 203988 AAAAAAGAAAAGATATCTCGGTAACGAAAACCATAGGAGGCTGTATTGGCGAGATCCAAG 204047

Query 493391 GGTTCACAGCTGCCCAAAAGAAAAACCGCCTGGAAGTCCGAGGACTTTTAGTACCGTACC 493450

||||||||||||||||||||||||||||||||||||||||||||||||||||||||||||

Sbjct 204048 GGTTCACAGCTGCCCAAAAGAAAAACCGCCTGGAAGTCCGAGGACTTTTAGTACCGTACC 204107

Query 493451 GAACCAGCAGCCTTCGTGCCAAGCGCCGACCGCTCTTGTCCCTTCCtttttttCCATTCA 493510

||||||||||||||||||||||||||||||||||||||||||||||||||||||||||||

Sbjct 204108 GAACCAGCAGCCTTCGTGCCAAGCGCCGACCGCTCTTGTCCCTTCCTTTTTTTCCATTCA 204167

Query 493511 GCCTACTTCTTCGCTTTCTTCCGTCAGTCTAAGGCAAAGCTTAAGAAGTGCTTCGCCTAT 493570

||||||||||||||||||||||||||||||||||||||||||||||||||||||||||||

Sbjct 204168 GCCTACTTCTTCGCTTTCTTCCGTCAGTCTAAGGCAAAGCTTAAGAAGTGCTTCGCCTAT 204227

Query 493571 CTCACTTGACGAAAGAAAGGGAACCAACTCCCTTTCCTTGACGGCTTGATGGAGATATTC 493630

||||||||||||||||||||||||||||||||||||||||||||||||||||||||||||

Sbjct 204228 CTCACTTGACGAAAGAAAGGGAACCAACTCCCTTTCCTTGACGGCTTGATGGAGATATTC 204287

Query 493631 AGTCAGCCTCATTCCTTACTTGTCTGTAAAAAGGAAGTGACGCTTTGCGTCTTCGCGGCC 493690

||||||||||||||||||||||||||||||||||||||||||||||||||||||||||||

Sbjct 204288 AGTCAGCCTCATTCCTTACTTGTCTGTAAAAAGGAAGTGACGCTTTGCGTCTTCGCGGCC 204347

Query 493691 GAGGGTTAGGGTATAGCCCCTTTCGTTCGAGCAAGCTTTCGCTTTTTCGGCTTTCCACAA 493750

||||||||||||||||||||||||||||||||||||||||||||||||||||||||||||

Sbjct 204348 GAGGGTTAGGGTATAGCCCCTTTCGTTCGAGCAAGCTTTCGCTTTTTCGGCTTTCCACAA 204407

Query 493751 CTGTCGCTTTAGCTTAAAATACCCTTAATTTTAAGTCTTGGTCGCCCGACGAGCTACAGG 493810

||||||||||||||||||||||||||||||||||||||||||||||||||||||||||||

Sbjct 204408 CTGTCGCTTTAGCTTAAAATACCCTTAATTTTAAGTCTTGGTCGCCCGACGAGCTACAGG 204467

Query 493811 AGCGAGCCTATAATGGTAACTATCAAACTGCTTGCCACACTGAAGTACCAAAGGTGCGCG 493870

||||||||||||||||||||||||||||||||||||||||||||||||||||||||||||

Sbjct 204468 AGCGAGCCTATAATGGTAACTATCAAACTGCTTGCCACACTGAAGTACCAAAGGTGCGCG 204527

Query 493871 GAGCTCGGaaaaaatgaaaaaGTTTGCTACTATTTAAAGAAGCCTCTTTACTAATATTCG 493930

||||||||||||||||||||||||||||||||||||||||||||||||||||||||||||

Sbjct 204528 GAGCTCGGAAAAAATGAAAAAGTTTGCTACTATTTAAAGAAGCCTCTTTACTAATATTCG 204587

Query 493931 CTTTCCTTTAGAATGAAAGTAGCTATGAATGAAGCCTAATCTACTGCCGATTCAAAAGGC 493990

||||||||||||||||||||||||||||||||||||||||||||||||||||||||||||

Sbjct 204588 CTTTCCTTTAGAATGAAAGTAGCTATGAATGAAGCCTAATCTACTGCCGATTCAAAAGGC 204647

Query 493991 GACATAGTCGTATGGGTGGGGTCTCTCTGGGGAGCTGCTGCCTTGGGAGGAAATCCTTCC 494050

||||||||||||||||||||||||||||||||||||||||||||||||||||||||||||

Sbjct 204648 GACATAGTCGTATGGGTGGGGTCTCTCTGGGGAGCTGCTGCCTTGGGAGGAAATCCTTCC 204707

Query 494051 ATATaaaaaaaGAACAAAGAAAGGGCGTGGTCCGTGACGAAAATATTTATTGATAAAAAT 494110

||||||||||||||||||||||||||||||||||||||||||||||||||||||||||||

Sbjct 204708 ATATAAAAAAAGAACAAAGAAAGGGCGTGGTCCGTGACGAAAATATTTATTGATAAAAAT 204767

Query 494111 AGGAAAGAGTTTTCTATATAGAAGATAGGGGAGAGGATCCATCTGCGCTTTCTCCATTTT 494170

||||||||||||||||||||||||||||||||||||||||||||||||||||||||||||

Sbjct 204768 AGGAAAGAGTTTTCTATATAGAAGATAGGGGAGAGGATCCATCTGCGCTTTCTCCATTTT 204827

Query 494171 CTTAGATGAGAAATTTCGAGAGAAAGAGCAGTGCGAACTATCTTAATCAAATAAAAGGAG 494230

||||||||||||||||||||||||||||||||||||||||||||||||||||||||||||

Sbjct 204828 CTTAGATGAGAAATTTCGAGAGAAAGAGCAGTGCGAACTATCTTAATCAAATAAAAGGAG 204887

Query 494231 AATCGGGATGAAATAATAGATACATAGACATCTCGACGTCAAAGGATGTATAAATTAAGG 494290

|| |||||||||||||||||||||||||||||||||||||||||||||||||||||||||

Sbjct 204888 AACCGGGATGAAATAATAGATACATAGACATCTCGACGTCAAAGGATGTATAAATTAAGG 204947

Query 494291 CTAGAAAGAAATCTCATCTATATCTTTTCTATCTATGAATATAGTAAAGAGAttttttaa 494350

||||||| |||||||||||||||||||||||||||||||||||||||||||| ||||| |

Sbjct 204948 CTAGAAATAAATCTCATCTATATCTTTTCTATCTATGAATATAGTAAAGAGAGTTTTTCA 205007

Query 494351 ttttttGGTTCCCGCCGAATGGATTGGATCCTTTCTGCTTCTACAAACGAAATGAAGGCC 494410

||||||||||||||||||||||||||||||||||||||||||||||||||||||||||||

Sbjct 205008 TTTTTTGGTTCCCGCCGAATGGATTGGATCCTTTCTGCTTCTACAAACGAAATGAAGGCC 205067

Query 494411 GCGCCCCTCCCGAATGCTTCCCCTATCCTTAAGTTCTCTATAAGAGAATAAAATGCTGCT 494470

||||||||||||||||||||||||||||||||||||||||||||||||||||||||||||

Sbjct 205068 GCGCCCCTCCCGAATGCTTCCCCTATCCTTAAGTTCTCTATAAGAGAATAAAATGCTGCT 205127

Query 494471 CCCCCTCCCTTTGTCTTTTTTCAGTGCAAGACAAGAGAGCGCCCTCTTCtttttttCATC 494530

||||||||||||||||||||||||||||||||||||||||||||||||||||||||||||

Sbjct 205128 CCCCCTCCCTTTGTCTTTTTTCAGTGCAAGACAAGAGAGCGCCCTCTTCTTTTTTTCATC 205187

Query 494531 CCTGCAGCCGGAGGCtttttttCTTGAAGACATGTCGTTTCTAGGGCCCAATCATGTTTG 494590

||||||||||||||||||||||||||||||||||||||||||||||||||||||||||||

Sbjct 205188 CCTGCAGCCGGAGGCTTTTTTTCTTGAAGACATGTCGTTTCTAGGGCCCAATCATGTTTG 205247

Query 494591 AGCCTATGTTCACCAGGGCCAAAAAAGGAGAATTCCTTCATGCCTGCCGACGGGGAATGC 494650

||||||||||||||||||||||||||||||||||||||||||||||||||||||||||||

Sbjct 205248 AGCCTATGTTCACCAGGGCCAAAAAAGGAGAATTCCTTCATGCCTGCCGACGGGGAATGC 205307

Query 494651 TTCCCAAACCGGCTCGACGAAGGGAGGTAGATGCGGCGGGGTAAGGAAAACGCTTTTGGA 494710

||||||||||||||||||||||||||||||||||||||||||||||||||||||||||||

Sbjct 205308 TTCCCAAACCGGCTCGACGAAGGGAGGTAGATGCGGCGGGGTAAGGAAAACGCTTTTGGA 205367

Query 494711 GATGTCGATTTATTTCTTTTTCATCGAAAACGAGGAAGGCCAGGGGTCTCGCAGCCCTCC 494770

||||||||||||||||||||||||||||||||||||||||||||||||||||||||||||

Sbjct 205368 GATGTCGATTTATTTCTTTTTCATCGAAAACGAGGAAGGCCAGGGGTCTCGCAGCCCTCC 205427

Query 494771 TTCGGGCTTTGCCTGAACATTTAGAATAAAGAATTCCGGCTTGGCCCGGGCGGGAAAGCG 494830

||||||||||||||||||||||||||||||||||||||||||||||||||||||||||||

Sbjct 205428 TTCGGGCTTTGCCTGAACATTTAGAATAAAGAATTCCGGCTTGGCCCGGGCGGGAAAGCG 205487

Query 494831 CTGGCAACAACATAAAGGAAAGGGGTCCGTGTAGCTGCTGCGCCCGCCCACGGAGCAGCA 494890

||||||||||||||||||||||||||||||||||||||||||||||||||||||||||||

Sbjct 205488 CTGGCAACAACATAAAGGAAAGGGGTCCGTGTAGCTGCTGCGCCCGCCCACGGAGCAGCA 205547

Query 494891 GGTTCGGCATCTACTaaaaaaaaGAGAGAATCCTTTAAAGTCAGATATCCACGTCTCTGC 494950

||||||||||||||||||||||||||||||||||||||||||||||||||||||||||||

Sbjct 205548 GGTTCGGCATCTACTAAAAAAAAGAGAGAATCCTTTAAAGTCAGATATCCACGTCTCTGC 205607

Query 494951 TAGAGGCAGCGTGTGGAATGGCCGAGAGCGGATCAAATGGATATATAATCCAAGCCAAGA 495010

||||||||||||||||||||||||||||||||||||||||||||||||||||||||||||

Sbjct 205608 TAGAGGCAGCGTGTGGAATGGCCGAGAGCGGATCAAATGGATATATAATCCAAGCCAAGA 205667

Query 495011 GTGGAGTTACGTGAACAGCCGTCTGATGGaaaaaaaCTTTCACGTTCGGTTCAGAGAGCA 495070

||||||||||||||||||||||||||||||||||||||||||||||||||||||||||||

Sbjct 205668 GTGGAGTTACGTGAACAGCCGTCTGATGGAAAAAAACTTTCACGTTCGGTTCAGAGAGCA 205727

Query 495071 CTTTTTTCGTCGAGAATAAAACCTTCCCTTTTGTGTGAATTCCGCAGCGGCGAATTAACA 495130

||||||||||||||||||||||||||||||||||||||||||||||||||||||||||||

Sbjct 205728 CTTTTTTCGTCGAGAATAAAACCTTCCCTTTTGTGTGAATTCCGCAGCGGCGAATTAACA 205787

Query 495131 ACTTGTGGGGCCCCATCTATTCAATCTCGCGAGCCCCCAAGAAATCAAACCCCCTCTCCC 495190

||||||||||||||||||||||||||||||||||||||||||||||||||||||||||||

Sbjct 205788 ACTTGTGGGGCCCCATCTATTCAATCTCGCGAGCCCCCAAGAAATCAAACCCCCTCTCCC 205847

Query 495191 TCGGACTCAATCTCTCTTTTGACTCTATATATGTGGGCACCTGATATCTATGAGGGTTCA 495250

||||||||||||||||||||||||||||||||||||||||||||||||||||||||||||

Sbjct 205848 TCGGACTCAATCTCTCTTTTGACTCTATATATGTGGGCACCTGATATCTATGAGGGTTCA 205907

Query 495251 CCCACCCCGGTGACAGCATTCCTTTCTATTGCGCCGAAAATCTCTATTTTTGCAAATATG 495310

||||||||||||||||||||||||||||||||||||||||||||||||||||||||||||

Sbjct 205908 CCCACCCCGGTGACAGCATTCCTTTCTATTGCGCCGAAAATCTCTATTTTTGCAAATATG 205967

Query 495311 TTACGTGTTTTTATTGTTGCTTCCTATGGAGGTACATTGCAACAAATCTTCTTTTTCTGC 495370

||||||||||||||||||||||||||||||||||||||||||||||||||||||||||||

Sbjct 205968 TTACGTGTTTTTATTGTTGCTTCCTATGGAGGTACATTGCAACAAATCTTCTTTTTCTGC 206027

Query 495371 AGCATTGCTTCTATGATCTTAGGAGCACTGGCCGCCATGGCCCAAACGAAAGTCAAAAGA 495430

||||||||||||||||||||||||||||||||||||||||||||||||||||||||||||

Sbjct 206028 AGCATTGCTTCTATGATCTTAGGAGCACTGGCCGCCATGGCCCAAACGAAAGTCAAAAGA 206087

Query 495431 CTTCTAGCTTATAGTTCGATTGGACATGTAGGTTATATTTGTATAGGTTTATCATGTGGA 495490

||||||||||||||||||||||||||||||||||||||||||||||||||||||||||||

Sbjct 206088 CTTCTAGCTTATAGTTCGATTGGACATGTAGGTTATATTTGTATAGGTTTATCATGTGGA 206147

Query 495491 ACCATAGAAGGAATTCAATCTCTACTCATTGGTATATTTATTTATGCATCAATGACGATA 495550

||||||||||||||||||||||||||||||||||||||||||||||||||||||||||||

Sbjct 206148 ACCATAGAAGGAATTCAATCTCTACTCATTGGTATATTTATTTATGCATCAATGACGATA 206207

Query 495551 GATGCATTCGCCATAGTTTTAGCATTACGCCAAACCCGTGTAAAATATATAGCGGATTTG 495610

||||||||||||||||||||||||||||||||||||||||||||||||||||||||||||

Sbjct 206208 GATGCATTCGCCATAGTTTTAGCATTACGCCAAACCCGTGTAAAATATATAGCGGATTTG 206267

Query 495611 GGCGCTCTTGCCAAAACGAATCCTATTTTGGCAATTACCTTCTCCATGACAATGTTTTCA 495670

||||||||||||||||||||||||||||||||||||||||||||||||||||||||||||

Sbjct 206268 GGCGCTCTTGCCAAAACGAATCCTATTTTGGCAATTACCTTCTCCATGACAATGTTTTCA 206327

Query 495671 TACGCAGGAATACCCCCGTTAGCCGGCTTTTGTAGCAAATTCTATTTGTTCTTCGCCGCT 495730

||||||||||||||||||||||||||||||||||||||||||||||||||||||||||||

Sbjct 206328 TACGCAGGAATACCCCCGTTAGCCGGCTTTTGTAGCAAATTCTATTTGTTCTTCGCCGCT 206387

Query 495731 TTGGGTTGTGGGGCTTACTTCCTAGCCTTAGTGGGAGTAGTGACTAGCGTTATAGGTTGT 495790

||||||||||||||||||||||||||||||||||||||||||||||||||||||||||||

Sbjct 206388 TTGGGTTGTGGGGCTTACTTCCTAGCCTTAGTGGGAGTAGTGACTAGCGTTATAGGTTGT 206447

Query 495791 TGGGCGGCCGGAAGGTTGCCATGAGTAAGGTTGGGGACCAAAAGAAGTTTTCCGTGCACC 495850

||||||||||||||||||||||||||||||||||||||||||||||||||||||||||||

Sbjct 206448 TGGGCGGCCGGAAGGTTGCCATGAGTAAGGTTGGGGACCAAAAGAAGTTTTCCGTGCACC 206507

Query 495851 GGACACGTAGCTTACCGAATCAGTTGCAACACAGATGGGAATGCATGCTACGAAAGAACA 495910

||||||||||||||||||||||||||||||||||||||||||||||||||||||||||||

Sbjct 206508 GGACACGTAGCTTACCGAATCAGTTGCAACACAGATGGGAATGCATGCTACGAAAGAACA 206567

Query 495911 GGTCGAGTCTTATACATCAACCGTCGACTCCATATCCTTGTACGAGTCCACAATCACTAC 495970

||||||||||||||||||||||||||||||||||||||||||||||||||||||||||||

Sbjct 206568 GGTCGAGTCTTATACATCAACCGTCGACTCCATATCCTTGTACGAGTCCACAATCACTAC 206627

Query 495971 ACGAGATGAACCTGGGTTTGGTGAATGAATGGGAGTTGGCGTTAGGTGGTTTAGGACCCC 496030

||||||||||||||||||||||||||||||||||||||||||||||||||||||||||||

Sbjct 206628 ACGAGATGAACCTGGGTTTGGTGAATGAATGGGAGTTGGCGTTAGGTGGTTTAGGACCCC 206687

Query 496031 CAGTTACTGCGCGCGATCGTATACTGAGGTGCTACCCGCCGTCGGTCGTTGGAACGACGC 496090

||||||||||||||||||||||||||||||||||||||||||||||||||||||||||||

Sbjct 206688 CAGTTACTGCGCGCGATCGTATACTGAGGTGCTACCCGCCGTCGGTCGTTGGAACGACGC 206747

Query 496091 GAGCCGGTGATTCCTTTCATAAAGATGAAGGGTCAGAGGTAAAAAATTCCCATCTAATTT 496150

||||||||||||||||||||||||||||||||||||||||||||||||||||||||||||

Sbjct 206748 GAGCCGGTGATTCCTTTCATAAAGATGAAGGGTCAGAGGTAAAAAATTCCCATCTAATTT 206807

Query 496151 GGGGCGGAAAACGAATCGACATCTCGATGTGAGAAAGCCCTTTCCCCTTTTTCGTTGGGA 496210

||||||||||||||||||||||||||||||||||||||||||||||||||||||||||||

Sbjct 206808 GGGGCGGAAAACGAATCGACATCTCGATGTGAGAAAGCCCTTTCCCCTTTTTCGTTGGGA 206867

Query 496211 AATAACGGCGAAATCCATCCGAACCGTCCAATGAAGAAATAAGAGGAGAGCAAAGCGCCA 496270

||||||||||||||||||||||||||||||||||||||||||||||||||||||||||||

Sbjct 206868 AATAACGGCGAAATCCATCCGAACCGTCCAATGAAGAAATAAGAGGAGAGCAAAGCGCCA 206927

Query 496271 ATGGCGCGCGAAGCGCATGCAGAAGCGGCGCGGAGAAATCAAGAAgggggggAGAAAAGC 496330

||||||||||||||||||||||||||||||||||||||||||||||||||||||||||||

Sbjct 206928 ATGGCGCGCGAAGCGCATGCAGAAGCGGCGCGGAGAAATCAAGAAGGGGGGGAGAAAAGC 206987

Query 496331 CGAGCTCTTTCCCTTCGCTTTCTGGACCAAAGAAAGCAGTGCTTTATTTCCTGGAAAACG 496390

||||||||||||||||||||||||||||||||||||||||||||||||||||||||||||

Sbjct 206988 CGAGCTCTTTCCCTTCGCTTTCTGGACCAAAGAAAGCAGTGCTTTATTTCCTGGAAAACG 207047

Query 496391 AAATCAAGGATTTGGCTTGGCAAGCAAAAGAGATATTAATCGAAATAAAGATAGATCCTT 496450

||||||||||||||||||||||||||||||||||||||||||||||||||||||||||||

Sbjct 207048 AAATCAAGGATTTGGCTTGGCAAGCAAAAGAGATATTAATCGAAATAAAGATAGATCCTT 207107

Query 496451 CTATCTTCCGGATATCTaaaaaaaaTCGATTTCATGAAACCTTTTATTAAAAGAAGCTTA 496510

||||||||||||||||||||||||||||||||||||||||||||||||||||||||||||

Sbjct 207108 CTATCTTCCGGATATCTAAAAAAAATCGATTTCATGAAACCTTTTATTAAAAGAAGCTTA 207167

Query 496511 GCCCCCTCTCATGAAACGGCTCTGCTGCAATGGATGGCAGAGGGTCCGTAGTACCCGAAG 496570

||||||||||||||||||||||||||||||||||||||||||||||||||||||||||||

Sbjct 207168 GCCCCCTCTCATGAAACGGCTCTGCTGCAATGGATGGCAGAGGGTCCGTAGTACCCGAAG 207227

Query 496571 CACTGGAGTGATCAAGTAGCCGGGAAGGGGCCTAGAAGTGCCTACTACTACACCACCACA 496630

||||||||||||||||||||||||||||||||||||||||||||||||||||||||||||

Sbjct 207228 CACTGGAGTGATCAAGTAGCCGGGAAGGGGCCTAGAAGTGCCTACTACTACACCACCACA 207287

Query 496631 CTCAACTTGGCTCCACACATTGAAAGATCTTACTCCTTTCCAGTGTCTGGCAGGGCAGAA 496690

||||||||||||||||||||||||||||||||||||||||||||||||||||||||||||

Sbjct 207288 CTCAACTTGGCTCCACACATTGAAAGATCTTACTCCTTTCCAGTGTCTGGCAGGGCAGAA 207347

Query 496691 CATTGGGGGCTTCAATCCTGACTCCTTTTCCCCATCTAAGCCCAGGCTAACGGAGCCTGA 496750

||||||||||||||||||||||||||||||||||||||||||||||||||||||||||||

Sbjct 207348 CATTGGGGGCTTCAATCCTGACTCCTTTTCCCCATCTAAGCCCAGGCTAACGGAGCCTGA 207407

Query 496751 CTGATTCAGGGGAGAGAGAGAATGAATGGAGCCGCCTATCGAAGCACTCTTGAGAGTAAG 496810

||||||||||||||||||||||||||||||||||||||||||||||||||||||||||||

Sbjct 207408 CTGATTCAGGGGAGAGAGAGAATGAATGGAGCCGCCTATCGAAGCACTCTTGAGAGTAAG 207467

Query 496811 ATCCTTCCTTGGCTCCTCTTCATTCAGGTCTATGTCCGGGGCAAAGATGGAAAAGATCAA 496870

||||||||||||||||||||||||||||||||||||||||||||||||||||||||||||

Sbjct 207468 ATCCTTCCTTGGCTCCTCTTCATTCAGGTCTATGTCCGGGGCAAAGATGGAAAAGATCAA 207527

Query 496871 AGAAAGGAATAAGAACAAAGTTCGCCttttttttATCATTTTTATAGAGAGAGGGGGATG 496930

||||||||||||||||||||||||||||||||||||||||||||||||||||||||||||

Sbjct 207528 AGAAAGGAATAAGAACAAAGTTCGCCTTTTTTTTATCATTTTTATAGAGAGAGGGGGATG 207587

Query 496931 GAAAAAGTGGACAAAACAGACTCACATTTGCCATTATAGGCTCGCAGGCTTCCCCTTTTT 496990

||||||||||||||||||||||||||||||||||||||||||||||||||||||||||||

Sbjct 207588 GAAAAAGTGGACAAAACAGACTCACATTTGCCATTATAGGCTCGCAGGCTTCCCCTTTTT 207647

Query 496991 CGTCTCGACAAAGAAAGAATCAGAAAAACGCTCCTAACCCCTTCGTAGGGCCTTGTATGG 497050

||||||||||||||||||||||||||||||||||||||||||||||||||||||||||||

Sbjct 207648 CGTCTCGACAAAGAAAGAATCAGAAAAACGCTCCTAACCCCTTCGTAGGGCCTTGTATGG 207707

Query 497051 TAAGTGATCCGAACCCGCCCGGAGCGAGCCCCCCTGAGAGGCAAGTGAAGTTGGTGAGCC 497110

||||||||||||||||||||||||||||||||||||||||||||||||||||||||||||

Sbjct 207708 TAAGTGATCCGAACCCGCCCGGAGCGAGCCCCCCTGAGAGGCAAGTGAAGTTGGTGAGCC 207767

Query 497111 GTATGATGGGCGACTATCTCCTGCGGTTCGGAGAGGACTCAGCTCTTAGTTAGTAccccc 497170

||||||||||||||||||||||||||||||||||||||||||||||||||||||||||||

Sbjct 207768 GTATGATGGGCGACTATCTCCTGCGGTTCGGAGAGGACTCAGCTCTTAGTTAGTACCCCC 207827

Query 497171 ccTTTCGGGGTGGACCCTTTCACTCTATTTTATTATATACGCTTAGTTAAAAGAATGTTT 497230

||||||||||||||||||||||||||||||||||||||||||||||||||||||||||||

Sbjct 207828 CCTTTCGGGGTGGACCCTTTCACTCTATTTTATTATATACGCTTAGTTAAAAGAATGTTT 207887

Query 497231 TTTGATAAACCTAGGACATGGATTCTTTATGAACCAATGGATCGTGAAAAGTCGTTACTA 497290

||||||||||||||||||||||||||||||||||||||||||||||||||||||||||||

Sbjct 207888 TTTGATAAACCTAGGACATGGATTCTTTATGAACCAATGGATCGTGAAAAGTCGTTACTA 207947

Query 497291 CTAGCAATGACTTCCTCTTTCATTACTTTATTCTTTCCATATCCCTCTCCCTTGTTCGAT 497350

||||||||||||||||||||||||||||||||||||||||||||||||||||||||||||

Sbjct 207948 CTAGCAATGACTTCCTCTTTCATTACTTTATTCTTTCCATATCCCTCTCCCTTGTTCGAT 208007

Query 497351 CTTACTCATCAAATGGCACTCAGTTCATATCTGTAAATTCGATCATTTACAAGGTTCAAA 497410

||||||||||||||||||||||||||||||||||||||||||||||||||||||||||||

Sbjct 208008 CTTACTCATCAAATGGCACTCAGTTCATATCTGTAAATTCGATCATTTACAAGGTTCAAA 208067

Query 497411 GAAAGGGTGGACTGCAGGTAAGCACTAACGCTACGCGCCTCTGGATTTCCGTGATGAGGG 497470

||||||||||||||||||||||||||||||||||||||||||||||||||||||||||||

Sbjct 208068 GAAAGGGTGGACTGCAGGTAAGCACTAACGCTACGCGCCTCTGGATTTCCGTGATGAGGG 208127

Query 497471 CGTACATAGCCCATTGTAGATTCCAGCCGAGAAGACTTAGGATTTGTGACAGCTACCATG 497530

||||||||||||||||||||||||||||||||||||||||||||||||||||||||||||

Sbjct 208128 CGTACATAGCCCATTGTAGATTCCAGCCGAGAAGACTTAGGATTTGTGACAGCTACCATG 208187

Query 497531 TTACTTGCTAGAATTTGCCTTTATCTACTCCAAAACTGGAGGCATCCATCTATTCATTCA 497590

||||||||||||||||||||||||||||||||||||||||||||| ||||||||||||||

Sbjct 208188 TTACTTGCTAGAATTTGCCTTTATCTACTCCAAAACTGGAGGCATTCATCTATTCATTCA 208247

Query 497591 AAAACTACTCCTAGTGAAGTTTTCCATGTGATACGCAGCTTACTAAAAGGGGGGTTACAC 497650

||||||||||||||||||||||||||||||||||||||||||||||||||||||||||||

Sbjct 208248 AAAACTACTCCTAGTGAAGTTTTCCATGTGATACGCAGCTTACTAAAAGGGGGGTTACAC 208307

Query 497651 CTTCTCGAGCAGGCGTTGGTCAATACGGCATCCTACCAAGCCAGATAAATTATGGCCGAT 497710

||||||||||||||||||||||||||||||||||||||||||||||||||||||||||||

Sbjct 208308 CTTCTCGAGCAGGCGTTGGTCAATACGGCATCCTACCAAGCCAGATAAATTATGGCCGAT 208367

Query 497711 AGGCGCTTTGGATGCTGTCATAGTCGAAGCAATGGCCCGCTTGCTTAGTTTGGAAATGGA 497770

||||||||||||||||||||||||||||||||||||||||||||||||||||||||||||

Sbjct 208368 AGGCGCTTTGGATGCTGTCATAGTCGAAGCAATGGCCCGCTTGCTTAGTTTGGAAATGGA 208427

Query 497771 CTCAATATACCATCTATATTCCAGGGAGTATGGGGTAGCGAGGGGTGCTAGGTCTTTCAT 497830

||||||||||||||||||||||||||||||||||||||||||||||||||||||||||||

Sbjct 208428 CTCAATATACCATCTATATTCCAGGGAGTATGGGGTAGCGAGGGGTGCTAGGTCTTTCAT 208487

Query 497831 CGCAAGCGTTTCCCGATGGCCTGAGATGGACTGGCTGATCCAATGCGACCAATAAGTAAG 497890

||||||||||||||||||||||||||||||||||||||||||||||||||||||||||||

Sbjct 208488 CGCAAGCGTTTCCCGATGGCCTGAGATGGACTGGCTGATCCAATGCGACCAATAAGTAAG 208547

Query 497891 TAGGTGTATAAGTCTAAGCATCCAAAGGGACCAGCCCCGCAATCAGAGCTGTGGGGAGCG 497950

||||||||||||||||||||||||||||||||||||||||||||||||||||||||||||

Sbjct 208548 TAGGTGTATAAGTCTAAGCATCCAAAGGGACCAGCCCCGCAATCAGAGCTGTGGGGAGCG 208607

Query 497951 AGAACTAGCTATAAGAATACTGGGAATAGGGTAACTCCGATTATCCGTGGTGCTATCTAT 498010

||||||||||||||||||||||||||||||||||||||||||||||||||||||||||||

Sbjct 208608 AGAACTAGCTATAAGAATACTGGGAATAGGGTAACTCCGATTATCCGTGGTGCTATCTAT 208667

Query 498011 TCCCTGGAGCAGTAATTAAAAGAAAGTACACTAACTTAAATCCGAGTTGGACACCCATAC 498070

||||||||||||||||||||||||||||||||||||||||||||||||||||||||||||

Sbjct 208668 TCCCTGGAGCAGTAATTAAAAGAAAGTACACTAACTTAAATCCGAGTTGGACACCCATAC 208727

Query 498071 ATAGATGATTCTCTCTCTGAGCAAATGTCAGAGTTGGACAGGGACCCCACCCACTTCTTT 498130

||||||||||||||||||||||||||||||||||||||||||||||||||||||||||||

Sbjct 208728 ATAGATGATTCTCTCTCTGAGCAAATGTCAGAGTTGGACAGGGACCCCACCCACTTCTTT 208787

Query 498131 CCGTCTGGTAATTCCTTCGTTAAAGCGGCGGCAACCTGGGAACTACTTAGCTTAGGGGAT 498190

||||||||||||||||||||||||||||||||||||||||||||||||||||||||||||

Sbjct 208788 CCGTCTGGTAATTCCTTCGTTAAAGCGGCGGCAACCTGGGAACTACTTAGCTTAGGGGAT 208847

Query 498191 AATATTAGATCGGGTAGCGAGCGCTCTCCTCTGTCAGGTAATCCCCTCCTCCCCTTAAGT 498250

||||||||||||||||||||||||||||||||||||||||||||||||||||||||||||

Sbjct 208848 AATATTAGATCGGGTAGCGAGCGCTCTCCTCTGTCAGGTAATCCCCTCCTCCCCTTAAGT 208907

Query 498251 AAATGCCAGCTAAAAGCTTCAAAGCCCGGCTAGAATAAATAGGTGACCAAGCGGGTGCTT 498310

||||||||||||||||||||||||||||||||||||||||||||||||||||||||||||

Sbjct 208908 AAATGCCAGCTAAAAGCTTCAAAGCCCGGCTAGAATAAATAGGTGACCAAGCGGGTGCTT 208967

Query 498311 TCTACACAGATTCAGAGAAAGAGTAACCACTAACGCACGTAATATCAAGAGTGGAATGAT 498370

||||||||||||||||||||||||||||||||||||||||||||||||||||||||||||

Sbjct 208968 TCTACACAGATTCAGAGAAAGAGTAACCACTAACGCACGTAATATCAAGAGTGGAATGAT 209027

Query 498371 TTAAGTACATAATTTAATCGCTCTCCAATACGCTGACGACACCCTAATTTGTAAGGAGTA 498430

||||||||||||||||||||||||||||||||||||||||||||||||||||||||||||

Sbjct 209028 TTAAGTACATAATTTAATCGCTCTCCAATACGCTGACGACACCCTAATTTGTAAGGAGTA 209087

Query 498431 TATGGTATAAGCTCTCAAGTGGGCACATAAAAGAAAATTCCTTCTTTACTGAATTCATTA 498490

||||||||||||||||||||||||||||||||||||||||||||||||||||||||||||

Sbjct 209088 TATGGTATAAGCTCTCAAGTGGGCACATAAAAGAAAATTCCTTCTTTACTGAATTCATTA 209147

Query 498491 CAATGTGTTTATGCATGCTTGTTTATTCGATTTCTACAATATGGGATAGAGTAGTGGATT 498550

||||||||||||||||||||||||||||||||||||||||||||||||||||||||||||

Sbjct 209148 CAATGTGTTTATGCATGCTTGTTTATTCGATTTCTACAATATGGGATAGAGTAGTGGATT 209207

Query 498551 TTTTAAGAATAAAGCAAGGATAAGAAAGTGGGCACACAATTCAATTAAGAATGAGCAGAA 498610

||||||||||||||||||||||||||||||||||||||||||||||||||||||||||||

Sbjct 209208 TTTTAAGAATAAAGCAAGGATAAGAAAGTGGGCACACAATTCAATTAAGAATGAGCAGAA 209267

Query 498611 AACAAAGCTGCTGATGCTTTGTCAAGAAGAGCAGAGCAAAAGTGGAAATAGAGAGGGGAA 498670

||||||||||||||||||||||||||||||||||||||||||||||||||||||||||||

Sbjct 209268 AACAAAGCTGCTGATGCTTTGTCAAGAAGAGCAGAGCAAAAGTGGAAATAGAGAGGGGAA 209327

Query 498671 AAGTTAAAGCAGTAACTGAGGTGTTGCCAAAGTGGATAGTATATATTGCATTGTTGTCCA 498730

||||||||||||||||||||||||||||||||||||||||||||||||||||||||||||

Sbjct 209328 AAGTTAAAGCAGTAACTGAGGTGTTGCCAAAGTGGATAGTATATATTGCATTGTTGTCCA 209387

Query 498731 CTTGGGAACCAAATTCGCTCGCACTGGAGTTCCATGCATCGCGCAAGCTCCGCTCGGCAC 498790

||||||||||||||||||||||||||||||||||||||||||||||||||||||||||||

Sbjct 209388 CTTGGGAACCAAATTCGCTCGCACTGGAGTTCCATGCATCGCGCAAGCTCCGCTCGGCAC 209447

Query 498791 TTGTAGAACATGCAGTTCGTATAGGACGAAGCttttttttttCCATAGGAAGGCATGTTA 498850

||||||||||||||||||||||||||||||||||||||||||||||||||||||||||||

Sbjct 209448 TTGTAGAACATGCAGTTCGTATAGGACGAAGCTTTTTTTTTTCCATAGGAAGGCATGTTA 209507

Query 498851 TCAAGGTTGGGTTCTTGTACTTGGAATCGTGGTCAGCATCAAGCTCGTTTTCCCACAGCG 498910

||||||||||||||||||||||||||||||||||||||||||||||||||||||||||||

Sbjct 209508 TCAAGGTTGGGTTCTTGTACTTGGAATCGTGGTCAGCATCAAGCTCGTTTTCCCACAGCG 209567

Query 498911 TCATCAATGCAGCATCAAACTTCACCACAAACTTTGCTAGGGTAGCCTTATTGGAAAGGT 498970

||||||||||||||||||||||||||||||||||||||||||||||||||||||||||||

Sbjct 209568 TCATCAATGCAGCATCAAACTTCACCACAAACTTTGCTAGGGTAGCCTTATTGGAAAGGT 209627

Query 498971 ATTTGTTAAAAAATTCATACTCTCAGCCCTTTGTGTGGTGGTAATACCAGCCCAAAATCG 499030

||||||||||||||||||||||||||||||||||||||||||||||||||||||||||||

Sbjct 209628 ATTTGTTAAAAAATTCATACTCTCAGCCCTTTGTGTGGTGGTAATACCAGCCCAAAATCG 209687

Query 499031 AGAATTTAGAAATACCGGCAcccccccTTATTTCAAACAGCTCATTGAACCATTAATTTT 499090

||||||||||||||||||||||||||||||||||||||||||||||||||||||||||||

Sbjct 209688 AGAATTTAGAAATACCGGCACCCCCCCTTATTTCAAACAGCTCATTGAACCATTAATTTT 209747

Query 499091 TCTGATAACCGAGATCTGCCATTAAATTATTCCATCTACCTTCAAATTCTTGTGTTGTGA 499150

||||||||||||||||||||||||||||||||||||||||||||||||||||||||||||

Sbjct 209748 TCTGATAACCGAGATCTGCCATTAAATTATTCCATCTACCTTCAAATTCTTGTGTTGTGA 209807

Query 499151 TTGAACCATACACCACAGAGTCTGAAAGAGTACTTTTATTGGGTACACGCCCCCATTTAA 499210

||||||||||||||||||||||||||||||||||||||||||||||||||||||||||||

Sbjct 209808 TTGAACCATACACCACAGAGTCTGAAAGAGTACTTTTATTGGGTACACGCCCCCATTTAA 209867

Query 499211 TAGGCAACTTGTGGAGAATGTGCCAAGAACAGTAAGGTTTCACAGTACTTGAAAAAACAT 499270

||||||||||||||||||||||||||||||||||||||||||||||||||||||||||||

Sbjct 209868 TAGGCAACTTGTGGAGAATGTGCCAAGAACAGTAAGGTTTCACAGTACTTGAAAAAACAT 209927

Query 499271 TTTTAATACCCTTCTCGAAGAGTCCCAAGGAAAAAGAGATGTTCTGTGGAAGTATCCTCT 499330

||||||||||||||||||||||||||||||||||||||||||||||||||||||||||||

Sbjct 209928 TTTTAATACCCTTCTCGAAGAGTCCCAAGGAAAAAGAGATGTTCTGTGGAAGTATCCTCT 209987

Query 499331 GGTGATGTACCTATAAATGCTGTTTAGGTAATTTCTTTTTCTATTTACAAACTaaaaaaa 499390

||||||||||||||||||||||||||||||||||||||||||||||||||||||||||||

Sbjct 209988 GGTGATGTACCTATAAATGCTGTTTAGGTAATTTCTTTTTCTATTTACAAACTAAAAAAA 210047

Query 499391 aGCTATACATCAGAGAAGCTATAGTCCATCATCTTCTTTTGAGGAATAGCTTCTCCTAAC 499450

||||||||||||||||||||||||||||||||||||||||||||||||||||||||||||

Sbjct 210048 AGCTATACATCAGAGAAGCTATAGTCCATCATCTTCTTTTGAGGAATAGCTTCTCCTAAC 210107

Query 499451 CTTATAATGGACACACTAGGAACTCTTGCGTTGGCTACTGAACCACCTACTGACAAGCTC 499510

||||||||||||||||||||||||||||||||||||||||||||||||||||||||||||

Sbjct 210108 CTTATAATGGACACACTAGGAACTCTTGCGTTGGCTACTGAACCACCTACTGACAAGCTC 210167

Query 499511 ATGAAACGGGCTCCTGTAGGTCGTAGGTACTTACTTTTTTACTCTTCCGCTCAGTTGTAA 499570

||||||||||||||||||||||||||||||||||||||||||||||||||||||||||||

Sbjct 210168 ATGAAACGGGCTCCTGTAGGTCGTAGGTACTTACTTTTTTACTCTTCCGCTCAGTTGTAA 210227

Query 499571 TATTTCGTCTCATACTTTCTTAAATGATTCGAATTTTCGGGAGACAATTTTGACGTTTTA 499630

||||||||||||||||||||||||||||||||||||||||||||||||||||||||||||

Sbjct 210228 TATTTCGTCTCATACTTTCTTAAATGATTCGAATTTTCGGGAGACAATTTTGACGTTTTA 210287

Query 499631 CATGAATGAAAGAATTTGTAAACCATTTCGTTGAAGTACCTGGTACATTCCCATTTTCTG 499690

||||||||||||||||||||||||||||||||||||||||||||||||||||||||| ||

Sbjct 210288 CATGAATGAAAGAATTTGTAAACCATTTCGTTGAAGTACCTGGTACATTCCCATTTTATG 210347

Query 499691 ATTTGGAGTGGGTATCCCTCCCCAAAGAAGCAGGTTGGTGCTCCATTAATTATCTTAAAT 499750

||||||||||||||||||||||||||||||||||||||||||||||||||||||||||||

Sbjct 210348 ATTTGGAGTGGGTATCCCTCCCCAAAGAAGCAGGTTGGTGCTCCATTAATTATCTTAAAT 210407

Query 499751 GACTGATACCTTCGTTGCAAGAAATTCCAAAAGAAGCACCTTGTTGATTTTTGCGTTTCA 499810

||||||||||||||||||||||||||||||||||||||||||||||||||||||||||||

Sbjct 210408 GACTGATACCTTCGTTGCAAGAAATTCCAAAAGAAGCACCTTGTTGATTTTTGCGTTTCA 210467

Query 499811 TGAGAGCTGACAGCTGAGTGCTTGGTGAACCTACCTTCTTAAATTATTCAATAGGGTTCT 499870

||||||||||||||||||||||||||||||||||||||||||||||||||||||||||||

Sbjct 210468 TGAGAGCTGACAGCTGAGTGCTTGGTGAACCTACCTTCTTAAATTATTCAATAGGGTTCT 210527

Query 499871 TCCTACCCAACTATCTTGTTCTTGCCCCTCTGATTTTACTGCTTTGACTGGTTTACTTTC 499930

||||||||||||||||||||||||||||||||||||||||||||||||||||||||||||

Sbjct 210528 TCCTACCCAACTATCTTGTTCTTGCCCCTCTGATTTTACTGCTTTGACTGGTTTACTTTC 210587

Query 499931 TGGTTCAGGACGAGTTTCGATATTATTCACTTCCAATTAAATCATAAGCTTGTTGATCTT 499990

||||||||||||||||||||||||||||||||||||||||||||||||||||||||||||

Sbjct 210588 TGGTTCAGGACGAGTTTCGATATTATTCACTTCCAATTAAATCATAAGCTTGTTGATCTT 210647

Query 499991 TGGAATAAATTTCTTTGCTCCCATGCTTGCTGGTAAGGTATGTTTGAAACCGTCAAATCC 500050

||||||||||||||||||||||||||||||||||||||||||||||||||||||||||||

Sbjct 210648 TGGAATAAATTTCTTTGCTCCCATGCTTGCTGGTAAGGTATGTTTGAAACCGTCAAATCC 210707

Query 500051 ACTACAGGCCTCCTGCATTTACTTACGTCAAGCAAAGTTTTGACATTTACAGATCAGTCT 500110

||||||||||||||||||||||||||||||||||||||||||||||||||||||||||||

Sbjct 210708 ACTACAGGCCTCCTGCATTTACTTACGTCAAGCAAAGTTTTGACATTTACAGATCAGTCT 210767

Query 500111 TAGGAGTTTTAAGGGCTCCAGTTACTATCCACTACCCCACCACTTTTCTTATCTTAGTAC 500170

||||||||||||||||||||||||||||||||||||||||||||||||||||||||||||

Sbjct 210768 TAGGAGTTTTAAGGGCTCCAGTTACTATCCACTACCCCACCACTTTTCTTATCTTAGTAC 210827

Query 500171 TGCCACTGCTAAAAGTAAAAGAATGGCTCTTGCTGCTACTGCAGCTATCACGGGGAAGGG 500230

||||||||||||||||||||||||||||||||||||||||||||||||||| ||||||||

Sbjct 210828 TGCCACTGCTAAAAGTAAAAGAATGGCTCTTGCTGCTACTGCAGCTATCACAGGGAAGGG 210887

Query 500231 ACTATTCAAGCGGGTATTTATGTCCGGCCGGGCTTCTCGACTTGTCTGATTGCTGCCCAG 500290

||||||||||||||||||||||||||||||||||||||||||||||||||||||||||||

Sbjct 210888 ACTATTCAAGCGGGTATTTATGTCCGGCCGGGCTTCTCGACTTGTCTGATTGCTGCCCAG 210947

Query 500291 TATGATTTGATCTGATAATCCATGCCTTCTTCCAGAAGCATCTGGCTTATGTAGTGGAGT 500350

||||||||||||||||||||||||||||||||||||||||||||||||||||||||||||

Sbjct 210948 TATGATTTGATCTGATAATCCATGCCTTCTTCCAGAAGCATCTGGCTTATGTAGTGGAGT 211007

Query 500351 TCAATGTTTTCTGTTATCTAACTATCTCTGTTATCTAACTATATTAAGTACAAAAACCTC 500410

||||||||||||||||||||||||||||||||||||||||||||||||||||||||||||

Sbjct 211008 TCAATGTTTTCTGTTATCTAACTATCTCTGTTATCTAACTATATTAAGTACAAAAACCTC 211067

Query 500411 TAAAATTATTTCGGTCGGTAAAAGGAAGTGCATGGATAGTTGCGCTCGGCTGGGGAGGCC 500470

||||||||||||||||||||||||||||||||||||||||||||||||||||||||||||

Sbjct 211068 TAAAATTATTTCGGTCGGTAAAAGGAAGTGCATGGATAGTTGCGCTCGGCTGGGGAGGCC 211127

Query 500471 GGTTGATTGAATGAATTGTACGAAGGCTTGTCTTCAAAGAGGAAAGAATCGTATGTGAGT 500530

||||||||||||||||||||||||||||||||||||||||||||||||||||||||||||

Sbjct 211128 GGTTGATTGAATGAATTGTACGAAGGCTTGTCTTCAAAGAGGAAAGAATCGTATGTGAGT 211187

Query 500531 TCATTCATTGTACCAGTGGCCAGAGCCTAAAACCTTTGAACCGCTAGAGAAAAGCCTGTG 500590

||||||||||||||||||||||||||||||||||||||||||||||||||||||||||||

Sbjct 211188 TCATTCATTGTACCAGTGGCCAGAGCCTAAAACCTTTGAACCGCTAGAGAAAAGCCTGTG 211247

Query 500591 TACAGCAGAAACACTCGAATTTTTCGTGCAAGAGTTTGCTGGAGCTGTTTCAATCCGGGT 500650

||||||||||||||||||||||||||||||||||||||||||||||||||||||||||||

Sbjct 211248 TACAGCAGAAACACTCGAATTTTTCGTGCAAGAGTTTGCTGGAGCTGTTTCAATCCGGGT 211307

Query 500651 CACTTTAGCTTGTGGGGTCCTTCGAGTTAGCAATTAAAAGATGTAACGCAGAAAGGTGCA 500710

||||||||||||||||||||||||||||||||||||||||||||||||||||||||||||

Sbjct 211308 CACTTTAGCTTGTGGGGTCCTTCGAGTTAGCAATTAAAAGATGTAACGCAGAAAGGTGCA 211367

Query 500711 ATCCTCTTTCAATTTCGTTCAAGAAAACCCCTCCTTAAATCGAGTATTATGCATGGGATT 500770

||||||||||||||||||||||||||||||||||||||||||||||||||||||||||||

Sbjct 211368 ATCCTCTTTCAATTTCGTTCAAGAAAACCCCTCCTTAAATCGAGTATTATGCATGGGATT 211427

Query 500771 ATAGGAATCCAACCTaaaaaaaaGGAACTGGTACTCTGAGAATGAGAAAGCATGGATTCA 500830

||||||||||||||||||||||||||||||||||||||||||||||||||||||||||||

Sbjct 211428 ATAGGAATCCAACCTAAAAAAAAGGAACTGGTACTCTGAGAATGAGAAAGCATGGATTCA 211487

Query 500831 ATCCGCCACATCCACCACACACACCGCCCCCCATTCTTCTAGTTTAGAGCGAAGTGTTCT 500890

||||||||||||||||||||||||||||||||||||||||||||||||||||||||||||

Sbjct 211488 ATCCGCCACATCCACCACACACACCGCCCCCCATTCTTCTAGTTTAGAGCGAAGTGTTCT 211547

Query 500891 CAGTTAGGACATCCTACTCTTTATAATTAAGGAAAGGAAGTTCGTTCTCTTTTAGAGGGC 500950

||||||||||||||||||||||||||||||||||||||||||||||||||||||||||||

Sbjct 211548 CAGTTAGGACATCCTACTCTTTATAATTAAGGAAAGGAAGTTCGTTCTCTTTTAGAGGGC 211607

Query 500951 CGGGCTCTTGAAGTAAGTAAGTGATTCTACTTGAAAAAGACTTGCTTTATTTTCTTTCTG 501010

||||||||||||||||||||||||||||||||||||||||||||||||||||||||||||

Sbjct 211608 CGGGCTCTTGAAGTAAGTAAGTGATTCTACTTGAAAAAGACTTGCTTTATTTTCTTTCTG 211667

Query 501011 CTTGCTTAAACAAAGATCTTTCTTCCTTAACAAGAGACTCTCTTTTTGCAAATGGAGCAA 501070

||||||||||||||||||||||||||||||||||||||||||||||||||||||||||||

Sbjct 211668 CTTGCTTAAACAAAGATCTTTCTTCCTTAACAAGAGACTCTCTTTTTGCAAATGGAGCAA 211727

Query 501071 TAGCTTACAGAGTTTCAGACCTGAGAAGATGCTAGACCCATGCAACTGACGGAAAACTGT 501130

||||||||||||||||||||||||||||||||||||||||||||||||||||||||||||

Sbjct 211728 TAGCTTACAGAGTTTCAGACCTGAGAAGATGCTAGACCCATGCAACTGACGGAAAACTGT 211787

Query 501131 ATTGCTCTCTCACACCACAACGCGCCGAGTTACTACTCATGAAAAAGCAGCTAGACGACC 501190

||||||||||||||||||||||||||||||||||||||||||||||||||||||||||||

Sbjct 211788 ATTGCTCTCTCACACCACAACGCGCCGAGTTACTACTCATGAAAAAGCAGCTAGACGACC 211847

Query 501191 GACCAGAGGCCTTACGCTTTGTGGCCGCCTTGAAGCAAGCAGCTGAGTAGTCAAATCAAT 501250

||||||||||||||||||||||||||||||||||||||||||||||||||||||||||||

Sbjct 211848 GACCAGAGGCCTTACGCTTTGTGGCCGCCTTGAAGCAAGCAGCTGAGTAGTCAAATCAAT 211907

Query 501251 CGGACTGACTAGGTTTGTCTCATTGTTGTAAAGAAAGGGCAAACACTCCCAACCAAAGGA 501310

||||||||||||||||||||||||||||||||||||||||||||||||||||||||||||

Sbjct 211908 CGGACTGACTAGGTTTGTCTCATTGTTGTAAAGAAAGGGCAAACACTCCCAACCAAAGGA 211967

Query 501311 ATGTAATGAAATACTCCCAAGATAAAATACTCGCGGGCATAATAACTATTTATTTAGACT 501370

||||||||||||||||||||||||||||||||||||||||||||||||||||||||||||

Sbjct 211968 ATGTAATGAAATACTCCCAAGATAAAATACTCGCGGGCATAATAACTATTTATTTAGACT 212027

Query 501371 GCAAGAACCCGGGGGCAATAACTATTTATTTCCAAATGCATAAGACTGGCGAAGACTACT 501430

||||||||||||||||||||||| ||||||||||||||||||||||||||||||||||||

Sbjct 212028 GCAAGAACCCGGGGGCAATAACTTTTTATTTCCAAATGCATAAGACTGGCGAAGACTACT 212087

Query 501431 TATTTATGCACAGTAAGCTTAATAAGACTTATGAATTGACCTAGGGTAAGCAGCTTTCTT 501490

||||||||||||||||||||||||||||||||||||||||||||||||||||||||||||

Sbjct 212088 TATTTATGCACAGTAAGCTTAATAAGACTTATGAATTGACCTAGGGTAAGCAGCTTTCTT 212147

Query 501491 TGCAGTTTCTTCAGTTGGTATTGAAGGATGGAGGAAGCTAGGCAGACTTCCTACTCGAGT 501550

||||||||||||||||||||||||||||||||||||||||||||||||||||||||||||

Sbjct 212148 TGCAGTTTCTTCAGTTGGTATTGAAGGATGGAGGAAGCTAGGCAGACTTCCTACTCGAGT 212207

Query 501551 ACGCAGCTTTGTGTTCTTAAGTGAAAGCTTAAGCAGGGTAGGAATATTTCTAGTATGGCT 501610

||||||||||||||||||||||||||||||||||||||||||||||||||||||||||||

Sbjct 212208 ACGCAGCTTTGTGTTCTTAAGTGAAAGCTTAAGCAGGGTAGGAATATTTCTAGTATGGCT 212267

Query 501611 CCGCTCCAAAGCATATTCTCTTTCCACTAATCTGGCGCATTGCACTCCTTCCTTTCACTT 501670

||||||||||||||||||||||||||||||||||||||||||||||||||||||||||||

Sbjct 212268 CCGCTCCAAAGCATATTCTCTTTCCACTAATCTGGCGCATTGCACTCCTTCCTTTCACTT 212327

Query 501671 CCGTTCGTTTCACTTCCTAAGCATTGGCTGTAGTGAGCTCATCTAAGAATATATCATCAG 501730

||||||||||||||||||||||||||||||||||||||||||||||||||||||||||||

Sbjct 212328 CCGTTCGTTTCACTTCCTAAGCATTGGCTGTAGTGAGCTCATCTAAGAATATATCATCAG 212387

Query 501731 ATGGAGAAAGAAAAGCAGTGTTGTAACAAAACCTTTTGAAGCTCAATCCAATATTGGGTG 501790

||||||||||||||||||||||||||||||||||||||||||||||||||||||||||||

Sbjct 212388 ATGGAGAAAGAAAAGCAGTGTTGTAACAAAACCTTTTGAAGCTCAATCCAATATTGGGTG 212447

Query 501791 AGTCTAGCAGTTCAACCCTTTTGAAAGGGCATCCACACAGTATTGTTGACCCAGGCTTCA 501850

||||||||||||||||||||||||||||||||||||||||||||||||||||||||||||

Sbjct 212448 AGTCTAGCAGTTCAACCCTTTTGAAAGGGCATCCACACAGTATTGTTGACCCAGGCTTCA 212507

Query 501851 TGTACCAGCATATTAGGTGTAGTCAAATTAATGAGCAGAAAGCCTAGGATACCAAGTAGG 501910

||||||||||||||||||||||||||||||||||||||||||||||||||||||||||||

Sbjct 212508 TGTACCAGCATATTAGGTGTAGTCAAATTAATGAGCAGAAAGCCTAGGATACCAAGTAGG 212567

Query 501911 GTACATTCACCAATCCATCTATCTTGCAACAATCTAGTCATTTTGGAATTCCCTAAAGTC 501970

||||||||||||||||||||||||||||||||||||||||||||||||||||||||||||

Sbjct 212568 GTACATTCACCAATCCATCTATCTTGCAACAATCTAGTCATTTTGGAATTCCCTAAAGTC 212627

Query 501971 CAAGTTGCAACCTGGGGCTAATTCCTAAAATAACCGGAAGACATTTTCCTACAACAGGTA 502030

||||||||||||||||||||||||||||||||||||||||||||||||||||||||||||

Sbjct 212628 CAAGTTGCAACCTGGGGCTAATTCCTAAAATAACCGGAAGACATTTTCCTACAACAGGTA 212687

Query 502031 TTAGACTTATACTTGCAATTAATAAGATCTTTCCTACTACTATTATTATATCTAGGGGGG 502090

||||||||||||||||||||||||||||||||||||||||||||||||||||||||||||

Sbjct 212688 TTAGACTTATACTTGCAATTAATAAGATCTTTCCTACTACTATTATTATATCTAGGGGGG 212747

Query 502091 ATCTCTCTATTTCAACCACCATTTTGACCTCATGAATTAGGGAAATGAAAAGTACCCACG 502150

||||||||||||||||||||||||||||||||||||||||||||||||||||||||||||

Sbjct 212748 ATCTCTCTATTTCAACCACCATTTTGACCTCATGAATTAGGGAAATGAAAAGTACCCACG 212807

Query 502151 TTTTTTCTTCTTACAAAAAGTTTTCCAATTTAAAAATGCATACTCCCTTATTTCCCCTTT 502210

||||||||||||||||||||||||||||||| ||||||||||||||||||||||||||||

Sbjct 212808 TTTTTTCTTCTTACAAAAAGTTTTCCAATTTCAAAATGCATACTCCCTTATTTCCCCTTT 212867

Query 502211 TTGCATATGCATGAAATTGTTGAAAAGGTGGTGCTTGGTAGGTGATAATTAAGAGTGTTT 502270

||||||||||||||||||||||||||||||||||||||||||||||||||||||||||||

Sbjct 212868 TTGCATATGCATGAAATTGTTGAAAAGGTGGTGCTTGGTAGGTGATAATTAAGAGTGTTT 212927

Query 502271 TTGGAGTAGGACTTAAAATGATATAAAAAAGAGTACTATTTATGTTTTTCAGTGCTGAGA 502330

||||||||||||||||||||||||||||||||||||||||| ||||||||||||||||||

Sbjct 212928 TTGGAGTAGGACTTAAAATGATATAAAAAAGAGTACTATTTCTGTTTTTCAGTGCTGAGA 212987

Query 502331 ATCAGAAGAATGCCTATTGTACGGGTGGTCAGACATAGATATTGAAATAAAAACGTGCTT 502390

||||||||||||||||||||||||||||||||||||||||||||||||||||||||||||

Sbjct 212988 ATCAGAAGAATGCCTATTGTACGGGTGGTCAGACATAGATATTGAAATAAAAACGTGCTT 213047

Query 502391 AATGCCTGCCTAGAATGCTATTTGTTGACTTTACACCAAAATGCATAGTACTTTATTTAA 502450

||||||||||||||||||||||||||||||||||||||||||||||||||||||||||||

Sbjct 213048 AATGCCTGCCTAGAATGCTATTTGTTGACTTTACACCAAAATGCATAGTACTTTATTTAA 213107

Query 502451 TGGTAAGATATTTTGACTAGTATAGAATAGAAGACCTGTTTGACCAACGGTGCCTGCTGG 502510

||||||||||||||||||||||||||||||||||||||||||||||||||||||||||||

Sbjct 213108 TGGTAAGATATTTTGACTAGTATAGAATAGAAGACCTGTTTGACCAACGGTGCCTGCTGG 213167

Query 502511 TCTTTTGATTATAGGCCCCCCAGCCTGTGGCAAGGAGAAGGTTATCTCTCTGGCGCTGCT 502570

||||||||||||||||||||||||||||||||||||||||||||||||||||||||||||

Sbjct 213168 TCTTTTGATTATAGGCCCCCCAGCCTGTGGCAAGGAGAAGGTTATCTCTCTGGCGCTGCT 213227

Query 502571 CCGAGCAAGCCTTGCCCTTCCTATAGGCTAGAGGAGTACCACAGGGATTTTTCCTAGTTC 502630

||||||||||||||||||||||||||||||||||||||||||||||||||||||||||||

Sbjct 213228 CCGAGCAAGCCTTGCCCTTCCTATAGGCTAGAGGAGTACCACAGGGATTTTTCCTAGTTC 213287

Query 502631 TTCACTACTCTTAATCGACGGGTAGTTTATTTCCGTCCTATTGATTCTATGTAGTTTGAG 502690

||||||||||||||||||||||||||||||||||||||||||||||||||||||||||||

Sbjct 213288 TTCACTACTCTTAATCGACGGGTAGTTTATTTCCGTCCTATTGATTCTATGTAGTTTGAG 213347

Query 502691 TTTTCCTCCGAGTCGAAGTCATCCCCCTTATCCTACCCCATGTTAAGTCAAGATTGTATT 502750

||||||||||||||||||||||||||||||||||||||||||||||||||||| ||||||

Sbjct 213348 TTTTCCTCCGAGTCGAAGTCATCCCCCTTATCCTACCCCATGTTAAGTCAAGACTGTATT 213407

Query 502751 CCTATCTCAACTTCTTCAATTCATATCAACTAGAAAAACCAGATAGTGATCCGTTATAAT 502810

||||||||||||||||||||||||||||||||||||||||||||||||||||||||||||

Sbjct 213408 CCTATCTCAACTTCTTCAATTCATATCAACTAGAAAAACCAGATAGTGATCCGTTATAAT 213467

Query 502811 TTTGTCAAGCCTTGGTAGATGTATCCCATCATTCCGTGTCAAGCAAGAGTAATATATTGC 502870

||||||||||||||||||||||||||||||||||||||||||||||||||||||||||||

Sbjct 213468 TTTGTCAAGCCTTGGTAGATGTATCCCATCATTCCGTGTCAAGCAAGAGTAATATATTGC 213527

Query 502871 AATTAACCCATGTGTTAAGCATaaaaaaaTTGGATACTCAGGTTAGAGCTTTGAGTATGA 502930

||||||||||||||||||||||||||||||||||||||||||||||||||||||||||||

Sbjct 213528 AATTAACCCATGTGTTAAGCATAAAAAAATTGGATACTCAGGTTAGAGCTTTGAGTATGA 213587

Query 502931 ACTGCGAGGGCCCAACTGAGTTCATTTAGTAATTAAGTTGCACTATGTGTGTAAAGTTCT 502990

|||||||||||||||||||||||||||||||||||||||||||||||||||| |||||||

Sbjct 213588 ACTGCGAGGGCCCAACTGAGTTCATTTAGTAATTAAGTTGCACTATGTGTGTCAAGTTCT 213647

Query 502991 CAGTCTTACCGTCCCAAGTATCATTCCCGTTGTTTGAGGTTATTTTCCTTTCTAAAGCGG 503050

||||||||||||||||||||||||||||||||||||||||||||||||||||||||||||

Sbjct 213648 CAGTCTTACCGTCCCAAGTATCATTCCCGTTGTTTGAGGTTATTTTCCTTTCTAAAGCGG 213707

Query 503051 TACTAGTAATGGAGGCGAGTAATTGCATGGTCGTGGAATCATATTCTTATTCGAATGTGG 503110

||||||||||||||||||||||||||||||||||||||||||||||||||||||||||||

Sbjct 213708 TACTAGTAATGGAGGCGAGTAATTGCATGGTCGTGGAATCATATTCTTATTCGAATGTGG 213767

Query 503111 TGAGTAAGTCCCTCGCTATATCTATTGTCAAGTACTCCATCTAAGTAGTCTAAAGTAGGA 503170

||||||||||||||||||||||||||||||||||||||||||||||||||||||||||||

Sbjct 213768 TGAGTAAGTCCCTCGCTATATCTATTGTCAAGTACTCCATCTAAGTAGTCTAAAGTAGGA 213827

Query 503171 ATTTAGTAGTCTAAAGTGATATTTACTGGATGAGTCATATGTGTTCCCTAGTCATATGTC 503230

||||||||||||||||||||||||||||||||||||||||||||||||||||||||||||

Sbjct 213828 ATTTAGTAGTCTAAAGTGATATTTACTGGATGAGTCATATGTGTTCCCTAGTCATATGTC 213887

Query 503231 GGATTGCTATATATGATCAAAAAGAAGGTAGGCATTGAGTTTGTGTGTCAATGTGTAATG 503290

||||||||||||||||||||||||||||||||||||||||||||||||||||||||||||

Sbjct 213888 GGATTGCTATATATGATCAAAAAGAAGGTAGGCATTGAGTTTGTGTGTCAATGTGTAATG 213947

Query 503291 ATAAGAAGAAATTCAAAGTAATCAGTTGAGTATGGGCCCTGTGTATAGGAATGTATATGG 503350

||||||||||||||||||||||||||||||||||||||||||||||||||||||||||||

Sbjct 213948 ATAAGAAGAAATTCAAAGTAATCAGTTGAGTATGGGCCCTGTGTATAGGAATGTATATGG 214007

Query 503351 TCTGTGAAGTTTTGTTTGGGTATCATAGTAGTTCCGTGATTTGTTTTGTGAGTAGTCTTT 503410

||||||||||||||||||||||||||||||||||||||||||||||||||||||||||||

Sbjct 214008 TCTGTGAAGTTTTGTTTGGGTATCATAGTAGTTCCGTGATTTGTTTTGTGAGTAGTCTTT 214067

Query 503411 AAGTTGAGTGCACTCGCTGTAGTAAGTAAGAGGGTAGTAGGGGTATAGCAGTTTCAAGGT 503470

||||||||||||||||||||||||||||||||||||||||||||||||||||||||||||

Sbjct 214068 AAGTTGAGTGCACTCGCTGTAGTAAGTAAGAGGGTAGTAGGGGTATAGCAGTTTCAAGGT 214127

Query 503471 GGAGTGGGCTTAAGGGTATAGGTAAGTAAAGAGTATAGTAATTCAAAGGTGAAGTTTGTG 503530

||||||||||||||||||||||||||||||||||||||||||||||||||||||||||||

Sbjct 214128 GGAGTGGGCTTAAGGGTATAGGTAAGTAAAGAGTATAGTAATTCAAAGGTGAAGTTTGTG 214187

Query 503531 TAAAGAGGAGTATCTATTTGTTCATAAAAAACAAGGTATATGTTATCCTTGGTAGGAGTA 503590

||||||||||||||||||||||||||||||||||||||||||||||||||||||||||||

Sbjct 214188 TAAAGAGGAGTATCTATTTGTTCATAAAAAACAAGGTATATGTTATCCTTGGTAGGAGTA 214247

Query 503591 TTTTCAGTGTTCATGGTGAGATAGGTAGGGCATAAAGTTGGTAGTCAGGAATGAGGACAG 503650

||||||||||||||||||||||||||||||||||||||||||||||||||||||||||||

Sbjct 214248 TTTTCAGTGTTCATGGTGAGATAGGTAGGGCATAAAGTTGGTAGTCAGGAATGAGGACAG 214307

Query 503651 TAAGGGATGTCAAGGCTGGGTACTTTCTGTTTGTCCCCAAAGGGTGTATAGGAAAAGAAG 503710

||||||||||||||||||||||||||||||||||||||||||||||||||||||||||||

Sbjct 214308 TAAGGGATGTCAAGGCTGGGTACTTTCTGTTTGTCCCCAAAGGGTGTATAGGAAAAGAAG 214367

Query 503711 CATTAGAATTACTATTAATACGAAGAAACCAAAGGCAGTATACACTCTCTATTGATTTGA 503770

||||||||||||||||||||||||||||||||||||||||||||||||||||||||||||

Sbjct 214368 CATTAGAATTACTATTAATACGAAGAAACCAAAGGCAGTATACACTCTCTATTGATTTGA 214427

Query 503771 TCATTTACTTACAGCTCTTGAAGACTCCTTCGTTTAAAACAGGCTTTTGCGATTAAGGGG 503830

||||||||||||||||||||||||||||||||||||||||||||||||||||||||||||

Sbjct 214428 TCATTTACTTACAGCTCTTGAAGACTCCTTCGTTTAAAACAGGCTTTTGCGATTAAGGGG 214487

Query 503831 GTTTCCTTTTTTCAAGTATGTTCTCCATTCAGTTGAGCCACTATAAGTGTTTCAGGTGTC 503890

||||||||||||||||||||||||||||||||||||||||||||||||||||||||||||

Sbjct 214488 GTTTCCTTTTTTCAAGTATGTTCTCCATTCAGTTGAGCCACTATAAGTGTTTCAGGTGTC 214547

Query 503891 TTACTTATAGTATTGAGGAAGGAGTTCAATCTGAGAAGCCAAGTCTATTCAAGGTCTAAC 503950

||||||||||||||||||||||||||||||||||||||||||||||||||||||||||||

Sbjct 214548 TTACTTATAGTATTGAGGAAGGAGTTCAATCTGAGAAGCCAAGTCTATTCAAGGTCTAAC 214607

Query 503951 TACCACTGATATCCCGACCCTTTAGATAATAGTATAAATCCCAATAGTTCAAAGGGTACT 504010

||||||||||||||||||||||||||||||||||||||||||||||||||||||||||||

Sbjct 214608 TACCACTGATATCCCGACCCTTTAGATAATAGTATAAATCCCAATAGTTCAAAGGGTACT 214667

Query 504011 CACTAAGTAAGGTTGGAAGGAATGTGAGACTCATTCATTCCTATTTACCTAAGAGCCTAA 504070

|||||||||||||||||||||||||||||||||||||||||||||||||||||||||||

Sbjct 214668 TACTAAGTAAGGTTGGAAGGAATGTGAGACTCATTCATTCCTATTTACCTAAGAGCCTAA 214727

Query 504071 GCAATAAATATCAAGCCATTGTAGTATTTCATCGTTAGACCTCGATGTTAATTCCTTAGT 504130

||||||||||||||||||||||||||||||||||||||||||||||||||||||||||||

Sbjct 214728 GCAATAAATATCAAGCCATTGTAGTATTTCATCGTTAGACCTCGATGTTAATTCCTTAGT 214787

Query 504131 CCCACAACTACTTAATGACCTTATCTACCTTATGAACTTATCTACCTTATGAACTTATTT 504190

||||||||||||||||||||||||||||||||||||||||||||||||||||||||||||

Sbjct 214788 CCCACAACTACTTAATGACCTTATCTACCTTATGAACTTATCTACCTTATGAACTTATTT 214847

Query 504191 ACCTTATGTATGTACAGCAATAAAAGGATAGGCCTTGCTTTCTTTAGCCCACCGGCTTTC 504250

||||||||||||||||||||||||||||||||||||||||||||||||||||||||||||

Sbjct 214848 ACCTTATGTATGTACAGCAATAAAAGGATAGGCCTTGCTTTCTTTAGCCCACCGGCTTTC 214907

Query 504251 TACTGCCGGTACTTCTTGCTTTCTACTGCCGGTGCTTCTTGACTGTAGCTTTCTTTTAAT 504310

||||||||||||||||||||||||||||||||||||||||||||||||||||||||||||

Sbjct 214908 TACTGCCGGTACTTCTTGCTTTCTACTGCCGGTGCTTCTTGACTGTAGCTTTCTTTTAAT 214967

Query 504311 CTATTCTTCGACAAGCCTAGCGATATGACCATTCTATAGCTACCCCGGTGAAAATGAATT 504370

||||||||||||||||||||||||||||||||||||||||||||||||||||||||||||

Sbjct 214968 CTATTCTTCGACAAGCCTAGCGATATGACCATTCTATAGCTACCCCGGTGAAAATGAATT 215027

Query 504371 TGCTCCGTCGGTGGCAACTATTAAGTATATCTTTTCTTCGCTCTGCTCTTCTCGTTCATG 504430

||||||||||||||||||||||||||||||||||||||||||||||||||||||||||||

Sbjct 215028 TGCTCCGTCGGTGGCAACTATTAAGTATATCTTTTCTTCGCTCTGCTCTTCTCGTTCATG 215087

Query 504431 TTTCATGTGCAGTTCGAGTTCTTGGCCCAAGGAAAGAGGATCGATTCAAGTAAGTCAAGT 504490

||||||||||||||||||||||||||||||||||||||||||||||||||||||||||||

Sbjct 215088 TTTCATGTGCAGTTCGAGTTCTTGGCCCAAGGAAAGAGGATCGATTCAAGTAAGTCAAGT 215147

Query 504491 ACAAGTCAAAGCCCAATTGCAGCTATATATAAGCTCAGAATATGTAGGCGCCTCTCATAA 504550

||||||||||||||||||||||||||||||||||||||||||||||||||||||||||||

Sbjct 215148 ACAAGTCAAAGCCCAATTGCAGCTATATATAAGCTCAGAATATGTAGGCGCCTCTCATAA 215207

Query 504551 CTGAACTCAACCAGCATTCCAGCTAGCAAGCCTATGAATGCTATATTCTCGTAGTTCATG 504610

||||||||||||||||||||||||||||||||||||||||||||||||||||||||||||

Sbjct 215208 CTGAACTCAACCAGCATTCCAGCTAGCAAGCCTATGAATGCTATATTCTCGTAGTTCATG 215267

Query 504611 TGCATTTTTCTATGGAGTAGCCAGTGCGCACCCCAGAAAAGCTTAGGATGTGCTCGCTTG 504670

||||||||||||||||||||||||||||||||||||||||||||||||||||||||||||

Sbjct 215268 TGCATTTTTCTATGGAGTAGCCAGTGCGCACCCCAGAAAAGCTTAGGATGTGCTCGCTTG 215327

Query 504671 CTATTGACTAGGAACCGATGAATAGAAAAACCTACTGGAAAAGGTAGCTCCCTGTCTATT 504730

||||||||||||||||||||||||||||||||||||||||||||||||||||||||||||

Sbjct 215328 CTATTGACTAGGAACCGATGAATAGAAAAACCTACTGGAAAAGGTAGCTCCCTGTCTATT 215387

Query 504731 CCTCTACCGCATCTGATCCTTTACTAGCAGACGCAAACTCCATTATGAAGGCAGCACCAA 504790

||||||||||||||||||||||||||||||||||||||||||||||||||||||||||||

Sbjct 215388 CCTCTACCGCATCTGATCCTTTACTAGCAGACGCAAACTCCATTATGAAGGCAGCACCAA 215447

Query 504791 TGAATTTCATCAATCAAATAGTCAAGACTAACTAACTGCTTTTAAGAAGTACGCTACTTA 504850

||||||||||||||||||||||||||||||||||||||||||||||||||||||||||||

Sbjct 215448 TGAATTTCATCAATCAAATAGTCAAGACTAACTAACTGCTTTTAAGAAGTACGCTACTTA 215507

Query 504851 CTAAAAGCTTTGAAGAAGTACGCTGAAGATAGCACTGATGGGGGTTGAGCTCTCTGAGAG 504910

||||||||||||||||||||||||||||||||||||||||||||||||||||||||||||

Sbjct 215508 CTAAAAGCTTTGAAGAAGTACGCTGAAGATAGCACTGATGGGGGTTGAGCTCTCTGAGAG 215567

Query 504911 TGGAAGGCTCGTGATCTGCTTACACGTCCTGGTTTTATTCGTTCTCCGGTAAGGGACAAC 504970

||||||||||||||||||||||||||||||||||||||||||||||||||||||||||||

Sbjct 215568 TGGAAGGCTCGTGATCTGCTTACACGTCCTGGTTTTATTCGTTCTCCGGTAAGGGACAAC 215627

Query 504971 CAATATGATTATCTTCATGAACAATATCTCATAGATAGGACAATGAACAATTTCTCAAGG 505030

||||||||||||||||||||||||||||||||||||||||||||||||||||||||||||

Sbjct 215628 CAATATGATTATCTTCATGAACAATATCTCATAGATAGGACAATGAACAATTTCTCAAGG 215687

Query 505031 ACAGAAGTGCAATTCCTTTCCTTGAACAGAAGTGGTGCAAATCCTTCTGGGCAAGAGATC 505090

||||||||||||||||||||||||||||||||||||||||||||||||||||||||||||

Sbjct 215688 ACAGAAGTGCAATTCCTTTCCTTGAACAGAAGTGGTGCAAATCCTTCTGGGCAAGAGATC 215747

Query 505091 TTTCATTCCTGATAGCTAGCGCGTTGCGTTGAAAGACGAGCTTGCAGAGTAGCAGCGTCC 505150

||||||||||||||||||||||||||||||||||||||||||||||||||||||||||||

Sbjct 215748 TTTCATTCCTGATAGCTAGCGCGTTGCGTTGAAAGACGAGCTTGCAGAGTAGCAGCGTCC 215807

Query 505151 TTCAGAGTCAGACCTTAATTCCTAGTGGAACACTTTGACCTAGCACCGTTTCCTTTGGTT 505210

||||||||||||||||||||||||||||||||||||||||||||||||||||||||||||

Sbjct 215808 TTCAGAGTCAGACCTTAATTCCTAGTGGAACACTTTGACCTAGCACCGTTTCCTTTGGTT 215867

Query 505211 GTAGCACTTTTCCTAACAGATAAGGATAAAGCTCTGAAAGTCAGGGTAAAGCTCTGAAAG 505270

||||||||||||||||||||||||||||||||||||||||||||||||||||||||||||

Sbjct 215868 GTAGCACTTTTCCTAACAGATAAGGATAAAGCTCTGAAAGTCAGGGTAAAGCTCTGAAAG 215927

Query 505271 CTCATACTAGTGGAAGCGAGAATATCATGTGATTTGAAAGTAAAGCTTCATTTCTCCAGG 505330

||||||||||||||||||||||||||||||||||||||||||||||||||||||||||||

Sbjct 215928 CTCATACTAGTGGAAGCGAGAATATCATGTGATTTGAAAGTAAAGCTTCATTTCTCCAGG 215987

Query 505331 AAAAATACAGAACTTCAGTGTTGAAGCTTACTGAGTTGACACAAAGGACACTGACAACAT 505390

||||||||||||||||||||||||||||||||||||||||||||||||||||||||||||

Sbjct 215988 AAAAATACAGAACTTCAGTGTTGAAGCTTACTGAGTTGACACAAAGGACACTGACAACAT 216047

Query 505391 ACATGTTTGAAGCATATAACTCTATACAGGCATATAATTATCTACATATAAATAACTATG 505450

||||||||||||||||||||||||||||||||||||||||||||||||||||||||||||

Sbjct 216048 ACATGTTTGAAGCATATAACTCTATACAGGCATATAATTATCTACATATAAATAACTATG 216107

Query 505451 CTAGCAAAAAGTCTAGGAATATCTTTCTAGCTCTAGTCAAGtttttttCGTCATAGACTC 505510

||||||||||||||||||||||||||||||||||||||||||||||||||||||||||||

Sbjct 216108 CTAGCAAAAAGTCTAGGAATATCTTTCTAGCTCTAGTCAAGTTTTTTTCGTCATAGACTC 216167

Query 505511 GCCTTGAAGTACTTCTTGGCAGGCTGCCTGCTTCACTGACATCTCGTTAGGCTAGGATTG 505570

||||||||||||||||||||||||||||||||||||||||||||||||||||||||||||

Sbjct 216168 GCCTTGAAGTACTTCTTGGCAGGCTGCCTGCTTCACTGACATCTCGTTAGGCTAGGATTG 216227

Query 505571 GTACATATTCAAATAAGGCAACAAGAGCTTCTATCGGTTCTACTTTCTTCTTCCTTCTTA 505630

||||||||||||||||||||||||||||||||||||||||||||||||||||||||||||

Sbjct 216228 GTACATATTCAAATAAGGCAACAAGAGCTTCTATCGGTTCTACTTTCTTCTTCCTTCTTA 216287

Query 505631 AAGATGTTCATCATGGGAAAGGAAATGTTGTTGTTTAGCAGGCTGATCCCTCCTAGTCCG 505690

||||||||||||||||||||||||||||||||||||||||||||||||||||||||||||

Sbjct 216288 AAGATGTTCATCATGGGAAAGGAAATGTTGTTGTTTAGCAGGCTGATCCCTCCTAGTCCG 216347

Query 505691 GTACGTGGTATAACTTATCCCAGTTCAAGCCAATTAGTTTATGCAATGTGGCGTACAAGA 505750

||||||||||||||||||||||||||||||||||||||||||||||||||||||||||||

Sbjct 216348 GTACGTGGTATAACTTATCCCAGTTCAAGCCAATTAGTTTATGCAATGTGGCGTACAAGA 216407

Query 505751 TAGTGGCTGGCAAAGGTGTGGGCTGAAACAGATTCTTCCTTACTGACCAGAGCTTAGTGC 505810

||||||||||||||||||||||||||||||||||||||||||||||||||||||||||||

Sbjct 216408 TAGTGGCTGGCAAAGGTGTGGGCTGAAACAGATTCTTCCTTACTGACCAGAGCTTAGTGC 216467

Query 505811 ATTTGTACCCAACCGAAAATGTGATGACAAATGATGATTAAGACGAGATTCGGCATTCTT 505870

||||||||||||||||||||||||||||||||||||||||||||||||||||||||||||

Sbjct 216468 ATTTGTACCCAACCGAAAATGTGATGACAAATGATGATTAAGACGAGATTCGGCATTCTT 216527

Query 505871 GAACCACGAAAGGCTTGGTTTCCATTTGGGTTGTAGATGTAACCAACCTGCAACTAAGGA 505930

||||||||||||||||||||||||||||||||||||||||||||||||||||||||||||

Sbjct 216528 GAACCACGAAAGGCTTGGTTTCCATTTGGGTTGTAGATGTAACCAACCTGCAACTAAGGA 216587

Query 505931 TATTGCAGAAAGAAGTAATAGAAAAAGAGCTCCAGTATAAAGATCCTCATTGCTACGTAA 505990

||||||||||||||||||||||||||||||||||||||||||||||||||||||||||||

Sbjct 216588 TATTGCAGAAAGAAGTAATAGAAAAAGAGCTCCAGTATAAAGATCCTCATTGCTACGTAA 216647

Query 505991 TCCGATTGTATACCACCACTGATAAAGACCAGAATAAGCAATATTGACTGGACCAGTAGC 506050

||||||||||||||||||||||||||||||||||||||||||||||||||||||||||||

Sbjct 216648 TCCGATTGTATACCACCACTGATAAAGACCAGAATAAGCAATATTGACTGGACCAGTAGC 216707

Query 506051 ACCTCCTCGAGTAAAGGCTTCTACAGCTGGTTGGCCAAAATGCGGATCCCAAATTGCATG 506110

||||||||||||||||||||||||||||||||||||||||||||||||||||||||||||

Sbjct 216708 ACCTCCTCGAGTAAAGGCTTCTACAGCTGGTTGGCCAAAATGCGGATCCCAAATTGCATG 216767

Query 506111 AGCAATAGGTCTTATATGCAAAGGATCCTGTATCCATAACTCAAAATTTCCTTGCCAAGC 506170

||||||||||||||||||||||||||||||||||||||||||||||||||||||||||||

Sbjct 216768 AGCAATAGGTCTTATATGCAAAGGATCCTGTATCCATAACTCAAAATTTCCTTGCCAAGC 216827

Query 506171 TACATGAAACAGATTTCCAGACGTCCACAGAAATATTATTGCTAACTGACCAAAGTGAGA 506230

||||||||||||||||||||||||||||||||||||||||||||||||||||||||||||

Sbjct 216828 TACATGAAACAGATTTCCAGACGTCCACAGAAATATTATTGCTAACTGACCAAAGTGAGA 216887

Query 506231 AGCAAAAATGTTCTGATAAAGACGTTCTTCAGTAATATCATCATGAATCTCGAAGTCATG 506290

||||||||||||||||||||||||||||||||||||||||||||||||||||||||||||

Sbjct 216888 AGCAAAAATGTTCTGATAAAGACGTTCTTCAGTAATATCATCATGAATCTCGAAGTCATG 216947

Query 506291 TGCAGTAGCAATACCAAACCAAATACGACGAGTAGTGGGGTCCTGAGCTAAACCTTGGCT 506350

||||||||||||||||||||||||||||||||||||||||||||||||||||||||||||

Sbjct 216948 TGCAGTAGCAATACCAAACCAAATACGACGAGTAGTGGGGTCCTGAGCTAAACCTTGGCT 217007

Query 506351 AAACCTCGGAAATCTTAATGCCATAATGCTTTTCAAATCCTCCTAGCCATTATCCTACTG 506410

||||||||||||||||||||||||||||||||||||||||||||||||||||||||||||

Sbjct 217008 AAACCTCGGAAATCTTAATGCCATAATGCTTTTCAAATCCTCCTAGCCATTATCCTACTG 217067

Query 506411 CAATAATTCTTGCTAAGAAGAATGCCCATGTTGTGGCAATTCCACCCAGAAGGTAATGGG 506470

||||||||||||||||||||||||||||||||||||||||||||||||||||||||||||

Sbjct 217068 CAATAATTCTTGCTAAGAAGAATGCCCATGTTGTGGCAATTCCACCCAGAAGGTAATGGG 217127

Query 506471 TTACTCCTACAGCACGTCCTTGTATAATACTCAAAGCTCTAGGCTGAGTAGCAGGAGCAA 506530

||||||||||||||||||||||||||||||||||||||||||||||||||||||||||||

Sbjct 217128 TTACTCCTACAGCACGTCCTTGTATAATACTCAAAGCTCTAGGCTGAGTAGCAGGAGCAA 217187

Query 506531 CTTTTAATTTGTTATGAGCCCAAACAATGGATTCAATAAGTTCTTGCCAATAACCACGAC 506590

||||||||||||||||||||||||||||||||||||||||||||||||||||||||||||

Sbjct 217188 CTTTTAATTTGTTATGAGCCCAAACAATGGATTCAATAAGTTCTTGCCAATAACCACGAC 217247

Query 506591 CACTAAATAGAAACATTAAACTGAAAGCCCAAACAAAATGAGCACCTAAGAAGAAAAGAC 506650

||||||||||||||||||||||||||||||||||||||||||||||||||||||||||||

Sbjct 217248 CACTAAATAGAAACATTAAACTGAAAGCCCAAACAAAATGAGCACCTAAGAAGAAAAGAC 217307

Query 506651 CATATGCAGATAATGAAGAACCATAAGATTGAATTACTTGAGAAGCCTGTGCCCATAGGA 506710

||||||||||||||||||||||||||||||||||||||||||||||||||||||||||||

Sbjct 217308 CATATGCAGATAATGAAGAACCATAAGATTGAATTACTTGAGAAGCCTGTGCCCATAGGA 217367

Query 506711 AATCTCTTAACCACCCATTAATCGTAATGGAACTTTGTGCAAAATTTCCTCCTGTGATAT 506770

||||||||||||||||||||||||||||||||||||||||||||||||||||||||||||

Sbjct 217368 AATCTCTTAACCACCCATTAATCGTAATGGAACTTTGTGCAAAATTTCCTCCTGTGATAT 217427

Query 506771 GAGTAATTATCCCTTGGTCGCTTACAGTACCCCAAACATCCGACTGCATTTTCCAACTGA 506830

|||||||||||||||||||||||| |||||||||||||||||||||||||||||||||||

Sbjct 217428 GAGTAATTATCCCTTGGTCGCTTATAGTACCCCAAACATCCGACTGCATTTTCCAACTGA 217487

Query 506831 AATGGAAAATTACTACCGAAATTGAATTGTACATCCAAAATAGACCTAAGAAAACATGAT 506890

||||||||||||||||||||||||||||||||||||||||||||||||||||||||||||

Sbjct 217488 AATGGAAAATTACTACCGAAATTGAATTGTACATCCAAAATAGACCTAAGAAAACATGAT 217547

Query 506891 CCCAAGCGGATACTTGACATGTTCCCCCTCGTCCAGGTCCATCACAAGGAAAACGAAAAC 506950

||||||||||||||||||||||||||||||||||||||||||||||||||||||||||||

Sbjct 217548 CCCAAGCGGATACTTGACATGTTCCCCCTCGTCCAGGTCCATCACAAGGAAAACGAAAAC 217607

Query 506951 CAAGATTTGCTTTATCAGGGATCAAACGGGAACTGCGAGCAAATAGAACACCCTTAAGTA 507010

||||||||||||||||||||||||||||||||||||||||||||||||||||||| ||||

Sbjct 217608 CAAGATTTGCTTTATCAGGGATCAAACGGGAACTGCGAGCAAATAGAACACCCTTCAGTA 217667

Query 507011 GTATCAATACAGTCACATGAATCGTAAATGCATGAATATGATGGACTAGAAAATCCGCGG 507070

||||||||||||||||||||||||||||||||||||||||||||||||||||||||||||

Sbjct 217668 GTATCAATACAGTCACATGAATCGTAAATGCATGAATATGATGGACTAGAAAATCCGCGG 217727

Query 507071 TTCCTAATGGAATAGGTAATAAAGCTATTTTGCTACCTACTGCTACTAATTCACTACCTC 507130

||||||||||||||||||||||||||||||||||||||||||||||||||||||||||||

Sbjct 217728 TTCCTAATGGAATAGGTAATAAAGCTATTTTGCTACCTACTGCTACTAATTCACTACCTC 217787

Query 507131 CCCAAGTTAAGCTGGTAGGCGTTGTTGCACCAGGAGCTGTTAGGCTAGGTGCTAAAGCAT 507190

|||||||||||||||||| |||||||||||||||||||||||||||||||||||||||||

Sbjct 217788 CCCAAGTTAAGCTGGTAGCCGTTGTTGCACCAGGAGCTGTTAGGCTAGGTGCTAAAGCAT 217847

Query 507191 GAGTATTTTGTATCCATTGAGCAAAGATGGGTTGTAATTGGATAGCGGTATCTGAAAACA 507250

||||||||||||||||||||||||||||||||||||||||||||||||||||||||||||

Sbjct 217848 GAGTATTTTGTATCCATTGAGCAAAGATGGGTTGTAATTGGATAGCGGTATCTGAAAACA 217907

Query 507251 TGTCTTGAGGACGTCCTAAAGCACTCATGGTATCATTATGGATATACAAGCCAAAACTGT 507310

||||||||||||||||||||||||||||||||||||||||||||||||||||||||||||

Sbjct 217908 TGTCTTGAGGACGTCCTAAAGCACTCATGGTATCATTATGGATATACAAGCCAAAACTGT 217967

Query 507311 GAAAACCTAAAAATATACATACCCAGTTAAGATGTGATATGATTGCATCGCGGTGTCTAA 507370

||||||||||||||||||||||||||||||||||||||||||||||||| ||||||||||

Sbjct 217968 GAAAACCTAAAAATATACATACCCAGTTAAGATGTGATATGATTGCATCACGGTGTCTAA 218027

Query 507371 GGATACGATCTAAAAGATCATTGTATCGAGTAGTTGGATCATAGTCTCTTACTAGAAAAA 507430

||||||||||||||||||||||||||||||||||||||||||||||||||||||||||||

Sbjct 218028 GGATACGATCTAAAAGATCATTGTATCGAGTAGTTGGATCATAGTCTCTTACTAGAAAAA 218087

Query 507431 TGGCTGCATGTGCAGCAGCACCAACTATCAGAAATCCACCAATCCACATGTGATGTGTGA 507490

||||||||||||||||||||||||||||||||||||||||||||||||||||||||||||

Sbjct 218088 TGGCTGCATGTGCAGCAGCACCAACTATCAGAAATCCACCAATCCACATGTGATGTGTGA 218147

Query 507491 ACAATGAAAGTTGTGTACCGTAGTCAGTAGCTAGATATGGATAAGGAGGCATTGAATACA 507550

||||||||||||||||||||||||||||||||||||||||||||||||||||||||||||

Sbjct 218148 ACAATGAAAGTTGTGTACCGTAGTCAGTAGCTAGATATGGATAAGGAGGCATTGAATACA 218207

Query 507551 TATGATGAGCTACAATAATGGTTAAGGAACCCAACATAGTTAGGTTAAGAGATAATTGAG 507610

||||||||||||||||||||||||||||||||||||||||||||||||||||||||||||

Sbjct 218208 TATGATGAGCTACAATAATGGTTAAGGAACCCAACATAGTTAGGTTAAGAGATAATTGAG 218267

Query 507611 CGTGCCATGAAGTTGTTAAAATTTCATAAAGACCCATATACCCATACCAGCCATGGCTAC 507670

||||||||||||||||||||||||||||||||||||||||||||||||||||||||||||

Sbjct 218268 CGTGCCATGAAGTTGTTAAAATTTCATAAAGACCCATATACCCATACCAGCCATGGCTAC 218327

Query 507671 CGACTCACCAGCCTCCTGGGCAAGATCCGGACCGAGTAACTTATTCTCACCTTTTTCCAC 507730

||||||||||||||||||||||||||||||||||||||||||||||||||||||||||||

Sbjct 218328 CGACTCACCAGCCTCCTGGGCAAGATCCGGACCGAGTAACTTATTCTCACCTTTTTCCAC 218387

Query 507731 CAACATCAGAAGAGGGAGATGCATCTTTCCTTGTTGCGGAATTTGCTTCATTGACTTGGA 507790

||||||||||||||||||||||||||||||||||||||||||||||||||||||||||||

Sbjct 218388 CAACATCAGAAGAGGGAGATGCATCTTTCCTTGTTGCGGAATTTGCTTCATTGACTTGGA 218447

Query 507791 CGTTATTCGATGCATCGGACTCAACTAGATAAGATAATTATTGCCTGAACCCTAGAAATG 507850

||||||||||||||||||||||||||||||||||||||||||||||||||||||||||||

Sbjct 218448 CGTTATTCGATGCATCGGACTCAACTAGATAAGATAATTATTGCCTGAACCCTAGAAATG 218507

Query 507851 CGGAAACTTCACCATAGGGAGGGAATTTGCAGATTCTTAATCTTTCCGATATCAAACGCT 507910

||||||||||||||||||||||||||||||||||||||||||||||||||||||||||||

Sbjct 218508 CGGAAACTTCACCATAGGGAGGGAATTTGCAGATTCTTAATCTTTCCGATATCAAACGCT 218567

Query 507911 TTGAAACGCATTTATGAAAGATAGACTGAAAGCCTACTCCACTGATACGGCAGGACTATC 507970

||||||||||||||||||||||||||||||||||||||||||||||||||||||||||||

Sbjct 218568 TTGAAACGCATTTATGAAAGATAGACTGAAAGCCTACTCCACTGATACGGCAGGACTATC 218627

Query 507971 AACCCTTAACCTTGCCCGCTGACTATTGACATATATTGGTGGATGCTTCGGAGTATAAGA 508030

||||||||||||||||||||||||||||||||||||||||||||||||||||||||||||

Sbjct 218628 AACCCTTAACCTTGCCCGCTGACTATTGACATATATTGGTGGATGCTTCGGAGTATAAGA 218687

Query 508031 ATGCATGCCAAACTCATTGACCAGAAGAGCTAAGAATGGAATTAGAATCTTAGGGATATG 508090

||||||||||||||||||||||||||||||||||||||||||||||||||||||||||||

Sbjct 218688 ATGCATGCCAAACTCATTGACCAGAAGAGCTAAGAATGGAATTAGAATCTTAGGGATATG 218747

Query 508091 CAATGAACTGTAATGACCTAGTATTGTGAACCTACAACTACTAGATCTTTTTCTAACGAC 508150

||||||||||||||||||||||||||||||||||||||||||||||||||||||||||||

Sbjct 218748 CAATGAACTGTAATGACCTAGTATTGTGAACCTACAACTACTAGATCTTTTTCTAACGAC 218807

Query 508151 GGAATATAGGATAGCAAATACAGGGTGGAACCCATAGGTATCAACTACTTATATCTTTAG 508210

||||||||||||||||||||||||||||||||||||||||||||||||||||||||||||

Sbjct 218808 GGAATATAGGATAGCAAATACAGGGTGGAACCCATAGGTATCAACTACTTATATCTTTAG 218867

Query 508211 CTTACTTGTTACTATATAtttttttttttCCTGAAATGGATTTATATGTTTTCCTACTGG 508270

|||||||||||||||||| |||||||||||||||||||||||||||||||||||||||||

Sbjct 218868 CTTACTTGTTACTATATA-TTTTTTTTTTCCTGAAATGGATTTATATGTTTTCCTACTGG 218926

Query 508271 CGACAAGACTTGAGCCAACTTCTCCAGACAATGAATAGATCGGACACTGTTGCTGTCTGC 508330

||||||||||||||||||||||||||||||||||||||||||||||||||||||||||||

Sbjct 218927 CGACAAGACTTGAGCCAACTTCTCCAGACAATGAATAGATCGGACACTGTTGCTGTCTGC 218986

Query 508331 GACTAAACTCCAACGTTTTCAGACACAGAGGTACTAGACTCATAACTCCACTAACTTGAC 508390

||||||||||||||||||||||||||||||||||||||||||||||||||||||||||||

Sbjct 218987 GACTAAACTCCAACGTTTTCAGACACAGAGGTACTAGACTCATAACTCCACTAACTTGAC 219046

Query 508391 CTATTCATTGAGGAAAGGAATTTACTCTAAGGCTTAGATAGAGAAAAGATGAAAAATTAG 508450

||||||||||||||||||||||||||||||||||||||||||||||||||||||||||||

Sbjct 219047 CTATTCATTGAGGAAAGGAATTTACTCTAAGGCTTAGATAGAGAAAAGATGAAAAATTAG 219106

Query 508451 GTGATAGTATTGACACAATAGAGTTTCTGAGGGCGTTCACCTTTGTCTTATGGTAAGTAG 508510

||||||||||||||||||||||||||||||||||||||||||||||||||||||||||||

Sbjct 219107 GTGATAGTATTGACACAATAGAGTTTCTGAGGGCGTTCACCTTTGTCTTATGGTAAGTAG 219166

Query 508511 AAAGCGGCGCAAAATCGTTCATTTATGTCTGAATGGAAAAGAATGCAAATTGAGATAATA 508570

||||||||||||||||||||||||||||||||||||||||||||||||||||||||||||

Sbjct 219167 AAAGCGGCGCAAAATCGTTCATTTATGTCTGAATGGAAAAGAATGCAAATTGAGATAATA 219226

Query 508571 TGTTAGTGAAATACACCAGTCTAAATAAGCGTAGCACTAATTTAATTGCCCGAGTTCGTG 508630

||||||||||||||||||||||||||||||||||||||||||||||||||||||||||||

Sbjct 219227 TGTTAGTGAAATACACCAGTCTAAATAAGCGTAGCACTAATTTAATTGCCCGAGTTCGTG 219286

Query 508631 TGCTATGTATAAATAATGAAAATATATACCAAAGCTGGTATTAAACGTAATTTAGGGGGT 508690

||||||||||||||||||||||||||||||||||||||||||||||||||||||||||||

Sbjct 219287 TGCTATGTATAAATAATGAAAATATATACCAAAGCTGGTATTAAACGTAATTTAGGGGGT 219346

Query 508691 ACACCGGTAAGCCGTCAGTTATCTTTGTACCAGGGCGCAGCTTGATGTAGAATTTCTCCC 508750

||||||||||||||||||||||||||||||||||||||||||||||||||||||||||||

Sbjct 219347 ACACCGGTAAGCCGTCAGTTATCTTTGTACCAGGGCGCAGCTTGATGTAGAATTTCTCCC 219406

Query 508751 CTTTCCTGTGGAAAGTACTAGTATTAGAATATCTTTGATATGGCTGAAGAAGATTCATAG 508810

||||||||||||||||||||||||||||||||||||||||||||||||||||||||||||

Sbjct 219407 CTTTCCTGTGGAAAGTACTAGTATTAGAATATCTTTGATATGGCTGAAGAAGATTCATAG 219466

Query 508811 TGAATTTCACAATAATTGTATGTTCTGAATAAATAATTTGTGGAGTGCAACATAAAAAAG 508870

||||||||||||||||||||||||||||||||||||||||||||||||||||||||||||

Sbjct 219467 TGAATTTCACAATAATTGTATGTTCTGAATAAATAATTTGTGGAGTGCAACATAAAAAAG 219526

Query 508871 TGCTTGGAACAATAGAAATAGAAATAGAAATAGCATGAACTACGAAGTATCCATTCAAAT 508930

|||||||||||||||||||||||||| |||||||||||||||||||||||||||||||||

Sbjct 219527 TGCTTGGAACAATAGAAATAGAAATATAAATAGCATGAACTACGAAGTATCCATTCAAAT 219586

Query 508931 AGAGAGAAAATGATCTTCGATTGAGAAAAGAACTGCACTTGGGAAAAAGGGAAGGATTTA 508990

||||||||||||||||||||||||||||||||||||||||||||||||||||||||||||

Sbjct 219587 AGAGAGAAAATGATCTTCGATTGAGAAAAGAACTGCACTTGGGAAAAAGGGAAGGATTTA 219646

Query 508991 CTAGCAGCTACCTCACTGGGTATGAAATCGTTAGCCGCTCCCAAGCCAAATCTGAAGACT 509050

||||||||||||||||||||||||||||||||||||||||||||||||||||||||||||

Sbjct 219647 CTAGCAGCTACCTCACTGGGTATGAAATCGTTAGCCGCTCCCAAGCCAAATCTGAAGACT 219706

Query 509051 TAGTGCAAAACCGGGCAGAAAGAATTGACTCTCTTTCTGTTCTGTCTGCTTGGGGGGACA 509110

||||||||||||||||||||||||||||||||||||||||||||||||||||||||||||

Sbjct 219707 TAGTGCAAAACCGGGCAGAAAGAATTGACTCTCTTTCTGTTCTGTCTGCTTGGGGGGACA 219766

Query 509111 GCACTTTCTATTTGCGGTGAGGTCAAGCTTACTCCCTGTAAAGCGAGGAAGGAATTGGCT 509170

||||||||||||||||||||||||||||||||||||||||||||||||||||||||||||

Sbjct 219767 GCACTTTCTATTTGCGGTGAGGTCAAGCTTACTCCCTGTAAAGCGAGGAAGGAATTGGCT 219826

Query 509171 CGCTTCGACGATTCCACAAGCTAACAAGAATTTATGCCAGCGAGGAAGGGCCTACTTAGT 509230

||||||||||||||||||||||||||||||||||||||||||||||||||||||||||||

Sbjct 219827 CGCTTCGACGATTCCACAAGCTAACAAGAATTTATGCCAGCGAGGAAGGGCCTACTTAGT 219886

Query 509231 GGAATGGAAAGCTAGGTATATTTATTCGAGATATTTCGATGATGATGGAGAAGATCCGTT 509290

||||||||||||||||||||||||||||||||||||||||||||||||||||||||||||

Sbjct 219887 GGAATGGAAAGCTAGGTATATTTATTCGAGATATTTCGATGATGATGGAGAAGATCCGTT 219946

Query 509291 CTGAGATTCTTATTATGGTACTGATTTTGATGGATGTTCAGCTACCCCAACCTCGGAAAC 509350

||||||||||||||||||||||||||||||||||||||||||||||||||||||||||||

Sbjct 219947 CTGAGATTCTTATTATGGTACTGATTTTGATGGATGTTCAGCTACCCCAACCTCGGAAAC 220006

Query 509351 AACATCTCCCACGACACGCGAATTACCCGCAACGAGTTCACTCTTCGGAAAGCCCCTACT 509410

||||||||||||||||||||||||||||||||||||||||||||||||||||||||||||

Sbjct 220007 AACATCTCCCACGACACGCGAATTACCCGCAACGAGTTCACTCTTCGGAAAGCCCCTACT 220066

Query 509411 ACCGGAATAAGATCCATTCGAGCGATCAACTTCATTGTTTGACAGTTTGTTTTCGAATTT 509470

||||||||||||||||||||||||||||||||||||||||||||||||||||||||||||

Sbjct 220067 ACCGGAATAAGATCCATTCGAGCGATCAACTTCATTGTTTGACAGTTTGTTTTCGAATTT 220126

Query 509471 CACACTCCCTGGTACTTGATATGGAGCCCCTTAGAAACTTCCTCCTTACGGACTACACCT 509530

||||||||||||||||||||||||||||||||||||||||||||||||||||||||||||

Sbjct 220127 CACACTCCCTGGTACTTGATATGGAGCCCCTTAGAAACTTCCTCCTTACGGACTACACCT 220186

Query 509531 TCGCTCTTAACGAACATAGAACTCGCCCTTGGATTTGACTGGGTTAGGGAGTATTTGTAA 509590

||||||||||||||||||||||||||||||||||||||||||||||||||||||||||||

Sbjct 220187 TCGCTCTTAACGAACATAGAACTCGCCCTTGGATTTGACTGGGTTAGGGAGTATTTGTAA 220246

Query 509591 GGACCAACTTGAGCAAAACATTGCTTTGGGTCTAAATCCAATACTCGTTGAGCAACATGA 509650

||||||||||||||||||||||||||||||||||||||||||||||||||||||||||||

Sbjct 220247 GGACCAACTTGAGCAAAACATTGCTTTGGGTCTAAATCCAATACTCGTTGAGCAACATGA 220306

Query 509651 TCCTTTTTCCTTCTTTTCTTGGCCAATTCTTTAAAAGATACTTTTTGTATGTACTGTTGA 509710

||||||||||||||||||||||||||||||||||||||||||||||||||||||||||||

Sbjct 220307 TCCTTTTTCCTTCTTTTCTTGGCCAATTCTTTAAAAGATACTTTTTGTATGTACTGTTGA 220366

Query 509711 ACCATAACTGTGAAACCCCTATTAGTACCAGTGTAAACCAACTGCTCAGAATGGCAATAG 509770

||||||||||||||||||||||||||||||||||||||||||||||||||||||||||||

Sbjct 220367 ACCATAACTGTGAAACCCCTATTAGTACCAGTGTAAACCAACTGCTCAGAATGGCAATAG 220426

Query 509771 AAAGTACTTTATGGACCAATATTTGCATGTTGAATCAGGAAAGAGAATCATGAGATAAAA 509830

||||||||||||||||||||||||||||||||||||||||||||||||||||||||||||

Sbjct 220427 AAAGTACTTTATGGACCAATATTTGCATGTTGAATCAGGAAAGAGAATCATGAGATAAAA 220486

Query 509831 TAGAACGACACAAGCCAATGAAGTCGATATGCAAGCTCATAGTATAAGAGACAAGCGTCA 509890

||||||||||||||||||||||||||||||||||||||||||||||||||||||||||||

Sbjct 220487 TAGAACGACACAAGCCAATGAAGTCGATATGCAAGCTCATAGTATAAGAGACAAGCGTCA 220546

Query 509891 TGCAAATAGTTGCACAGCTGTGGAGCAAGCAAGAACCACCAGTAAAAGAAAGCACCCCTG 509950

||||||||||||||||||||||||||||||||||||||||||||||||||||||||||||

Sbjct 220547 TGCAAATAGTTGCACAGCTGTGGAGCAAGCAAGAACCACCAGTAAAAGAAAGCACCCCTG 220606

Query 509951 AAAAATTCCCCTTTGGTTGTTAAGGGGAGATTTCTAGTCTCTGATCTCGTCTGGGGTAAA 510010

||||||||||||||||||||||||||||||||||||||||||||||||||||||||||||

Sbjct 220607 AAAAATTCCCCTTTGGTTGTTAAGGGGAGATTTCTAGTCTCTGATCTCGTCTGGGGTAAA 220666

Query 510011 GTGAAAAGTAACCCAAGGTGGCCGGGTGTCATAATAAACCCATCTTACTTACGGGTCAAA 510070

||||||||||||||||||||||||||||||||||||||||||||||||||||||||||||

Sbjct 220667 GTGAAAAGTAACCCAAGGTGGCCGGGTGTCATAATAAACCCATCTTACTTACGGGTCAAA 220726

Query 510071 CCTCGCGTCATTAAATATCAAAAAAGAAAGGCCTTTTTAGTGACTTATTATGGGGATAAG 510130

||||||||||||||||||||||||||||||||||||||||||||| ||||||||||

Sbjct 220727 CCTCGCGTCATTAAATATCAAAAAAGAAAGGCCTTTTTAGTGACT-----TGGGGATAAG 220781

Query 510131 ACCTTTGCATGGTTTGATTGATGAGTCCCAGTTGAAAGCCTTCCTTCAAAGCTTCTTACA 510190

||||||||||||||||||||||||||||||||||||||||||||||||||||||||||||

Sbjct 220782 ACCTTTGCATGGTTTGATTGATGAGTCCCAGTTGAAAGCCTTCCTTCAAAGCTTCTTACA 220841

Query 510191 TTTGGAAAAGCAAGCGAGTTAAGATGCGTTTTCTAATGCAGTTAGGAGTGTGTTAATAGA 510250

||||||||||||||||||||||||||||||||||||||||||||||||||||||||||||

Sbjct 220842 TTTGGAAAAGCAAGCGAGTTAAGATGCGTTTTCTAATGCAGTTAGGAGTGTGTTAATAGA 220901

Query 510251 GGTCTCTAGAAGGTTTGAGGTTGGTTCAATTTGCTCTTGCTTGGAAGAAAAGTTGTTTGC 510310

||||||||||||||||||||||||||||||||||||||||||||||||||||||||||||

Sbjct 220902 GGTCTCTAGAAGGTTTGAGGTTGGTTCAATTTGCTCTTGCTTGGAAGAAAAGTTGTTTGC 220961

Query 510311 TCAAGAAGTCTATCCAAAATGAGAAAATGCTGGTGTAAGGCAAGGGTTTTTGAACTGTGC 510370

||||||||||||||||||||||||||||||||||||||||||||||||||||||||||||

Sbjct 220962 TCAAGAAGTCTATCCAAAATGAGAAAATGCTGGTGTAAGGCAAGGGTTTTTGAACTGTGC 221021

Query 510371 TTTTGAGACAAAATAGGTTATTATTAGGCTTCTTTCTTTTTTCAAAGATTTGGCACTGGT 510430

|||||||||||||||| |||||||||||||||||||||||||||||||||||||||||||

Sbjct 221022 TTTTGAGACAAAATAGATTATTATTAGGCTTCTTTCTTTTTTCAAAGATTTGGCACTGGT 221081

Query 510431 ACCGGCTAAGACCACAACAGACAAGCTGGAGCTCGTGATGGTTACATCTCAGAAAAAAGC 510490

||||||||||||||||||||||||||||||||||||||||||||||||||||||||||||

Sbjct 221082 ACCGGCTAAGACCACAACAGACAAGCTGGAGCTCGTGATGGTTACATCTCAGAAAAAAGC 221141

Query 510491 CTTTTACCGGTCTTCGGGTTACTCTTTACCTCCAAAATACCTTCCTTTTTGGCCTCTAGA 510550

||||||||||||||||||||||||||||||||||||||||||||||||||||||||||||

Sbjct 221142 CTTTTACCGGTCTTCGGGTTACTCTTTACCTCCAAAATACCTTCCTTTTTGGCCTCTAGA 221201

Query 510551 GGAGTAAATTGAACCAGTGATTGTACCAACTAAAAGCGAGAAATTACCAGAGAACCGCAT 510610

||||||||||||||||||||||||||||||||||||||||||||||||||||||||||||

Sbjct 221202 GGAGTAAATTGAACCAGTGATTGTACCAACTAAAAGCGAGAAATTACCAGAGAACCGCAT 221261

Query 510611 TATGCAAGTAAAAACCCCGCCTGCTGTGGCTCCACCTGCTTGAACTCCAAGTGTGACTTC 510670

||||||||| ||||||||||||||||||||||||||||||||||||||||||||||||||

Sbjct 221262 TATGCAAGTCAAAACCCCGCCTGCTGTGGCTCCACCTGCTTGAACTCCAAGTGTGACTTC 221321

Query 510671 TACTTCAGAAAAAGACTCTCATCAGTCCGACATTAGAAGGAAAAGAACGATGAAAGAGTC 510730

||||||||||||||||||||||||||||||||||||||||||||||||||||||||||||

Sbjct 221322 TACTTCAGAAAAAGACTCTCATCAGTCCGACATTAGAAGGAAAAGAACGATGAAAGAGTC 221381

Query 510731 CAACTTACTTCACTCAAAGAGTAGAAGCTTCATCCTCCTAGCGAGCCAGCCGACAAGAAA 510790

||||||||||||||||||||||||||||||||||||||||||||||||||||||||||||

Sbjct 221382 CAACTTACTTCACTCAAAGAGTAGAAGCTTCATCCTCCTAGCGAGCCAGCCGACAAGAAA 221441

Query 510791 AGAAAGCAGTTTAGTAACTAACAAGTTGAATATTAAGTAGTTGAGTGTTTAGTGTAGTTT 510850

||||||||||||||||||||||||||||||||||||||||||||||||||||||||||||

Sbjct 221442 AGAAAGCAGTTTAGTAACTAACAAGTTGAATATTAAGTAGTTGAGTGTTTAGTGTAGTTT 221501

Query 510851 GAAGAAAGAGCGAAGTAAATATTTTCCAATAGACAGTTTTGTTTAAAGGAGCTTATGGGC 510910

||||||||||||||||||||||||||||||||||||||||||||||||||||||||||||

Sbjct 221502 GAAGAAAGAGCGAAGTAAATATTTTCCAATAGACAGTTTTGTTTAAAGGAGCTTATGGGC 221561

Query 510911 CTTACGAGAGTACTTTTTAAAGTAGTGACGGGAAAAGAAAGTGACGTTTGCCCACAAGGC 510970

||||||||||||||||||||||||||||||||||||||||||||||||||||||||||||

Sbjct 221562 CTTACGAGAGTACTTTTTAAAGTAGTGACGGGAAAAGAAAGTGACGTTTGCCCACAAGGC 221621

Query 510971 ACAAGCAGACAAGAAGGAAGATAGGCGGCCAATACCTAATTAATAATATATCATTTTTAG 511030

||||||||||||||||||||||||||||||||||||||||||||||||||||||||||||

Sbjct 221622 ACAAGCAGACAAGAAGGAAGATAGGCGGCCAATACCTAATTAATAATATATCATTTTTAG 221681

Query 511031 ATACTAACTGGTATTACCTTTTCTTAACTTTAATCAAATACCCTTCCTTACACTGCAAAA 511090

||||||||||||||||||||||||||||||||||||||||||||| ||||||||||

Sbjct 221682 ATACTAACTGGTATTACCTTTTCTTAACTTTAATCAAATACCCTT-----CACTGCAAAA 221736

Query 511091 GACGTGTACAAGCTCTTCCTTATGTTGTCAAACATGTCCTTACTAGCGATTTTATTACTT 511150

||||||||||||||||||||||||||||||||||||||||||||||||||||||||||||

Sbjct 221737 GACGTGTACAAGCTCTTCCTTATGTTGTCAAACATGTCCTTACTAGCGATTTTATTACTT 221796

Query 511151 ATAGGTCTAAAATAAGAACTTGTCTGCTATCTATGAGTACTCTTCTTCTTTAACCAACTC 511210

||||||||||||||||||||||||||||||||||||||||||||||||||||||||||||

Sbjct 221797 ATAGGTCTAAAATAAGAACTTGTCTGCTATCTATGAGTACTCTTCTTCTTTAACCAACTC 221856

Query 511211 TTCATCACACTTAACTAAGGAGCATTTGTATGTATCACTTAATGTTTATGGTAACTCCTT 511270

||||||||||||||||||||||||||||||||||||||||||||||||||||||||||||

Sbjct 221857 TTCATCACACTTAACTAAGGAGCATTTGTATGTATCACTTAATGTTTATGGTAACTCCTT 221916

Query 511271 ATTAGAGCATGGATTGCCAGGGTAGTCTCTTTCTCACCACACCGAAAACATAAGCCGGCG 511330

||||||||||||||||||||||||||||||||||||||||||||||||||||||||||||

Sbjct 221917 ATTAGAGCATGGATTGCCAGGGTAGTCTCTTTCTCACCACACCGAAAACATAAGCCGGCG 221976

Query 511331 AGTCGCCCTCCAAATTACTTGATTTCCCTGAAGTTTAGGATGATAATGCTTCCTTGGATC 511390

|||||||||||||||||||||||||||||||||||||||||| |||||||||||| ||||

Sbjct 221977 AGTCGCCCTCCAAATTACTTGATTTCCCTGAAGTTTAGGATGCTAATGCTTCCTTTGATC 222036

Query 511391 TTGTAGCTTTTTCTCACCTTCATAAGGTCGAACATCATGTTAGCTTTATGAAGTGAATAG 511450

||||||||||||||||||||||||||||||||||||||||||||||||||||||||||||

Sbjct 222037 TTGTAGCTTTTTCTCACCTTCATAAGGTCGAACATCATGTTAGCTTTATGAAGTGAATAG 222096

Query 511451 TTGAAAAGATTGATCACAAGCCATAATGTAACGAAGCGCCTTTCCGAATGAAGAGGAAGC 511510

||||||||||||||||||||||||||||||||||||||||||||||||||||||||||||

Sbjct 222097 TTGAAAAGATTGATCACAAGCCATAATGTAACGAAGCGCCTTTCCGAATGAAGAGGAAGC 222156

Query 511511 ACATTGAAGTCACAATCCTGAAGCAGGCGCTTAAGAAGAAGACGACATACTATAAATGTA 511570

||||||||||||||||||||||||||||||||||||||||||||||||||||||||||||

Sbjct 222157 ACATTGAAGTCACAATCCTGAAGCAGGCGCTTAAGAAGAAGACGACATACTATAAATGTA 222216

Query 511571 CAACTCAATAGTCAAGCGCTACTTTGGGCGAGTCTACACTTAGTCAAGGGTGCCCCTTAT 511630

||||||||||||||||||||||||||||||||||||||||||||||||||||||||||||

Sbjct 222217 CAACTCAATAGTCAAGCGCTACTTTGGGCGAGTCTACACTTAGTCAAGGGTGCCCCTTAT 222276

Query 511631 CTACTTAGCACTTATAAAAATGGCTGGCTAAATTAGAGAAAGAAAGAGCATCTTTCGCCC 511690

||||||||||||||||||||||||||||||||||||||||||||||||||||||||||||

Sbjct 222277 CTACTTAGCACTTATAAAAATGGCTGGCTAAATTAGAGAAAGAAAGAGCATCTTTCGCCC 222336

Query 511691 GTTAGTGaaaacagaaaaaaggaaaaaaCAGAGTGCACATGCTTTAGAGAGCATCAGCAG 511750

||||||||||||||||||||||||||||||||||||||||||||||||||||||

Sbjct 222337 ------GAAAACAGAAAAAAGGAAAAAACAGAGTGCACATGCTTTAGAGAGCATCAGCAG 222390

Query 511751 GTATGGCAAGCCTATTCAAAAAGATGAAAGCTTGTTAACCAAACAGCTTAGTAGGATCCG 511810

||||||||||||||||||||||||||||||||||||||||||||||||||||||||||||

Sbjct 222391 GTATGGCAAGCCTATTCAAAAAGATGAAAGCTTGTTAACCAAACAGCTTAGTAGGATCCG 222450

Query 511811 TACCTAGTTAAACTGTTTTGAGTTAATAAGAGCAGAAGATGTGTTAAGCGTAtttttttt 511870

||||||||||||||||||||||||||||||||||||||||||||||||||||||||||||

Sbjct 222451 TACCTAGTTAAACTGTTTTGAGTTAATAAGAGCAGAAGATGTGTTAAGCGTATTTTTTTT 222510

Query 511871 ttGTAACAGCGTAGTCAGACCCCTTTTTCCTTGAGCTTATCCTTTTTATTCTGATGAGCC 511930

||||||||||||||||||||||||||||||||||||||||||||||||||||||||||||

Sbjct 222511 TTGTAACAGCGTAGTCAGACCCCTTTTTCCTTGAGCTTATCCTTTTTATTCTGATGAGCC 222570

Query 511931 CAAATCTTATTAGAAAAACCCTATCATATGCATAGAAGCATATATAATGGAAAAGCAGAA 511990

||||||||||||||||||||||||||||||||||||||||||||||||||||||||||||

Sbjct 222571 CAAATCTTATTAGAAAAACCCTATCATATGCATAGAAGCATATATAATGGAAAAGCAGAA 222630

Query 511991 TAAGTGTAGTATaaaaaaaaaGATGTAGGTATTACTTAGCTCCTAAAACATTACAGCTAT 512050

||||||||||||||||||||||||||||||||||||||||||||||||||||||||||||

Sbjct 222631 TAAGTGTAGTATAAAAAAAAAGATGTAGGTATTACTTAGCTCCTAAAACATTACAGCTAT 222690

Query 512051 CACTCCGAGCTGAAACATCTAAAGCTCTATAAAAGCTTTCATGCTGAAGCTTGTTAGTGT 512110

||||||||||||||||||||||||||||||||||||||||||||||||||||||||||||

Sbjct 222691 CACTCCGAGCTGAAACATCTAAAGCTCTATAAAAGCTTTCATGCTGAAGCTTGTTAGTGT 222750

Query 512111 GAAGAAGACCCAATAGCCTATAGCATCAAAGTGGATGTAGTAGTCATGGAACAGTGCAGT 512170

||||||||||||||||||||||||||||||||||||||||||||||||||||||||||||

Sbjct 222751 GAAGAAGACCCAATAGCCTATAGCATCAAAGTGGATGTAGTAGTCATGGAACAGTGCAGT 222810

Query 512171 GCGTGATGTATTATCTTGAACCTTTCGTCAGCCTTGAAACACTAACTTTCTATGTGTCAT 512230

||||||||||||||||||||||||||||||||||||||||||||||||||||||||||||

Sbjct 222811 GCGTGATGTATTATCTTGAACCTTTCGTCAGCCTTGAAACACTAACTTTCTATGTGTCAT 222870

Query 512231 TCCACTCTCGAGCACTATAAAGGAATTGTATCAACCGGGAGTTTATGAACATTCCATGAA 512290

||||||||||||||||||||||||||||||||||||||||||||||||||||||||||||

Sbjct 222871 TCCACTCTCGAGCACTATAAAGGAATTGTATCAACCGGGAGTTTATGAACATTCCATGAA 222930

Query 512291 AATAGCATTTCTTGGTTTGCAAGTATATCGTTCGAAAAAGAGGGTATCTCGGAGGATCCA 512350

||||||||||||||||||||||||||||||||||||||||||||||||||||||||||||

Sbjct 222931 AATAGCATTTCTTGGTTTGCAAGTATATCGTTCGAAAAAGAGGGTATCTCGGAGGATCCA 222990

Query 512351 ATTATGAATGATCAAACAATTGATAAGATGTCAAGCACTAGCAAGAGTATGAAAAACTTC 512410

||||||||||||||||||||||||||||||||||||||||||||||||||||||||||||

Sbjct 222991 ATTATGAATGATCAAACAATTGATAAGATGTCAAGCACTAGCAAGAGTATGAAAAACTTC 223050

Query 512411 AACATATAGTAGGCCATAGGTCTTCATTAGATCCTGGTGCTATAAAGCAATGGTGAAAAG 512470

||||||||||||||||||||||||||||||||||||||||||||||||||||||||||||

Sbjct 223051 AACATATAGTAGGCCATAGGTCTTCATTAGATCCTGGTGCTATAAAGCAATGGTGAAAAG 223110

Query 512471 AGTCAAAAAAGGCATACCCCCGAAAGTGTTCAATATCAAAAAGCATAACATGCTATAGAA 512530

||||||||||||||||||||||||||||||||||||||||||||||||||||||||||||

Sbjct 223111 AGTCAAAAAAGGCATACCCCCGAAAGTGTTCAATATCAAAAAGCATAACATGCTATAGAA 223170

Query 512531 GCCTGCATCCTTCTGGAGTATAAGACGCAAAGGTGTAACGTTTCGATTTAGCTTAATCCC 512590

||||||||||||||||||||||||||||||||||||||||||||||||||||||||||||

Sbjct 223171 GCCTGCATCCTTCTGGAGTATAAGACGCAAAGGTGTAACGTTTCGATTTAGCTTAATCCC 223230

Query 512591 TCAACGTGGTGGGTATATAAACAAATAGAAAGGGTCTGGTTTTTTGGTATTAAACAAAGA 512650

||||||||||||||||||||||||||||||||||||||||||||||||||||||||||||

Sbjct 223231 TCAACGTGGTGGGTATATAAACAAATAGAAAGGGTCTGGTTTTTTGGTATTAAACAAAGA 223290

Query 512651 GGTAGTTAATTAGTAACACTTGATATAGTGTGCAAATGGTTGCCCATGATAGCACACAGG 512710

||||||||||||||||||||||||||||||||||||||||||||||||||||||||||||

Sbjct 223291 GGTAGTTAATTAGTAACACTTGATATAGTGTGCAAATGGTTGCCCATGATAGCACACAGG 223350

Query 512711 TTGATCTAATAACCCCGAAAACCAACCAACACCATTGACATTAGGTTCTGTATAGCTGGA 512770

||||||||||||||||||||||||||||||||||||||||||||||||||||||||||||

Sbjct 223351 TTGATCTAATAACCCCGAAAACCAACCAACACCATTGACATTAGGTTCTGTATAGCTGGA 223410

Query 512771 TACTCGTACTCCCAGAAGCTATACATGTCCTTATAAAACTTGCTCGCTGCACCCAAGAAG 512830

||||||||||||||||||||||||||||||||||||||||||||||||||||||||||||

Sbjct 223411 TACTCGTACTCCCAGAAGCTATACATGTCCTTATAAAACTTGCTCGCTGCACCCAAGAAG 223470

Query 512831 CAAGGCATTGAGCGCTAGCGTGCCTAAAGCGTGAGTTTCCATCCCTTTTCTTGCTGAGTG 512890

||||||||||||||||||||||||||||||||||||||||||||||||||||||||||||

Sbjct 223471 CAAGGCATTGAGCGCTAGCGTGCCTAAAGCGTGAGTTTCCATCCCTTTTCTTGCTGAGTG 223530

Query 512891 AAGTCTAGTGCATTCAGACAATCCGTTTTTGTGCACTATCTTTGTTTAATAAACAAGCGG 512950

||||||||||||||||||||||||||||||||||||||||||||||||||||||||||||

Sbjct 223531 AAGTCTAGTGCATTCAGACAATCCGTTTTTGTGCACTATCTTTGTTTAATAAACAAGCGG 223590

Query 512951 AGACCTTTTTTaaaaaaaCTTACGGGTTAGCTGATGCTTGACTAGATCTGCCAACTCCTT 513010

||||||||||||||||||||||||||||||||||||||||||||||||||||||||||||

Sbjct 223591 AGACCTTTTTTAAAAAAACTTACGGGTTAGCTGATGCTTGACTAGATCTGCCAACTCCTT 223650

Query 513011 ATCCAAAAACTTCTAAATACTAGCCTGAAATTAATAAGAGATCTTGATACAAATCTCGAA 513070

||||||||||||||||||||||||||||||||||||||||||||||||||||||||||||

Sbjct 223651 ATCCAAAAACTTCTAAATACTAGCCTGAAATTAATAAGAGATCTTGATACAAATCTCGAA 223710

Query 513071 GCAAGTGCTCGCTACCAACACTAGCAAGTAGTAAATTAGTTCTCATGCCTAAATCCTCGT 513130

||||||||||||||||||||||||||||||||||||||||||||||||||||||||||||

Sbjct 223711 GCAAGTGCTCGCTACCAACACTAGCAAGTAGTAAATTAGTTCTCATGCCTAAATCCTCGT 223770

Query 513131 GTAAAAGTAAGTAGCTCATGATTTGGTATGCGCTAGATTCAGCATCCCTAGTAATAGGTA 513190

||||||||||||||||||||||||||||||||||||||||||||||||||||||||||||

Sbjct 223771 GTAAAAGTAAGTAGCTCATGATTTGGTATGCGCTAGATTCAGCATCCCTAGTAATAGGTA 223830

Query 513191 TAGTCTGAACCCTCTCAGCAGTGACCCCTCTCAGGCTCTAGGTAATGAAATGTGAAATGA 513250

||||||||||||||||||||||||||||||||||||||||||||||||||||||||||||

Sbjct 223831 TAGTCTGAACCCTCTCAGCAGTGACCCCTCTCAGGCTCTAGGTAATGAAATGTGAAATGA 223890

Query 513251 AATGGAACGGTTTTAGAATGACTACTAAATGATACTAATTCATCGCACCACTTACTTTCT 513310

||||||||||||||||||||||||||||||||||||||||||||||||||||||||||||

Sbjct 223891 AATGGAACGGTTTTAGAATGACTACTAAATGATACTAATTCATCGCACCACTTACTTTCT 223950

Query 513311 TTCTGTCATATACACTAAACGATTAAAGATATCTTTACTCCTTGGAGCCTTAAGATTTAT 513370

||||||||||||||||||||||||||||||||||||||||||||||||||||||||||||

Sbjct 223951 TTCTGTCATATACACTAAACGATTAAAGATATCTTTACTCCTTGGAGCCTTAAGATTTAT 224010

Query 513371 AAAAGACATATGGTGATAGGCAGTTGCTTCTAACTAAGTACGATATGCCTCCTAATGATT 513430

||||||||||||||||||||||||||||||||||||||||||||||||||||||||||||

Sbjct 224011 AAAAGACATATGGTGATAGGCAGTTGCTTCTAACTAAGTACGATATGCCTCCTAATGATT 224070

Query 513431 CAAACTAATAGCTTTCTTAGGCTGAGAAAAGCATATAAGACTTATCACTAGATCACGCTC 513490

||||||||||||||||||||||||||||||||||||||||||||||||||||||||||||

Sbjct 224071 CAAACTAATAGCTTTCTTAGGCTGAGAAAAGCATATAAGACTTATCACTAGATCACGCTC 224130

Query 513491 GTGGAAAAGAATATCTGATCTATAGATTCTACCACGTAAATCAAAGAAGGCCAATAAGTC 513550

||||||||||||||||||||||||||||||||||||||||||||||||||||||||||||

Sbjct 224131 GTGGAAAAGAATATCTGATCTATAGATTCTACCACGTAAATCAAAGAAGGCCAATAAGTC 224190

Query 513551 TAATTGATAACCTTCGTATGCCTTAGCTAGTTCAAATAAAAATCTCTCATTCCTAGCTTT 513610

||||||||||||||||||||||||||||||||||||||||||||||||||||||||||||

Sbjct 224191 TAATTGATAACCTTCGTATGCCTTAGCTAGTTCAAATAAAAATCTCTCATTCCTAGCTTT 224250

Query 513611 TGTTGCACACGTTTATCTAATATACTCCAAAGCGATAATCCACGTAACAAGATTTCTTTG 513670

||||||||||||||||||||||||||||||||||||||||||||||||||||||||||||

Sbjct 224251 TGTTGCACACGTTTATCTAATATACTCCAAAGCGATAATCCACGTAACAAGATTTCTTTG 224310

Query 513671 AAGAACTTCTTCAGTGCAGTTTGTATATATTTGAGACAAGAAAGATAAGTAGGGATAAGT 513730

||||||||||||||||||||||||||||||||||||||||||||||||||||||||||||

Sbjct 224311 AAGAACTTCTTCAGTGCAGTTTGTATATATTTGAGACAAGAAAGATAAGTAGGGATAAGT 224370

Query 513731 AGGCCAACTTTCACTAAATGCTCCTTATTTTATCTTCTAGAAAGTGTCAAACTTCGCTTC 513790

||||||||||||||||||||||||||||||||||||||||||||||||||||||||||||

Sbjct 224371 AGGCCAACTTTCACTAAATGCTCCTTATTTTATCTTCTAGAAAGTGTCAAACTTCGCTTC 224430

Query 513791 ATGAAAACCAACACTTTGAAGTAGTGTAAGTGTATAGCACAATTGAATGTAGGATTTATC 513850

||||||||||||||||||||||||||||||||||||||||||||||||||||||||||||

Sbjct 224431 ATGAAAACCAACACTTTGAAGTAGTGTAAGTGTATAGCACAATTGAATGTAGGATTTATC 224490

Query 513851 TAGTAGTAAAGCGTAATAGTGGTCAGAATCTTTAGTGTTGTGTAAGCGGGATGGCATATA 513910

||||||||||||||||||||||||||||||||||||||||||||||||||||||||||||

Sbjct 224491 TAGTAGTAAAGCGTAATAGTGGTCAGAATCTTTAGTGTTGTGTAAGCGGGATGGCATATA 224550

Query 513911 CATTTTATACGTACCTGGATTCAGGTAACAACCAACTAATTCACCAACTTTTGGTATTAC 513970

||||||||||||||||||||||||||||||||||||||||||||||||||||||||||||

Sbjct 224551 CATTTTATACGTACCTGGATTCAGGTAACAACCAACTAATTCACCAACTTTTGGTATTAC 224610

Query 513971 ACATTTAGACCTACCATCCTCTACTCCCTATCCAACAGGAACACAAACCATAGGTAATTT 514030

||||||||||||||||||||||||||||||||||||||||||||||||||||||||||||

Sbjct 224611 ACATTTAGACCTACCATCCTCTACTCCCTATCCAACAGGAACACAAACCATAGGTAATTT 224670

Query 514031 ACAAGCAAGTGGTAATAATTCTACATAAAATAAGGGTTAAACAAATGATTTTTGACTTAG 514090

||||||||||||||||||||||||||||||||||||| ||||||||||||||||||||||

Sbjct 224671 ACAAGCAAGTGGTAATAATTCTACATAAAATAAGGGTGAAACAAATGATTTTTGACTTAG 224730

Query 514091 GTACAAATTCACCAGCTACTTTCTTCACACCATCTGCTGATTTACCAGAAGCCGCTACGC 514150

||||||||||||||||||||||||||||||||||||||||||||||||||||||||||||

Sbjct 224731 GTACAAATTCACCAGCTACTTTCTTCACACCATCTGCTGATTTACCAGAAGCCGCTACGC 224790

Query 514151 CCTCTCATAACCAGAAACTCAACTAAATAAGTGTCTATGGTGAAAGCCACTTTTGTGTGA 514210

||||||||||||||||||||||||||||||||||||||||||||||||||||||||||||

Sbjct 224791 CCTCTCATAACCAGAAACTCAACTAAATAAGTGTCTATGGTGAAAGCCACTTTTGTGTGA 224850

Query 514211 ACAGGAGTTCGATCCGGGCACCAACCTCCAAATTCCATTTACCTTCGTCCAGAGTCACAG 514270

||||||||||||||||||||||||||||||||||||||||||||||||||||||||||||

Sbjct 224851 ACAGGAGTTCGATCCGGGCACCAACCTCCAAATTCCATTTACCTTCGTCCAGAGTCACAG 224910

Query 514271 CCTTCTCAACCTCTGTCAGGAAACGTTCACTGGGGAGTTTCTCATCAAAATAGGACCAGC 514330

||||||||||||||||||||||||||||||||||||||||||||||||||||||||||||

Sbjct 224911 CCTTCTCAACCTCTGTCAGGAAACGTTCACTGGGGAGTTTCTCATCAAAATAGGACCAGC 224970

Query 514331 CTTACTACGTGGAATAGCCACTTCGTACATAAAGACAAGGGCGTAGCTGGAGTAGGGTTA 514390

|| |||||||||||||||||||||||||||||||||||||||||||||||||||||||||

Sbjct 224971 CTGACTACGTGGAATAGCCACTTCGTACATAAAGACAAGGGCGTAGCTGGAGTAGGGTTA 225030

Query 514391 AGAAAGACGATGTTCGGTGAGGATTCAGACACTTCTGAGAATAGCATAGATATAGAACGA 514450

||||||||||||||||||||||||||||||||||||||||||||||||||||||||||||

Sbjct 225031 AGAAAGACGATGTTCGGTGAGGATTCAGACACTTCTGAGAATAGCATAGATATAGAACGA 225090

Query 514451 AGGCATAACAACTCTAGGTCATATTGCTCCAAAGAATTGAGTAAACCAACCTCAGCATCT 514510

||||||||||||||||||||||||||||||||||||||||||||||||||||||||||||

Sbjct 225091 AGGCATAACAACTCTAGGTCATATTGCTCCAAAGAATTGAGTAAACCAACCTCAGCATCT 225150

Query 514511 GTTAGAACTTTGGCACTCCCCTTATTAGCTTTTCTAGCTAAAAATGCAATCTTTAGCATT 514570

||||||||||||||||||||||||||||||||||||||||||||||||||||||||||||

Sbjct 225151 GTTAGAACTTTGGCACTCCCCTTATTAGCTTTTCTAGCTAAAAATGCAATCTTTAGCATT 225210

Query 514571 GATCTTATCCGGTTGAATCAACCTTGACATCTGCCCTGGAAACCTACGATATGCAGCTGA 514630

||||||||||||||||||||||||||||||||||||||||||||||||||||||||||||

Sbjct 225211 GATCTTATCCGGTTGAATCAACCTTGACATCTGCCCTGGAAACCTACGATATGCAGCTGA 225270

Query 514631 TGGATCAGCAAAGCTTTCTAAAGACAGAAGTGAAATTGCATGTTGAAGCTCCACCATTTT 514690

||||||||||||||||||||||||||||||||||||||||||||||||||||||||||||

Sbjct 225271 TGGATCAGCAAAGCTTTCTAAAGACAGAAGTGAAATTGCATGTTGAAGCTCCACCATTTT 225330

Query 514691 ACTTTCATTGCTGTTGCCTATACTCGTTAAATAATCTAGACATTTCCAATAGATAAGTTG 514750

||||||||||||||||||||||||||||||||||||||||||||||||||||||||||||

Sbjct 225331 ACTTTCATTGCTGTTGCCTATACTCGTTAAATAATCTAGACATTTCCAATAGATAAGTTG 225390

Query 514751 CCCTACCTTCACCCACCTGATTTGTAAACACATTTTAGACTCACCTGCTTTGAGCTTTTA 514810

|||||||||||||||||||||||||||||||||||||||||||||||||||||||||| |

Sbjct 225391 CCCTACCTTCACCCACCTGATTTGTAAACACATTTTAGACTCACCTGCTTTGAGCTTTGA 225450

Query 514811 CTCAAACTCATTTATATATAATAGAAATCGGGGGTATAGTGAAAGAAGAATATGAGCGAG 514870

||||||||||||||||||||||||||||||||||||||||||||||||||||||||||||

Sbjct 225451 CTCAAACTCATTTATATATAATAGAAATCGGGGGTATAGTGAAAGAAGAATATGAGCGAG 225510

Query 514871 TAAATGTATAAGGtttttttGAAAACATGTTTGGTACGAATGCCATGTGAATTGGTTCTA 514930

||||||||||||||||||||||||||||||||||||||||||||||||||||||||||||

Sbjct 225511 TAAATGTATAAGGTTTTTTTGAAAACATGTTTGGTACGAATGCCATGTGAATTGGTTCTA 225570

Query 514931 TTGAATAAGGTAAACACTCGAGTTGTATTATTGAAATACGAAAGAGGACGCCTTCGGTGA 514990

||||||||||||||||||||||||||||||||||||||||||||||||||||||||||||

Sbjct 225571 TTGAATAAGGTAAACACTCGAGTTGTATTATTGAAATACGAAAGAGGACGCCTTCGGTGA 225630

Query 514991 ACTTGAAAACTTTCACTGTACAAAAGCAACAATCTTCATTTACACTAACTTTACCAGACT 515050

||||||||||||||||||||||||||||||||||||||||||||||||||||||||||||

Sbjct 225631 ACTTGAAAACTTTCACTGTACAAAAGCAACAATCTTCATTTACACTAACTTTACCAGACT 225690

Query 515051 CAAACAAAAGAATCAGAGTTTATGATACACAAGGCAAGTGGGTCGATACTCAACCTTTTA 515110

||||||||||||||||||||||||||||||||||||||||||||||||||||||||||||

Sbjct 225691 CAAACAAAAGAATCAGAGTTTATGATACACAAGGCAAGTGGGTCGATACTCAACCTTTTA 225750

Query 515111 ATAAAGAAGCTGTCCCAGGAGTAGATGATTCTATACAAATACTAATCAAAGCTTTATCAG 515170

||||||||||||||||||||||||||||||||||||||||||||||||||||||||||||

Sbjct 225751 ATAAAGAAGCTGTCCCAGGAGTAGATGATTCTATACAAATACTAATCAAAGCTTTATCAG 225810

Query 515171 AGAAAATCTCAGATCAACAGGGTTCTGAGAAGAAAACACCTGATCAACAGAGCTCTGAGA 515230

||||||||||||||||||||||||||||||||||||||||||||||||||||||||||||

Sbjct 225811 AGAAAATCTCAGATCAACAGGGTTCTGAGAAGAAAACACCTGATCAACAGAGCTCTGAGA 225870

Query 515231 AGAAAACCCCACAACTAAACCGCTGGTTGGAgaaagaacaaagagagctgaagaagcgga 515290

||||||||||||||||||||||||||||||||||||||||||||||||||||||||||||

Sbjct 225871 AGAAAACCCCACAACTAAACCGCTGGTTGGAGAAAGAACAAAGAGAGCTGAAGAAGCGGA 225930

Query 515291 agaagaaacagcttaaaaggggaacagtgaaaaaaCCACCTAACAAAGCACGCTGTGATG 515350

||||||||||||||||||||||||||||||||||||||||||||||||||||||||||||

Sbjct 225931 AGAAGAAACAGCTTAAAAGGGGAACAGTGAAAAAACCACCTAACAAAGCACGCTGTGATG 225990

Query 515351 CAGTGAAAGATTCAACCGTACGAAACCCTGTGTTTTTGTCCATTTTCCCCTCTTCCCTTG 515410

|||||||||||||||||||||||||||| |||||||||||||||||||||||||||||||

Sbjct 225991 CAGTGAAAGATTCAACCGTACGAAACCCCGTGTTTTTGTCCATTTTCCCCTCTTCCCTTG 226050

Query 515411 GGAGACTGCCCTATTGTGTGTGAAAAGTTGGATTCCCCGTGTTTTCGGGCATAACTTCAT 515470

||||||||||||||||||||||||||||||||||||||||||||||||||||||||||||

Sbjct 226051 GGAGACTGCCCTATTGTGTGTGAAAAGTTGGATTCCCCGTGTTTTCGGGCATAACTTCAT 226110

Query 515471 TTTCCTAGAGTCAGATGCAACCAAAACCTAACAAAGTCTAAGCAAAGTGCACGAGCAGCT 515530

||||||||||||||||||||||||||||||||||||||||||||||||||||||||||||

Sbjct 226111 TTTCCTAGAGTCAGATGCAACCAAAACCTAACAAAGTCTAAGCAAAGTGCACGAGCAGCT 226170

Query 515531 AGTAGTATAGGTCTTGTGTATCTAGTATACAAGTCAAGTAGAATGTTCAGGTAAGCTTGA 515590

||||||||||||||||||||||||||||||||||||||||||||||||||||||||||||

Sbjct 226171 AGTAGTATAGGTCTTGTGTATCTAGTATACAAGTCAAGTAGAATGTTCAGGTAAGCTTGA 226230

Query 515591 TTTCATTCTCCGAGTACAGGTAGATTCTTAGCACTTCAAAGAAGATTTTTGAAATAAAGA 515650

||||||||||||||||||||||||||||||||||||||||||||||||||||||||||||

Sbjct 226231 TTTCATTCTCCGAGTACAGGTAGATTCTTAGCACTTCAAAGAAGATTTTTGAAATAAAGA 226290

Query 515651 AGAAGGTCGGGAGAATCCTTCAACAAGAGCAAAACCCCCTTTCTCCACCCCTGCGTTTAT 515710

||||||||||||||||||||||||||||||||||||||||||||||||||||||||||||

Sbjct 226291 AGAAGGTCGGGAGAATCCTTCAACAAGAGCAAAACCCCCTTTCTCCACCCCTGCGTTTAT 226350

Query 515711 GCAGTCTACAATCCCTTTGTAAAGAAAACATTTGTTTCCTGGTTAGTCCGTGGAGCAAGG 515770

||||||||||||||||||||||||||||||||||||||||||||||||||||||||||||

Sbjct 226351 GCAGTCTACAATCCCTTTGTAAAGAAAACATTTGTTTCCTGGTTAGTCCGTGGAGCAAGG 226410

Query 515771 CTTCGTAATAGTGAGGCTTACTAGTCAAGTAAGATCCGCTTGATCCTCTAAATTAGTAGC 515830

||||||||||||||||||||||||||||||||||||||||||||||||||||||||||||

Sbjct 226411 CTTCGTAATAGTGAGGCTTACTAGTCAAGTAAGATCCGCTTGATCCTCTAAATTAGTAGC 226470

Query 515831 CTCCCTCTTCTCCGGCTATGGAATTCTCCCTCACGTGGGAAGCTTTTCCTATTCTCTACA 515890

||||||||||||||||||||||||||||||||||||||||||||||||||||||||||||

Sbjct 226471 CTCCCTCTTCTCCGGCTATGGAATTCTCCCTCACGTGGGAAGCTTTTCCTATTCTCTACA 226530

Query 515891 AATCCTAGCAAGGTAAGTCCCCGCCAGGGTTAAACCCCGGTCCTAGCCTCAAAAATAGGA 515950

||||||||||||||||||||||||||||||||||||||||||||||||||||||||||||

Sbjct 226531 AATCCTAGCAAGGTAAGTCCCCGCCAGGGTTAAACCCCGGTCCTAGCCTCAAAAATAGGA 226590

Query 515951 AGGAAAAAAGGGGAAAGTCAACTAGGTATCTCGATCATTAGACTCAATAAAGGGTGGGTA 516010

||||||||||||||||||||||||||||||||||||||||||||||||||||||||||||

Sbjct 226591 AGGAAAAAAGGGGAAAGTCAACTAGGTATCTCGATCATTAGACTCAATAAAGGGTGGGTA 226650

Query 516011 CAGTTTCTGGGCAACCCCATCTGACTTGATCTTACTTACTCTGCTCACAGAAATCCAAGA 516070

||||||||||||||||||||||||||||||||||||||||||||||||| |||||||| |

Sbjct 226651 CAGTTTCTGGGCAACCCCATCTGACTTGATCTTACTTACTCTGCTCACATAAATCCAATA 226710

Query 516071 GTTTTCAAGCGGGAAAGAGACTGACTTCAAATAGTAGCTCAACCAGAAAAAGAGGAATAG 516130

||||| ||||||||||||||||||||||||||||||||||||||||||||||||||||||

Sbjct 226711 GTTTTAAAGCGGGAAAGAGACTGACTTCAAATAGTAGCTCAACCAGAAAAAGAGGAATAG 226770

Query 516131 AGTATTAGGCCTTAAGCACAGGAACTTACATATCCTTGCCCGAGCAAATAGCCTCCGGTC 516190

||||||||||||||||||||||||||||||||||||||||||||||||||||||||||||

Sbjct 226771 AGTATTAGGCCTTAAGCACAGGAACTTACATATCCTTGCCCGAGCAAATAGCCTCCGGTC 226830

Query 516191 AACTTCTTCTTCGGAACTGACGTTAGAGACTAAAGACTAAGTAAACCGTTCAAGGTCAAA 516250

||||||||||||||||||||||||||||||||||||||||||||||||||||||||||||

Sbjct 226831 AACTTCTTCTTCGGAACTGACGTTAGAGACTAAAGACTAAGTAAACCGTTCAAGGTCAAA 226890

Query 516251 TAGCTCGGCGGAAATCTAACTACCTGAGGACAGAAGAAAAGACTTTCAACAACCTGACGC 516310

||||||||||||||||||||||||||||||||||||||||||||||||||||||||||||

Sbjct 226891 TAGCTCGGCGGAAATCTAACTACCTGAGGACAGAAGAAAAGACTTTCAACAACCTGACGC 226950

Query 516311 ACTTCCTTAAAGAAAAGAACTGTGCTTAACTGGGTGGTAAATCTCAATAGCTCCGTCCAA 516370

||||||||||||||||||||||||||||||||||||||||||||||||||||||||||||

Sbjct 226951 ACTTCCTTAAAGAAAAGAACTGTGCTTAACTGGGTGGTAAATCTCAATAGCTCCGTCCAA 227010

Query 516371 GCGCTTTGAACAGCTCTTCTGCTTTAAGGCTTAGAAGCTGACAGAAATACCACGATTCCC 516430

||||||||||||||||||||||||||||||||||||||||||||||||||||||||||||

Sbjct 227011 GCGCTTTGAACAGCTCTTCTGCTTTAAGGCTTAGAAGCTGACAGAAATACCACGATTCCC 227070

Query 516431 CGTATTTTTGTCCTACTTGGCAATTTCTCTTCTCTTAAGCGGATCGCCAAATGGACCAAA 516490

||||||||||||||||||||||||||||||||||||||||||||||||||||||||||||

Sbjct 227071 CGTATTTTTGTCCTACTTGGCAATTTCTCTTCTCTTAAGCGGATCGCCAAATGGACCAAA 227130

Query 516491 AAGGAGTGTTTTTGCCGGTAGGGCGGATAATAAAGTGGCAAGGCAGGAATGCTCTTTTTT 516550

||||||||||||||||||||||||||||||||||||||||||||||||||||||||||||

Sbjct 227131 AAGGAGTGTTTTTGCCGGTAGGGCGGATAATAAAGTGGCAAGGCAGGAATGCTCTTTTTT 227190

Query 516551 CATGTATGTGGTACTTGGTCGATTACCTGATTGCCAACTAAACTACATATTATATTGGTT 516610

||||||||||||||||||||||||||||||||||||||||||||||||||||||||||||

Sbjct 227191 CATGTATGTGGTACTTGGTCGATTACCTGATTGCCAACTAAACTACATATTATATTGGTT 227250

Query 516611 GTTGTCACTCTCTGAAATAGATAAAGAAAAGATTTGATCTTTGACTTATATGCGCGCGCG 516670

||||||||||||||||||||||||||||||||||||||||||||||||||||||||||||

Sbjct 227251 GTTGTCACTCTCTGAAATAGATAAAGAAAAGATTTGATCTTTGACTTATATGCGCGCGCG 227310

Query 516671 TGAGTTAATAGTAGACAGATTTCTATTTACGATAACGAATTGGATTCGAACCAATGTCGC 516730

||||||||||||||||||||||||||||||||||||||||||||||||||||||||||||

Sbjct 227311 TGAGTTAATAGTAGACAGATTTCTATTTACGATAACGAATTGGATTCGAACCAATGTCGC 227370

Query 516731 TAGACTACAACCGAATCTGGATCGTGATAATACTTCCTCCTATTCCAAGTCTCTTTGTTT 516790

||||||||||||||||||||||||||||||||||||||||||||||||||||||||||||

Sbjct 227371 TAGACTACAACCGAATCTGGATCGTGATAATACTTCCTCCTATTCCAAGTCTCTTTGTTT 227430

Query 516791 TCGTTCTTCTGACTTTATTAGTAGAGGGTGGTTGCTTTGGAATTCTGTAAGTAGCTCGTG 516850

||||||||||||||||||||||||||||||||||||||||||||||||||||||||||||

Sbjct 227431 TCGTTCTTCTGACTTTATTAGTAGAGGGTGGTTGCTTTGGAATTCTGTAAGTAGCTCGTG 227490

Query 516851 CTTAGTGATCGTGCCCTTTTGGTCCCTTATCCTTGGCATCTGATTCTTCTTTCTTGAGCT 516910

||||||||||||||||||||||||||||||||||||||||||||||||||||||||||||

Sbjct 227491 CTTAGTGATCGTGCCCTTTTGGTCCCTTATCCTTGGCATCTGATTCTTCTTTCTTGAGCT 227550

Query 516911 CTCTCTTTAGTAGCTTTCTAGAAAGAAGATTTAGACCTTGTCCTCTTTGCTTTTTGAAAC 516970

||||||||||||||||||||||||||||||||||||||||||||||||||||||||||||

Sbjct 227551 CTCTCTTTAGTAGCTTTCTAGAAAGAAGATTTAGACCTTGTCCTCTTTGCTTTTTGAAAC 227610

Query 516971 CGAAACCTTTTCTATTTGAAGAAACATGTACCTTCGCCCGCTTAGGTATTCGTATCTTAC 517030

||||||||||||||||||||||||||||||||||||||||||||||||||||||||||||

Sbjct 227611 CGAAACCTTTTCTATTTGAAGAAACATGTACCTTCGCCCGCTTAGGTATTCGTATCTTAC 227670

Query 517031 TTCCCAAAAGGTCTTATAATCTTAACCACTTAAGCTGCACTTAGTGTATATCAATCAAGA 517090

||||||||||||||||||||||||||||||||||||||||||||||||||||||||||||

Sbjct 227671 TTCCCAAAAGGTCTTATAATCTTAACCACTTAAGCTGCACTTAGTGTATATCAATCAAGA 227730

Query 517091 AGTTCCTCGAGCGTACAAGGACCAGGTTTTCGAAACGAAAGACCCGAGCAATCGTTTTCT 517150

||||||||||||||||||||||||||||||||||||||||||||||||||||||||||||

Sbjct 227731 AGTTCCTCGAGCGTACAAGGACCAGGTTTTCGAAACGAAAGACCCGAGCAATCGTTTTCT 227790

Query 517151 CCGCGGGTATTTCAAAGCATAGCGCAGATAGGACTCAACGTGCTCATTAAGTATTCTGAG 517210

||||||||||||||||||||||||||||||||||||||||||||||||||||||||||||

Sbjct 227791 CCGCGGGTATTTCAAAGCATAGCGCAGATAGGACTCAACGTGCTCATTAAGTATTCTGAG 227850

Query 517211 GAAGTCGGACATGGTGTTTGGGCAGCCGCTTTCCTCCATCTGCTTGATAACATTCTGAGC 517270

||||||||||||||||||||||||||||||||||||||||||||||||||||||||||||

Sbjct 227851 GAAGTCGGACATGGTGTTTGGGCAGCCGCTTTCCTCCATCTGCTTGATAACATTCTGAGC 227910

Query 517271 AGTGAGTTTAGCCTGCCATTTCTTCATTGGGATGGTAACCTCGATGTGGTCTTCCTTGGT 517330

||||||||||||||||||||||||||||||||||||||||||||||||||||||||||||

Sbjct 227911 AGTGAGTTTAGCCTGCCATTTCTTCATTGGGATGGTAACCTCGATGTGGTCTTCCTTGGT 227970

Query 517331 CAATGACATAACTAACCCTAGCACTTGTGGATTCCCTGGCCCTGTTTTTACAATGAAATT 517390

||||||||||||||||||||||||||||||||||||||||||||||||||||||||||||

Sbjct 227971 CAATGACATAACTAACCCTAGCACTTGTGGATTCCCTGGCCCTGTTTTTACAATGAAATT 228030

Query 517391 AGGTCGGTAGATCCGCTTCCCAGTGAAGTCCAGTTTATAGAAGCCACAGAGGCTCTTTTT 517450

||||||||||||||||||||||||||||||||||||||||||||||||||||||||||||

Sbjct 228031 AGGTCGGTAGATCCGCTTCCCAGTGAAGTCCAGTTTATAGAAGCCACAGAGGCTCTTTTT 228090

Query 517451 CTTTCTCCTCAAGGCTTGCTTGGCATCGGGCGCTCTTACTGCTCGGCGTGGAAGACCGAA 517510

||||||||||||||||||||||||||||||||||||||||||||||||||||||||||||

Sbjct 228091 CTTTCTCCTCAAGGCTTGCTTGGCATCGGGCGCTCTTACTGCTCGGCGTGGAAGACCGAA 228150

Query 517511 AGAGAAGGGAAGGTTGGAAAAGGGGAGATCCCAGGTTACCCCTTTTTTCAGTTAGTTATG 517570

||||||||||||||||||||||||||||||||||||||||||||||||||||||||||||

Sbjct 228151 AGAGAAGGGAAGGTTGGAAAAGGGGAGATCCCAGGTTACCCCTTTTTTCAGTTAGTTATG 228210

Query 517571 TTGGGGTTAAGAGCAAGCTGAAGTAGTGGAATGATAAAAACTATTCTGGTAGGCGTCGTC 517630

||||||||||||||||||||||||||||||||||||||||||||||||||||||||||||

Sbjct 228211 TTGGGGTTAAGAGCAAGCTGAAGTAGTGGAATGATAAAAACTATTCTGGTAGGCGTCGTC 228270

Query 517631 AAAGCAGGGTTTTCTTCAGGAACAAATCCAAGCTAGCCCAACCCCAACCTATGTAGAGAG 517690

||||||||||||||||||||||||||||||||||||||||||||||||||||||||||||

Sbjct 228271 AAAGCAGGGTTTTCTTCAGGAACAAATCCAAGCTAGCCCAACCCCAACCTATGTAGAGAG 228330

Query 517691 GAGGCCACTGCTGCTAAAGAGTATGGGAACATATGTAGAGCTATAGAGAACTTCCAACGa 517750

||||||||||||||||||||||||||||||||||||||||||||||||||||||||||||

Sbjct 228331 GAGGCCACTGCTGCTAAAGAGTATGGGAACATATGTAGAGCTATAGAGAACTTCCAACGA 228390

Query 517751 aaaaaaGATAGATCGGAGTTCACTGCTTTCTTTTGGATAGAGTTCACTGCTTTCTTTTGA 517810

||||||||||||||||||||||||||||||||||||||||||||||||||||||||||||

Sbjct 228391 AAAAAAGATAGATCGGAGTTCACTGCTTTCTTTTGGATAGAGTTCACTGCTTTCTTTTGA 228450

Query 517811 AAGAGAGGGTGGAGCTGGGCTTGATGTTCTCAATGGGTTCAAGTTATTCTTTAAAGACGG 517870

||||||||||||||||||||||||||||||||||||||||||||||||||||||||||||

Sbjct 228451 AAGAGAGGGTGGAGCTGGGCTTGATGTTCTCAATGGGTTCAAGTTATTCTTTAAAGACGG 228510

Query 517871 TAGAATATTAAAAGAATTGAACGTAAGGACGATTGCACTCATCGGTCCCAAATCCAAATA 517930

||||||||||||||||||||||||||||||||||||||||||||||||||||||||||||

Sbjct 228511 TAGAATATTAAAAGAATTGAACGTAAGGACGATTGCACTCATCGGTCCCAAATCCAAATA 228570

Query 517931 AGTTGTGAGACTTTACCCCAGCTAGAAAGAGTGTCGCTGAACACCTGGGTATTTTTTAAG 517990

||||||||||||||||||||||||||||||||||||||||||||||||||||||||||||

Sbjct 228571 AGTTGTGAGACTTTACCCCAGCTAGAAAGAGTGTCGCTGAACACCTGGGTATTTTTTAAG 228630

Query 517991 GAAGCCTCTTCGTTCTCTTTGATCAAGAGAGCCCCCAAGGCGCGAAGCGTCCTGTTATCC 518050

||||||||||||||||||||||||||||||||||||||||||||||||||||||||||||

Sbjct 228631 GAAGCCTCTTCGTTCTCTTTGATCAAGAGAGCCCCCAAGGCGCGAAGCGTCCTGTTATCC 228690

Query 518051 TCGAGCAAATAGCGCTTGATCTTAGACTCTACTCTCCTATAGGCAAGTCGCAACTAAGCT 518110

||||||||||||||||||||||||||||||||||||||||||||||||||||||||||||

Sbjct 228691 TCGAGCAAATAGCGCTTGATCTTAGACTCTACTCTCCTATAGGCAAGTCGCAACTAAGCT 228750

Query 518111 AGGACTACTTTCTCAGTAGGCAGTTGTACATGACTCGTTGAGCGTGAGAACACTTGTTGC 518170

||||||||||||||||||||||||||||||||||||||||||||||||||||||||||||

Sbjct 228751 AGGACTACTTTCTCAGTAGGCAGTTGTACATGACTCGTTGAGCGTGAGAACACTTGTTGC 228810

Query 518171 TTGTCTGGCCAGAAGGGAAGGGCCTCAGAGAAGAGATGAGAAGAAGCTCACGCTTTTCAA 518230

||||||||||||||||||||||||||||||||||||||||||||||||||||||||||||

Sbjct 228811 TTGTCTGGCCAGAAGGGAAGGGCCTCAGAGAAGAGATGAGAAGAAGCTCACGCTTTTCAA 228870

Query 518231 CTCTACAGTACCTGAACTAGCAGCGTATTTGAAATTATGATGTTGTGGGGGCGTGGAAAT 518290

||||||||||||||||||||||||||||||||||||||||||||||||||||||||||||

Sbjct 228871 CTCTACAGTACCTGAACTAGCAGCGTATTTGAAATTATGATGTTGTGGGGGCGTGGAAAT 228930

Query 518291 GTCGAGAGATGGTGATGGATAATACCAGGACGAATTGTAGCCGGCCGCTTTAAAAACAGC 518350

||||||||||||||||||||||||||||||||||||||||||||||||||||||||||||

Sbjct 228931 GTCGAGAGATGGTGATGGATAATACCAGGACGAATTGTAGCCGGCCGCTTTAAAAACAGC 228990

Query 518351 ACTCAATGTCTCTTTTAAATAAATAAATACCGAGTTGCTTATTCTCACCTTTTTCCACCA 518410

||||||||||||||||||||||||||||||||||||||||||||||||||||||||||||

Sbjct 228991 ACTCAATGTCTCTTTTAAATAAATAAATACCGAGTTGCTTATTCTCACCTTTTTCCACCA 229050

Query 518411 ACCGTGCCGACGAGCCTTCGATTGAGCCTGAACTATCCTACTTTCTGAACTGAACTGCCT 518470

||||||||||||||||||||||||||||||||||||||||||||||||||||||||||||

Sbjct 229051 ACCGTGCCGACGAGCCTTCGATTGAGCCTGAACTATCCTACTTTCTGAACTGAACTGCCT 229110

Query 518471 ATACTACCCAAGCCTTGTCTTTGAATGCCAGCTTTTTCTTATGAAATGCAAAGCAAGTTC 518530

||||||||||||||||||||||||||||||||||||||||||||||||||||||||||||

Sbjct 229111 ATACTACCCAAGCCTTGTCTTTGAATGCCAGCTTTTTCTTATGAAATGCAAAGCAAGTTC 229170

Query 518531 TTTCCAATGCTACTTCCAAAAAGCCAGTGCACGGATTTGATCTCTTGACTTTTATTAGCT 518590

||||||||||||||||||||||||||||||||||||||||||||||||||||||||||||

Sbjct 229171 TTTCCAATGCTACTTCCAAAAAGCCAGTGCACGGATTTGATCTCTTGACTTTTATTAGCT 229230

Query 518591 CCTGAGATAGATAAAGCTACGTGCTCGCTAACTCTATTCGTCCTGGCAAAGCGGCGAACC 518650

||||||||||||||||||||||||||||||||||||||||||||||||||||||||||||

Sbjct 229231 CCTGAGATAGATAAAGCTACGTGCTCGCTAACTCTATTCGTCCTGGCAAAGCGGCGAACC 229290

Query 518651 TTGATCTCAGCCTGCCTAATGAAGAAAGAGAAGTCGATCTAGAGAAGCTTCTTAAGTAAG 518710

||||||||||||||||||||||||||||||||||||||||||||||||||||||||||||

Sbjct 229291 TTGATCTCAGCCTGCCTAATGAAGAAAGAGAAGTCGATCTAGAGAAGCTTCTTAAGTAAG 229350

Query 518711 AATAAGATGGAAAAAGAGTATAAATTTGAAAGTTCATTCGCTTGGAAGCTAGCTTCATCT 518770

|||||||||| ||||||||| |||||||||||||||||||||||||||||||||||||||

Sbjct 229351 AATAAGATGGCAAAAGAGTAGAAATTTGAAAGTTCATTCGCTTGGAAGCTAGCTTCATCT 229410

Query 518771 GGAGCATACCCTCAGGGAGGGGTATCACAATCTTCTGTACCTATATGGAAATCGTAGCAA 518830

||||||||||||||||||||||||||||||||||||||||||||||||||||||||||||

Sbjct 229411 GGAGCATACCCTCAGGGAGGGGTATCACAATCTTCTGTACCTATATGGAAATCGTAGCAA 229470

Query 518831 GGCTAGCTTGTAACATCGAAGAGCAGGCATTCCCAATTAGTTGAAACGTTGAAAAAACCA 518890

|||||||||||||||||||||||||||||||||||||||||||||| |||||||||||||

Sbjct 229471 GGCTAGCTTGTAACATCGAAGAGCAGGCATTCCCAATTAGTTGAAATGTTGAAAAAACCA 229530

Query 518891 TAGAGCTGGCTTATTGTTCCTTTTTGCATTATATATTTCACGCCTTCTTGCTCATCTAGA 518950

||||||||||||||||||||||||||||||||||||||||||||||||||||||||||||

Sbjct 229531 TAGAGCTGGCTTATTGTTCCTTTTTGCATTATATATTTCACGCCTTCTTGCTCATCTAGA 229590

Query 518951 AACTCTCCCTCTCATATCTTGTGACAACTATTCCTGTCTCGGTTATCTGTGCTATACAAA 519010

||||||||||||||||||||||||||||||||||||||||||||||||||||||||||||

Sbjct 229591 AACTCTCCCTCTCATATCTTGTGACAACTATTCCTGTCTCGGTTATCTGTGCTATACAAA 229650

Query 519011 TGCCTCTTTGTAAGCCCGTGCAGACTGAAATGCATATGCCTCCCAAAGCGGATTAAGCAT 519070

||||||||||||||||||||||||||||||||||||||||||||||||||||||||||||

Sbjct 229651 TGCCTCTTTGTAAGCCCGTGCAGACTGAAATGCATATGCCTCCCAAAGCGGATTAAGCAT 229710

Query 519071 ATCCTCGCTCTCTCTACCCTTTCCTCTCTCATTCCAGAAAAGAAGTGCAATATGCGATCA 519130

||||||||||||||||||||||||||||||||||||||||||||||||||||||||||||

Sbjct 229711 ATCCTCGCTCTCTCTACCCTTTCCTCTCTCATTCCAGAAAAGAAGTGCAATATGCGATCA 229770

Query 519131 ATAAGCTTACGGATTGCTGCGCCCGCATCATAAGTAGTGCCGTAGGTTCAATCCCAACCA 519190

||||||||||||||||||||||||||||||||||||||||||||||||||||||||||||

Sbjct 229771 ATAAGCTTACGGATTGCTGCGCCCGCATCATAAGTAGTGCCGTAGGTTCAATCCCAACCA 229830

Query 519191 ATGCCCAGCTTCAGCAAATAGAAGAAGGGTTAACATCAATAATATGCGATCAAGCAAAGA 519250

||||||||||||||||||||||||||||||||||||||||||||||||||||||||||||

Sbjct 229831 ATGCCCAGCTTCAGCAAATAGAAGAAGGGTTAACATCAATAATATGCGATCAAGCAAAGA 229890

Query 519251 GTGAAGAAGCAGCAATACGTGTTTTGAGTTCTCTTTCTTTTTTCTTCTATAAAAGCAATA 519310

||||||||||||||||||||||||||||||||||||||||||||||||||||||||||||

Sbjct 229891 GTGAAGAAGCAGCAATACGTGTTTTGAGTTCTCTTTCTTTTTTCTTCTATAAAAGCAATA 229950

Query 519311 GAAAAGAAGAAAGAATTCCGTAGTGTGTATCAAGTTGTGTATTAACGAGATAGGCTGTAG 519370

||||||||||||||||||||||||||||||||||||||||||||||||||||||||||||

Sbjct 229951 GAAAAGAAGAAAGAATTCCGTAGTGTGTATCAAGTTGTGTATTAACGAGATAGGCTGTAG 230010

Query 519371 TACGTTATGTTCTTTCGCATGGTGCCTTTCTAGCTAGGTGCTTTGGTACAGCTAATTTCC 519430

||||||||||||||||||||||||||||||||||||||||||||||||||||||||||||

Sbjct 230011 TACGTTATGTTCTTTCGCATGGTGCCTTTCTAGCTAGGTGCTTTGGTACAGCTAATTTCC 230070

Query 519431 CGGAGCATATACGATAGAAAGATTTACACTTGAAAGTACGTACTGATGCTTTCTTGAGAA 519490

||||||||||||||||||||||||||||||||||||||||||||||||||||||||||||

Sbjct 230071 CGGAGCATATACGATAGAAAGATTTACACTTGAAAGTACGTACTGATGCTTTCTTGAGAA 230130

Query 519491 TATGGAAGGAGTACGGTTGTGATGCCTATTAGTCCTTGTCAGATCATACGTGCCAGCCAG 519550

||||||||||||||||||||||||||||||||||||||||||||||||||||||||||||

Sbjct 230131 TATGGAAGGAGTACGGTTGTGATGCCTATTAGTCCTTGTCAGATCATACGTGCCAGCCAG 230190

Query 519551 CAGCAAAGGGCTTGATAGCGACGGTTGCAAGACAAAAGATAATTGAAAGGTGAACTCCGT 519610

||||||||||||||||||||||||||||||||||||||||||||||||||||||||||||

Sbjct 230191 CAGCAAAGGGCTTGATAGCGACGGTTGCAAGACAAAAGATAATTGAAAGGTGAACTCCGT 230250

Query 519611 AGGAAGAAGTGGAATTTCTTCAAGCAGAAGCTTATGCTTGCAAGTTCTCGGGTAAGGACT 519670

||||||||||||||||||||||||||||||||||||||||||||||||||||||||||||

Sbjct 230251 AGGAAGAAGTGGAATTTCTTCAAGCAGAAGCTTATGCTTGCAAGTTCTCGGGTAAGGACT 230310

Query 519671 TTCTTGTGTAGttttttttATTGACTTATAAAGAAAGGAACTCTGGAGGCGAAAGACttt 519730

||||||||||||||||||||||||||||||||||||||||||||||||||||||||||||

Sbjct 230311 TTCTTGTGTAGTTTTTTTTATTGACTTATAAAGAAAGGAACTCTGGAGGCGAAAGACTTT 230370

Query 519731 ttttttACTTTTAACACGTGTCAGCTAGTGAAAGAAAGGTATCTTGCCAAACTCTATGGA 519790

||||||||||||||||||||||||||||||||||||||||||||||||||||||||||||

Sbjct 230371 TTTTTTACTTTTAACACGTGTCAGCTAGTGAAAGAAAGGTATCTTGCCAAACTCTATGGA 230430

Query 519791 ATCTTTGATCCGGGTGCTCCGATATAGAGGTTGTTGGATATCCTCATCCTGTAATCGCCA 519850

||||||||||||||||||||||||||||||||||||||||||||||||||||||||||||

Sbjct 230431 ATCTTTGATCCGGGTGCTCCGATATAGAGGTTGTTGGATATCCTCATCCTGTAATCGCCA 230490

Query 519851 TAGTTCGACTATAGAACTCTCCAAGAAACTATGATTCTGGATAAGATAGGCATCCGGAGC 519910

||||||||||||||||||||||||||||||||||||||||||||||||||||||||||||

Sbjct 230491 TAGTTCGACTATAGAACTCTCCAAGAAACTATGATTCTGGATAAGATAGGCATCCGGAGC 230550

Query 519911 ATTAATAAGATAAGATATCCGGTCTATGATAGCATGAATAAGGAGAGGCATCGTTCGTGT 519970

||||||||||||||||||||||||||||||||||||||||||||||||||||||||||||

Sbjct 230551 ATTAATAAGATAAGATATCCGGTCTATGATAGCATGAATAAGGAGAGGCATCGTTCGTGT 230610

Query 519971 GAGTCTCAGAATCCGGATTTTAGCAAAGGAAGCCATCAAGCTTCGCCCTTTCTTTAAGCA 520030

||||||||||||||||||||||||||||||||||||||||||||||||||||||||||||

Sbjct 230611 GAGTCTCAGAATCCGGATTTTAGCAAAGGAAGCCATCAAGCTTCGCCCTTTCTTTAAGCA 230670

Query 520031 TATCAAATATATTATATATGGAATCCATATCACGTGGCGTGGTGAGTGATGTGCTTTCCT 520090

||| ||||||||||||||||||||||||||||||||||||||||||||||||||||||||

Sbjct 230671 TATAAAATATATTATATATGGAATCCATATCACGTGGCGTGGTGAGTGATGTGCTTTCCT 230730

Query 520091 TGGATTCGCTTTTTTGCCTAAGCCTAAAGGCAAGCTCCTAAGATTCCTCACCCAAGACCA 520150

||||||||||||||||||||||||||||||||||||||||||||||||||||||||||||

Sbjct 230731 TGGATTCGCTTTTTTGCCTAAGCCTAAAGGCAAGCTCCTAAGATTCCTCACCCAAGACCA 230790

Query 520151 TGAAGCCTAGCTTCTTTCTAAACCCAAGCGCGT-----AGCTAAGGATGCCTGGTAAAGA 520205

||||||||||||||||||||||||||||||||| ||||||||||||||||||||||

Sbjct 230791 TGAAGCCTAGCTTCTTTCTAAACCCAAGCGCGTAGCTAAGCTAAGGATGCCTGGTAAAGA 230850

Query 520206 ATCCTGTAAGGAGGGAGGCTTAATTGTTGTGCTTCTTTTGAGAGTTGCTATATGTACTTT 520265

||||||||||||||||||||||||||||||||||||||||||||||||||||||||||||

Sbjct 230851 ATCCTGTAAGGAGGGAGGCTTAATTGTTGTGCTTCTTTTGAGAGTTGCTATATGTACTTT 230910

Query 520266 TACCAACAAATACTATGTTCAAGCCATTTTTTACCTGCCCATTTTTAGAGGTATGCTACC 520325

||||||||||||||||||||||||||||||||||||||||||||||||||||||||||||

Sbjct 230911 TACCAACAAATACTATGTTCAAGCCATTTTTTACCTGCCCATTTTTAGAGGTATGCTACC 230970

Query 520326 CTATTTTTTGGAAGTAGGGCGCAGCAAAGTaaaaaaaaGGTAATTAGACTAAATAGTTAT 520385

| ||||||||||||||||||||||||||||||||||||||||||||

Sbjct 230971 C---------------GGGCGCAGCAAAGTAAAAAAAAGGTAATTAGACTAAATAGTTAT 231015

Query 520386 TACGAACGAGTTCTATTCGATATTTACCGGTACTGGTCTTTACATGTATGGACAGGAACT 520445

||||||||||||||||||||||||||||||||||||||||||||||||||||||||||||

Sbjct 231016 TACGAACGAGTTCTATTCGATATTTACCGGTACTGGTCTTTACATGTATGGACAGGAACT 231075

Query 520446 GGATTTGAAACTTCAGGGGGAAGCTGCCAAAAACTTGTATTGGTTAATGAGGATTTGACA 520505

||||||||||||||||||||||||||||||||||||||||||||||||||||||||||||

Sbjct 231076 GGATTTGAAACTTCAGGGGGAAGCTGCCAAAAACTTGTATTGGTTAATGAGGATTTGACA 231135

Query 520506 CATTAATGAGTTAAGAATTTCAACCGCTACGCGCCTTTAACTTCACTTCCTAACAACGAG 520565

||||||||||||||||||||||||||||||||||||||||||||||||||||||||||||

Sbjct 231136 CATTAATGAGTTAAGAATTTCAACCGCTACGCGCCTTTAACTTCACTTCCTAACAACGAG 231195

Query 520566 GTTCCTGGTCAACGAAAGAAAGCCATCACATACCTCCTTACGTTCTACCAAGGCCAAGGA 520625

||||||||||||||||||||||||||||||||||||||||||||||||||||||||||||

Sbjct 231196 GTTCCTGGTCAACGAAAGAAAGCCATCACATACCTCCTTACGTTCTACCAAGGCCAAGGA 231255

Query 520626 ATGAATAGGCTCGGGTAATTCTTTCACCAAGAAAGCGGGAAAGAATGGATAGTATCGAGA 520685

||||||||||||||||||||||||||||||||||||||||||||||||||||||||||||

Sbjct 231256 ATGAATAGGCTCGGGTAATTCTTTCACCAAGAAAGCGGGAAAGAATGGATAGTATCGAGA 231315

Query 520686 ACGGCTGTTAAGTTAAGTAGGGCTAAATAGGTCTAGTTTTCTGCCTTACAAGGGGATAGC 520745

||||||||||||||||||||||||||||||||||||||||||||||||||||||||||||

Sbjct 231316 ACGGCTGTTAAGTTAAGTAGGGCTAAATAGGTCTAGTTTTCTGCCTTACAAGGGGATAGC 231375

Query 520746 TAAAGGGACTCCAGGTACCAGACTGAAATCACGTACTATCTTCTCACTGCCCCCATCCGA 520805

||||||||||||||||||||||||||||||||||||||||||||||||||||||||||||

Sbjct 231376 TAAAGGGACTCCAGGTACCAGACTGAAATCACGTACTATCTTCTCACTGCCCCCATCCGA 231435

Query 520806 GTCATACCAGAGTTGGTAAGTAAACTTAATCTTTCAAAGGGACGCTGGGAACTAGCCAAA 520865

||||||||||||||||||||||||||||||||||||||||||||||||||||||||||||

Sbjct 231436 GTCATACCAGAGTTGGTAAGTAAACTTAATCTTTCAAAGGGACGCTGGGAACTAGCCAAA 231495

Query 520866 AGCAAGAGCTTTTCAGAAGAAGGAAGACCTTTCAGACTACCTCAATCAAAGTATGCGTCT 520925

||||||||||||||||||||||||||||||||||||||||||||||||||||||||||||

Sbjct 231496 AGCAAGAGCTTTTCAGAAGAAGGAAGACCTTTCAGACTACCTCAATCAAAGTATGCGTCT 231555

Query 520926 TCTTGATTGAAGGAAGAATATCTTATATAGTCTAGTTTTTTCATATATAGTCCAGTTTTG 520985

||||||||||||||||||||||||||||||||||||||||||||||||||||||||||||

Sbjct 231556 TCTTGATTGAAGGAAGAATATCTTATATAGTCTAGTTTTTTCATATATAGTCCAGTTTTG 231615

Query 520986 GTGAGTTGGTGTTCCTCTCTCATGTTCACTTTCTCGACCCGAGAAAAGACTAAGAAATTG 521045

||||||||||||||||||||||||||||||||||||||||||||||||||||||||||||

Sbjct 231616 GTGAGTTGGTGTTCCTCTCTCATGTTCACTTTCTCGACCCGAGAAAAGACTAAGAAATTG 231675

Query 521046 AAATCAGACTCATAGTAGGACTATTGATGTCCACTATAAGAGGATGAACAAGTACTCGAG 521105

||||||||||||||||||||||||||||||||||||||||||||||||||||||||||||

Sbjct 231676 AAATCAGACTCATAGTAGGACTATTGATGTCCACTATAAGAGGATGAACAAGTACTCGAG 231735

Query 521106 GATTATGGTCAGAGATAGGAGAATGAAAAGAGATGATCCCGAGAAAAGGAAATGAAAAGA 521165

||||||||||||||||||||||||||||||||||||||||||||||||||||||||||||

Sbjct 231736 GATTATGGTCAGAGATAGGAGAATGAAAAGAGATGATCCCGAGAAAAGGAAATGAAAAGA 231795

Query 521166 CAGGAAAGGATCTTTGTTTTAATTGTCTTTCACACCTAGGAGAATAAGAAGAATAAGACA 521225

||||||||||||||||||||||||||||||||||||||||||||||||||||||||||||

Sbjct 231796 CAGGAAAGGATCTTTGTTTTAATTGTCTTTCACACCTAGGAGAATAAGAAGAATAAGACA 231855

Query 521226 TATTATTGCTTTGCTGGATCTTTTTAGTTGAACTGAGTAGAGCAACATAATGAAAAACTG 521285

||||||||||||||||||||||||||||||||||||||||||||||||||||||||||||

Sbjct 231856 TATTATTGCTTTGCTGGATCTTTTTAGTTGAACTGAGTAGAGCAACATAATGAAAAACTG 231915

Query 521286 GCTTTCGTTACTACCTTCACTTCTGGTCCCTTCTCTATGTACGGTTATATGGTTTTCGTC 521345

||||||||||||||||||||||||||||||||||||||||||||||||||||||||||||

Sbjct 231916 GCTTTCGTTACTACCTTCACTTCTGGTCCCTTCTCTATGTACGGTTATATGGTTTTCGTC 231975

Query 521346 TTCTCTTCTCATCTTTCCATTCCGAGTGAGTGAGTTGAACTAGGGATGTTCACATAAAGT 521405

||||||||||||||||||||||||||||||||||||||||||||||||||||||||||||

Sbjct 231976 TTCTCTTCTCATCTTTCCATTCCGAGTGAGTGAGTTGAACTAGGGATGTTCACATAAAGT 232035

Query 521406 CTTGTCTAGCGGATGGATAGAATTTTACATGGCTTCTATTTAGTGGATACGCCCTCACCC 521465

||||||||||||||||||||||||| ||||||||||||||||||||||||||||||||||

Sbjct 232036 CTTGTCTAGCGGATGGATAGAATTTGACATGGCTTCTATTTAGTGGATACGCCCTCACCC 232095

Query 521466 GATACTAGGTGTATGATCGATTTCTAATGGCTTTTACCTATCCATTCACGTGCTTCATAG 521525

||||||||||||||||||||||||||||||||||||||||||||||||||||||||||||

Sbjct 232096 GATACTAGGTGTATGATCGATTTCTAATGGCTTTTACCTATCCATTCACGTGCTTCATAG 232155

Query 521526 TTCCTCTTTCTTGTTATAGTGTTTAGGAGTAGGAGTATGTCTGTGAAAAGAGGCAGACAT 521585

||||||||||||||||||||||||||||||||||||||||||||||||||||||||||||

Sbjct 232156 TTCCTCTTTCTTGTTATAGTGTTTAGGAGTAGGAGTATGTCTGTGAAAAGAGGCAGACAT 232215

Query 521586 AGCGAAAAGGGCAAAAGGGGGGAAAAAGTGAATGAAGGAAGAGATGCTATTTCTATATAT 521645

||||||||||||||||||||||||||||||||||||||||||||||||||||||||||||

Sbjct 232216 AGCGAAAAGGGCAAAAGGGGGGAAAAAGTGAATGAAGGAAGAGATGCTATTTCTATATAT 232275

Query 521646 TTTTGGTTTTATCTTTCTTTATTTGTGTCTTGGTTTGGTCCTCTGGTGCCTCTGCCCCGA 521705

||||||||||||||||||||||||||||||||||||||||||||||||||||||||||||

Sbjct 232276 TTTTGGTTTTATCTTTCTTTATTTGTGTCTTGGTTTGGTCCTCTGGTGCCTCTGCCCCGA 232335

Query 521706 CATGGAGCTCCTGTTGGACCTCGCTTCGGGACTCGCCGCTTGGGTTCACCAATTTTTCTA 521765

||||||||||||||||||||||||||||||||||||||||||||||||||||||||||||

Sbjct 232336 CATGGAGCTCCTGTTGGACCTCGCTTCGGGACTCGCCGCTTGGGTTCACCAATTTTTCTA 232395

Query 521766 CCAACTGGTAGATTTGGTGAAAGGCGCGTCTAAGGGTAAAGAGCCCATGTCTTTAGGCAG 521825

||||||||||||||||||||||||||||||||||||||||||||||||||||||||||||

Sbjct 232396 CCAACTGGTAGATTTGGTGAAAGGCGCGTCTAAGGGTAAAGAGCCCATGTCTTTAGGCAG 232455

Query 521826 CCCCCTGGAACAAGTCCCGTCCGCGGGCGGGGTGGGTATGAGCACTCCTTCGCCGATTAG 521885

||||||||||||||||||||||||||||||||||||||||||||||||||||||||||||

Sbjct 232456 CCCCCTGGAACAAGTCCCGTCCGCGGGCGGGGTGGGTATGAGCACTCCTTCGCCGATTAG 232515

Query 521886 AAATTCCGGCAAGGAGGCGGGGCAACACACTGAAACCCTGGGCACAACATCCTTAATGGA 521945

||||||||||||||||||||||||||||||||||||||||||||||||||||||||||||

Sbjct 232516 AAATTCCGGCAAGGAGGCGGGGCAACACACTGAAACCCTGGGCACAACATCCTTAATGGA 232575

Query 521946 TGGGCCGGGGCAAGGACAAGTGGAAGGGGGCCACCGCCACCTGAATCTTGATTTAAATGC 522005

|||||||||||||||||||||||||||||||||||||||||||||||||||||||||||

Sbjct 232576 AGGGCCGGGGCAAGGACAAGTGGAAGGGGGCCACCGCCACCTGAATCTTGATTTAAATGC 232635

Query 522006 ACCCCCTCCGGGTCCGGACAGCTTGTGTCCACGGGAATATCGCCTGGCCGAGAACAAGCT 522065

||||||||||||||||||||||||||||||||||||||||||||||||||||||||||||

Sbjct 232636 ACCCCCTCCGGGTCCGGACAGCTTGTGTCCACGGGAATATCGCCTGGCCGAGAACAAGCT 232695

Query 522066 CAAAGATATTCTCATGGGGATGAATAAAGCCCCTCGTTCCGACTTACTGCCTCATTACGA 522125

||||||||||||||||||||||||||||||||||||||||||||||||||||||||||||

Sbjct 232696 CAAAGATATTCTCATGGGGATGAATAAAGCCCCTCGTTCCGACTTACTGCCTCATTACGA 232755

Query 522126 AGAAGTAGAGCCAAGGCGGAGGGAAACCTATGTAAGGAGCAAAATCTCTGAATACCTGAA 522185

||||||||||||||||||||||||||||||||||||||||||||||||||||||||||||

Sbjct 232756 AGAAGTAGAGCCAAGGCGGAGGGAAACCTATGTAAGGAGCAAAATCTCTGAATACCTGAA 232815

Query 522186 GCAACACTCCCCGGAAGATATACAAACAAACGTGGGTGAGCTTCTGGTTATGAAGAAGAG 522245

||||||||||||||||||||||||||||||||||||||||||||||||||||||||||||

Sbjct 232816 GCAACACTCCCCGGAAGATATACAAACAAACGTGGGTGAGCTTCTGGTTATGAAGAAGAG 232875

Query 522246 CTCCTCCCTTTATAAGGGTTTTTAACAATACCTTCTCCAACAAAAGAACAGAGGAGAAGA 522305

|||||||||||||||||||||| |||||||||||||||||||||||||||||||||||||

Sbjct 232876 CTCCTCCCTTTATAAGGGTTTTGAACAATACCTTCTCCAACAAAAGAACAGAGGAGAAGA 232935

Query 522306 GTAGTGTTCCTTTCCGTCCGTGAGGTTCAAAGAAAGGCGGGAGGTTAGGTAGCAGGAGTC 522365

||||||||||||||||||||||||||||||||||||||||||||||||||||||||||||

Sbjct 232936 GTAGTGTTCCTTTCCGTCCGTGAGGTTCAAAGAAAGGCGGGAGGTTAGGTAGCAGGAGTC 232995

Query 522366 CTGGAAAAGAGAGTAGCTTCAGGGGAGCAGGGCCCAGTATTTGTTTTCCTTTGAATTACA 522425

||||||||||||||||||||||||||||||||||||||||||||||||||||||||||||

Sbjct 232996 CTGGAAAAGAGAGTAGCTTCAGGGGAGCAGGGCCCAGTATTTGTTTTCCTTTGAATTACA 233055

Query 522426 CTAATTGAATTGAATTTGGCACCGGAAACTCTTCTAGGAGAAGTTCGAATTCGTTCCCTT 522485

||||||||||||||||||||||||||||||||||||||||||||||||||||||||||||

Sbjct 233056 CTAATTGAATTGAATTTGGCACCGGAAACTCTTCTAGGAGAAGTTCGAATTCGTTCCCTT 233115

Query 522486 CGGATATTGAGCGGTCTTGTTTTTACATGGTTTACGTGCTACTGGTTCTCGGAAGAGCTA 522545

||||||||||||||||||||||||||||||||||||||||||||||||||||||||||||

Sbjct 233116 CGGATATTGAGCGGTCTTGTTTTTACATGGTTTACGTGCTACTGGTTCTCGGAAGAGCTA 233175

Query 522546 ATTTATTCATTAGCTAAACCCTTTCTAACCCTACCTTTGGACTCGTATTTTGTTTGTACA 522605

||||||||||||||||||||||||||||||||||||||||||||||||||||||||||||

Sbjct 233176 ATTTATTCATTAGCTAAACCCTTTCTAACCCTACCTTTGGACTCGTATTTTGTTTGTACA 233235

Query 522606 CAATTAACGGAGGCCTTCTCTACTTATCTTGCAACGTCTTCAATAGCATGCTCTTACTTC 522665

||||||||||||||||||||||||||||||||||||||||||||||||||||||||||||

Sbjct 233236 CAATTAACGGAGGCCTTCTCTACTTATCTTGCAACGTCTTCAATAGCATGCTCTTACTTC 233295

Query 522666 GTCTTTCCCTTCATCAGTTATCAAATTTGGTGCTTCTCGATACCCAGTTGCTATGGGGAA 522725

||||||||||||||||||||||||||||||||||||||||||||||||||||||||||||

Sbjct 233296 GTCTTTCCCTTCATCAGTTATCAAATTTGGTGCTTCTCGATACCCAGTTGCTATGGGGAA 233355

Query 522726 AAAAGGAGGAAATACAATCGATTCCTCTATTTCAGTGTTTCTTGCTTTTCCTTATTCCTG 522785

||||||||||||||||||||||||||||||||||||||||||||||||||||||||||||

Sbjct 233356 AAAAGGAGGAAATACAATCGATTCCTCTATTTCAGTGTTTCTTGCTTTTCCTTATTCCTG 233415

Query 522786 TTCCTAACTCTTTCCTGGGTAATTCCCAATGTTTGGCACTTTCTATACTTCATGGGTGAA 522845

||||||||||||||||||||||||||||||||||||||||||||||||||||||||||||

Sbjct 233416 TTCCTAACTCTTTCCTGGGTAATTCCCAATGTTTGGCACTTTCTATACTTCATGGGTGAA 233475

Query 522846 ACATCAACAAATTCGCTAATGATAAAGTTACAACCTAAGATCTATGACTATATTATGTTC 522905

||||||||||||||||||||||||||||||||||||||||||||||||||||||||||||

Sbjct 233476 ACATCAACAAATTCGCTAATGATAAAGTTACAACCTAAGATCTATGACTATATTATGTTC 233535

Query 522906 ACTCTTCGTATTTTGTTCATTCCATCGGTATGCTCCCAGGTACCTGTCATTGTGATCTGT 522965

||||||||||||||||||||||||||||||||||||||||||||||||||||||||||||

Sbjct 233536 ACTCTTCGTATTTTGTTCATTCCATCGGTATGCTCCCAGGTACCTGTCATTGTGATCTGT 233595

Query 522966 TTGCCAGAACCAAGGGGTCTCTCTGTGGAAATGCTCACGAGCAATCGTCGTTTTTTGATG 523025

||||||||||||||||||||||||||||||||||||||||||||||||||||||||||||

Sbjct 233596 TTGCCAGAACCAAGGGGTCTCTCTGTGGAAATGCTCACGAGCAATCGTCGTTTTTTGATG 233655

Query 523026 GTTTTTTCACTGATCACAGCTGCTCTTTCCACACCTCCGGATATCTGGTGCCAAATTGTC 523085

||||||||||||||||||||||||||||||||||||||||||||||||||||||||||||

Sbjct 233656 GTTTTTTCACTGATCACAGCTGCTCTTTCCACACCTCCGGATATCTGGTGCCAAATTGTC 233715

Query 523086 GCCTCTTTACTTATTTATTCAATCATAGAGCTTGCTATCTTTGTGGCATTGATTATAAAA 523145

||||||||||||||||||||||||||||||||||||||||||||||||||||||||||||

Sbjct 233716 GCCTCTTTACTTATTTATTCAATCATAGAGCTTGCTATCTTTGTGGCATTGATTATAAAA 233775

Query 523146 GTTCGTGAAGAGGGCTGGACGAAACGAATGAGGGAAAGCGGTTCGATCGAGaaaaaaaGA 523205

||||||||||||||||||||||||||||||||||||||||||||||||||||||||||||

Sbjct 233776 GTTCGTGAAGAGGGCTGGACGAAACGAATGAGGGAAAGCGGTTCGATCGAGAAAAAAAGA 233835

Query 523206 AGAGTAAAGTAGAACCCACCAAAAGTAACTAAAAAGTAATTCCATTAATTCTCGTCGATA 523265

||||||||||||||||||||||||||||||||||||||||||||||||||||||||||||

Sbjct 233836 AGAGTAAAGTAGAACCCACCAAAAGTAACTAAAAAGTAATTCCATTAATTCTCGTCGATA 233895

Query 523266 AAAAAGAGCAGACTCCTGACTGGAATGAGAGGGCGTAGCGTACACTCTGGAATGCTCAAA 523325

||||||||||||||||||||||||||||||||||||||||||||||||||||||||||||

Sbjct 233896 AAAAAGAGCAGACTCCTGACTGGAATGAGAGGGCGTAGCGTACACTCTGGAATGCTCAAA 233955

Query 523326 CCTTGAGAAGCAAGTTATTAATGAATGGTTCGAAGCGACAAATCACATAAGGTAAAGAGC 523385

||||||||||||||||||||||||||||||||||||||||||||||||||||||||||||

Sbjct 233956 CCTTGAGAAGCAAGTTATTAATGAATGGTTCGAAGCGACAAATCACATAAGGTAAAGAGC 234015

Query 523386 GCCTGCTTAATTGAAGGTAGTAGATAGAAGCAAGGGGTATTTCGGCTCTAATGAGCTAGC 523445

||||||||||||||||||||||||||||||||||||||||||||||||||||||||||||

Sbjct 234016 GCCTGCTTAATTGAAGGTAGTAGATAGAAGCAAGGGGTATTTCGGCTCTAATGAGCTAGC 234075

Query 523446 TCCTTTTTCCCTTTACGAGATCTTGACAGGAATGGTTGGAGAGAGAATGAATAGAGGGAG 523505

||||||||||||||||||||||||||||||||||||||||||||||||||||||||||||

Sbjct 234076 TCCTTTTTCCCTTTACGAGATCTTGACAGGAATGGTTGGAGAGAGAATGAATAGAGGGAG 234135

Query 523506 CaaaaaaaaGGCTTTGCTCCCCTCCCCTTGATTCAAATGAAGAAAGAAGGTTTGAAGTTT 523565

||||||||||||||||||||||||||||||||||||||||||||||||||||||||||||

Sbjct 234136 CAAAAAAAAGGCTTTGCTCCCCTCCCCTTGATTCAAATGAAGAAAGAAGGTTTGAAGTTT 234195

Query 523566 AGACCGCTCACAGTAGTTCTACCCATAGAAAAGATCATGAAAGAGGCGATCAGAACGGTA 523625

||||||||||||||||||||||||||||||||||||||||||||||||||||||||||||

Sbjct 234196 AGACCGCTCACAGTAGTTCTACCCATAGAAAAGATCATGAAAGAGGCGATCAGAACGGTA 234255

Query 523626 CCCGAATTCATTTACGATCCCGAGTTTCCAGACACATCGCACTTCCGCTCGGGTCGAGGT 523685

||||||||||||||||||||||||||||||||||||||||||||||||||||||||||||

Sbjct 234256 CCCGAATTCATTTACGATCCCGAGTTTCCAGACACATCGCACTTCCGCTCGGGTCGAGGT 234315

Query 523686 TGGCACTCGGCCCTCAGACGGATAAAAGAAGATTGGGGAACCTCTCGCTGGTTTTTGGAA 523745

||||||||||||||||||||||||||||||||||||||||||||||||||||||||||||

Sbjct 234316 TGGCACTCGGCCCTCAGACGGATAAAAGAAGATTGGGGAACCTCTCGCTGGTTTTTGGAA 234375

Query 523746 TTCGACATCAGGAAGTGTTTTCACACCATCGACCAAGACCGATTCATCTCCATCTTGAAG 523805

||||||||||||||||||||||||||||||||||||||||||||||||||||||||||||

Sbjct 234376 TTCGACATCAGGAAGTGTTTTCACACCATCGACCAAGACCGATTCATCTCCATCTTGAAG 234435

Query 523806 GAAGAGATCGACGATTCCAAGTTCTTTTACTCCACTCAGAAACTTTTTTATTCCGGACGA 523865

||||||||||||||||||||||||||||||||||||||||||||||||||||||||||||

Sbjct 234436 GAAGAGATCGACGATTCCAAGTTCTTTTACTCCACTCAGAAACTTTTTTATTCCGGACGA 234495

Query 523866 CCCGTAAGAGGTGGGAGGAGGGGCCCCTCCTCCGTCCCACACAGTGTACTACTCTCGGCC 523925

| ||||||||||||||||||||||||||||||||||||||||||||||||||||||||||

Sbjct 234496 CTCGTAAGAGGTGGGAGGAGGGGCCCCTCCTCCGTCCCACACAGTGTACTACTCTCGGCC 234555

Query 523926 CTACTAGGCAATATCTATCTACACAAGCTCGATCAGGAGATAGGGAGGATCCGAAAGAAG 523985

||||||||||||||||||||||||||||||||||||||||||||||||||||||||||||

Sbjct 234556 CTACTAGGCAATATCTATCTACACAAGCTCGATCAGGAGATAGGGAGGATCCGAAAGAAG 234615

Query 523986 CACGAAATTCCTCTTGTTCAGAGAATAAGATCGGTTCTCTTAAGGACAGGTCGTCGTATT 524045

||||||||||||||||||||||||||||||||||||||||||||||||||||||||||||

Sbjct 234616 CACGAAATTCCTCTTGTTCAGAGAATAAGATCGGTTCTCTTAAGGACAGGTCGTCGTATT 234675

Query 524046 GATGACCAAGAAAAGTATGGAGAAGAAGCAAGCTTCAATGCTCCACAAGACAACAGAGCC 524105

||||||||||||||||||||||||||||||||||||||||||||||||||||||||||||

Sbjct 234676 GATGACCAAGAAAAGTATGGAGAAGAAGCAAGCTTCAATGCTCCACAAGACAACAGAGCC 234735

Query 524106 TTCATTGTGGGGAGGGTAAAGAGCATCCAACGCAAACCAACCTTTCATTCCCTTGGATCG 524165

||||||||||||||||||||||||||||||||||||||||||||||||||||||||||||

Sbjct 234736 TTCATTGTGGGGAGGGTAAAGAGCATCCAACGCAAACCAACCTTTCATTCCCTTGGATCG 234795

Query 524166 TCGTGGCACACCTCCCCCACAAGCTCCCTCCGGCGAAGGGGAGACCAGAAAAAGCCTTCC 524225

||||||||||||||||||||||||||||||||||||||||||||||||||||||||||||

Sbjct 234796 TCGTGGCACACCTCCCCCACAAGCTCCCTCCGGCGAAGGGGAGACCAGAAAAAGCCTTCC 234855

Query 524226 TTTTTACCCTCTTCAGCGTCCCTTGTAGCCTTCCTGAACAAGCCCTCGAGCCTCCTTTGC 524285

||||||||||||||||||||||||||||||||||||||||||||||||||||||||||||

Sbjct 234856 TTTTTACCCTCTTCAGCGTCCCTTGTAGCCTTCCTGAACAAGCCCTCGAGCCTCCTTTGC 234915

Query 524286 ACCGCCTTCCTAATAGAAGCCGCTGGGTTGACCCCGAAGGCCGAATTCTATGGTAGAAAA 524345

||||||||||||||||||||||||||||||||||||||||||||||||||||||||||||

Sbjct 234916 ACCGCCTTCCTAATAGAAGCCGCTGGGTTGACCCCGAAGGCCGAATTCTATGGTAGAAAA 234975

Query 524346 GGTGGCTTAACTCATAATTTGGCCATGAGAGACCTTCTGCAGTATTGCAAAAGAAGGGGC 524405

||||||||||||||||||||||||||||||||||||||||||||||||||||||||||||

Sbjct 234976 GGTGGCTTAACTCATAATTTGGCCATGAGAGACCTTCTGCAGTATTGCAAAAGAAGGGGC 235035

Query 524406 CTGTTGATAGAGCTGGGCGGGGAGGCGCAACTTTTTATCAGCTCCTCAGAGAGAGGCCAG 524465

||||||||||||||||||||||||||||||||||||||||||||||||||||||||||||

Sbjct 235036 CTGTTGATAGAGCTGGGCGGGGAGGCGCAACTTTTTATCAGCTCCTCAGAGAGAGGCCAG 235095

Query 524466 GTCCGTAAGCCGGCCCCCTTTAAAAGCCATTCCTTCTTTATAAGGATTTTTTACGCGCGA 524525

||||||||||||||||||||||||||||||||||||||||||||||||||||||||||||

Sbjct 235096 GTCCGTAAGCCGGCCCCCTTTAAAAGCCATTCCTTCTTTATAAGGATTTTTTACGCGCGA 235155

Query 524526 TATGCCGACGACTTACTACTAGGAATCGTGGGTGCCGTATTTTTTCTCATAGAAATACAA 524585

||||||||||||||||||||||||||||||||||||||||||||||||||||||||||||

Sbjct 235156 TATGCCGACGACTTACTACTAGGAATCGTGGGTGCCGTATTTTTTCTCATAGAAATACAA 235215

Query 524586 AAACGTCTAACCCACTTCCTAAAAACCGGCCTTAACCTTTGGGTGGGCTCCGCAGGATCA 524645

||||||||||||||||||||||||||||||||||||||||||||||||||||||||||||

Sbjct 235216 AAACGTCTAACCCACTTCCTAAAAACCGGCCTTAACCTTTGGGTGGGCTCCGCAGGATCA 235275

Query 524646 ACAACCATAGCTGCGCGGAGTAAGGTAGAATTCCTCGGTACGGTCATTCGGGAAGTACCT 524705

||||||||||||||||||||||||||||||||||||||||||||||||||||||||||||

Sbjct 235276 ACAACCATAGCTGCGCGGAGTAAGGTAGAATTCCTCGGTACGGTCATTCGGGAAGTACCT 235335

Query 524706 CCGAGGACGACTCCCATCAAATTCTTGCGAGAGCTGGAGAAGCGTCTACGGGTAAAGCAC 524765

||||||||||||||||||||||||||||||||||||||||||||||||||||||||||||

Sbjct 235336 CCGAGGACGACTCCCATCAAATTCTTGCGAGAGCTGGAGAAGCGTCTACGGGTAAAGCAC 235395

Query 524766 CGTATCCAGATAACTGCTTGCCACCTACGCTCTGCCATCCATTCCAAGTTTAGGGACCTA 524825

||||||||||||||||||||||||||||||||||||||||||||||||||||||||||||

Sbjct 235396 CGTATCCAGATAACTGCTTGCCACCTACGCTCTGCCATCCATTCCAAGTTTAGGGACCTA 235455

Query 524826 GGAACTAGTATCCCGATCAAAGAGCTTACGAAGGAGATGAGCGGAAGAGGTCGTTTACTG 524885

||||||||||||||||||||||||||||||||||||||||||||||||||||||||||||

Sbjct 235456 GGAACTAGTATCCCGATCAAAGAGCTTACGAAGGAGATGAGCGGAAGAGGTCGTTTACTG 235515

Query 524886 GATGCGGTTCAACTAGCCGATACTCTTGAAAAAGAAGGACTCAGAAGTGCCCAAGTGAAC 524945

||||||||||||||||||||||||||||||||||||||||||||||||||||||||||||

Sbjct 235516 GATGCGGTTCAACTAGCCGATACTCTTGAAAAAGAAGGACTCAGAAGTGCCCAAGTGAAC 235575

Query 524946 GTATTCTGGGAAACCCTCAAACACATCCGGCAAGGAGCAAGGGCGATCTCTTTGTTGCAT 525005

||||||||||||||||||||||||||||||||||||||||||||||||||||||||||||

Sbjct 235576 GTATTCTGGGAAACCCTCAAACACATCCGGCAAGGAGCAAGGGCGATCTCTTTGTTGCAT 235635

Query 525006 AGCTCAGGTCGAAGCAAGGTGCCTTTGGACAAAAGGCAGTCGAAGGCTATGAGTTTCTTT 525065

||||||||||||||||||||||||||||||||||||||||||||||||||||||||||||

Sbjct 235636 AGCTCAGGTCGAAGCAAGGTGCCTTTGGACAAAAGGCAGTCGAAGGCTATGAGTTTCTTT 235695

Query 525066 CTAAAAGAATTGGAGCCGGGCCGGAAGGCGGCGGGGAAAGGAAGGGGGCACTGGGCGGGA 525125

||||||||||||||||||||||||||||||||||||||||||||||||||||||||||||

Sbjct 235696 CTAAAAGAATTGGAGCCGGGCCGGAAGGCGGCGGGGAAAGGAAGGGGGCACTGGGCGGGA 235755

Query 525126 TCGTTAAGCAGCGAATTCCCCATACAGATAGAGGCGCCTATAAAAAAGATACTCCGAAGG 525185

||||||||||||||||||||||||||||||||||||||||||||||||||||||||||||

Sbjct 235756 TCGTTAAGCAGCGAATTCCCCATACAGATAGAGGCGCCTATAAAAAAGATACTCCGAAGG 235815

Query 525186 CTTCGAGATCGAGGTATCATTAGCCGAAAAAGACCCAGGCCAATCCACGTGGCCTCTTTG 525245

||||||||||||||||||||||||||||||||||||||||||||||||||||||||||||

Sbjct 235816 CTTCGAGATCGAGGTATCATTAGCCGAAAAAGACCCAGGCCAATCCACGTGGCCTCTTTG 235875

Query 525246 ACCAACGTAAGCGACGGAGACATAGTAAATCGGTTCGCGGGCATCGCGATAAGTCTTTTG 525305

||||||||||||||||||||||||||||||||||||||||||||||||||||||||||||

Sbjct 235876 ACCAACGTAAGCGACGGAGACATAGTAAATCGGTTCGCGGGCATCGCGATAAGTCTTTTG 235935

Query 525306 TCTTACTACAGGTGCTGCGACAACCTTTACCAAGTCCGAACGATTGTCGACTACCAGATC 525365

||||||||||||||||||||||||||||||||||||||||||||||||||||||||||||

Sbjct 235936 TCTTACTACAGGTGCTGCGACAACCTTTACCAAGTCCGAACGATTGTCGACTACCAGATC 235995

Query 525366 CGCTGGTCCGCTATATTCACCCTAGCCCACAAGCACAAATCTTCGGCGCGTCATATAATC 525425

||||||||||||||||||||||||||||||||||||||||||||||||||||||||||||

Sbjct 235996 CGCTGGTCCGCTATATTCACCCTAGCCCACAAGCACAAATCTTCGGCGCGTCATATAATC 236055

Query 525426 CCAAAGTACCCCAAAGACTTAAAAATAGTTAATAAAAAAGGTTGTAATACTCTTGCGGAG 525485

||||||||||||||||||||||||||||||||||||||||||||||| ||||||||||||

Sbjct 236056 CCAAAGTACCCCAAAGACTTAAAAATAGTTAATAAAAAAGGTTGTAAGACTCTTGCGGAG 236115

Query 525486 TTCCCAAACAACATAGAGCTTGGGAAGCTCGGACCCGGTCAAGATTAAAACAACGACTTG 525545

||||||||||||||||||||||||||||||||||||||||||||||||||||||||||||

Sbjct 236116 TTCCCAAACAACATAGAGCTTGGGAAGCTCGGACCCGGTCAAGATTAAAACAACGACTTG 236175

Query 525546 GaaaaaaaCTATCTAGACGGTAGGCGGGCGAGGAGCGGGAGGGTGGGCCTCGCCAATTTG 525605

||||||||||||||||||||||||||||||||||||||||||||||||||||||||||||

Sbjct 236176 GAAAAAAACTATCTAGACGGTAGGCGGGCGAGGAGCGGGAGGGTGGGCCTCGCCAATTTG 236235

Query 525606 TGTCTTTCTTTGGGAAAATGAAATGTGAAAATATCATATGATAAGGCGCGCGCGCAGCGA 525665

||||||||||||||||||||||||||||||||||||||||||||||||||||||||||||

Sbjct 236236 TGTCTTTCTTTGGGAAAATGAAATGTGAAAATATCATATGATAAGGCGCGCGCGCAGCGA 236295

Query 525666 TGATGGTGAAACGGAAAAGGGTACTTTGATGTGTGAGCAAGAACTAGTGTGGGCAAAGGG 525725

||||||||||||||||||||||||||||||||||||||||||||||||||||||||||||

Sbjct 236296 TGATGGTGAAACGGAAAAGGGTACTTTGATGTGTGAGCAAGAACTAGTGTGGGCAAAGGG 236355

Query 525726 GGAGTTTTTCGTGCGGGATGTGAAATGTAAAGTAAAGCACTTCTTTCATAATCCGGGCCC 525785

||||||||||||||||||||||||||||||||||||||||||||||||||||||||||||

Sbjct 236356 GGAGTTTTTCGTGCGGGATGTGAAATGTAAAGTAAAGCACTTCTTTCATAATCCGGGCCC 236415

Query 525786 CGGCTTTGAGTGAATGAACTCGCGTTCTGGCTCGATCCTTCGCTTGCTGGGATCGATTTG 525845

||||||||||||||||||||||||||||||||||||||||||||||||||||||||||||

Sbjct 236416 CGGCTTTGAGTGAATGAACTCGCGTTCTGGCTCGATCCTTCGCTTGCTGGGATCGATTTG 236475

Query 525846 GTTTACGTATATATATGAGGTTGATAAAAAGGCTAAAGGGGGGAGAGCACTTAGACATTT 525905

||||||||||||||||||||||||||||||||||||||||||||||||||||||||||||

Sbjct 236476 GTTTACGTATATATATGAGGTTGATAAAAAGGCTAAAGGGGGGAGAGCACTTAGACATTT 236535

Query 525906 TACTTTGAGTACAGGTCCGCAGGGCGTATTCttttttttttCATCGAGGGGAGGGATTGC 525965

||||||||||||||||||||||||||||||||||||||||||||||||||||||||||||

Sbjct 236536 TACTTTGAGTACAGGTCCGCAGGGCGTATTCTTTTTTTTTTCATCGAGGGGAGGGATTGC 236595

Query 525966 AGCGAAAAATTGACGTCTGTCTATTGGCATTTTATGTAGAAAGGATCGAATATGACCCTC 526025

||||||||||||||||||||||||||||||||||||||||||||||||||||||||||||

Sbjct 236596 AGCGAAAAATTGACGTCTGTCTATTGGCATTTTATGTAGAAAGGATCGAATATGACCCTC 236655

Query 526026 ATCGTTCTTCTAGGATCGCTCAAGATGGATCGAAGGGGTGCAGCTACGCCGGAGGAATTT 526085

||||||||||||||||||||||||||||||||||||||||||||||||||||||||||||

Sbjct 236656 ATCGTTCTTCTAGGATCGCTCAAGATGGATCGAAGGGGTGCAGCTACGCCGGAGGAATTT 236715

Query 526086 CCAGCCGATAGAAGAGTTTGCCCCTACGCGTAAGATCCTCGAATCAGGCCACCATCGCTA 526145

||||||||||||||||||||||||||||||||||||||||||||||||||||||||||||

Sbjct 236716 CCAGCCGATAGAAGAGTTTGCCCCTACGCGTAAGATCCTCGAATCAGGCCACCATCGCTA 236775

Query 526146 CGCGCCTTTGTTCGtttttttCTCTGCCCGTGGATCGATCAAAGAAAGCTAGCTTGCTCC 526205

||||||||||||||||||||||||||||||||||||||||||||||||||||||||||||

Sbjct 236776 CGCGCCTTTGTTCGTTTTTTTCTCTGCCCGTGGATCGATCAAAGAAAGCTAGCTTGCTCC 236835

Query 526206 CAAGCCAAATGCGTGCTTACGCTTTATGTAGTGGGCGGCCTTCCTATTTTCATGCCTCTA 526265

||||||||||||||||||||||||||||||||||||||||||||||||||||||||||||

Sbjct 236836 CAAGCCAAATGCGTGCTTACGCTTTATGTAGTGGGCGGCCTTCCTATTTTCATGCCTCTA 236895

Query 526266 GAAGCTTCTACAAAGCTTTGCTTCCGGCCGGTAGAAGCTAGTCGCTTCGCTTGCCTGCCA 526325

||||||||||||||||||||||| |||||||||||||||||||||||||||||||||

Sbjct 236896 GAAGCTTCTACAAAGCTTTGCTT----CCGGTAGAAGCTAGTCGCTTCGCTTGCCTGCCA 236951

Query 526326 AGCAGCCGCCTATAGGTATAGGGGAAGGGCCGAAGGATGGAGCGTGCAGAGTCGATCGTG 526385

||||||||||||||||||||||||||||||||||||||||||||||||||||||||||||

Sbjct 236952 AGCAGCCGCCTATAGGTATAGGGGAAGGGCCGAAGGATGGAGCGTGCAGAGTCGATCGTG 237011

Query 526386 CACCTGTAACTTTTCTATTAGCCAGTCATCAATGTTCCGCGTTGGTGATGAATTGTTATT 526445

||||||||||||||||||||||||||||||||||||||||||||||||||||||||||||

Sbjct 237012 CACCTGTAACTTTTCTATTAGCCAGTCATCAATGTTCCGCGTTGGTGATGAATTGTTATT 237071

Query 526446 TCTCCAAACCTTCTAAAAACGGCTTTTTGCGACCGACCTGCCCAGAATGCACATACCTTC 526505

||||||||||||||||||||||||||||||||||||||||||||||||||||||||||||

Sbjct 237072 TCTCCAAACCTTCTAAAAACGGCTTTTTGCGACCGACCTGCCCAGAATGCACATACCTTC 237131

Query 526506 GGTTCCAAGACCTGCACAGAGAATAAAAGCCGGGTTGAATGAAGAGTGAAGTAAGCTGGC 526565

||||||||||||||||||||||||||||||||||||||||||||||||||||||||||||

Sbjct 237132 GGTTCCAAGACCTGCACAGAGAATAAAAGCCGGGTTGAATGAAGAGTGAAGTAAGCTGGC 237191

Query 526566 TGCTTCTTTGCCACGCCCCCCGGCCGGAGATACTTGATATCAATTAAAAAGTCTAAAATT 526625

|||||||||||||||||||||||||||||||||||||||||||| |||||||||||||||

Sbjct 237192 TGCTTCTTTGCCACGCCCCCCGGCCGGAGATACTTGATATCAATGAAAAAGTCTAAAATT 237251

Query 526626 GCATACCATTAGCTTATATACGTCTGGGAACATTGGCACATGATATTGAATGTCATCCGG 526685

||||||||||||||||||||||||||||||||||||||||||||||||||||||||||||

Sbjct 237252 GCATACCATTAGCTTATATACGTCTGGGAACATTGGCACATGATATTGAATGTCATCCGG 237311

Query 526686 GTGGTCAAGGCGCAAAGCTGGCTCGAGCCGCAGGAACTTATGCTAAAATAATCAAGGAGC 526745

||||||||||||||||||||||||||||||||||||||||||||||||||||||||||||

Sbjct 237312 GTGGTCAAGGCGCAAAGCTGGCTCGAGCCGCAGGAACTTATGCTAAAATAATCAAGGAGC 237371

Query 526746 CAGCCCGTGTGCGTCTACCTAAGTCATTGATTCGTACTACCTACTTTTAGTAGAGGGTGC 526805

||||||||||||||||||||||||||||||||||||||||||||||||||||||||||||

Sbjct 237372 CAGCCCGTGTGCGTCTACCTAAGTCATTGATTCGTACTACCTACTTTTAGTAGAGGGTGC 237431

Query 526806 GCATAAGCAACGaaaaaaaGGAGGACAAAGGTGGTTAGGCAGACGCCCCATTGTTCGTGG 526865

||||||||||||||||||||||||||||||||||||||||||||||||||||||||||||

Sbjct 237432 GCATAAGCAACGAAAAAAAGGAGGACAAAGGTGGTTAGGCAGACGCCCCATTGTTCGTGG 237491

Query 526866 TGTTGCAATGAATCCAGTGGATCTTCCGGGGAGGTGAGGGGCGCACGAAAGGGCGTAGAC 526925

||||||||||||||||||||||||||||||||||||||||||||||||||||||||||||

Sbjct 237492 TGTTGCAATGAATCCAGTGGATCTTCCGGGGAGGTGAGGGGCGCACGAAAGGGCGTAGAC 237551

Query 526926 CTTCGGTGTCACCTTGGGGTAAGCCCACCAAAGCAGGATTTCGGGCAAGGGGTGGTAAAG 526985

||||||||||||||||||||||||||||||||||||||||||||||||||||||||||||

Sbjct 237552 CTTCGGTGTCACCTTGGGGTAAGCCCACCAAAGCAGGATTTCGGGCAAGGGGTGGTAAAG 237611

Query 526986 GGCAGAATTTCTTTTATGCCACGACGATCCATATGGAAGGGAAGTTTTGTTGATGCTTTC 527045

||||||||||||||||||||||||||||||||||||||||||||||||||||||||||||

Sbjct 237612 GGCAGAATTTCTTTTATGCCACGACGATCCATATGGAAGGGAAGTTTTGTTGATGCTTTC 237671

Query 527046 CTTTCACGAATGAAGAAGAAAGAAAATCTGATGAGCAGGAGAATTTGGTCACGTAGATCT 527105

||||||||||||||||||||||||||||||||||||||||||||||||||||||||||||

Sbjct 237672 CTTTCACGAATGAAGAAGAAAGAAAATCTGATGAGCAGGAGAATTTGGTCACGTAGATCT 237731

Query 527106 TCTATTTCGCCGGAATTCGTTGATTGCTCCGTACTCATTTACAATGGAAAAACTCCTGTT 527165

||||||||||||||||||||||||||||||||||||||||||||||||||||||||||||

Sbjct 237732 TCTATTTCGCCGGAATTCGTTGATTGCTCCGTACTCATTTACAATGGAAAAACTCCTGTT 237791

Query 527166 CGTTGTAAGATTACTGAAGGAAAGGTTGGTCATAAATTTGGAGAGTTTGCTAATACACGG 527225

||||||||||||||||||||||||||||||||||||||||||||||||||||||||||||

Sbjct 237792 CGTTGTAAGATTACTGAAGGAAAGGTTGGTCATAAATTTGGAGAGTTTGCTAATACACGG 237851

Query 527226 AGACGACGAAGACCTTCTAAAACAAAGGGGAAATAGAAAATGCTTCGGGAGTAAGAACTA 527285

||||||||||||||||||||||||||||||||||||||||||||||||||||||||||||

Sbjct 237852 AGACGACGAAGACCTTCTAAAACAAAGGGGAAATAGAAAATGCTTCGGGAGTAAGAACTA 237911

Query 527286 GTGATTGATAAGGCAAAgggggggAAGGACATAGGAAAGAGGGATGCCTACTTCAAATTG 527345

||||||||||||||||||||||||||||||||||||||||||||||||||||||||||||

Sbjct 237912 GTGATTGATAAGGCAAAGGGGGGGAAGGACATAGGAAAGAGGGATGCCTACTTCAAATTG 237971

Query 527346 CTCGGAAATTCTCAGCTATATGGGTGACTTGGATGGTGAGCAAAAAGAATTGATAAAGAA 527405

||||||||||||||||||||||||||||||||||||||||||||||||||||||||||||

Sbjct 237972 CTCGGAAATTCTCAGCTATATGGGTGACTTGGATGGTGAGCAAAAAGAATTGATAAAGAA 238031

Query 527406 ATTGGTCAACTTTCGCATGATCGATGGTAAAAGAACGAGAGTTCGTGCTATTGTTTATAA 527465

||||||||||||||||||||||||||||||||||||||||||||||||||||||||||||

Sbjct 238032 ATTGGTCAACTTTCGCATGATCGATGGTAAAAGAACGAGAGTTCGTGCTATTGTTTATAA 238091

Query 527466 TACTTTTCATCGCCTAGCTCGAAATGAACTCGATGTAATCAAACTGATGGTTGAGGCCGT 527525

||||||||||||||||||||||||||||||||||||||||||||||||||||||||||||

Sbjct 238092 TACTTTTCATCGCCTAGCTCGAAATGAACTCGATGTAATCAAACTGATGGTTGAGGCCGT 238151

Query 527526 AGATAATATTAAGCCCATATGCGAAGTGATCAAAGTAGGAGTCGCAGGTACTATTTATGA 527585

||||||||||||||||||||||||||||||||||||||||||||||||||||||||||||

Sbjct 238152 AGATAATATTAAGCCCATATGCGAAGTGATCAAAGTAGGAGTCGCAGGTACTATTTATGA 238211

Query 527586 TGTCCCTGGAATTGTAGCTCGGAATCGTCAACAAACCTTAGCTATTCGTTGGATAATGTT 527645

||||||||||||||||||||||||||||||||||||||||||||||||||||||||||||

Sbjct 238212 TGTCCCTGGAATTGTAGCTCGGAATCGTCAACAAACCTTAGCTATTCGTTGGATAATGTT 238271

Query 527646 CGCAGCTTTTAAACGACGCATAAGCTACAGGATAAGCTTAGAGAAATGTTTATTTGCTGA 527705

||||||||||||||||||||||||||||||||||||||||||||||||||||||||||||

Sbjct 238272 CGCAGCTTTTAAACGACGCATAAGCTACAGGATAAGCTTAGAGAAATGTTTATTTGCTGA 238331

Query 527706 GATACTGGATGCTTACAACAAGAAGGGAATTGCACATAAGAGAAGGCAGAATCTTCATGG 527765

||||||||||||||||||||||||||||||||||||||||||||||||||||||||||||

Sbjct 238332 GATACTGGATGCTTACAACAAGAAGGGAATTGCACATAAGAGAAGGCAGAATCTTCATGG 238391

Query 527766 ACTGGCTTCCACCAATCGAAGTGTCGCCCATTTCAGATGGTGGTAAAGACCACATAAGGA 527825

||||||||||||||||||||||||||||||||||||||||||||||||||||||||||||

Sbjct 238392 ACTGGCTTCCACCAATCGAAGTGTCGCCCATTTCAGATGGTGGTAAAGACCACATAAGGA 238451

Query 527826 GCACTTCCTCCCTCTTAGGCCATCCCATCATAAGGTATCCGCTCTCTCTTGCGGAGATTT 527885

||||||||||||||||||||||||||||||||||||||||||||||||||||||||||||

Sbjct 238452 GCACTTCCTCCCTCTTAGGCCATCCCATCATAAGGTATCCGCTCTCTCTTGCGGAGATTT 238511

Query 527886 CATTAAATTAAAAGTATGCTTCTCACGAGTTCCCCTGAGCTCTTCACTCAGCGTGGTGCT 527945

||||||||||||||||||||||||||||||||||||||||||||||||||||||||||||

Sbjct 238512 CATTAAATTAAAAGTATGCTTCTCACGAGTTCCCCTGAGCTCTTCACTCAGCGTGGTGCT 238571

Query 527946 GCTAGAAGCATGCTTCTTCTCAAAAGAAATAGGAGGAAATAAGAAGCTTCTGATGAGCAT 528005

||||||||||||||||||||||||||||||||||||||||||||||||||||||||||||

Sbjct 238572 GCTAGAAGCATGCTTCTTCTCAAAAGAAATAGGAGGAAATAAGAAGCTTCTGATGAGCAT 238631

Query 528006 CTATTGGAATCGATCATTTCCAAGATCTAATTCGAGTTTTTTATTAAGTAGTGGATACGC 528065

||||||||||||||||||||||||||||||||||||||||||||||||||||||||||||

Sbjct 238632 CTATTGGAATCGATCATTTCCAAGATCTAATTCGAGTTTTTTATTAAGTAGTGGATACGC 238691

Query 528066 CTCAAAATCTTCAGTGATACGCTTAAGGGATGATAAGTTCTTAGTGGATACAGGACTTGG 528125

||||||||||||||||||||||||||||||||||||||||||||||||||||||||||||

Sbjct 238692 CTCAAAATCTTCAGTGATACGCTTAAGGGATGATAAGTTCTTAGTGGATACAGGACTTGG 238751

Query 528126 TACCCCAAAAATTTGTATGAAAGATGAGCTGACTAAAGTTCCACAAAACCGACGAACCGC 528185

||||||||||||||||||||||||||||||||||||||||||||||||||||||||||||

Sbjct 238752 TACCCCAAAAATTTGTATGAAAGATGAGCTGACTAAAGTTCCACAAAACCGACGAACCGC 238811

Query 528186 CAGGTTCGAGAATAAGGTGGGATCCTCCTTTAATGTAATGGCTGGTGAATCAACGATCAA 528245

||||||||||||||||||||||||||||||||||||||||||||||||||||||||||||

Sbjct 238812 CAGGTTCGAGAATAAGGTGGGATCCTCCTTTAATGTAATGGCTGGTGAATCAACGATCAA 238871

Query 528246 AAAGCGACGGAATTATGAGAGAATCTTCAAGGATCTAGTGACCGGTGAATCACTGATCAA 528305

||||||||||||||||||||||||||||||||||||||||||||||||||||||||||||

Sbjct 238872 AAAGCGACGGAATTATGAGAGAATCTTCAAGGATCTAGTGACCGGTGAATCACTGATCAA 238931

Query 528306 AGAGCGAACAGCCGCCAGCTTGAATTCTTCTTTGGGATCCTTGGATGTAGCGGCGGGTGA 528365

||||||||||||||||||||||||||||||||||||||||||||||||||||||||||||

Sbjct 238932 AGAGCGAACAGCCGCCAGCTTGAATTCTTCTTTGGGATCCTTGGATGTAGCGGCGGGTGA 238991

Query 528366 ACCCCTTGTTCTTCCACGAAGATTCAGACAAAACCGAGCTTGGATAGAACTGCAGAAGAT 528425

||||||||||||||||||||||||||||||||||||||||||||||||||||||||||||

Sbjct 238992 ACCCCTTGTTCTTCCACGAAGATTCAGACAAAACCGAGCTTGGATAGAACTGCAGAAGAT 239051

Query 528426 TTGGCGAACGAATAAAAAGGCCAAAGGCTTTATTATTAATAAAGTCAAAAGAGGTTATTC 528485

||||||||||||||||||||||||||||||||||||||||||||||||||||||||||||

Sbjct 239052 TTGGCGAACGAATAAAAAGGCCAAAGGCTTTATTATTAATAAAGTCAAAAGAGGTTATTC 239111

Query 528486 AGTAGCCATCGTGGGTTTCATTACTTTTCTTCCATTATTaaaaaaaaaGAAGGGATTTTG 528545

||||||||||||||||||||||||||||||||||||||||||||||||||||||||||||

Sbjct 239112 AGTAGCCATCGTGGGTTTCATTACTTTTCTTCCATTATTAAAAAAAAAGAAGGGATTTTG 239171

Query 528546 GTCTCAAAGATGGAGTGGCTTGGGTAAGGTCAACCGCCCTTCGTTGTGAGATATGAAGTT 528605

||||||||||||||||||||||||||||||||||||||||||||||||||||||||||||

Sbjct 239172 GTCTCAAAGATGGAGTGGCTTGGGTAAGGTCAACCGCCCTTCGTTGTGAGATATGAAGTT 239231

Query 528606 ----ACCGAGCGAAAAACTTCTTTCTTTGAGCCTCGGCCCGGGCGGCCGGTGATCTTATC 528661

||||||||||||||||||||||||||||||||||||||||||||||||||||||||

Sbjct 239232 ACCGACCGAGCGAAAAACTTCTTTCTTTGAGCCTCGGCCCGGGCGGCCGGTGATCTTATC 239291

Query 528662 GATATGTGACTGGCAGTGACAGTAAGAGCAGAGATATTTTTATAAGAAAGTAGCGCTTAG 528721

||||||||||||||||||||||||||||||||||||||||||||||||||||||||||||

Sbjct 239292 GATATGTGACTGGCAGTGACAGTAAGAGCAGAGATATTTTTATAAGAAAGTAGCGCTTAG 239351

Query 528722 TCATAAAGAAAATCGTAAGAGAGTAGGAGGACGGCGGCTTCTTTCTACTATGTCATCAAA 528781

||||||||||||||||||||||||||||||||||||||||||||||||||||||||||||

Sbjct 239352 TCATAAAGAAAATCGTAAGAGAGTAGGAGGACGGCGGCTTCTTTCTACTATGTCATCAAA 239411

Query 528782 GGCCAACTGGTCTAATTCAAATTCTTTCAATTTTACAGAATATTTAAGCGACTTCCTTCT 528841

||||||||||||||||||||||||||||||||||||||||||||||||||||||||||||

Sbjct 239412 GGCCAACTGGTCTAATTCAAATTCTTTCAATTTTACAGAATATTTAAGCGACTTCCTTCT 239471

Query 528842 TAGTATCTTCCCAGGCTTTCAGCGCAGCTTGTTCATCTTCTATCGTTCTAATGGAAATGA 528901

||||||||||||||||||||||||||||||||||||||||||||||||||||||||||||

Sbjct 239472 TAGTATCTTCCCAGGCTTTCAGCGCAGCTTGTTCATCTTCTATCGTTCTAATGGAAATGA 239531

Query 528902 GACAAAGTGGTTATGAGAGAAGCAACATCTTTCACAGCATCTGGAGCCACTCAAGAAATA 528961

||||||||||||||||||||||||||||||||||||||||||||||||||||||||||||

Sbjct 239532 GACAAAGTGGTTATGAGAGAAGCAACATCTTTCACAGCATCTGGAGCCACTCAAGAAATA 239591

Query 528962 GGAAATCTAGTGAGATTCTTACTGAAACCATGACAGCAATATCTATTATTTCTCTCCCTT 529021

||||||||||||| ||||||||||||||||||||||||||||||||||||| ||||||||

Sbjct 239592 GGAAATCTAGTGATATTCTTACTGAAACCATGACAGCAATATCTATTATTTATCTCCCTT 239651

Query 529022 AGGTAAACAATAATGAAAGAAATGCAGTCCCTTTAGTAAGTTCATAATATAATAGGGGTA 529081

|||||||||||||||||||||||||||||||||||||||||| |||||||||||||||||

Sbjct 239652 AGGTAAACAATAATGAAAGAAATGCAGTCCCTTTAGTAAGTTAATAATATAATAGGGGTA 239711

Query 529082 TTTACTCATTCCCATCGTTGGATTATAATTTGGTTAAGAAATAAAAACGTATAAAATGAC 529141

||||||||||||||||||||||||| ||||||||||||||||||||||||||||||||||

Sbjct 239712 TTTACTCATTCCCATCGTTGGATTAGAATTTGGTTAAGAAATAAAAACGTATAAAATGAC 239771

Query 529142 ACTTTACTACCTCAAAAAAGATGCATTATCTTTTTAGTACACGAGCACATGAGTATAATG 529201

||||||||||||||||||||||||||||||||||||||||||||||||||||||||||||

Sbjct 239772 ACTTTACTACCTCAAAAAAGATGCATTATCTTTTTAGTACACGAGCACATGAGTATAATG 239831

Query 529202 GTTCTATACATCCAACTATGCAATCAGTAGGAGGTATGAAAGAAATGCAATTGTTTGTTG 529261

||||||||||||||||||||||||||||||||||||||||||||||||||||||||||||

Sbjct 239832 GTTCTATACATCCAACTATGCAATCAGTAGGAGGTATGAAAGAAATGCAATTGTTTGTTG 239891

Query 529262 ATGAGAAAGTACGAGTTGAAGATCAGAGAAAGATTGAGGGCCCAGGACCCGATTTCTTGT 529321

||||||||||||||||||||||||||||||||||||||||||||||||||||||||||||

Sbjct 239892 ATGAGAAAGTACGAGTTGAAGATCAGAGAAAGATTGAGGGCCCAGGACCCGATTTCTTGT 239951

Query 529322 TTAATAAATAATTGTGAAGATACATATGTAGGTAGCAATTTGGTGAAGGGAGAGCTCCCA 529381

|||| |||||||||||||||||||||||||||||||||||||||||||||||||||||||

Sbjct 239952 TTAAGAAATAATTGTGAAGATACATATGTAGGTAGCAATTTGGTGAAGGGAGAGCTCCCA 240011

Query 529382 CTGACCAAAAGCCCTATCGACAGGTTCAGTCAACGAAGTCCCCAGCTCCTTTAGGTGAGC 529441

||||||||||||||||||||||||||||||||||||||||||||||||||||||||||||

Sbjct 240012 CTGACCAAAAGCCCTATCGACAGGTTCAGTCAACGAAGTCCCCAGCTCCTTTAGGTGAGC 240071

Query 529442 AGGAAAAGAAGACGCAGATGTTTTCAGGTTGAGGCTTGCTTGCCCCGATTGATCCACTCG 529501

||||||||||||||||||||||||||||||||||||||||||||||||||||||||||||

Sbjct 240072 AGGAAAAGAAGACGCAGATGTTTTCAGGTTGAGGCTTGCTTGCCCCGATTGATCCACTCG 240131

Query 529502 AGAAGAATATACAGCGTTGGTTGCTGTCACAATCATAGATGGGCATAAAAAGACGGTTGA 529561

||||||||||||||||||||||||||||||||||||||||||||||||||||||||||||

Sbjct 240132 AGAAGAATATACAGCGTTGGTTGCTGTCACAATCATAGATGGGCATAAAAAGACGGTTGA 240191

Query 529562 TGCGAAACTGTCAAGCCCAAAGGCCATACCAAAAAGGTGTCGCCAATTCGTACTTCCCGA 529621

||||||||||||||||||||||||||||||||||||||||||||||||||||||||||||

Sbjct 240192 TGCGAAACTGTCAAGCCCAAAGGCCATACCAAAAAGGTGTCGCCAATTCGTACTTCCCGA 240251

Query 529622 GGACCCACATCTAGGTTTGTCCATTCCATTCAATGTTTACTTGAAATAAACTGTCGATCC 529681

||||||||||||||||||||||||||||||||||||||||||||||||||||||||||||

Sbjct 240252 GGACCCACATCTAGGTTTGTCCATTCCATTCAATGTTTACTTGAAATAAACTGTCGATCC 240311

Query 529682 TCATTATGCCTAGCACTTCCTTGTCCGATTCGATTCCCTAGGTAGGCTTTCCTGTCGTCT 529741

||||||||||||||||||||||||||||||||||||||||||||||||||||||||||||

Sbjct 240312 TCATTATGCCTAGCACTTCCTTGTCCGATTCGATTCCCTAGGTAGGCTTTCCTGTCGTCT 240371

Query 529742 TCTTCCAGGTTAGAAAATGCTGGTTCGATAGAGCCAGGGGAGCAGGAGTTAATCGTTCCC 529801

||||||||||||||||||||||||||||||||||||||||||||||||||||||||||||

Sbjct 240372 TCTTCCAGGTTAGAAAATGCTGGTTCGATAGAGCCAGGGGAGCAGGAGTTAATCGTTCCC 240431

Query 529802 CCCTTTGGGATTTCTCTTCCATCCAATCAAGTTTGAGTTGTAGTTCCCAGGAGAGCATTA 529861

||||||||||||||||||||||||||||||||||||||||||||||||||||||||||||

Sbjct 240432 CCCTTTGGGATTTCTCTTCCATCCAATCAAGTTTGAGTTGTAGTTCCCAGGAGAGCATTA 240491

Query 529862 GGTGAGTGTAGGTTAAGGAAGTGACGGGGGCTCTGTGTACAATTAGAGTATCTCATCCTG 529921

||||||||||||||||||||||||||||||||||||||||||||||||||||||||||||

Sbjct 240492 GGTGAGTGTAGGTTAAGGAAGTGACGGGGGCTCTGTGTACAATTAGAGTATCTCATCCTG 240551

Query 529922 CCCTAAACAAATAGATAACTTAAGATAGTTTTTGAGGAAAGCTAGAAAGCTACTACTTGA 529981

||||||||||||||||||||||||||||||||||||||||||||||||||||||||||||

Sbjct 240552 CCCTAAACAAATAGATAACTTAAGATAGTTTTTGAGGAAAGCTAGAAAGCTACTACTTGA 240611

Query 529982 TCTCGTGCATCCGACCTCTAGTCCTCAAAGCGAACACATGCCTTTGGAAAGCACTGCTAT 530041

||||||||||||||||||||||||||||||||||||||||||||||||||||||||||||

Sbjct 240612 TCTCGTGCATCCGACCTCTAGTCCTCAAAGCGAACACATGCCTTTGGAAAGCACTGCTAT 240671

Query 530042 GAAAAGGAAAGACCCGAAGGGATGCAAGAAATAAAGATAACGTTAGGGCCGGCAGCCTTC 530101

||||||||||||||||||||||||||||||||||||||||||||||||||||||||||||

Sbjct 240672 GAAAAGGAAAGACCCGAAGGGATGCAAGAAATAAAGATAACGTTAGGGCCGGCAGCCTTC 240731

Query 530102 CTTGTGCTCGTTGTCTAAGGGCTACTTTCTATCCTACGAAAAGAAAAAGAAAGGCCGCCG 530161

||||||||||||||||||||||||||||||||||||||||||||||||||||||||||||

Sbjct 240732 CTTGTGCTCGTTGTCTAAGGGCTACTTTCTATCCTACGAAAAGAAAAAGAAAGGCCGCCG 240791

Query 530162 CCTTACTAGTTCCACCAGCACCTTTTTTGCCCTAGAAAAATCCTTGCCTGTGAGAGCGAA 530221

||||||||||||||||||||||||||||||||||||||||||||||||||||||||||||

Sbjct 240792 CCTTACTAGTTCCACCAGCACCTTTTTTGCCCTAGAAAAATCCTTGCCTGTGAGAGCGAA 240851

Query 530222 GGACTTTCGAGAGGATACATAATTATTTTCTGAATCGAAGAAATAGATTCAGAAAACCCT 530281

||||||||||||||||||||||||||||||||||||||||||||||||||||||||||||

Sbjct 240852 GGACTTTCGAGAGGATACATAATTATTTTCTGAATCGAAGAAATAGATTCAGAAAACCCT 240911

Query 530282 ATATATGCTCTCGCTAACTACCCTCACAAAGTTCTGAACCTATCCTATTCCTGCCCCAGC 530341

||||||||||||||||||||||||||||||||||||||||||||||||||||||||||||

Sbjct 240912 ATATATGCTCTCGCTAACTACCCTCACAAAGTTCTGAACCTATCCTATTCCTGCCCCAGC 240971

Query 530342 TCCAACATCTGCTTCTGAAACGGAAGAAGCTTTGGCTTCTGGAGAAGAGGGAAAAGCGAT 530401

||||||||||||||||||||||||||||||||||||||||||||||||||||||||||||

Sbjct 240972 TCCAACATCTGCTTCTGAAACGGAAGAAGCTTTGGCTTCTGGAGAAGAGGGAAAAGCGAT 241031

Query 530402 CTATGATTGATACTTCACTTCGCCTTACTTTTCCCTTACTCCCTAAGATAAGACGATCGC 530461

||||||||||||||||||||||||||||||||||||||||||||||||||||||||||||

Sbjct 241032 CTATGATTGATACTTCACTTCGCCTTACTTTTCCCTTACTCCCTAAGATAAGACGATCGC 241091

Query 530462 TTTGGACGATACCCCTTTTTCCTCTACTTCCGACTTACCAAAGAGGGATTGCTGCGTATG 530521

||||||||||||||||||||||||||||||||||||||||||||||||||||||||||||

Sbjct 241092 TTTGGACGATACCCCTTTTTCCTCTACTTCCGACTTACCAAAGAGGGATTGCTGCGTATG 241151

Query 530522 TCCCTACCCCTTGTCTTTCTTCCGATTTTCTATCCCTATTCTCGAATCTTTTTGCTTTTG 530581

||||||||||||||||||||||||||||||||||||||||||||||||||||||||||||

Sbjct 241152 TCCCTACCCCTTGTCTTTCTTCCGATTTTCTATCCCTATTCTCGAATCTTTTTGCTTTTG 241211

Query 530582 AGAATTCCAGACAGGAAGAAGGAAAGGACTGACTGAACTTAATAATGAAATAATGACATA 530641

||||||||||||||||||||||||||||||||||||||||||||||||||||||||||||

Sbjct 241212 AGAATTCCAGACAGGAAGAAGGAAAGGACTGACTGAACTTAATAATGAAATAATGACATA 241271

Query 530642 AATTGAAAGAATTTTCCCAGATTTTTCTAATAAAAGAAGTAAAGAAAGAAAGATGCTCTA 530701

||||||||||||||||||||||||||||||||||||||||||||||||||||||||||||

Sbjct 241272 AATTGAAAGAATTTTCCCAGATTTTTCTAATAAAAGAAGTAAAGAAAGAAAGATGCTCTA 241331

Query 530702 CCACTCTATAGAACGAATTCTTCCTTTATAACCTAATTACTTAATTACTTAATTACCTAT 530761

||||||||||||||||||||||||||||||||||||||||||||||||||||||||||||

Sbjct 241332 CCACTCTATAGAACGAATTCTTCCTTTATAACCTAATTACTTAATTACTTAATTACCTAT 241391

Query 530762 TAACAATTCATAACACCATTCTAATGTTCAACTCTCTGATTTACTTTTTCCTTGACAACT 530821

||||||||||||||||||||||||||||||||||||||||||||||||||||||||||||

Sbjct 241392 TAACAATTCATAACACCATTCTAATGTTCAACTCTCTGATTTACTTTTTCCTTGACAACT 241451

Query 530822 GAAGGACTTAGTTACTTTGTTTCAGCCGAGTTCAAATTAGGTTTTTGGCTTTGATGATTC 530881

||||||||||||||||||||||||||||||||||||||||||||||||||||||||||||

Sbjct 241452 GAAGGACTTAGTTACTTTGTTTCAGCCGAGTTCAAATTAGGTTTTTGGCTTTGATGATTC 241511

Query 530882 CATTTCTGTTCCCAGTTGCACTTTTCCCGAGCTGAGAGAGACTGAATTTCTTTTTATAGG 530941

||||||||||||||||||||||||||||||||||||||||||||||||||||||||||||

Sbjct 241512 CATTTCTGTTCCCAGTTGCACTTTTCCCGAGCTGAGAGAGACTGAATTTCTTTTTATAGG 241571

Query 530942 GCCTCACTCTGTTTGGTAGGTTTTGTCGTCGAGTCAAACGCCATCACCGCGGTTTCCTGG 531001

||||||||||||||||||||||||||||||||||||||||||||||||||||||||||||

Sbjct 241572 GCCTCACTCTGTTTGGTAGGTTTTGTCGTCGAGTCAAACGCCATCACCGCGGTTTCCTGG 241631

Query 531002 TTCGATCACCCATATATCTAGTTTATAGGTGATCAACAGATTAGGTTCTTTCATATACGA 531061

||||||||||||||||||||||||||||||||||||||||||||||||||||||||||||

Sbjct 241632 TTCGATCACCCATATATCTAGTTTATAGGTGATCAACAGATTAGGTTCTTTCATATACGA 241691

Query 531062 TTTGGTTCAAATACGATAATACTTGGTGCCTTCTTCTTATCGACGTTTAGCTGTTCTATC 531121

||||||||||||||||||||||||||||||||||||||||||||||||||||||||||||

Sbjct 241692 TTTGGTTCAAATACGATAATACTTGGTGCCTTCTTCTTATCGACGTTTAGCTGTTCTATC 241751

Query 531122 CTCCCTTCTTCTCTTTCCTATTCTGAGTTGATACCCGCTCCACCTCGCTCGCCTTCTTTA 531181

||||||||||||||||||||||||||||||||||||||||||||||||||||||||||||

Sbjct 241752 CTCCCTTCTTCTCTTTCCTATTCTGAGTTGATACCCGCTCCACCTCGCTCGCCTTCTTTA 241811

Query 531182 TAGTATTTGCCCGACATCTCTTTTTCGATTATTTTTACCAACTCTTTCAGTTTGGACGTG 531241

||||||||||||||||||||||||||||||||||||||||||||||||||||||||||||

Sbjct 241812 TAGTATTTGCCCGACATCTCTTTTTCGATTATTTTTACCAACTCTTTCAGTTTGGACGTG 241871

Query 531242 AAACTCCTCCTGACTTGATCCAGGGTTTGTTTTTCTATCCCACTGTTTTGACACTTAGTC 531301

||||||||||||||||||||||||||||||||||||||||||||||||||||||||||||

Sbjct 241872 AAACTCCTCCTGACTTGATCCAGGGTTTGTTTTTCTATCCCACTGTTTTGACACTTAGTC 241931

Query 531302 TCCCGAATGAATCGAGAGGATCGGTCAAGTAGGAGATGGGTGAATAATTTTAAGAGGGTC 531361

||||||||||||||||||||||||||||||||||||||| |||| |||||||||||||||

Sbjct 241932 TCCCGAATGAATCGAGAGGATCGGTCAAGTAGGAGATGGTTGAAGAATTTTAAGAGGGTC 241991

Query 531362 AGAGTTTGATGCTGGAAAGCAGATGAGAAGATTTTTCATCTGAAACACATGGTGCAAATG 531421

||||||||||||||||||||||||||||||||||||||||||||||||||||||||||||

Sbjct 241992 AGAGTTTGATGCTGGAAAGCAGATGAGAAGATTTTTCATCTGAAACACATGGTGCAAATG 242051

Query 531422 CTTTGATCCGGGGCAAGCAACCAAACCTACTTCC 531455

||||||||||||||||||||||||||||||||||

Sbjct 242052 CTTTGATCCGGGGCAAGCAACCAAACCTACTTCC 242085

Range 2: 78144 to 116506

Score:70053 bits(37935), Expect:0.0,

Identities:38247/38388(99%), Gaps:59/38388(0%), Strand: Plus/Plus

Query 309540 TGGATAGAGAGCTCGGCTTCTA-----TTAGCTTCGAATGGTTTTTTCAAAGAAAGGTTT 309594

|||||||||||||||||||||| |||||||||||||||||||||||||||||||||

Sbjct 78144 TGGATAGAGAGCTCGGCTTCTATTAGCTTAGCTTCGAATGGTTTTTTCAAAGAAAGGTTT 78203

Query 309595 CGCTATACAACAACAATGAACTCACTTATGATCAAATTGGATGAATCGGTAATCTTTCTC 309654

||||||||||||||||||||||||||||||||||||||||||||||||||||||||||||

Sbjct 78204 CGCTATACAACAACAATGAACTCACTTATGATCAAATTGGATGAATCGGTAATCTTTCTC 78263

Query 309655 TGTCTTGAGAGAGAGAGTCACTTGTGAGAGCAGAATAAGGATTCATTTTTCATGTGTTCG 309714

||||||||||||||||||||||||||||||||||||||||||||||||||||||||||||

Sbjct 78264 TGTCTTGAGAGAGAGAGTCACTTGTGAGAGCAGAATAAGGATTCATTTTTCATGTGTTCG 78323

Query 309715 GTATTTGTCCTTTTCCTGGAAGGCCGATGCTTTTTGTATTTATTTTGATTTGATGTTACA 309774

||||||||||||||||||||||||||||||||||||||||||||||||||||||||||||

Sbjct 78324 GTATTTGTCCTTTTCCTGGAAGGCCGATGCTTTTTGTATTTATTTTGATTTGATGTTACA 78383

Query 309775 GTATTATTAGCTTCTTCAGCTTGCTTGTTATCCAACCGGCGAGGGTCAAGTGCTTACCTA 309834

||||||||||||||||||||||||||||||||||||||||||||||||||||||||||||

Sbjct 78384 GTATTATTAGCTTCTTCAGCTTGCTTGTTATCCAACCGGCGAGGGTCAAGTGCTTACCTA 78443

Query 309835 CCTATTAGAACATGGCTTTAGCAGAGCTTGCCCACCAGCTACAACTTCGTGCATTCGAAT 309894

||||||||||||||||||||||||||||||||||||||||||||||||||||||||||||

Sbjct 78444 CCTATTAGAACATGGCTTTAGCAGAGCTTGCCCACCAGCTACAACTTCGTGCATTCGAAT 78503

Query 309895 AGAAGGAAAAATAAATTGAAGGCTGCGGAAGAACAGaaaaaaaagggccaggaaaaaacc 309954

||||||||||| ||||||||||||||||||||||||||||||||||||||||||||||||

Sbjct 78504 AGAAGGAAAAAGAAATTGAAGGCTGCGGAAGAACAGAAAAAAAAGGGCCAGGAAAAAACC 78563

Query 309955 cattaaaaataaaGCGAGCATAATCAAAGGCCACCCAAAGAAGCATCCATGGAAAGGCTA 310014

||| ||||| ||||||||||||||||||||||||||||||||||||||||||||||||||

Sbjct 78564 CATGAAAAAGAAAGCGAGCATAATCAAAGGCCACCCAAAGAAGCATCCATGGAAAGGCTA 78623

Query 310015 GTGATAAAAGCATGAGAGTACGCGTTTTCACAAATGGTATGCCGCAAAATATGGGCGCAT 310074

||||||||||||||||||||||||||||||||||||||||||||||||||||||||||||

Sbjct 78624 GTGATAAAAGCATGAGAGTACGCGTTTTCACAAATGGTATGCCGCAAAATATGGGCGCAT 78683

Query 310075 TTCTATCGGTAAGCCGTTCAATAGAAGGTAAGTTTCTCAAATGTATTAAGTTAAGTGAAA 310134

||||||||||||||||||||||||||||||||||||||||||||||||||||||||||||

Sbjct 78684 TTCTATCGGTAAGCCGTTCAATAGAAGGTAAGTTTCTCAAATGTATTAAGTTAAGTGAAA 78743

Query 310135 AAAGGCCTGATTCGCTTTGACACATGCATTAAGTTTCATAAAAAGAATATCaaaaaaaTG 310194

||||||||||||||||||||||||||||||||||||||||||||||||||||||||||||

Sbjct 78744 AAAGGCCTGATTCGCTTTGACACATGCATTAAGTTTCATAAAAAGAATATCAAAAAAATG 78803

Query 310195 GATAACGCGTCTTCCTTTGACCAGAGCAGGTTCTTATGATAGGCACAGAGCAAGTGAAGC 310254

||||||||||||||||||||||||||||||||||||||||||||||||||||||||||||

Sbjct 78804 GATAACGCGTCTTCCTTTGACCAGAGCAGGTTCTTATGATAGGCACAGAGCAAGTGAAGC 78863

Query 310255 GAGCCGATAAAGTCTCAGTAGTGGGTCGGAAAAAATGTCAGTGCAGTCCGGCATAAAAGC 310314

||||||||||||||||||||||||||||||||||||||||||||||||||||||||||||

Sbjct 78864 GAGCCGATAAAGTCTCAGTAGTGGGTCGGAAAAAATGTCAGTGCAGTCCGGCATAAAAGC 78923

Query 310315 ACCCTTGGTCTGATGTGCTTCCCCCTAAATAAGTCTATGTCATCACAAGAAAGAGTCGTG 310374

||||||||||||||||||||||||||||||||||||||||||||||||||||||||||||

Sbjct 78924 ACCCTTGGTCTGATGTGCTTCCCCCTAAATAAGTCTATGTCATCACAAGAAAGAGTCGTG 78983

Query 310375 GGCGATAGAGAAAAAAGCCCAACTGCTTGTCCCAATATAGAAATTCTATAGTAAAATTTT 310434

||||||||||||||||||||||||||||||||||||||| ||||||||||||||||||||

Sbjct 78984 GGCGATAGAGAAAAAAGCCCAACTGCTTGTCCCAATATATAAATTCTATAGTAAAATTTT 79043

Query 310435 CTAGAGGTAAGTGAGGGATGGATCTGTCCTCTTCTTCCCTTAACCAAAGAGTCGTCCCTC 310494

||||||||||||||||||||||||||||||||||||||||||||||||||||||||||||

Sbjct 79044 CTAGAGGTAAGTGAGGGATGGATCTGTCCTCTTCTTCCCTTAACCAAAGAGTCGTCCCTC 79103

Query 310495 TGGAGGTATTTCTTTTCTGCCGTGGGCCAGTGGAAAAAATAAATCGGCGTAACCGAAGTT 310554

||||||||||||||||||||||||||||||||||||||||||||||||||||||||||||

Sbjct 79104 TGGAGGTATTTCTTTTCTGCCGTGGGCCAGTGGAAAAAATAAATCGGCGTAACCGAAGTT 79163

Query 310555 AAATCCCATATTGTCTAAGCGACCTTGCCTCAGAGAATAGGTAGGTGTGTTAAAGGAAAC 310614

||||||||||||||||||||||||||||||||||||||||||||||||||||||||||||

Sbjct 79164 AAATCCCATATTGTCTAAGCGACCTTGCCTCAGAGAATAGGTAGGTGTGTTAAAGGAAAC 79223

Query 310615 TTGAAATAAGTAGGCTTTGCCACCCATTATTTTTTAATAGCTTAGTAGCCTTGCACTTCT 310674

||||||||||||||||||||| ||||||||||||||||||||||||||||||||||||||

Sbjct 79224 TTGAAATAAGTAGGCTTTGCCGCCCATTATTTTTTAATAGCTTAGTAGCCTTGCACTTCT 79283

Query 310675 AAAAGAAAGATAAGCGTAGCACAGTGAAATAATAACTAAAATAAAATCATCTATaaaaaa 310734

||||||||||||||||||||||||||||||||||||||||||||||||||||||||||||

Sbjct 79284 AAAAGAAAGATAAGCGTAGCACAGTGAAATAATAACTAAAATAAAATCATCTATAAAAAA 79343

Query 310735 aaGAAGCCCCTGAGGCGCTGTACCAAAGGCAACTTTACTTTATAAAAAGTTCTAGTGAGC 310794

||||||||||||||||||||||||||||||||||||||||||||||||||||||||||||

Sbjct 79344 AAGAAGCCCCTGAGGCGCTGTACCAAAGGCAACTTTACTTTATAAAAAGTTCTAGTGAGC 79403

Query 310795 CCACTTGCCTGAGTAGTATGTATCCGGGGGGTAGAGTAGCAAGTGGGAAAGAAAGAAGTG 310854

||||||||||||||||||||||||||||||||||||||||||||||||||||||||||||

Sbjct 79404 CCACTTGCCTGAGTAGTATGTATCCGGGGGGTAGAGTAGCAAGTGGGAAAGAAAGAAGTG 79463

Query 310855 AGGTTCCTTCCCTCCAGCTTTCAATTGAGCGTATTACTTTAAGTCTCCGGTCCGGGTAAA 310914

||||||||||||||||||||||||||||||||||||||||||||||||||||||||||||

Sbjct 79464 AGGTTCCTTCCCTCCAGCTTTCAATTGAGCGTATTACTTTAAGTCTCCGGTCCGGGTAAA 79523

Query 310915 GCTACTACAATGTAGAGAGACCATGCCAGCTCCTTTCCTCCGCCAGTCGGGAGATTGCCA 310974

||||||||||||||||||||||||||||||||||||||||||||||||||||||||||||

Sbjct 79524 GCTACTACAATGTAGAGAGACCATGCCAGCTCCTTTCCTCCGCCAGTCGGGAGATTGCCA 79583

Query 310975 CCAGTTACTTTTATTGAGAAACAGGTCCTTAACGaaaaaaaaTCTTTAATCCCCCAGTTA 311034

||||||||||||||||||||| ||||||||||||||||||||||||||||||||||||||

Sbjct 79584 CCAGTTACTTTTATTGAGAAAAAGGTCCTTAACGAAAAAAAATCTTTAATCCCCCAGTTA 79643

Query 311035 CTCGCTTGCCTCTTTCTTATGCATTCTAAACTGCAGCATCTTCTGAACCTCGCCCCGGTC 311094

||||||||||||||||||||||||||||||||||||||||||||||||||||||||||||

Sbjct 79644 CTCGCTTGCCTCTTTCTTATGCATTCTAAACTGCAGCATCTTCTGAACCTCGCCCCGGTC 79703

Query 311095 AAAGTCTTTCCTTTAGTCTGGATATGTAGGAGAATCCTTATAGAACAAGGAGGCTCCCCA 311154

||||||||||||||||||||||||||||||||||||||||||||||||||||||||||||

Sbjct 79704 AAAGTCTTTCCTTTAGTCTGGATATGTAGGAGAATCCTTATAGAACAAGGAGGCTCCCCA 79763

Query 311155 TTTCCCCTAGCGAGGGTATTGGGAAAGCTAGTTCTTCAGTAAATTTAGTTCCTTCCTAAT 311214

||||||||||||||||||||||||||||||||||||||||||||||||||||||||||||

Sbjct 79764 TTTCCCCTAGCGAGGGTATTGGGAAAGCTAGTTCTTCAGTAAATTTAGTTCCTTCCTAAT 79823

Query 311215 AGAAAAGCATCCGAGTGGAGCCCCTTTTCCTTTCAACGTGGCCGAATAActttctcttgc 311274

||||||||||||||||||||||||||||||||||||||||||||||||||||||||||||

Sbjct 79824 AGAAAAGCATCCGAGTGGAGCCCCTTTTCCTTTCAACGTGGCCGAATAACTTTCTCTTGC 79883

Query 311275 tctccccctttatctttctctctgtttcactcttctttcttattatttcGCTAACTTGCT 311334

||||||||||||||||||||||||||||||||||||||||||||||||||||||||||||

Sbjct 79884 TCTCCCCCTTTATCTTTCTCTCTGTTTCACTCTTCTTTCTTATTATTTCGCTAACTTGCT 79943

Query 311335 TGCGGAAAGACTACATACAGTATGGAAAGGTGCTCAGCAGCACGTGAATATCCTAACTTG 311394

||||||||||||||||||||||||||||||||||||||||||||||||||||||||||||

Sbjct 79944 TGCGGAAAGACTACATACAGTATGGAAAGGTGCTCAGCAGCACGTGAATATCCTAACTTG 80003

Query 311395 CCTCGCAACAAGTGACAGCGTTTATTTCATTTATCTATATTATATTAGTTTAGCTTAAAC 311454

||||||||||||||||||||||||||||||||||||||||||||||||||||||||||||

Sbjct 80004 CCTCGCAACAAGTGACAGCGTTTATTTCATTTATCTATATTATATTAGTTTAGCTTAAAC 80063

Query 311455 GGAACGAGTGTTCCACAAATCCATCAATAGAACCAAGTTCGGATGAAGTACTTGGTAGGA 311514

||||||||||||||||||||||||||||||||||||||||||||||||||||||||||||

Sbjct 80064 GGAACGAGTGTTCCACAAATCCATCAATAGAACCAAGTTCGGATGAAGTACTTGGTAGGA 80123

Query 311515 CCGTAGCCCAAGCCACCAGGCGAGGCCGCTAAGGAAGTACATACCACAGTAGAATCATAT 311574

||||||||||||||||||||||||||||||||||||||||||||||||||||||||||||

Sbjct 80124 CCGTAGCCCAAGCCACCAGGCGAGGCCGCTAAGGAAGTACATACCACAGTAGAATCATAT 80183

Query 311575 ACAGTGTATAGCTGTAGAAAAGTTTTCGACTAGAGATGGGCTTTATATCTAACAGGTAGT 311634

||||||||||||||||||||||||||||||||||||||||||||||||||||||||||||

Sbjct 80184 ACAGTGTATAGCTGTAGAAAAGTTTTCGACTAGAGATGGGCTTTATATCTAACAGGTAGT 80243

Query 311635 CGGAGTGATCAAAGCGCTCCATCCAAGCTTGCTTTGTCGGACAACCTCGTCATTCCGATA 311694

||||||||||||||||||||||||||||||||||||||||||||||||||||||||||||

Sbjct 80244 CGGAGTGATCAAAGCGCTCCATCCAAGCTTGCTTTGTCGGACAACCTCGTCATTCCGATA 80303

Query 311695 CAGAATTCTAAGTAAAGCTATTGACATTCCTTTGTAGCTTTGCCCCTGTAGCCTTAAGTA 311754

||||||||||||||||||||||||||||||||||||||||||||||||||||||||||||

Sbjct 80304 CAGAATTCTAAGTAAAGCTATTGACATTCCTTTGTAGCTTTGCCCCTGTAGCCTTAAGTA 80363

Query 311755 TAGGTTTCCCCCAATCAACTCCAGATTCTGCTAATCAGGCTACACGCTAGGAAGTTGGCT 311814

||||||||||||||||||||||||||||||||||||||||||||||||||||||||||||

Sbjct 80364 TAGGTTTCCCCCAATCAACTCCAGATTCTGCTAATCAGGCTACACGCTAGGAAGTTGGCT 80423

Query 311815 AGTTATATATAGTTGGGCAACCCTTACTGAGTTTAGGTTTAGCTGGTATCACCAACTCAC 311874

||||||||||||||||||||||||||||||||||||||||||||||||||||||||||||

Sbjct 80424 AGTTATATATAGTTGGGCAACCCTTACTGAGTTTAGGTTTAGCTGGTATCACCAACTCAC 80483

Query 311875 AGGGCATAAGGTTTAAACAAAATAGCCGGGATATCTTCATCCGGGTGGGGGAAAATAGCA 311934

||||||||||||||||||||||||||||||||||||||||||||||||||||||||||||

Sbjct 80484 AGGGCATAAGGTTTAAACAAAATAGCCGGGATATCTTCATCCGGGTGGGGGAAAATAGCA 80543

Query 311935 GAAATCTTTCTTTGTTGGACAACCAGGGCTGAAAAGTAATCCACAGTCTCCTTTTTCGCT 311994

||||||||||||||||||||||||||||||||||||||||||||||||||||||||||||

Sbjct 80544 GAAATCTTTCTTTGTTGGACAACCAGGGCTGAAAAGTAATCCACAGTCTCCTTTTTCGCT 80603

Query 311995 GAACCAGGGAGGGGCATTAGCAGTTGAAGAAAGACAGAATTTATTCCAGTAGCTTTACGA 312054

||||||||||||||||||||||||||||||||||||||||||||||||||||||||||||

Sbjct 80604 GAACCAGGGAGGGGCATTAGCAGTTGAAGAAAGACAGAATTTATTCCAGTAGCTTTACGA 80663

Query 312055 CATACATTTCTGAATAGGAAAAGGAAGTATGTGAAATCTTTCCGGGTCATACCCCTAGTT 312114

||||||||||||||||||||||||||||||||||||||||||||||||||||||||||||

Sbjct 80664 CATACATTTCTGAATAGGAAAAGGAAGTATGTGAAATCTTTCCGGGTCATACCCCTAGTT 80723

Query 312115 ACGAGTCTTTCCTATCCCTGTAGCCCCACTTACATGTCTTTGGCCTTTACTCCCCGATGA 312174

||||||||||||||||||||||||||||||||||||||||||||||||||||||||||||

Sbjct 80724 ACGAGTCTTTCCTATCCCTGTAGCCCCACTTACATGTCTTTGGCCTTTACTCCCCGATGA 80783

Query 312175 ACAAATAGAGTCAAAAAGGGGATTCTACTGTACCTATAACAATCACTTACGGCAAATCGA 312234

||||||||||||||||||||||||||||||||||||||||||||||||||||||||||||

Sbjct 80784 ACAAATAGAGTCAAAAAGGGGATTCTACTGTACCTATAACAATCACTTACGGCAAATCGA 80843

Query 312235 GTACCCGCACTATACGTTTATCTACGGCGTAATCGTTACAAGTTGAGAATTCCATGAAAA 312294

||||||||||||||||||||||||||||||||||||||||||||||||||||||||||||

Sbjct 80844 GTACCCGCACTATACGTTTATCTACGGCGTAATCGTTACAAGTTGAGAATTCCATGAAAA 80903

Query 312295 GCCTTTCTTTATAGCACGCGCCTAATCTATCAACTAGCGGCGTATACTCCTACTAGTCTG 312354

||||||||||||||||||||||||||||||||||||||||||||||||||||||||||||

Sbjct 80904 GCCTTTCTTTATAGCACGCGCCTAATCTATCAACTAGCGGCGTATACTCCTACTAGTCTG 80963

Query 312355 TCAGGGATGAATATGATTGATCTTACCAAGAGGAAGATACTCGCTCTTCTGTCTGGTCCA 312414

||||||||||||||||||||||||||||||||||||||||||||||||||||||||||||

Sbjct 80964 TCAGGGATGAATATGATTGATCTTACCAAGAGGAAGATACTCGCTCTTCTGTCTGGTCCA 81023

Query 312415 ATACACTAGCATACTTCGTATGGCTCGCTTCACTCTCGTATTGCTCTTGCTTTTGGGTCA 312474

||||||||||||||||||||||||||||||||||||||||||||||||||||||||||||

Sbjct 81024 ATACACTAGCATACTTCGTATGGCTCGCTTCACTCTCGTATTGCTCTTGCTTTTGGGTCA 81083

Query 312475 AGCGGAAGTAAAATCCTGTTGTTCAGAAATAGGGTAAGGTGGTTTTGGGTCACCCTCATG 312534

||||||||| ||||||||||||||||||||||||||||||||||||||||||||||||||

Sbjct 81084 AGCGGAAGTCAAATCCTGTTGTTCAGAAATAGGGTAAGGTGGTTTTGGGTCACCCTCATG 81143

Query 312535 TAAGCGTAGGCGTGGTCAAAGCCGCAGTTTTCAACCCTTATCCTGTTTGACTTTTCAGCT 312594

||||||||||||||||||||||||||||||||||||||||||||||||||||||||||||

Sbjct 81144 TAAGCGTAGGCGTGGTCAAAGCCGCAGTTTTCAACCCTTATCCTGTTTGACTTTTCAGCT 81203

Query 312595 GTACCCGGCTCGAAGAGAGCGTCAATGTTCCGTCTGATTATGCCAATGGATTGTTACAGG 312654

||||||||||||||||||||||||||||||||||||||||||||||||||||||||||||

Sbjct 81204 GTACCCGGCTCGAAGAGAGCGTCAATGTTCCGTCTGATTATGCCAATGGATTGTTACAGG 81263

Query 312655 TACCCGGTATGATAACGTATTGTGTCAAGCATGCGGGATGTGAAGCAAAAGGAAGCACTC 312714

||||||||||||||||||||||||||||||||||||||||||||||||||||||||||||

Sbjct 81264 TACCCGGTATGATAACGTATTGTGTCAAGCATGCGGGATGTGAAGCAAAAGGAAGCACTC 81323

Query 312715 AACACTACTCTTGGTTTCTGTTAGAATGGCGGGTAAGTAAGACTGGTTAAGTACTCTTCT 312774

||||||||||||||||||||||||||||||||||||||||||||||||||||||||||||

Sbjct 81324 AACACTACTCTTGGTTTCTGTTAGAATGGCGGGTAAGTAAGACTGGTTAAGTACTCTTCT 81383

Query 312775 TCGTGAAGCTAAAGGAAGTGGATTGCTCATAAGAAAAAGAAGTGAAGGTAGCTGAGCTCC 312834

||||||||||||||||||||||||||||||||||||||||||||||||||||||||||||

Sbjct 81384 TCGTGAAGCTAAAGGAAGTGGATTGCTCATAAGAAAAAGAAGTGAAGGTAGCTGAGCTCC 81443

Query 312835 AGCCAAGCCTAAGCGATCAAGAATTTCTAGCTAAGTGAAAGAGAGTGAGTTTTCCTCATA 312894

||||||||||||||||||||||||||||||||||||||||||||||||||||||||||||

Sbjct 81444 AGCCAAGCCTAAGCGATCAAGAATTTCTAGCTAAGTGAAAGAGAGTGAGTTTTCCTCATA 81503

Query 312895 GGCATTTGTACCACTGGCGAAGATGACTGGTTATGATGGCCTTTTCTTTGAAAAGATACA 312954

||||||||||||||||||||||||||||||||||||||||||||||||||||||||||||

Sbjct 81504 GGCATTTGTACCACTGGCGAAGATGACTGGTTATGATGGCCTTTTCTTTGAAAAGATACA 81563

Query 312955 CTTAGCCGAAGCCTTTTCCACCCCGAGATCCTAGAGTTTCTTTCACGGATTTTATGGTTT 313014

||||||||||||||||||||||||||||||||||||||||||||||||||||||||||||

Sbjct 81564 CTTAGCCGAAGCCTTTTCCACCCCGAGATCCTAGAGTTTCTTTCACGGATTTTATGGTTT 81623

Query 313015 TTGCTAAGTCACTACCTCTTTTCTTATGGCTAGGAATGTGCTAGAGTTTGGGAAGGTTGT 313074

||||||||||||||||||||||||||||||||||||||||||||||||||||||||||||

Sbjct 81624 TTGCTAAGTCACTACCTCTTTTCTTATGGCTAGGAATGTGCTAGAGTTTGGGAAGGTTGT 81683

Query 313075 TATTGGCCTTACTTTACCTACCGTGTTGCCTTACCAACTCCACTAGCTATATGTGCGTTA 313134

||||||||||||||||||||||||||||||||||||||||||||||||||||||||||||

Sbjct 81684 TATTGGCCTTACTTTACCTACCGTGTTGCCTTACCAACTCCACTAGCTATATGTGCGTTA 81743

Query 313135 GTAGGTGGCAGGTTCACCTGGTCTAGTTATGCTCTTTATCTCACAGTCTTATCCACGGGA 313194

||||||||||||||||||||||||||||||||||||||||||||||||||||||||||||

Sbjct 81744 GTAGGTGGCAGGTTCACCTGGTCTAGTTATGCTCTTTATCTCACAGTCTTATCCACGGGA 81803

Query 313195 TGCTGACCGATTGACACCtttttttACCAGATGGTTCACTTACGCTACTCATTTATATAC 313254

||||||||||||||||||||||||||||||||||||||||||||||||||||||||||||

Sbjct 81804 TGCTGACCGATTGACACCTTTTTTTACCAGATGGTTCACTTACGCTACTCATTTATATAC 81863

Query 313255 CATTCCGGAttttcttttttaccgtttttACCACGCCTCTAGAACCATATATTCTTCTGA 313314

||||||||||||||||||||||||||||||||||||||||||||||||||||||||||||

Sbjct 81864 CATTCCGGATTTTCTTTTTTACCGTTTTTACCACGCCTCTAGAACCATATATTCTTCTGA 81923

Query 313315 TCCGATTACGACTCCAAGGGGGTTTTTCCTATGAGCTAATCATAGCTGGAGTTGAACTAA 313374

||||||||||||||||||||||||||||||||||||||||||||||||||||||||||||

Sbjct 81924 TCCGATTACGACTCCAAGGGGGTTTTTCCTATGAGCTAATCATAGCTGGAGTTGAACTAA 81983

Query 313375 CTGTTCACAGGTTCTTATTTAGTCCTGCTTATCACTCCGGAACATATAGTTTCTTGTGGT 313434

||||||||||||||||||||||||||||||||||||||||||||||||||||||||||||

Sbjct 81984 CTGTTCACAGGTTCTTATTTAGTCCTGCTTATCACTCCGGAACATATAGTTTCTTGTGGT 82043

Query 313435 GGCTAGACACCTCTCCGATAGCCTTCTCAGGTAATCCCAGACAAAGGGTAATAATACCCT 313494

|||||||||||||||||||||||||||||||||||||||||||||||||||||| |||||

Sbjct 82044 GGCTAGACACCTCTCCGATAGCCTTCTCAGGTAATCCCAGACAAAGGGTAATAAGACCCT 82103

Query 313495 CATATGATAGAACAGCTATCTACACTGCTGGTCTGGCCTTATATGTATATGTAAAAATTT 313554

||||||||||||||||||||||||||||||||||||||||||||||||||||||||||||

Sbjct 82104 CATATGATAGAACAGCTATCTACACTGCTGGTCTGGCCTTATATGTATATGTAAAAATTT 82163

Query 313555 TGTTGAAGTTTTACATACCGACAAACATTGCTTTCTTTGATCATAGGCAAATTATACTAA 313614

||||||||||| ||||||||||||||||||||||||||||||||||||||||||||||||

Sbjct 82164 TGTTGAAGTTTGACATACCGACAAACATTGCTTTCTTTGATCATAGGCAAATTATACTAA 82223

Query 313615 ACAGCTACTATATTGATGCTGGTATGGGTCATAGGTTCTATGATTATATGTGGAACAGGT 313674

||||||||||||||||||||||||||||||||||||||||||||||||||||||||||||

Sbjct 82224 ACAGCTACTATATTGATGCTGGTATGGGTCATAGGTTCTATGATTATATGTGGAACAGGT 82283

Query 313675 CGGACATATATTCAATCTCAATGTAGCGCAAATTTACTAGAAAGTTGATTTGGAAAAGGT 313734

|||||||||||||||||||||||||||||||||||||||||||||| |||||||||||||

Sbjct 82284 CGGACATATATTCAATCTCAATGTAGCGCAAATTTACTAGAAAGTTTATTTGGAAAAGGT 82343

Query 313735 CGGACATATATGCCTATGGTGTTCAGCTTCTAGAACTGCTAACTGGGAAACCACCAGTAG 313794

||||||||||||||||||||||||||||||||||||||||||||||||||||||||||||

Sbjct 82344 CGGACATATATGCCTATGGTGTTCAGCTTCTAGAACTGCTAACTGGGAAACCACCAGTAG 82403

Query 313795 CCCAACAGAGCATAGTTTCGAATGAGCTGCTGGCGTGGGTCCGGTCTGTCAGAGAGGAAG 313854

||||||||||||||||||||||||||||||||||||||||||||||||||||||||||||

Sbjct 82404 CCCAACAGAGCATAGTTTCGAATGAGCTGCTGGCGTGGGTCCGGTCTGTCAGAGAGGAAG 82463

Query 313855 GAGAAAGTGCAGACGATGAAAGACTAAGCATGATTGTGGACATAGCGGCAACATGCATTC 313914

||||||||||||||||||||||||||||||||||||||||||||||||||||||||||||

Sbjct 82464 GAGAAAGTGCAGACGATGAAAGACTAAGCATGATTGTGGACATAGCGGCAACATGCATTC 82523

Query 313915 AATTGTCTCCAGAGAGTCGACCGACAGCATGGGAGGTTCTGAAGATGATCCAAGAGGTTA 313974

||||||||||||||||||||||||||||||||||||||||||||||||||||||||||||

Sbjct 82524 AATTGTCTCCAGAGAGTCGACCGACAGCATGGGAGGTTCTGAAGATGATCCAAGAGGTTA 82583

Query 313975 AGGAGGCCGAAGCTGGAGACCGGGGAATGAGAGCTCCATACACCATGATGAATGGAGTCT 314034

||||||||||||||||||||||||||||||||||||||||||||||||||||||||||||

Sbjct 82584 AGGAGGCCGAAGCTGGAGACCGGGGAATGAGAGCTCCATACACCATGATGAATGGAGTCT 82643

Query 314035 TTCCTCTCGTTTTTTGGTTGTTTGTGTATATACCCAACTGTAGGTCACCTATGTGACCAA 314094

||||||||||||||||||||||||||||||||||||||||||||||||||||||||||||

Sbjct 82644 TTCCTCTCGTTTTTTGGTTGTTTGTGTATATACCCAACTGTAGGTCACCTATGTGACCAA 82703

Query 314095 TCCTAACCATTGGTTTCACAGCACTTAGTAAGCATACGTTTGAAGACCGAGTTGGTTGAT 314154

||||||||||||||||||||||||||||||||||||||||||||||||||||||||||||

Sbjct 82704 TCCTAACCATTGGTTTCACAGCACTTAGTAAGCATACGTTTGAAGACCGAGTTGGTTGAT 82763

Query 314155 TTGGTCTGTCCATTCGCTCTAGGAGGGGCTCGACTTCCTTCGAGCTATGATAAATTAATA 314214

||||||||||||||||||||||||||||||||||||||||||||||||||||||||||||

Sbjct 82764 TTGGTCTGTCCATTCGCTCTAGGAGGGGCTCGACTTCCTTCGAGCTATGATAAATTAATA 82823

Query 314215 GGGGAGCGCCTTCTTGTTTTTTCTGCGATCTCCTTGGCTACAAAGAGAGCTATGGAGCCT 314274

||||||||||||||||||||||||||||||||||||||||||||||||||||||||||||

Sbjct 82824 GGGGAGCGCCTTCTTGTTTTTTCTGCGATCTCCTTGGCTACAAAGAGAGCTATGGAGCCT 82883

Query 314275 AGCTGAAAGCTTGACAAGGCCTATTTAGTGTATTCGGCCACCCAACCACTCGCAGAAAGA 314334

||||||||||||||||||||||||||||||||||||||||||||||||||||||||||||

Sbjct 82884 AGCTGAAAGCTTGACAAGGCCTATTTAGTGTATTCGGCCACCCAACCACTCGCAGAAAGA 82943

Query 314335 ATGGGGCATTCAAAACCAGACAGGGATTCCTTCTCCTAGGGTTGCTCTGAGGTAACCGCT 314394

||||||||||||||||||||||||||||||||||||||||||||||||||||||||||||

Sbjct 82944 ATGGGGCATTCAAAACCAGACAGGGATTCCTTCTCCTAGGGTTGCTCTGAGGTAACCGCT 83003

Query 314395 CGTCTTGATGCTGGCAATCAAGAAGGCAAAGAGTTGACTTCTACTTCTGAAGTGTACGGA 314454

||||||||||||||||||||||||||||||||||||||||||||||||||||||||||||

Sbjct 83004 CGTCTTGATGCTGGCAATCAAGAAGGCAAAGAGTTGACTTCTACTTCTGAAGTGTACGGA 83063

Query 314455 GTTTACCTTCCTTCGCCTATCTCGGATTCTGCTACAGATTGGAAAAAGAGTTCACATTCA 314514

||||||||||||||||||||||||||||||||||||||||||||||||||||||||||||

Sbjct 83064 GTTTACCTTCCTTCGCCTATCTCGGATTCTGCTACAGATTGGAAAAAGAGTTCACATTCA 83123

Query 314515 CTTGGGAATACCAAATATATATAATGCAGTAGAGACAACCACAAGTAGAGGAACGACCGA 314574

||||||||||||||||||||||||||||||||||||||||||||||||||||||||||||

Sbjct 83124 CTTGGGAATACCAAATATATATAATGCAGTAGAGACAACCACAAGTAGAGGAACGACCGA 83183

Query 314575 CACAGCTGGCATGCCCCCGCAACCTTCGATCCATCTATCTAACGTTCTACTACAGAACTG 314634

||||||||||||||||||||||||||||||||||||||||||||||||||||||||||||

Sbjct 83184 CACAGCTGGCATGCCCCCGCAACCTTCGATCCATCTATCTAACGTTCTACTACAGAACTG 83243

Query 314635 AGTTCATCATAGCTGCCAGCCTCGGTAAGGTTAACTAGCAATTTGACTCACAGCACTAAA 314694

||||||||||||||||||||||||||||||||||||||||||||||||||||||||||||

Sbjct 83244 AGTTCATCATAGCTGCCAGCCTCGGTAAGGTTAACTAGCAATTTGACTCACAGCACTAAA 83303

Query 314695 GATAGCAAAGCGACAATCGGTATGGGTGGAGCCTAGTATAGCTGCGctttcttttcttct 314754

||||||||||||||||||||||||||||||||||||||||||||||||||||||||||||

Sbjct 83304 GATAGCAAAGCGACAATCGGTATGGGTGGAGCCTAGTATAGCTGCGCTTTCTTTTCTTCT 83363

Query 314755 ttcttcttGGTCTAAGTACCCCTCTCTTTCCAGCACCCTTATAGAGATAGAGAGATTGAT 314814

||||||||||||||||||||||||||||||||||||||||||||||||||||||||||||

Sbjct 83364 TTCTTCTTGGTCTAAGTACCCCTCTCTTTCCAGCACCCTTATAGAGATAGAGAGATTGAT 83423

Query 314815 TGTGATTGAGTCTTTTTATCATTGACTGGCCCACTATTCACTTTCTTCTTTTTTACTCCC 314874

||||||||||||||||||||||||||||||||||||||||||||||||||||||||||||

Sbjct 83424 TGTGATTGAGTCTTTTTATCATTGACTGGCCCACTATTCACTTTCTTCTTTTTTACTCCC 83483

Query 314875 TACTTTCTTATAGAGATGACGAAACGAGGGTTAGTCTTTCTATGGCAAAGGGGTTGTGCG 314934

||||||||||||||||||||||||||||||||||||||||||||||||||||||||||||

Sbjct 83484 TACTTTCTTATAGAGATGACGAAACGAGGGTTAGTCTTTCTATGGCAAAGGGGTTGTGCG 83543

Query 314935 GACCTTACCCAGCCGAGCTCAGCAGACTTTAATCGACCCTCTCTATGCTGAGCACAGGCA 314994

||||||||||||||||||||||||||||||||||||||||||||||||||||||||||||

Sbjct 83544 GACCTTACCCAGCCGAGCTCAGCAGACTTTAATCGACCCTCTCTATGCTGAGCACAGGCA 83603

Query 314995 ACTACGCTGGGACTCTTCACAGTAAAATTTTATACAAAAGTATGCTACACTTTCGAGCTC 315054

||||||||||||||||||||||||||||||||||||||||||||||||||||||||||||

Sbjct 83604 ACTACGCTGGGACTCTTCACAGTAAAATTTTATACAAAAGTATGCTACACTTTCGAGCTC 83663

Query 315055 CCTCCCTTATAGAGATAAAGATTACATaaaaaaaaTAAGAGGTAATAAAAAGTATCCCTT 315114

||||||||||||||||||||||||||||||||||||||||||||||||||||||||||||

Sbjct 83664 CCTCCCTTATAGAGATAAAGATTACATAAAAAAAATAAGAGGTAATAAAAAGTATCCCTT 83723

Query 315115 ACTATTTGGGTGCGAACTGCGGACGTTTCACTTAGTAGTTTGGcttatctattattctct 315174

||||||||||||||||||||||||||||||||||||||||||||||||||||||||||||

Sbjct 83724 ACTATTTGGGTGCGAACTGCGGACGTTTCACTTAGTAGTTTGGCTTATCTATTATTCTCT 83783

Query 315175 cttatcttctcttctctactcttctctGAACTTCATAGTTAGGGCCGGTCTTTTATCCGA 315234

||||||||||||||||||||||||||||||||||||||||||||||||||||||||||||

Sbjct 83784 CTTATCTTCTCTTCTCTACTCTTCTCTGAACTTCATAGTTAGGGCCGGTCTTTTATCCGA 83843

Query 315235 AAGTCTTTTAGTTCCTTCCCTTTGTTTGGTCATTTTAGGATTTTACCTTCTCACATAATG 315294

||||||||||||||||||||||||||||||||||||||||||||||||||||||||||||

Sbjct 83844 AAGTCTTTTAGTTCCTTCCCTTTGTTTGGTCATTTTAGGATTTTACCTTCTCACATAATG 83903

Query 315295 ATGACTTATGTCATGGTACAGTTCCGTATTACTTGTAGCATGGTGAACATCCGTATTATG 315354

||||||||||||||||||||||||||||||||||||||||||||||||||||||||||||

Sbjct 83904 ATGACTTATGTCATGGTACAGTTCCGTATTACTTGTAGCATGGTGAACATCCGTATTATG 83963

Query 315355 AATTCTTTAATCGTTCGTAAAAGAGCTTCTCTCATTTCTTTATTCTCTTTGAATGTTCCT 315414

||||||||||||||||||||||||||||||||||||||||||||||||||||||||||||

Sbjct 83964 AATTCTTTAATCGTTCGTAAAAGAGCTTCTCTCATTTCTTTATTCTCTTTGAATGTTCCT 84023

Query 315415 TTTAGATACTCCTCTTACTCGCTGCGCGCCTTGCTACTGTCTAATACTATCGCAACTGGC 315474

||||||||||||||||||||||||||||||||||||||||||||||||||||||||||||

Sbjct 84024 TTTAGATACTCCTCTTACTCGCTGCGCGCCTTGCTACTGTCTAATACTATCGCAACTGGC 84083

Query 315475 CTACTCCCCTCGCTCGTCCCCTCGCTCGCTACTGTATTGCTGGCCTAGCCACAAAACAAC 315534

|||||||||||||||| |||||||||||||||||||||||||||||||||||||||||||

Sbjct 84084 CTACTCCCCTCGCTCGCCCCCTCGCTCGCTACTGTATTGCTGGCCTAGCCACAAAACAAC 84143

Query 315535 CTTTACTATAGCCAGCCAAGCACTTTTGCAACTTCCTCATCTCTTAATCAGAAATTCTTT 315594

||||||||||||||||||||||||||||||||||||||||||||||||||||||||||||

Sbjct 84144 CTTTACTATAGCCAGCCAAGCACTTTTGCAACTTCCTCATCTCTTAATCAGAAATTCTTT 84203

Query 315595 CtttttttGAATCGAAGAGTTCTCTTCGGAATGGCACAAAGCATGATGTGCAACGTGAAT 315654

||||||||||||||||||||||||||||||||||||||||||||||||||||||||||||

Sbjct 84204 CTTTTTTTGAATCGAAGAGTTCTCTTCGGAATGGCACAAAGCATGATGTGCAACGTGAAT 84263

Query 315655 CGGTGTTCGATGCTTTTCTTTAAACTGGTTTTTTGCCTCGAATGGCTGCCTGGTCCAAAG 315714

||||||||||||||||||||||||||||||||||||||||||||||||||||||||||||

Sbjct 84264 CGGTGTTCGATGCTTTTCTTTAAACTGGTTTTTTGCCTCGAATGGCTGCCTGGTCCAAAG 84323

Query 315715 GGCGGATTATTGGAGGAGATAGAAATGAACATGATGGTGGCTGTGATCAAGCACCTGATG 315774

||||||||||||||||||||||||||||||||||||||||||||||||||||||||||||

Sbjct 84324 GGCGGATTATTGGAGGAGATAGAAATGAACATGATGGTGGCTGTGATCAAGCACCTGATG 84383

Query 315775 AGAGTTTTAAGGAAATGAACGTCTGCAAGGCAAAAAAGAAGCCCATCCCTTCGTCCACAA 315834

||||||||||||||||||||||||||||||||||||||||||||||||||||||||||||

Sbjct 84384 AGAGTTTTAAGGAAATGAACGTCTGCAAGGCAAAAAAGAAGCCCATCCCTTCGTCCACAA 84443

Query 315835 AATCTTCTCTTCCAGGATGTGCAGTTGCTCTAATGGTGTGTGTGTATCTTCAACCAAATT 315894

||||||||||||||||||||||||||||||||||| |||||||||||||||||||||

Sbjct 84444 AATCTTCTCTTCCAGGATGTGCAGTTGCTCTAATG----GTGTGTATCTTCAACCAAATT 84499

Query 315895 GCATTATTCTACTTTTCACTAATGATGTCTAACACCTGATGGTCTGGTCCTAAAAAACTT 315954

||||||||||||||||||||||||||||||||||||||||||||||||||||||||||||

Sbjct 84500 GCATTATTCTACTTTTCACTAATGATGTCTAACACCTGATGGTCTGGTCCTAAAAAACTT 84559

Query 315955 TAATGTTTCAGCCGGGTCAATATTTCCAGCAATGttttttctttttCTATTTGTATGTAT 316014

||||||||||||||||||||||||||||||||||||||||||||||||||||||||||||

Sbjct 84560 TAATGTTTCAGCCGGGTCAATATTTCCAGCAATGTTTTTTCTTTTTCTATTTGTATGTAT 84619

Query 316015 TGTTGCTTATGGTTACAGTCTGTTGGTGTCAAATGTGTAGTCTATGGTTATATACACAGT 316074

|||||||||||||||||||||||||||||||||||||||||||||||||||||| |||||

Sbjct 84620 TGTTGCTTATGGTTACAGTCTGTTGGTGTCAAATGTGTAGTCTATGGTTATATAAACAGT 84679

Query 316075 AGTTCTCTTGGTATGAAATGTTGCTGGAATTCTCTATTAATTCTAGCTTTTGCATTGACG 316134

||||||||||||||||||||||||||||||||||||||||||||||||||||||||||||

Sbjct 84680 AGTTCTCTTGGTATGAAATGTTGCTGGAATTCTCTATTAATTCTAGCTTTTGCATTGACG 84739

Query 316135 AACAATGTTTGATTTTTTATAAGGTGTGGTTCTTTGAGCACTTAAGGCCAAGGAAATTGG 316194

||||||||||||||||||||||||||||||||||||||||||||||||||||||||||||

Sbjct 84740 AACAATGTTTGATTTTTTATAAGGTGTGGTTCTTTGAGCACTTAAGGCCAAGGAAATTGG 84799

Query 316195 AGAAAGGTACTGGACGTATTCCTAGGTACCTAAATTGGAAATTCGAGCCCAGGGAAAGAT 316254

||||||||||||||||||||||||||||||||||||||||||||||||||||||||||||

Sbjct 84800 AGAAAGGTACTGGACGTATTCCTAGGTACCTAAATTGGAAATTCGAGCCCAGGGAAAGAT 84859

Query 316255 TTCGAAAACTGCTCGTAGAAGTCCCTGCTGGCCAAGTCATTTCTGACTCAATCAATAAAG 316314

||||||||||||||||||||||||||||||||||||||||||||||||||||||||||||

Sbjct 84860 TTCGAAAACTGCTCGTAGAAGTCCCTGCTGGCCAAGTCATTTCTGACTCAATCAATAAAG 84919

Query 316315 TGTTCGGTGCCTGAAAAGCTAATAAGCTTAACTGGATTTCGTGCATCTAAGTATGTTGCA 316374

||||||||||||| |||||||||||||||||||||||||||||||||||||||||||

Sbjct 84920 TGTTCGGTGCCTG----GCTAATAAGCTTAACTGGATTTCGTGCATCTAAGTATGTTGCA 84975

Query 316375 ATTCTTCATCATGAAAGTGATTTTCGCAAACACCACAATCAAGATTTAGCATACCTACTC 316434

||||||||||||||||||||||||||||||||||||||||||||||||||||||| ||||

Sbjct 84976 ATTCTTCATCATGAAAGTGATTTTCGCAAACACCACAATCAAGATTTAGCATACCCACTC 85035

Query 316435 CAGCAAATGGCCCCAATTTCTAGCAAGTTTCATTTCCCTATTTGCCCACTATGTAATGGA 316494

||||||||||||||||||||||||||||||||||||||||||||||||||||||||||||

Sbjct 85036 CAGCAAATGGCCCCAATTTCTAGCAAGTTTCATTTCCCTATTTGCCCACTATGTAATGGA 85095

Query 316495 TGAAGTTACCGGGCTCGAAAGTCTGGAATATTCACCAAAACATGTGCCTGTTGAGGGTTT 316554

||||||||||||||||||||||||||||||||||||||||||||||||||||||||||||

Sbjct 85096 TGAAGTTACCGGGCTCGAAAGTCTGGAATATTCACCAAAACATGTGCCTGTTGAGGGTTT 85155

Query 316555 GGATGAGGTCACCCCAACCTTGGAAGCAGCATTCTGCCAGGAAGCTTCGAGGGTCGAGAT 316614

||||||||||||||||||||||||||||||||||||||||||||||||||||||||||||

Sbjct 85156 GGATGAGGTCACCCCAACCTTGGAAGCAGCATTCTGCCAGGAAGCTTCGAGGGTCGAGAT 85215

Query 316615 AGAACTCGAGATTCACAGGGACCAGAAGAATGATCCTACAATGAAG----CCAAAGGTAT 316670

|||||||||||||||||||||||||||||||||||||||||||||| ||||||||||

Sbjct 85216 AGAACTCGAGATTCACAGGGACCAGAAGAATGATCCTACAATGAAGCGTTCCAAAGGTAT 85275

Query 316671 ATAAATCGAATATTTTCTATTTCTTAATGCGTGAGTGGAAAAAGGCTATAGTCAAATTAA 316730

||||||||||||||||||||||||||||||||||||||||||||||||||||||||||||

Sbjct 85276 ATAAATCGAATATTTTCTATTTCTTAATGCGTGAGTGGAAAAAGGCTATAGTCAAATTAA 85335

Query 316731 GTAGTGACAACTTACATCTTAATCTTTCTGTCAGTGGCTCATGAAATTGGGGCTGATTGT 316790

||||||||||||||||||||||||||||||||||||||||||||||||||||||||||||

Sbjct 85336 GTAGTGACAACTTACATCTTAATCTTTCTGTCAGTGGCTCATGAAATTGGGGCTGATTGT 85395

Query 316791 GACGTTTCCCAGAAGAGTGTTTCTATGTGTGCATCAGAAGCGCCATCCCGGGAAATTTAT 316850

||||||||||||||||||||||||||||||||||||||||||||||||||||||||||||

Sbjct 85396 GACGTTTCCCAGAAGAGTGTTTCTATGTGTGCATCAGAAGCGCCATCCCGGGAAATTTAT 85455

Query 316851 ATCAAATAAGCTGAAACTCCGTCCATTAGGACCTCTCCTAGACTCTATCAGGCCTCATCA 316910

||||||||||||||||||||||||||||||||||||||||||||||||||||||||||||

Sbjct 85456 ATCAAATAAGCTGAAACTCCGTCCATTAGGACCTCTCCTAGACTCTATCAGGCCTCATCA 85515

Query 316911 AAGCAACAAACTCCAAGCACTCCAATCACCCGATCTCTGAGTCGAATCCAGCACAACAAC 316970

||||||||||||||||||||||||||||||||||||||||||||||||||||||||||||

Sbjct 85516 AAGCAACAAACTCCAAGCACTCCAATCACCCGATCTCTGAGTCGAATCCAGCACAACAAC 85575

Query 316971 ATCATGGAAGTAACGATAGGCAGCTCTACCTCGACCATCAGCCCAAAGAACATTTACTAA 317030

||||||||||| ||||||||||||||||||||||||||||||||||||||||||||||||

Sbjct 85576 ATCATGGAAGTTACGATAGGCAGCTCTACCTCGACCATCAGCCCAAAGAACATTTACTAA 85635

Query 317031 CTAGACGACCATCTTTGTCGAAGTTGTATGAGAAAAAGAATTGAGGATCCTTCATCATCT 317090

||||||||||||||||||||||||||||||||||||||||||||||||||||||||||||

Sbjct 85636 CTAGACGACCATCTTTGTCGAAGTTGTATGAGAAAAAGAATTGAGGATCCTTCATCATCT 85695

Query 317091 TCTTCTCAACCATCAAAACAGTTGCATCGTTACCGGGCAGTCGCTTAGCTTTCTCCCTTC 317150

||||||||||||||||||||||||||||||||||||||||||||||||||||||||||||

Sbjct 85696 TCTTCTCAACCATCAAAACAGTTGCATCGTTACCGGGCAGTCGCTTAGCTTTCTCCCTTC 85755

Query 317151 TATACTTGTCAACATGATTTCTAGCATCTTTACGGGTGAATGTAGCCTTCCCGTATCCAC 317210

||||||||||||||||||||||||||||||||||||||||||||||||||||||||||||

Sbjct 85756 TATACTTGTCAACATGATTTCTAGCATCTTTACGGGTGAATGTAGCCTTCCCGTATCCAC 85815

Query 317211 CAGCATGCTTAATGACAGTGGTAATATTCAACCAAGCGGGCATCGCATCATCATCATTTG 317270

||||||||||||||||||||||||||||||||||||||||||||||||||||||||||||

Sbjct 85816 CAGCATGCTTAATGACAGTGGTAATATTCAACCAAGCGGGCATCGCATCATCATCATTTG 85875

Query 317271 TCTCCAATTCTTTCTTAAAACGAAGAGGCGCGGAGCGATATGTGGTACTCAGATATGAAC 317330

||||||||||||||||||||||||||||||||||||||||||||||||||||||||||||

Sbjct 85876 TCTCCAATTCTTTCTTAAAACGAAGAGGCGCGGAGCGATATGTGGTACTCAGATATGAAC 85935

Query 317331 TGTCTGGTGTTAACACATGATTGTGCTCCAAATTCACTTTAGTAATAACTCTTCGATTTA 317390

||||||||||||||||||||||||||||||||||||||||||||||||||||||||||||

Sbjct 85936 TGTCTGGTGTTAACACATGATTGTGCTCCAAATTCACTTTAGTAATAACTCTTCGATTTA 85995

Query 317391 GGAAACCCGGATCAGATATTGTTAACATTGCTTTGCATTCAGTACCAAGAACAGGCCTAT 317450

||||||||||||||||||||||||||||||||||||||||||||||||||||||||||||

Sbjct 85996 GGAAACCCGGATCAGATATTGTTAACATTGCTTTGCATTCAGTACCAAGAACAGGCCTAT 86055

Query 317451 TTCTCTCTGGTAATGGCGGCACATTACTCTCTCTACCGGCTTCTTGTGTTTGTTGCAAGC 317510

||||||||||||||||||||||||||||||||||||||||||||||||||||||||||||

Sbjct 86056 TTCTCTCTGGTAATGGCGGCACATTACTCTCTCTACCGGCTTCTTGTGTTTGTTGCAAGC 86115

Query 317511 AAAGGAATCATGGTAAAATGCACCAGCCCTTTTTGTGGTTTGACCTTTTCACAATACCAA 317570

||||||||||||||||||||||||||||||||||||||||||||||||||||||||||||

Sbjct 86116 AAAGGAATCATGGTAAAATGCACCAGCCCTTTTTGTGGTTTGACCTTTTCACAATACCAA 86175

Query 317571 AACCTTTTCTCAACCCATAATCTTTGTAGAACTGGTACACATCATCAGAAGAGTCAAATT 317630

||||||||||||||||||||||||||||||||||||||||||||||||||||||||||||

Sbjct 86176 AACCTTTTCTCAACCCATAATCTTTGTAGAACTGGTACACATCATCAGAAGAGTCAAATT 86235

Query 317631 CCATACCAACCTCTGGTTCTAGAATCTCAGTGATACCTACATCATCGTATTCTGGATGGT 317690

||||||||||||||||||||||||||||||||||||||||||||||||||||||||||||

Sbjct 86236 CCATACCAACCTCTGGTTCTAGAATCTCAGTGATACCTACATCATCGTATTCTGGATGGT 86295

Query 317691 CATCCTGTTGTGTAGCCTCTTGTGTACCCCCTAATACACCCTCTTCTCTACCTTCTTGTA 317750

||||||||||||||||||||||||||||||||||||||||||||||||||||||||||||

Sbjct 86296 CATCCTGTTGTGTAGCCTCTTGTGTACCCCCTAATACACCCTCTTCTCTACCTTCTTGTA 86355

Query 317751 TACCATCTTGTCTACCCTCACTGGGATTGTCACTTGCACCAAAAAGAGCATTTCAATCAT 317810

||||||||||||||||||||||||||||||||||||||||||||||||||||||||||||

Sbjct 86356 TACCATCTTGTCTACCCTCACTGGGATTGTCACTTGCACCAAAAAGAGCATTTCAATCAT 86415

Query 317811 CTTCTTTTATTCTCAGCAACAACCACCGGTACGAGAGACGTATTCAGATCAAAATTCTTC 317870

||||||||||||||||||||||||||||||||||||||||||||||||||||||||||||

Sbjct 86416 CTTCTTTTATTCTCAGCAACAACCACCGGTACGAGAGACGTATTCAGATCAAAATTCTTC 86475

Query 317871 ACAATCCTAACAAGAAACACAAATAGAGTATAATATTAATCAGGCttttttttACAGGGG 317930

|||||||||||||||||||||||||||||||||||||||||||||||||||||||||| |

Sbjct 86476 ACAATCCTAACAAGAAACACAAATAGAGTATAATATTAATCAGGCTTTTTTTTACAGGAG 86535

Query 317931 AATTCAGAAAAATGTACAGACATGAAAGAATTATGTCAGTCAGATAGTTCATTAAGTGCA 317990

||||||||||||||||||||||||||||||||||||||||||||||||||||||||||||

Sbjct 86536 AATTCAGAAAAATGTACAGACATGAAAGAATTATGTCAGTCAGATAGTTCATTAAGTGCA 86595

Query 317991 TCAGGTGTTTATATAGGTGTCCTAGGTAGATATATCCTAGGTGTTTAGATATATCAACAC 318050

||||||||||||||||||||||||||||||||||||||||||||||||||||||||||||

Sbjct 86596 TCAGGTGTTTATATAGGTGTCCTAGGTAGATATATCCTAGGTGTTTAGATATATCAACAC 86655

Query 318051 GTGTACATATCAGTAAAATAGTTAAGTGTTCATCATATGAATCAGGAATGTTTAtttttt 318110

||||||||||||||||||||||||||||||||||||||||||||||||||||||||||||

Sbjct 86656 GTGTACATATCAGTAAAATAGTTAAGTGTTCATCATATGAATCAGGAATGTTTATTTTTT 86715

Query 318111 tCATAAAAAATCAGCACTAAATACATAACTAAATCCCTAAATCATAATCAACATAATATT 318170

||||||||||||||||||||||||||||||||||||||||||||||||||||||||||||

Sbjct 86716 TCATAAAAAATCAGCACTAAATACATAACTAAATCCCTAAATCATAATCAACATAATATT 86775

Query 318171 CTTCATATACATGTCGTGTTTACATAAATCAAATAGTATTTTTCAAGCTGTAAAACATGT 318230

||||||||||||||||||||||||||||||||||||||||||||||||||||||||||||

Sbjct 86776 CTTCATATACATGTCGTGTTTACATAAATCAAATAGTATTTTTCAAGCTGTAAAACATGT 86835

Query 318231 GTTTAGATGAAAAATATAGAATAATATGATCTACACAGACCGATCTATAACAAAACAGAG 318290

||||||||||||||||||||||||||||||||||||||||||||||||||||||||||||

Sbjct 86836 GTTTAGATGAAAAATATAGAATAATATGATCTACACAGACCGATCTATAACAAAACAGAG 86895

Query 318291 ATATATTATCAACAAAACAAGATTTATATGAATAAACATCAATATAACCAGATGTATATC 318350

||||||||||||||||||||||||||||||||||||||||||||||||||||||||||||

Sbjct 86896 ATATATTATCAACAAAACAAGATTTATATGAATAAACATCAATATAACCAGATGTATATC 86955

Query 318351 AGCAATCAGATCTACATAAGCAGATCTAACATAATAAAAAATCAGATGTAAAATAATCAA 318410

||||||||||||||||||||||||||||||||||||||||||||||||||||||||||||

Sbjct 86956 AGCAATCAGATCTACATAAGCAGATCTAACATAATAAAAAATCAGATGTAAAATAATCAA 87015

Query 318411 ATCTAAGCATGTTTATTGTAGGAAAAGAAAGAAAAGAGATCAGTTGATGAATATCACGAA 318470

||||||||||||||||||||||||||||||||||||||||||||||||||||||||||||

Sbjct 87016 ATCTAAGCATGTTTATTGTAGGAAAAGAAAGAAAAGAGATCAGTTGATGAATATCACGAA 87075

Query 318471 ATTGAATTGAAATACTTGAATGAATACAAAACAACATGCTAGAACACAATAAAATTCTaa 318530

|||||||||||||||||||||||||||||||||||||||||||||||||||||||||| |

Sbjct 87076 ATTGAATTGAAATACTTGAATGAATACAAAACAACATGCTAGAACACAATAAAATTCT-A 87134

Query 318531 aaaaaaaaTAAACAGAAGAGGTAAATTAATCAACTAATTGATGAACAGATCTCAAAACAT 318590

|||||||||||| |||||||||||||||||||||||||||||||||||||||||||||||

Sbjct 87135 AAAAAAAATAAAAAGAAGAGGTAAATTAATCAACTAATTGATGAACAGATCTCAAAACAT 87194

Query 318591 GCTGAATTGAACGATTTGCACAATAGAAAAGCTTGAATTGAAATGATCTGCACAATAGAA 318650

||||||||||||||||||||||||||||||||||||||||||||||||||||||||||||

Sbjct 87195 GCTGAATTGAACGATTTGCACAATAGAAAAGCTTGAATTGAAATGATCTGCACAATAGAA 87254

Query 318651 AAGTCAGAAAACATACCTAGATATATTTTTAAGATCAAAGGATGAAAATAAGCTTCAATT 318710

||||||||||||||||||||||||||||||||||||||||||||||||||||||||||||

Sbjct 87255 AAGTCAGAAAACATACCTAGATATATTTTTAAGATCAAAGGATGAAAATAAGCTTCAATT 87314

Query 318711 GAACGATTTAAACATCCAGAGAGTTGAATTACGCTggaggagacaccagaggagaagcag 318770

||||||||||||||||||||||||||||||||||||||||||||||||||||||||||||

Sbjct 87315 GAACGATTTAAACATCCAGAGAGTTGAATTACGCTGGAGGAGACACCAGAGGAGAAGCAG 87374

Query 318771 agagatcgccagtgagagaaagaagagagcatcggaagaagaaagatcgccaggggagaa 318830

||||||||||||||||||||||||||||||||||||||||||||||||||||||||||||

Sbjct 87375 AGAGATCGCCAGTGAGAGAAAGAAGAGAGCATCGGAAGAAGAAAGATCGCCAGGGGAGAA 87434

Query 318831 agagagatcgagTGCCAGATGAGGAAAGAGCGAAATTCAAATCAGAGTAACGAGAAAAGG 318890

||||||||||||||||||||||||||||||||||||||||||||||||||||||||||||

Sbjct 87435 AGAGAGATCGAGTGCCAGATGAGGAAAGAGCGAAATTCAAATCAGAGTAACGAGAAAAGG 87494

Query 318891 TCGGTAACGTACTACTCTTTCTTCCCTTCGTCCGCAAACGTACTATTCTACTCGTGGTGA 318950

||||||||||||||||||||||||||||||||||||||||||||||||||||||||||||

Sbjct 87495 TCGGTAACGTACTACTCTTTCTTCCCTTCGTCCGCAAACGTACTATTCTACTCGTGGTGA 87554

Query 318951 CGTCCGGAATATAATCCACTTTCCTCCCACTGAAGAACCGAATCAATATGAGACTTTGCT 319010

||||||||||||||||||||||||||||||||||||||||||||||||||||||||||||

Sbjct 87555 CGTCCGGAATATAATCCACTTTCCTCCCACTGAAGAACCGAATCAATATGAGACTTTGCT 87614

Query 319011 GTACCCAGGACCGTGACTGCTACCTCCTTATCCCATAGCTATGCTAACCCTCCAGGTTCC 319070

||||||||||||||||||||||||||||||||||||||||||||||||||||||||||||

Sbjct 87615 GTACCCAGGACCGTGACTGCTACCTCCTTATCCCATAGCTATGCTAACCCTCCAGGTTCC 87674

Query 319071 GGTATAGGGCCTAGCCACAAAACAACCTTTACTATAGCCAGCCAAGCACTTTTGCAACTT 319130

||||||||||||||||||||||||||||||||||||||||||||||| ||||||||||||

Sbjct 87675 GGTATAGGGCCTAGCCACAAAACAACCTTTACTATAGCCAGCCAAGCTCTTTTGCAACTT 87734

Query 319131 CTTCCATTCACATTATTCATTCTAGCATGATAATATAGTCTTCTCCTTAATAAATAATCA 319190

||||||||||||||||||||||||||||||||||||||||||||||||||||||||||||

Sbjct 87735 CTTCCATTCACATTATTCATTCTAGCATGATAATATAGTCTTCTCCTTAATAAATAATCA 87794

Query 319191 AGCAAGAAATCAAGCTTAGCGCCCTCCAGTCTCGTTCGCTGTTATAGGCTTATGTTGGAT 319250

||||||||||||||||||||||||||||||||||||||||||||||||||||||||||||

Sbjct 87795 AGCAAGAAATCAAGCTTAGCGCCCTCCAGTCTCGTTCGCTGTTATAGGCTTATGTTGGAT 87854

Query 319251 GTCGCAACGTTTATATGTCTATAtttttcctgttttttccttttCTAATCCGATTCCTAC 319310

||||||||||||||||||||||||||||||||||||||||||||||||||||||||||||

Sbjct 87855 GTCGCAACGTTTATATGTCTATATTTTTCCTGTTTTTTCCTTTTCTAATCCGATTCCTAC 87914

Query 319311 TTTCTATTCTGGATAACTTGTTAGAGCATTTAGCTAGCCTGGTATAGTAAAGTAAAGATT 319370

||||||||||||||||||||||||||||||||||||||||||||||||||||||||||||

Sbjct 87915 TTTCTATTCTGGATAACTTGTTAGAGCATTTAGCTAGCCTGGTATAGTAAAGTAAAGATT 87974

Query 319371 TTAAGGCTTGTGGAAGAATAGATTCGATTCGGAATTCGCCTACTAGTAACCATAGCCCAA 319430

||||||||||||||||||||||||||||||||||||||||||||||||||||||||||||

Sbjct 87975 TTAAGGCTTGTGGAAGAATAGATTCGATTCGGAATTCGCCTACTAGTAACCATAGCCCAA 88034

Query 319431 ACCACAAGTTTGGTATTTCTAGTCAAAAGGTAttttttttACCCAGCTGAAATTCCTTTA 319490

||||||||||||||||||||||||||||||||||||||||||||||||||||||||||||

Sbjct 88035 ACCACAAGTTTGGTATTTCTAGTCAAAAGGTATTTTTTTTACCCAGCTGAAATTCCTTTA 88094

Query 319491 CTCCCCTGTCTTTCCCGGATCAGATTATGCTTTCTATGCCCCTTTCCCTTATTCTATTCA 319550

||||||||||||||||||||||||||||||||||||||||||||||||||||||||||||

Sbjct 88095 CTCCCCTGTCTTTCCCGGATCAGATTATGCTTTCTATGCCCCTTTCCCTTATTCTATTCA 88154

Query 319551 GGAAGTTATTTTCGCCGTCTTTCAAAATCCTTCTTTGACGAAAGACATAGCGTGTCACGC 319610

||||||||||||||||||||||||||||||||||||||||||||||||||||||||||||

Sbjct 88155 GGAAGTTATTTTCGCCGTCTTTCAAAATCCTTCTTTGACGAAAGACATAGCGTGTCACGC 88214

Query 319611 TGGaaaaaaaCTAGAATATTTTCAACAAGTCAGTGTTCAAGTTCTATTATCTTGAAAGGC 319670

||||||||||||||||||||||||||||||||||||||||||||||||||||||||||||

Sbjct 88215 TGGAAAAAAACTAGAATATTTTCAACAAGTCAGTGTTCAAGTTCTATTATCTTGAAAGGC 88274

Query 319671 TTTCAATAGCATTACAGAAAGAGAAGCCAGAACCTACTACCAGTTGGCCAGCATCAAAGC 319730

||||||||||||||||||||||||||||||||||||||||||||||||||||||||||||

Sbjct 88275 TTTCAATAGCATTACAGAAAGAGAAGCCAGAACCTACTACCAGTTGGCCAGCATCAAAGC 88334

Query 319731 AGAAGGAGCTTTACCCCAACCCAAAGTTTGGAATTAAAGAAAGACTCTATTAAAGAGCCG 319790

||||||||||||||||||||||||||||||||||||||||||||||||||||||||||||

Sbjct 88335 AGAAGGAGCTTTACCCCAACCCAAAGTTTGGAATTAAAGAAAGACTCTATTAAAGAGCCG 88394

Query 319791 GGAGTTTAGATTTCATACATAGACTTTAGATTACTAATGAAGTCACCGGCATGTGATTAT 319850

||||||||||||||||||||||||||||||||||||||||||||||||||||||||||||

Sbjct 88395 GGAGTTTAGATTTCATACATAGACTTTAGATTACTAATGAAGTCACCGGCATGTGATTAT 88454

Query 319851 TAACACTTGCGCACAACTAAAAAATGCTCCCCTACTGAGCAATTTTGGAAATGGTTACTG 319910

||||||||||||||||||||||||||||||||||||||||||||| ||||||||||||||

Sbjct 88455 TAACACTTGCGCACAACTAAAAAATGCTCCCCTACTGAGCAATTTGGGAAATGGTTACTG 88514

Query 319911 GCAGTAGATGAAGTAAAGAGGGAGCCACTACACTAGTAGGCCAGCAAGTTTTCAAGCCAC 319970

||||||||||||||||||||||||||||||||||||||||||||||||||||||||||||

Sbjct 88515 GCAGTAGATGAAGTAAAGAGGGAGCCACTACACTAGTAGGCCAGCAAGTTTTCAAGCCAC 88574

Query 319971 ATGAGAGTGAATTTTCTTTCCATTTCAAAGCGGGAAAAGCGGGTAACGCGTTGCACAGGC 320030

||||||||||||||||||||||||||||||||||||||||||||||||||||||||||||

Sbjct 88575 ATGAGAGTGAATTTTCTTTCCATTTCAAAGCGGGAAAAGCGGGTAACGCGTTGCACAGGC 88634

Query 320031 TAATAGTTGACTGAGAATAGAATACCATACATACGGACCACGTAAGAATTCGATATTAAA 320090

||||||||||||||||||||||||||||||||||||||||||||||||||||||||||||

Sbjct 88635 TAATAGTTGACTGAGAATAGAATACCATACATACGGACCACGTAAGAATTCGATATTAAA 88694

Query 320091 ACCCCACGCTTCAGACATAAGAGAGGAAAAAATGATAAAAGGTTTGAACAACCAACCTTA 320150

||||||||||||||||||||||||||||||||||||||||||||||||||||||||||||

Sbjct 88695 ACCCCACGCTTCAGACATAAGAGAGGAAAAAATGATAAAAGGTTTGAACAACCAACCTTA 88754

Query 320151 CATAAGGCGCGCAGCGGTTCCTTACGTTTGGGAAAATCATTTTGTAAAGAAGGGTGCGTT 320210

||||||||||||||||||||||||||||||||||||||||||||||||||||||||||||

Sbjct 88755 CATAAGGCGCGCAGCGGTTCCTTACGTTTGGGAAAATCATTTTGTAAAGAAGGGTGCGTT 88814

Query 320211 GCATTTGGTCTAGCATCAATATCATATCCCTACCTCTCACTCATTGTTGCTTCTTTACGA 320270

||||||||||||||||||||||||||||||||||||||||||||||||||||||||||||

Sbjct 88815 GCATTTGGTCTAGCATCAATATCATATCCCTACCTCTCACTCATTGTTGCTTCTTTACGA 88874

Query 320271 TGAGTTCTTTCTATTGGTAACTGCATTGCTTCACTCTGCTCCCAGCTTGCATTCACCCTC 320330

||||||||||||||||||||||||||||||||||||||||||||||||||||||||||||

Sbjct 88875 TGAGTTCTTTCTATTGGTAACTGCATTGCTTCACTCTGCTCCCAGCTTGCATTCACCCTC 88934

Query 320331 TACTTGACCATGACTCCGGGTACTGAGTTCTTCTTTCTTTTAGATGGGATTTTCCCCTGT 320390

||||||||||||||||||||||||||||||||||||||||||||||||||||||||||||

Sbjct 88935 TACTTGACCATGACTCCGGGTACTGAGTTCTTCTTTCTTTTAGATGGGATTTTCCCCTGT 88994

Query 320391 CTAATCTGTTGACTTTGTTTCCAAGCTAGCTAGATATTACTCCG----ATATTACGGATA 320446

|||||||||||||||||||||||||||||||||||||||||||| ||||||||||||

Sbjct 88995 CTAATCTGTTGACTTTGTTTCCAAGCTAGCTAGATATTACTCCGATATATATTACGGATA 89054

Query 320447 ATTTCGAGCTAATTTTAGCAATTATGATACTGTCGTACCAGACCAAACAGTACCAGCACA 320506

||||||||||||||||||||||||||||||||||||||||||||||||||||||||||||

Sbjct 89055 ATTTCGAGCTAATTTTAGCAATTATGATACTGTCGTACCAGACCAAACAGTACCAGCACA 89114

Query 320507 AGATAATTGGACgagaaattatagaattaaggaaagatagagaaggagaaagagacagag 320566

||| ||||||||||||||||||||||||||||||||||||||||||||||||||||||||

Sbjct 89115 AGAGAATTGGACGAGAAATTATAGAATTAAGGAAAGATAGAGAAGGAGAAAGAGACAGAG 89174

Query 320567 aaaagaagaTTTCAAATCCAAATCAGAAGGAAGCTAGCCCAGCACACTTAACATAAATTT 320626

||||||||||||||||||||||||||||||||||||||||||||||||||||||||||||

Sbjct 89175 AAAAGAAGATTTCAAATCCAAATCAGAAGGAAGCTAGCCCAGCACACTTAACATAAATTT 89234

Query 320627 TAGTGTCAGCTGTTCAAATCTTTCACTGCTGCAGGTGGAAGGAAGTGGTCATTTTTAAGG 320686

||||||||||||||||||||||||||||||||||||||||||||||||||||||||||||

Sbjct 89235 TAGTGTCAGCTGTTCAAATCTTTCACTGCTGCAGGTGGAAGGAAGTGGTCATTTTTAAGG 89294

Query 320687 TAACTACGTACAAGAAAGAAATCCGTTACCGCTCAAGTTGATGAGACTAGTGTCGACCAA 320746

||||||||||||||||||||||||||||||||||||||||||||||||||||||||||||

Sbjct 89295 TAACTACGTACAAGAAAGAAATCCGTTACCGCTCAAGTTGATGAGACTAGTGTCGACCAA 89354

Query 320747 GAAGCCTCTTTCAGTCCGCCCATTCTACATGTATGGCATAACTTTTTTATCTGAACAACT 320806

||||||||||||||||||||||||||||||||||||||||||||||||||||||||||||

Sbjct 89355 GAAGCCTCTTTCAGTCCGCCCATTCTACATGTATGGCATAACTTTTTTATCTGAACAACT 89414

Query 320807 TCTTTCGTTGAAATTGACACATTGCTGCTTTCCTTCTTCTTTCAAATGTAAGTACTACTT 320866

||||||||||||||||||||||||||||||||||||||||||||||||||||||||||||

Sbjct 89415 TCTTTCGTTGAAATTGACACATTGCTGCTTTCCTTCTTCTTTCAAATGTAAGTACTACTT 89474

Query 320867 TCCTTCTCTGGCTTTGATGGGATTCTTCTTAGAAACCAAATGCTTCGCCATGACCGTGGA 320926

||||||||||||||||||||||||||||||||||||||||||||||||||||||||||||

Sbjct 89475 TCCTTCTCTGGCTTTGATGGGATTCTTCTTAGAAACCAAATGCTTCGCCATGACCGTGGA 89534

Query 320927 GACTTAAGCCTCTTTATGAGGAAGGGTGAACTTTACCAGTTGGACGTCAGGTAGGAATAA 320986

||||||||||||||||||||||||||||||||||||||||||||||||||||||||||||

Sbjct 89535 GACTTAAGCCTCTTTATGAGGAAGGGTGAACTTTACCAGTTGGACGTCAGGTAGGAATAA 89594

Query 320987 ACTTATGACGTTTCGGTCGGGACTCGTTGAAGCGAGCGGGTCACTAGCCTCACTAGCTAA 321046

||||||||||||||||||||||||||||||||||||||||||||||||||||||||||||

Sbjct 89595 ACTTATGACGTTTCGGTCGGGACTCGTTGAAGCGAGCGGGTCACTAGCCTCACTAGCTAA 89654

Query 321047 AAGTATCTGCCCGGGTAAAAGGTGAAGTGGATCATGCATCAGTGAGTATCAAT--TC--G 321102

||||||||||||||||||||||||||||||||||||||||||||||||||||| || |

Sbjct 89655 AAGTATCTGCCCGGGTAAAAGGTGAAGTGGATCATGCATCAGTGAGTATCAATAATCTAG 89714

Query 321103 ATGGGATGAAGGACGAGTTGATCGAGTATATATAAGGAAGCAATAAATCTATCTGCTGGG 321162

||||||||||||||||||||||||||||||||||||||||||||||||||||||||||||

Sbjct 89715 ATGGGATGAAGGACGAGTTGATCGAGTATATATAAGGAAGCAATAAATCTATCTGCTGGG 89774

Query 321163 AGGGGTCATACTTAGTTGATAAGCTAGGGAGCAGGGTTTGTGTTTTTGTCCTTGATAGAA 321222

||||||||||||||||||||||||||||||||||||||||||||||||||||||||||||

Sbjct 89775 AGGGGTCATACTTAGTTGATAAGCTAGGGAGCAGGGTTTGTGTTTTTGTCCTTGATAGAA 89834

Query 321223 AATCGCGTGCTCCTTTTGTAGATAGAAATGAAGCCGCCGTCCTCCTACTCTCTTAACAAT 321282

||||||||||||||||||||||||||||||||||||||||||||||||||||||||||||

Sbjct 89835 AATCGCGTGCTCCTTTTGTAGATAGAAATGAAGCCGCCGTCCTCCTACTCTCTTAACAAT 89894

Query 321283 TCTCTTTATTACTAAATCAAGTTCGCTTCCATCCCTTTCATTCCTCATTCCACAACTTCG 321342

||||||||||||||||||||||||||||||||||||||||||||||||||||||||||||

Sbjct 89895 TCTCTTTATTACTAAATCAAGTTCGCTTCCATCCCTTTCATTCCTCATTCCACAACTTCG 89954

Query 321343 TTCttttttttCATATACCTCCCCACCAATAGATAGAGACAAATAGGAATAACCAAACCA 321402

||||||||||||||||||||||||||||||||||||||||||||||||||||||||||||

Sbjct 89955 TTCTTTTTTTTCATATACCTCCCCACCAATAGATAGAGACAAATAGGAATAACCAAACCA 90014

Query 321403 CGTCAACAAAATGCCAATACCATGCAGCAGCTTCAAAGCCAACGTGATGCTCCTTGGTAA 321462

||||||||||||||||||||||||||||||||||||||||||||||||||||||||||||

Sbjct 90015 CGTCAACAAAATGCCAATACCATGCAGCAGCTTCAAAGCCAACGTGATGCTCCTTGGTAA 90074

Query 321463 GATGACCAAGATACTGGCGAATACCACATATGATCAAGAAAATAGTCCCTATAATCACAT 321522

||||||||||||||||||||||||||||||||||||||||||||||||||||||||||||

Sbjct 90075 GATGACCAAGATACTGGCGAATACCACATATGATCAAGAAAATAGTCCCTATAATCACAT 90134

Query 321523 GAAAACCATGAAAGCCAGTTGCTAAGAAAAAGGTCGAACCATAAATACTATCTGAAATGG 321582

||||||||||||||||||||||||||||||||||||||||||||||||||||||||||||

Sbjct 90135 GAAAACCATGAAAGCCAGTTGCTAAGAAAAAGGTCGAACCATAAATACTATCTGAAATGG 90194

Query 321583 TGAAGGGTGCTTGGTAATATTCCATTCCTTGAAAGCCAGTGAATACTAGAGCCAGTAAAA 321642

||||||||||||||||||||||||||||||||||||||||||||||||||||||||||||

Sbjct 90195 TGAAGGGTGCTTGGTAATATTCCATTCCTTGAAAGCCAGTGAATACTAGAGCCAGTAAAA 90254

Query 321643 CGGTAGCTACTAAAGCGTAAACTGCTCGTTTTTCCTTCCCCGCGAGTATAGCATGATGAG 321702

||||||||||||||||||||||||||||||||||||||||||||||||||||||||||||

Sbjct 90255 CGGTAGCTACTAAAGCGTAAACTGCTCGTTTTTCCTTCCCCGCGAGTATAGCATGATGAG 90314

Query 321703 CCCAAGTTACGGCAGCTCCGGATGAAAGGAGAATCAGGGTATTAAGAAAAGGTATTTCCC 321762

||||||||||||||||||||||||||||||||||||||||||||||||||||||||||||

Sbjct 90315 CCCAAGTTACGGCAGCTCCGGATGAAAGGAGAATCAGGGTATTAAGAAAAGGTATTTCCC 90374

Query 321763 AAGGATCTAAAACCCCAATCCCTTTTGGGGGCCAAATACCTCCGATCTCTACCGTAGGTG 321822

||||||||||||||||||||||||||||||||||||||||||||||||||||||||||||

Sbjct 90375 AAGGATCTAAAACCCCAATCCCTTTTGGGGGCCAAATACCTCCGATCTCTACCGTAGGTG 90434

Query 321823 CCAAAGAAGAATGaaaaaaagcccaaaaaaaagcaaaaaaGAACATCACCTCCGAGACTA 321882

||||||||||||||||||||||||||||||||||||||||||||||||||||||||||||

Sbjct 90435 CCAAAGAAGAATGAAAAAAAGCCCAAAAAAAAGCAAAAAAGAACATCACCTCCGAGACTA 90494

Query 321883 TGAACAGAATAAAACCATATCGAAGTCCTAATTGTACAGCTTTTGTATGATGTCCTTCCA 321942

||||||||||||||||||||||||||||||||||||||||||||||||||||||||||||

Sbjct 90495 TGAACAGAATAAAACCATATCGAAGTCCTAATTGTACAGCTTTTGTATGATGTCCTTCCA 90554

Query 321943 ATGTGGACTCACGTAGAACATCGCGCCACCATACATACATGGTATATAGGAGAAAGATGA 322002

||||||||||||||||||||||||||||||||||||||||||||||||||||||||||||

Sbjct 90555 ATGTGGACTCACGTAGAACATCGCGCCACCATACATACATGGTATATAGGAGAAAGATGA 90614

Query 322003 GACCCAAACTTAGAAGTGTTGCACCCCCTTGAAATGAGTGCATGTACATCACACCACCTA 322062

||||||||||||||||||||||||||||||||||||||||||||||||||||||||||||

Sbjct 90615 GACCCAAACTTAGAAGTGTTGCACCCCCTTGAAATGAGTGCATGTACATCACACCACCTA 90674

Query 322063 CGGTGGTTGCCAAAGCTCCGAGTGAAGCCGAAATAGGCCATGGACTTGGATCTACCAAAT 322122

||||||||||||||||||||||||||||||||||||||||||||||||||||||||||||

Sbjct 90675 CGGTGGTTGCCAAAGCTCCGAGTGAAGCCGAAATAGGCCATGGACTTGGATCTACCAAAT 90734

Query 322123 GATAAGAATGCCTCTGAGATTCAATCATCAACCACTGTGCTCCGGTTGTATGTAAAcccc 322182

||||||||||||||||||||||||||||||||||||||||||||||||||||||||||||

Sbjct 90735 GATAAGAATGCCTCTGAGATTCAATCATCAACCACTGTGCTCCGGTTGTATGTAAACCCC 90794

Query 322183 ccttcacccccccTGGTAAAGAAGGGGGCTCCTTATTTATTTTCTTCTC-tttttttttC 322241

||||||||||||||||||||||||||||||||||||||||||||||||| ||||||||||

Sbjct 90795 CCTTCACCCCCCCTGGTAAAGAAGGGGGCTCCTTATTTATTTTCTTCTCTTTTTTTTTTC 90854

Query 322242 TCTCTGATAGGAATTGGAAGGGATAGTTCTTTTCTTTATTGCGCATTTCTAAAAGAAGTG 322301

||||||||||||||||||||||||||||||||||||||||||||||||||||||||||||

Sbjct 90855 TCTCTGATAGGAATTGGAAGGGATAGTTCTTTTCTTTATTGCGCATTTCTAAAAGAAGTG 90914

Query 322302 AGACTCTCAAACTCTCTCTTTTTATTTTTATAAGAGAATTCTTGCAAAGAGACTTTAATT 322361

||||||||||||||||||||||||||||||||||||||||||||||||||||||||||||

Sbjct 90915 AGACTCTCAAACTCTCTCTTTTTATTTTTATAAGAGAATTCTTGCAAAGAGACTTTAATT 90974

Query 322362 CATCTTTCTTTGGTCCTCACGCATTTTTGGTTGGCGTCAGCTTCTTCTTCCAATGCCTTC 322421

||||||||||||||||||||||||||||||||||||||||||||||||||||||||||||

Sbjct 90975 CATCTTTCTTTGGTCCTCACGCATTTTTGGTTGGCGTCAGCTTCTTCTTCCAATGCCTTC 91034

Query 322422 TTaaaaaaaaaaGAAATTGGACTTTATTTGCTTGCAGACGACCAGGATGTCAATTTTATC 322481

|| |||||||||||||||||||||||||||||||||||||||||||||||||||||| ||

Sbjct 91035 TT-AAAAAAAAAGAAATTGGACTTTATTTGCTTGCAGACGACCAGGATGTCAATTTTCTC 91093

Query 322482 CTCCCTATGTCACAGATAATTAAGAGAGTCCCAGGAAAGAACCCttttttttATCTGGTC 322541

||||||||||||||||||||||||||||||||||||||||| ||||||||||||||||||

Sbjct 91094 CTCCCTATGTCACAGATAATTAAGAGAGTCCCAGGAAAGAA-CCTTTTTTTTATCTGGTC 91152

Query 322542 TTCGTCATCTGATGACGGGCCAGATGGCGGTTAGAGCGTTAAGCGCAGTCTATCTCGAGA 322601

||||||||||||||||||||||||||||||||||||||||||||||||||||||||||||

Sbjct 91153 TTCGTCATCTGATGACGGGCCAGATGGCGGTTAGAGCGTTAAGCGCAGTCTATCTCGAGA 91212

Query 322602 AGGGAGTTGTGTCCACCTTGGCGGCTTCCTTTTCAGACTTCCTCGTCGCCGACAGATCCC 322661

||||||||||||||||||||||||||||||||||||||||||||||||||||||||||||

Sbjct 91213 AGGGAGTTGTGTCCACCTTGGCGGCTTCCTTTTCAGACTTCCTCGTCGCCGACAGATCCC 91272

Query 322662 TCATTGCTGGCTGGCTACAATAAAAAGACTACCACACCAGCTGCTTTTTTATTGAAGAAG 322721

||||||||||||||||||||||||||||||||||||||||||||||||||||||||||||

Sbjct 91273 TCATTGCTGGCTGGCTACAATAAAAAGACTACCACACCAGCTGCTTTTTTATTGAAGAAG 91332

Query 322722 TCCAAGAGGGGGATATTTCTTCTTCCAAAAATATTATTTCTAATAATAATTAAGTTGAAA 322781

||||||||||||||||||||||||||||||||||||||||||||||||||||||||||||

Sbjct 91333 TCCAAGAGGGGGATATTTCTTCTTCCAAAAATATTATTTCTAATAATAATTAAGTTGAAA 91392

Query 322782 AATGCCTTTCTAACCAAATTTCAAGCTTCGCCCTCGTCTATCTAAGAGAAAAATAGGGCG 322841

||||||||||||||||||||||||||||||||||||||||||||||||||||||||||||

Sbjct 91393 AATGCCTTTCTAACCAAATTTCAAGCTTCGCCCTCGTCTATCTAAGAGAAAAATAGGGCG 91452

Query 322842 CCAGAGAAAGTCTTCACAGTCTCCGATGGTCCAATTCCCCGTGAAAAGAGTGCCGAGTTG 322901

||||||||||||||||||||||||||||||||||||||||||||||||||||||||||||

Sbjct 91453 CCAGAGAAAGTCTTCACAGTCTCCGATGGTCCAATTCCCCGTGAAAAGAGTGCCGAGTTG 91512

Query 322902 GCATGAATTCGAAAAGCTTGTGAGCCCTTTGTTAAAAAGTTATCGACTCCTGTGTCATCT 322961

||||||||||||||||||||||||||||||||||||||||||||||||||||||||||||

Sbjct 91513 GCATGAATTCGAAAAGCTTGTGAGCCCTTTGTTAAAAAGTTATCGACTCCTGTGTCATCT 91572

Query 322962 TCTACCACAAGATTGAGTCGGTACCTCAGGGCGTAGCTTGGTTTAAGTACGAGAAGCCAT 323021

||||||||||||||||||||||||||||||||||||||||||||||||||||||||||||

Sbjct 91573 TCTACCACAAGATTGAGTCGGTACCTCAGGGCGTAGCTTGGTTTAAGTACGAGAAGCCAT 91632

Query 323022 ATGCCCATAACATATCGATAATAAATATTACTTACTTAGATGAAATATAGCAAAAGTACG 323081

||| ||||||||||||||||||||||||||||||||||||||||||||||||||||||||

Sbjct 91633 ATGTCCATAACATATCGATAATAAATATTACTTACTTAGATGAAATATAGCAAAAGTACG 91692

Query 323082 AATAAGTCGGGGTCtttttttATCTTAAAGAAGC-tttttttttGTGCACTCGTATCCTC 323140

|||||||||||||||||||||||||||||||||| |||||||||||||||||||||||||

Sbjct 91693 AATAAGTCGGGGTCTTTTTTTATCTTAAAGAAGCTTTTTTTTTTGTGCACTCGTATCCTC 91752

Query 323141 CGGAGGCATGATGCACGCACGCCACCACGGCAATGAAAGCAGGACTTCTAGTGCCATCCT 323200

||||||||||||||||||||||||||||||||||||||||||||||| ||||||||||||

Sbjct 91753 CGGAGGCATGATGCACGCACGCCACCACGGCAATGAAAGCAGGACTTATAGTGCCATCCT 91812

Query 323201 TCGCTGCGATGCCTATCACCCACCAGCGCTTGACAGCAAAACTTATCTATCTGCGATGGA 323260

|||||||||||||||||||||||||||||||||||| |||||||||||||||||||||||

Sbjct 91813 TCGCTGCGATGCCTATCACCCACCAGCGCTTGACAGTAAAACTTATCTATCTGCGATGGA 91872

Query 323261 AACAAAATAAGAAATCATGTATGTATACAATATATAGTGATAATAATTTAGACATGACTA 323320

||||||||||||||||||||||||||||||||||||||||||||||||||||||||||||

Sbjct 91873 AACAAAATAAGAAATCATGTATGTATACAATATATAGTGATAATAATTTAGACATGACTA 91932

Query 323321 ACCATGTTTCCGGCAGCTTTCATAGTTTCtttttttATTTTCACTGATTAGTTGTGGGTA 323380

|||||||||||||||||||||||||||| |||||||||||||||||||||||||||||||

Sbjct 91933 ACCATGTTTCCGGCAGCTTTCATAGTTTATTTTTTTATTTTCACTGATTAGTTGTGGGTA 91992

Query 323381 CATGGTCTTTTGTGCCTGCATCAGCAATCGGGGCAGAAGCTAAACCAAGCCGCCTATAAC 323440

||||||||||||||||||||||||||||||||||||||||||||||||||||||||||||

Sbjct 91993 CATGGTCTTTTGTGCCTGCATCAGCAATCGGGGCAGAAGCTAAACCAAGCCGCCTATAAC 92052

Query 323441 ATGAAATGAGATTCAGGCCAGTGGAATGTTTACCGAAGGTATAAGAAGAAAACATGTGTA 323500

||||||||||||||||||||||||||||||||||||||||||||||||||||||||||||

Sbjct 92053 ATGAAATGAGATTCAGGCCAGTGGAATGTTTACCGAAGGTATAAGAAGAAAACATGTGTA 92112

Query 323501 GAGATATATATACCTTGTTCTTTAGTCATTAAATTGTAGCATATCACAGTaaaaaaaGCA 323560

||| |||||||||||||||||||||||||||||| ||||||||||||||||||||||

Sbjct 92113 GAG----ATATACCTTGTTCTTTAGTCATTAAATTGTGGCATATCACAGTAAAAAAAGCA 92168

Query 323561 TCGAGTTCCTTAGGAGGATGCTACAGAGAGCCTCGCTCCTAATCCCGTGAAAAAATGAGA 323620

|||||||||||||||||||| |||||||||||||||||||||||||||||||||||||||

Sbjct 92169 TCGAGTTCCTTAGGAGGATGTTACAGAGAGCCTCGCTCCTAATCCCGTGAAAAAATGAGA 92228

Query 323621 CGTTCCTTATCAAACATATTAGCTTTTGAGCATAGGAGATATGTTTTTAAAAATGTTTAC 323680

||||||||||||||||||||||||||||||||||||||||||||||||||||||||||||

Sbjct 92229 CGTTCCTTATCAAACATATTAGCTTTTGAGCATAGGAGATATGTTTTTAAAAATGTTTAC 92288

Query 323681 CGTGCAATTTTTTAACAATCAATCCAGCCAAGATGGCAACAATCGGCCTCGACTGGCTTG 323740

||||||||||||||||||||||||||||||||||||||||||||||||||||||||||||

Sbjct 92289 CGTGCAATTTTTTAACAATCAATCCAGCCAAGATGGCAACAATCGGCCTCGACTGGCTTG 92348

Query 323741 CTTTGCTTCTTGGGCCGAAGCAACGAAGTAAGAAAAGTCCGCCTTTCAAAGACCAAAGGG 323800

||||||||||||||||||||||||||||||||||||||||||||||||||||||||||||

Sbjct 92349 CTTTGCTTCTTGGGCCGAAGCAACGAAGTAAGAAAAGTCCGCCTTTCAAAGACCAAAGGG 92408

Query 323801 AAGACCTAGCCTCTAGTTATTGTCCTAGCCTGAACTTTGGAACAAGCAAGTTTATCAACC 323860

||||||||||||||||||||||||||||||||||||||||||||||||||||||||||||

Sbjct 92409 AAGACCTAGCCTCTAGTTATTGTCCTAGCCTGAACTTTGGAACAAGCAAGTTTATCAACC 92468

Query 323861 TTCTTCTCAACTATCTTACTTTCTAGAAATCTTAAGCTTGCTATCTCCTGGAACTACATC 323920

||||||||||||||||||||||||||||||||||||||||||||||||||||||||||||

Sbjct 92469 TTCTTCTCAACTATCTTACTTTCTAGAAATCTTAAGCTTGCTATCTCCTGGAACTACATC 92528

Query 323921 TTCTCAACTATGTATGTCCTGGTGTCTTCTGAGTCAATAATATATGCTATTCTTCTTACT 323980

||||||||||||||||||||||||||||||||||||||||||||||||||||||||||||

Sbjct 92529 TTCTCAACTATGTATGTCCTGGTGTCTTCTGAGTCAATAATATATGCTATTCTTCTTACT 92588

Query 323981 TTGTGCTGGATAATAGAGTAGCAAAATCTTGCATCTGGAACAATACTTTGCTCTAGCCTC 324040

||||||||||||||||||||||||||||||||||||||||||||||||||||||||||||

Sbjct 92589 TTGTGCTGGATAATAGAGTAGCAAAATCTTGCATCTGGAACAATACTTTGCTCTAGCCTC 92648

Query 324041 ACACACCCAGGCCTAGGAGAGAAGACTTCCATATTGGCAGTAACACTTTCTTCAATACGT 324100

||||||||||||||||||||||||||||||||||||||||||||||||||||||||||||

Sbjct 92649 ACACACCCAGGCCTAGGAGAGAAGACTTCCATATTGGCAGTAACACTTTCTTCAATACGT 92708

Query 324101 ATGGGTCTTCAAATGCTACTGGTAGAAAGGTACTGGAGCCGCGGGTGGTCTTGAAAATGG 324160

||||||||||||||||||||||||||||||||||||||||||||||||||||||||||||

Sbjct 92709 ATGGGTCTTCAAATGCTACTGGTAGAAAGGTACTGGAGCCGCGGGTGGTCTTGAAAATGG 92768

Query 324161 TGCTTTGGTACTGTATAACCTTTCAGGAATCTGCTAACGCTAAATGCGAAGAACTTAGGG 324220

||||||||||||||||||||||||||||||||||||||||||||||||||||||||||||

Sbjct 92769 TGCTTTGGTACTGTATAACCTTTCAGGAATCTGCTAACGCTAAATGCGAAGAACTTAGGG 92828

Query 324221 TGGGGGACTTTATATGGCATGTAGGGGCTTACTAACTGCTTCTATTGCTATTATCTTGAA 324280

||||||||||||||||||||||||||||||||||||||||||||||||||||||||||||

Sbjct 92829 TGGGGGACTTTATATGGCATGTAGGGGCTTACTAACTGCTTCTATTGCTATTATCTTGAA 92888

Query 324281 GTTCCTAGTCTGAACAATCCTAGCTCAGTAACACCTTTCCTAACGCTTTATTTATTCCCG 324340

||||||||||||||||||||||||||||||||||||||||||||||||||||||||||||

Sbjct 92889 GTTCCTAGTCTGAACAATCCTAGCTCAGTAACACCTTTCCTAACGCTTTATTTATTCCCG 92948

Query 324341 CGCAGGATCTAATTGACTCCCGGAACACCGCATCGCTGGGTCTATCGCATCTTATCTTTA 324400

||||||||||||||||||||||||||||||||||||||||||||||||||||||||||||

Sbjct 92949 CGCAGGATCTAATTGACTCCCGGAACACCGCATCGCTGGGTCTATCGCATCTTATCTTTA 93008

Query 324401 CGGCTTCTACGCTACTTCTAAGTCAGTAAATTAGTCATTGTAAGTAAATAAGTCATTACG 324460

||||||||||||||||||||||||||||||||||||||||||||||||||||||||||||

Sbjct 93009 CGGCTTCTACGCTACTTCTAAGTCAGTAAATTAGTCATTGTAAGTAAATAAGTCATTACG 93068

Query 324461 TGTGTGGTAAATCCAATTAGGTAGGAAGAGAAATAAGTAATAAGTATGGAAGAGAATTAT 324520

||||||||||||||||||||||||||||||||||||||||||||||||||||||||||||

Sbjct 93069 TGTGTGGTAAATCCAATTAGGTAGGAAGAGAAATAAGTAATAAGTATGGAAGAGAATTAT 93128

Query 324521 CTTGTACCGAGGCCGAACTATCAGAGTTATAGGAATAATCTAGCCTTGACTATACCTTTT 324580

||||||||||||||||||||||||||||||||||||||||||||||||||||||||||||

Sbjct 93129 CTTGTACCGAGGCCGAACTATCAGAGTTATAGGAATAATCTAGCCTTGACTATACCTTTT 93188

Query 324581 GTACTTGTTTACCTGAACCAAGCACCTTACTTAGCCTTGAAGAATTTGCACTAGCTCTTA 324640

||||||||||||||||||||||||||||||||||||||||||||||||||||||||||||

Sbjct 93189 GTACTTGTTTACCTGAACCAAGCACCTTACTTAGCCTTGAAGAATTTGCACTAGCTCTTA 93248

Query 324641 AGCATGCATGCTTCTCGGTTCGTGGACCAGACCTATCCTATCTCCCTCAGTGACCCTCAT 324700

||||||||||||||||||||||||||||||||||||||||||||||||||||||||||||

Sbjct 93249 AGCATGCATGCTTCTCGGTTCGTGGACCAGACCTATCCTATCTCCCTCAGTGACCCTCAT 93308

Query 324701 GCCTCATTTTGATAAGAGGTAGCCCGCATCAGTTAATTTAGAAAAGGCGCGAAGCGGGTA 324760

||||||||||||||||||||||||||||||||||||||||||||||||||||||||||||

Sbjct 93309 GCCTCATTTTGATAAGAGGTAGCCCGCATCAGTTAATTTAGAAAAGGCGCGAAGCGGGTA 93368

Query 324761 AGGAGGCCCATACCTGAAGTGGAGGCAGTGGTCAAAGGGAGTTGCTGATTGGACCCTACT 324820

||||||||||||||||||||||||||||||||||||||||||||||||||||||||||||

Sbjct 93369 AGGAGGCCCATACCTGAAGTGGAGGCAGTGGTCAAAGGGAGTTGCTGATTGGACCCTACT 93428

Query 324821 TCAGAGTCAGCTGATACCAGGACGGGACAATTCTCTTCATCTCCTCGTTCTTCATCAGCT 324880

||||||||||||||||||||||||||||||||||||||||||||||||||||||||||||

Sbjct 93429 TCAGAGTCAGCTGATACCAGGACGGGACAATTCTCTTCATCTCCTCGTTCTTCATCAGCT 93488

Query 324881 TGGAAAGCATAAATTACTGGGTCTGTCAGCTCCTCTTCATGAATCCATGGAGTGCCACCA 324940

||||||||||||||||||||||||||||||||||||||||||||||||||||||||||||

Sbjct 93489 TGGAAAGCATAAATTACTGGGTCTGTCAGCTCCTCTTCATGAATCCATGGAGTGCCACCA 93548

Query 324941 TCACCATTTTAGCCTCCCTAGAGCCTGTTGCGAAGAGCTTGCAAAGGTACCAAGGTTGAA 325000

||||||||||||||||||||||||||||||||||||||||||||||||||||||||||||

Sbjct 93549 TCACCATTTTAGCCTCCCTAGAGCCTGTTGCGAAGAGCTTGCAAAGGTACCAAGGTTGAA 93608

Query 325001 TTGAAACGTTCTTGACCTAAAGATAATGCACGTCATAAGTTGTAGGTTAAAATAAGAGGC 325060

||||||||||||||||||||||||||||||||||||||||||||||| ||||||||||||

Sbjct 93609 TTGAAACGTTCTTGACCTAAAGATAATGCACGTCATAAGTTGTAGGTGAAAATAAGAGGC 93668

Query 325061 AAAGTTTGCATTTGGCAAGTATCCCTCAATAGCAGCAGTTTACCAGTGAAGCACCGTTTG 325120

||||||||||||||||||||||||||||||||||||||||||||||||||||||||||||

Sbjct 93669 AAAGTTTGCATTTGGCAAGTATCCCTCAATAGCAGCAGTTTACCAGTGAAGCACCGTTTG 93728

Query 325121 ACCCAAATACCATAAATTGCTTGAGCTCCACCTCTGATTGATAATCCATTCGCTTACAAG 325180

||||||||||||||||||||||||||||||||||||||||||||||||||||||||||||

Sbjct 93729 ACCCAAATACCATAAATTGCTTGAGCTCCACCTCTGATTGATAATCCATTCGCTTACAAG 93788

Query 325181 TCAAAACATTTTCTTATCAATGTACTTTGAATTCTTATTGTCTCGTGATTCTCACATAAG 325240

||||||||||||||||||||||||||||||||||||||||||||||||||||||||||||

Sbjct 93789 TCAAAACATTTTCTTATCAATGTACTTTGAATTCTTATTGTCTCGTGATTCTCACATAAG 93848

Query 325241 GTATAGTAATTATTAGAAATCCAGTCAAAAAGACAGAATAGACATCAAATAGAAAAAAGA 325300

||||||||||||||||||||||||||||||||||||||||||||||||||||||||||||

Sbjct 93849 GTATAGTAATTATTAGAAATCCAGTCAAAAAGACAGAATAGACATCAAATAGAAAAAAGA 93908

Query 325301 CTCGAAAAGAATATTATTATTTGTTTGCTAATATAGTTTATCTTTTTCTTTTCAAAATAT 325360

||||||||||||||||||||||||||||||||||||||||||||||||||||||||||||

Sbjct 93909 CTCGAAAAGAATATTATTATTTGTTTGCTAATATAGTTTATCTTTTTCTTTTCAAAATAT 93968

Query 325361 TAACGCAACTCCCCGATTCTTTCCCATCTATCTATATCCATTTTAATTAGATATAAAAAT 325420

||||||||||||||||||||||||| ||||||||||||||||||||||||||| ||||||

Sbjct 93969 TAACGCAACTCCCCGATTCTTTCCCCTCTATCTATATCCATTTTAATTAGATAGAAAAAT 94028

Query 325421 AGAGATAAGATTTGGttttttcatttttATTAAAAGGGAAGAACTCGTGGAACTTCAATA 325480

||||||||||||||||||||||||||||||||||||||||||||||||||||||||||||

Sbjct 94029 AGAGATAAGATTTGGTTTTTTCATTTTTATTAAAAGGGAAGAACTCGTGGAACTTCAATA 94088

Query 325481 ATTTACTAAGAAGAAATACCTTTTCAAAGTTTACGGGGTACAATTGAATCCAAAGAGCCC 325540

||||||||||||||||||||||||||||||||||||||||||||||||||||||||||||

Sbjct 94089 ATTTACTAAGAAGAAATACCTTTTCAAAGTTTACGGGGTACAATTGAATCCAAAGAGCCC 94148

Query 325541 TTTTCAAATAAGTATTCAGCTTCTTGTACACCCTCGGGTACCTCTATCTTCATTACTTCT 325600

||||||||||||||||||||||||||||||||||||||||||||||||||||||||||||

Sbjct 94149 TTTTCAAATAAGTATTCAGCTTCTTGTACACCCTCGGGTACCTCTATCTTCATTACTTCT 94208

Query 325601 TCAAGAACTCTTTGACCCATGTATGCATCTGCTTCGCCAAGAATCCTATTTCCAAGCATT 325660

||||||||||||||||||||||||||||||||||||||||||||||||||||||||||||

Sbjct 94209 TCAAGAACTCTTTGACCCATGTATGCATCTGCTTCGCCAAGAATCCTATTTCCAAGCATT 94268

Query 325661 CCAGGCTTTGACACCACCTTTTCTAGGTGACTAAAAAGAAGTTTTTAATTGATAAATAAA 325720

||||||||||||||||||||||||||||||||||||||||||||||||||||||||||||

Sbjct 94269 CCAGGCTTTGACACCACCTTTTCTAGGTGACTAAAAAGAAGTTTTTAATTGATAAATAAA 94328

Query 325721 ATAAAAGCAAAGAGATTTGAGCCATATGCATCAAGCTGAAACTTCCTTCTTGCATACGAG 325780

||||||||||||||||||||||||||||||||||||||||||||||||||||||||||||

Sbjct 94329 ATAAAAGCAAAGAGATTTGAGCCATATGCATCAAGCTGAAACTTCCTTCTTGCATACGAG 94388

Query 325781 CAGAAGCACAGGAAATTCTTAGAGGTAAGGATTGATTACTAGCATATTCAATTAGATGGG 325840

|||||||||| |||||||||||||||||||||||||||||||||||||||||||||||||

Sbjct 94389 CAGAAGCACAAGAAATTCTTAGAGGTAAGGATTGATTACTAGCATATTCAATTAGATGGG 94448

Query 325841 TTCtttttttATATATTGATTGAGTCTGGCTAGCTAGTTCCGTCTATCGGTCATGGGAAA 325900

||||||||||||||||||||||||||||||||||||||||||||||||||||||||||||

Sbjct 94449 TTCTTTTTTTATATATTGATTGAGTCTGGCTAGCTAGTTCCGTCTATCGGTCATGGGAAA 94508

Query 325901 GCCAAAACTTTGAGAGAATAAGGTCGAGACTCTTTCTTATTGGGAAGACAGCCCCATAAT 325960

||||||||||||||||||||||||||||||||||||||||||||||||||||||||||||

Sbjct 94509 GCCAAAACTTTGAGAGAATAAGGTCGAGACTCTTTCTTATTGGGAAGACAGCCCCATAAT 94568

Query 325961 TCCAAAGATTTCATGCAATGTTCTTAGGTTTTTCCATAGAATCTAGAATATTTTCTATAT 326020

|||||||||||||||||||||||||||| |||||||||||||||||||||||||||||||

Sbjct 94569 TCCAAAGATTTCATGCAATGTTCTTAGGGTTTTCCATAGAATCTAGAATATTTTCTATAT 94628

Query 326021 AAGCTTTTTTCTTTACAAAATTTGAGTTA----TCTATATGATGCTAGGTTCATTTAGTT 326076

||||||||||||||||||||||||||||| |||||||||||||||||||||||||||

Sbjct 94629 AAGCTTTTTTCTTTACAAAATTTGAGTTAGACTTCTATATGATGCTAGGTTCATTTAGTT 94688

Query 326077 TGTCGATGTTCCTGATTATCACATTAATATTGTGATCACACTAATAACGTTTGTTTTCAT 326136

||||||||||||||||||||||||||||||||||||||||||||||||||||||||||||

Sbjct 94689 TGTCGATGTTCCTGATTATCACATTAATATTGTGATCACACTAATAACGTTTGTTTTCAT 94748

Query 326137 CGAAGTTATATTTAAGTATAATTTTTTCTAGACAAGGACTAACTATATTATTTGATCATC 326196

||||||||||||||||||||||||||||||||||||||||||||||||||||||||||||

Sbjct 94749 CGAAGTTATATTTAAGTATAATTTTTTCTAGACAAGGACTAACTATATTATTTGATCATC 94808

Query 326197 ACGTTTTTACCTTACATAGATTGAAACATGAATAGGTCGACTTGCAATTAATTATTCTTT 326256

||||||||||||||||||||||||||||||||||||||||||||||||||||||||||||

Sbjct 94809 ACGTTTTTACCTTACATAGATTGAAACATGAATAGGTCGACTTGCAATTAATTATTCTTT 94868

Query 326257 CTGAGAGAGGAAACGAAATTCTATTAAGCAAAGCAGGCCAACAGGTTTCTTGCttttttt 326316

||||||||||||||||||||||||||||||||||||||||||||||||||||||||||||

Sbjct 94869 CTGAGAGAGGAAACGAAATTCTATTAAGCAAAGCAGGCCAACAGGTTTCTTGCTTTTTTT 94928

Query 326317 ATGATTCCATCCACTTCTAATAGCAATCAAAGGTATAAGAGAAAAGGTTCTCCTTTTAGT 326376

|||||||||||||||| |||||||||||||||||||||||||||||||||||||||||||

Sbjct 94929 ATGATTCCATCCACTTATAATAGCAATCAAAGGTATAAGAGAAAAGGTTCTCCTTTTAGT 94988

Query 326377 TGCAATAGTCTCTGCAACCTCACCAGCGCTGAGATCGCCTGCAGTAGCGCATTTGAAAAT 326436

||||||||||||||||||||||||||||||||||||||||||||||||||||||||||||

Sbjct 94989 TGCAATAGTCTCTGCAACCTCACCAGCGCTGAGATCGCCTGCAGTAGCGCATTTGAAAAT 95048

Query 326437 CCAACCCTGCGCAGAGATAGATGGGACAGAGGCGGTCCTTGGCGAGAGAAAGCAATTAAC 326496

||||||||||||||||||||||||||||||||||||||||||||||||||||||||||||

Sbjct 95049 CCAACCCTGCGCAGAGATAGATGGGACAGAGGCGGTCCTTGGCGAGAGAAAGCAATTAAC 95108

Query 326497 ACCATGGACATTTATCAAGGATATTTTTCTACATTCTAAAGTaaaaaaaCAATTGGTGTT 326556

||||||||||||||||||||||||||||||||||||||||||||||||||||||||||||

Sbjct 95109 ACCATGGACATTTATCAAGGATATTTTTCTACATTCTAAAGTAAAAAAACAATTGGTGTT 95168

Query 326557 GGTTAATGAGAATTTCCTAACAAAACCCTCCCAAAGCAAGCAATTGCAACAACAAATTTA 326616

||||||||||||||||||||||||||||||||||||||||||||||||||||||||||||

Sbjct 95169 GGTTAATGAGAATTTCCTAACAAAACCCTCCCAAAGCAAGCAATTGCAACAACAAATTTA 95228

Query 326617 AGCATATATCTAGCATTCATTTCCAATTCATGATAAATCTATAGCGGGCAAAGACAAGAT 326676

||||||||||||||||||||||||||||||||||||||||||||||||||||||||||||

Sbjct 95229 AGCATATATCTAGCATTCATTTCCAATTCATGATAAATCTATAGCGGGCAAAGACAAGAT 95288

Query 326677 AGAGAGAGCTATACAGTAGCTTAGCATATTAAGAAACACAAAGAAAGCAGGAAAATTGGT 326736

||||||||||||||||||||||||||||||||||||||||||||||||||||||||||||

Sbjct 95289 AGAGAGAGCTATACAGTAGCTTAGCATATTAAGAAACACAAAGAAAGCAGGAAAATTGGT 95348

Query 326737 GAGACATCAGGTCATCGAAAGTTTTTCATATTCTTTCAAATCTCTGGAATTAATGTATAA 326796

||||||||||||||||||||||||||||||||||||||||||||||||||||||||||||

Sbjct 95349 GAGACATCAGGTCATCGAAAGTTTTTCATATTCTTTCAAATCTCTGGAATTAATGTATAA 95408

Query 326797 ACTGACTACTTTCTTGAATTATGTGTTACGTGAAAAATAGAAAGATGCCATCTAATTTTC 326856

||||||||||||||||||||||||||||||||||||||||||||||||||||||||||||

Sbjct 95409 ACTGACTACTTTCTTGAATTATGTGTTACGTGAAAAATAGAAAGATGCCATCTAATTTTC 95468

Query 326857 ACGAGGATGCATGATCTGTGTCAATAAGGTAGAGAGATTATAACATAATTTTTTAATCTA 326916

||||||||||||||||||||||||||||||||||||||||||||||||||||||||||||

Sbjct 95469 ACGAGGATGCATGATCTGTGTCAATAAGGTAGAGAGATTATAACATAATTTTTTAATCTA 95528

Query 326917 AGTCAACTTTAGGAAATGCAAAGCTTCGCCCTTGATGTCAGAATCATCACAACACAAAAT 326976

||||||||||||||||||||||||||||||||||||||||||||||||||||||||||||

Sbjct 95529 AGTCAACTTTAGGAAATGCAAAGCTTCGCCCTTGATGTCAGAATCATCACAACACAAAAT 95588

Query 326977 GAAGAAAAATCTAGCACACAATATTTTAGAAATAcccccccTCAAATTATGGATCACTTT 327036

||||||||||||||||||||||||||||||||||||||||||||||||||||||||||||

Sbjct 95589 GAAGAAAAATCTAGCACACAATATTTTAGAAATACCCCCCCTCAAATTATGGATCACTTT 95648

Query 327037 CCTTCCTCATCAGATTGTGAATCAGTAATTTGCCTACCATGTTGAGTATCTCCAAGGCCA 327096

||||||||||||||||||||||||||||||||||||||||||||||||||||||||||||

Sbjct 95649 CCTTCCTCATCAGATTGTGAATCAGTAATTTGCCTACCATGTTGAGTATCTCCAAGGCCA 95708

Query 327097 TCATCAGAACTACCATCGTCACGGGGTTGAATTTTGATATGTGACCTGGAATCGGAGAAA 327156

||||||||||||||||||||||||||||||||||||||||||||||||||||||||||||

Sbjct 95709 TCATCAGAACTACCATCGTCACGGGGTTGAATTTTGATATGTGACCTGGAATCGGAGAAA 95768

Query 327157 TCCGCCGGGTCTACTGGGTCAACCGGATATGCGCCTTATTCTTGCCAGTTAGATAGAGGG 327216

||||||||||||||||||||||||||||||||||||||||||||||||||||||||||||

Sbjct 95769 TCCGCCGGGTCTACTGGGTCAACCGGATATGCGCCTTATTCTTGCCAGTTAGATAGAGGG 95828

Query 327217 TTGGTAAGCTCTCATAAGGGATCTCTCTAAGTATATTGTAGCAAATTTCATCACTTAAGG 327276

||||||||||||||||||||||||||||||||||||||||||||||||||||||||||||

Sbjct 95829 TTGGTAAGCTCTCATAAGGGATCTCTCTAAGTATATTGTAGCAAATTTCATCACTTAAGG 95888

Query 327277 GAATCATCGCATCGATCCATTATAGACTGTATTATTACTCTTGTTACCATTAGGATAAAT 327336

||||||||||||||||||||||||||||||||||||||||||||||||||||||||||||

Sbjct 95889 GAATCATCGCATCGATCCATTATAGACTGTATTATTACTCTTGTTACCATTAGGATAAAT 95948

Query 327337 CTGACCCCTTCCTCTGTTTCCACATACATATATAGGATATTTTCAGAAGTGAATGTCTTT 327396

||||||||||||||||||||||||||||||||||||||||||||||||||||||||||||

Sbjct 95949 CTGACCCCTTCCTCTGTTTCCACATACATATATAGGATATTTTCAGAAGTGAATGTCTTT 96008

Query 327397 CTTCGTAGATAATAAGTCTGTCTTCTAATAGTAGAACGCTGAGTCTTCCTTCTGCATCTT 327456

||||||||||||||||||||||||||||||||||||||||||||||||||||||||||||

Sbjct 96009 CTTCGTAGATAATAAGTCTGTCTTCTAATAGTAGAACGCTGAGTCTTCCTTCTGCATCTT 96068

Query 327457 TGAACttttttttCTCTTGAGACAGAGCCTCACTTGCTTCTTATACAAGGCTCTCCTTCC 327516

||||||||||||||||||||||||||||||||||||||||||||||||||||||||||||

Sbjct 96069 TGAACTTTTTTTTCTCTTGAGACAGAGCCTCACTTGCTTCTTATACAAGGCTCTCCTTCC 96128

Query 327517 TCAAAGTCTTCTTTATTTTAGAGGAAAAAGCCAAATTAGGAGAATGTCATGAATGCTCCG 327576

|||||||||||||||||| |||||||||||||||||||||||||||||||||||||||||

Sbjct 96129 TCAAAGTCTTCTTTATTTGAGAGGAAAAAGCCAAATTAGGAGAATGTCATGAATGCTCCG 96188

Query 327577 GTACAGGAACAGCTGAAATGAAGCTTAAAAAGGATTTAGTTATCCAGTCTCAAATCCAGC 327636

||||||||||||||||||||||||||||||||||||||||||||||||||||||||||||

Sbjct 96189 GTACAGGAACAGCTGAAATGAAGCTTAAAAAGGATTTAGTTATCCAGTCTCAAATCCAGC 96248

Query 327637 AAGCAAGAATAGGATGCAGCAAGCAAGAAAACGGACAGCATTTCTCGTACTCCAGGCATA 327696

||||||||||||||||||||||||||||||||||||||||||||||||||||||||||||

Sbjct 96249 AAGCAAGAATAGGATGCAGCAAGCAAGAAAACGGACAGCATTTCTCGTACTCCAGGCATA 96308

Query 327697 GGCACTCCAGCAAAGGCATCGAAGAGTATTGGTCAGATAATGAATAGCCGGTTCAAACCA 327756

||||||||||||||||||||||||||||||||||||||||||||||||||||||||||||

Sbjct 96309 GGCACTCCAGCAAAGGCATCGAAGAGTATTGGTCAGATAATGAATAGCCGGTTCAAACCA 96368

Query 327757 TACATTTCTTTCTCATCTTTGATGATCACTCGCTCTTCACATTCAGAACTCTTATTCCCC 327816

||||||||||||||||||||||||||||||||||||||||||||||||||||||||||||

Sbjct 96369 TACATTTCTTTCTCATCTTTGATGATCACTCGCTCTTCACATTCAGAACTCTTATTCCCC 96428

Query 327817 TCCGACCGCTTATTAACTTTGACTCGTGCATCCGGGAGTCTCTCTCTCTTTCTATGAGGA 327876

||||||||||||||||||||||||||||||||||||||||||||||||||||||||||||

Sbjct 96429 TCCGACCGCTTATTAACTTTGACTCGTGCATCCGGGAGTCTCTCTCTCTTTCTATGAGGA 96488

Query 327877 GTTAATCCTAGGTAGAGCACCCTTTTTTGAAGTGCATCACTTTCCCAGCTTTGCATTCTG 327936

||||||||||||||||||||||||||||||||||||||||||||||||||||||||||||

Sbjct 96489 GTTAATCCTAGGTAGAGCACCCTTTTTTGAAGTGCATCACTTTCCCAGCTTTGCATTCTG 96548

Query 327937 AAAAGACTAACTCTAGCTTTTGACTAAGTACCCAACTTTCTACTTGTCAAACTGTCGATG 327996

||||||||||||||||||||||||||||||||| ||||||||||||||||||||||||||

Sbjct 96549 AAAAGACTAACTCTAGCTTTTGACTAAGTACCCTACTTTCTACTTGTCAAACTGTCGATG 96608

Query 327997 GCTATGGATCTCCGTCCTGCTTCAGTTCGAAGTGCCATCCTGACTTGAGTATGGTTCTTG 328056

||||||||||||||||||||||||||||||||||||||||||||||||||||||||||||

Sbjct 96609 GCTATGGATCTCCGTCCTGCTTCAGTTCGAAGTGCCATCCTGACTTGAGTATGGTTCTTG 96668

Query 328057 CATTCTTTCGGTAGAATTTCCATTTTGCTTGTGAGCCATATCCTTCTTTCCGGGAAACAA 328116

||||||||||||| ||||||||||||||||||||||||||||||||||||||||||||||

Sbjct 96669 CATTCTTTCGGTATAATTTCCATTTTGCTTGTGAGCCATATCCTTCTTTCCGGGAAACAA 96728

Query 328117 GTGACTGCCCGGCTGGTCTCTTCACTCGATCTAAGACACGCCTTTCCAAGAGGGTAGTTT 328176

||||||||||||||||||||||||||||||||||||||||||||||||||||||||||||

Sbjct 96729 GTGACTGCCCGGCTGGTCTCTTCACTCGATCTAAGACACGCCTTTCCAAGAGGGTAGTTT 96788

Query 328177 GTGATCATTAAAAAGCATAAAGAGAAGAAAGATGAAGAGGTATGGAATAGGCCAATGGTT 328236

||||||||||||||||||||||||||||||||||||||||||||||||||||||||||||

Sbjct 96789 GTGATCATTAAAAAGCATAAAGAGAAGAAAGATGAAGAGGTATGGAATAGGCCAATGGTT 96848

Query 328237 CGAAACCTCGCTATAATTGTCAAAAATCAACTCGATACCGAGGAAAAAGCTATCTCAAAA 328296

||||||||||||||||||||||||||||||||||||||||||||||||||||||||||||

Sbjct 96849 CGAAACCTCGCTATAATTGTCAAAAATCAACTCGATACCGAGGAAAAAGCTATCTCAAAA 96908

Query 328297 TGCATCTTTCACTACTGTCACCATTCGCTAGCTTAAGGAAGTGAGCTTCATTCACCGATT 328356

||||||||||||||||||||||||||||||||||||||||||||||||||||||||||||

Sbjct 96909 TGCATCTTTCACTACTGTCACCATTCGCTAGCTTAAGGAAGTGAGCTTCATTCACCGATT 96968

Query 328357 GCTAGACCTATTAGCTAGCCAAGCCCAACAGAAAGAAAGCTTTGCTTGCCAGTTTCCAAT 328416

||||||||||||||||||||||||||||||||||||||||||||||||||||||||||||

Sbjct 96969 GCTAGACCTATTAGCTAGCCAAGCCCAACAGAAAGAAAGCTTTGCTTGCCAGTTTCCAAT 97028

Query 328417 TGTGAGATTTCTTAGCTTTTAATAGGCAGGTGAGAAGTGAACATTTTCTAAACTTAACAC 328476

||||||||||||||||||||||||||||||||||||||||||||||||||||||||||||

Sbjct 97029 TGTGAGATTTCTTAGCTTTTAATAGGCAGGTGAGAAGTGAACATTTTCTAAACTTAACAC 97088

Query 328477 AACCACCAGTCGGTTGATTCTTTTTGCTTTTTATGGGTACCACAGTGGGTTTTCGCGCAC 328536

||||||||||||||||||||||||||||||||||||||||||||||||||||||||||||

Sbjct 97089 AACCACCAGTCGGTTGATTCTTTTTGCTTTTTATGGGTACCACAGTGGGTTTTCGCGCAC 97148

Query 328537 CTACTTCTTAATGGATGCTCATTGGAAAGAATGGATGAACGAAATTTGGGAACAGAAACT 328596

||||||||||||||||||||||||||||||||||||||||||||||||||||||||||||

Sbjct 97149 CTACTTCTTAATGGATGCTCATTGGAAAGAATGGATGAACGAAATTTGGGAACAGAAACT 97208

Query 328597 CATAAATAGTCTTTCTAGCTATAATGGGAAGCAAGTAAATAGGGGAATAGGAAAAACTTA 328656

|||||||||||||||||||||||| |||||||||||||||||||||||||||||||||||

Sbjct 97209 CATAAATAGTCTTTCTAGCTATAAAGGGAAGCAAGTAAATAGGGGAATAGGAAAAACTTA 97268

Query 328657 GCACCTCTAAATCAGTCAAGCTTCTATAGCTGTAGGAACTTTCCTCTGGTGACTTTGACC 328716

||||||||||||||||||||||||||||||||||||||||||||||||||||||||||||

Sbjct 97269 GCACCTCTAAATCAGTCAAGCTTCTATAGCTGTAGGAACTTTCCTCTGGTGACTTTGACC 97328

Query 328717 TTGAGCTTAAGTGAACAATGTCAAGAAAAGAGGAGAATTTTCAGTGAACAAGCAAGCGAA 328776

||||||||||||||||||||||||||||||||||||||||||||||||||||||||||||

Sbjct 97329 TTGAGCTTAAGTGAACAATGTCAAGAAAAGAGGAGAATTTTCAGTGAACAAGCAAGCGAA 97388

Query 328777 GAGATTGAGTTGTTAGCGAATAAATACTACTTCCAGTACTTCCTACTCACAAGTCAGGAA 328836

||||||||||||||||||||||||||||||||||||||||||||||||||||||||||||

Sbjct 97389 GAGATTGAGTTGTTAGCGAATAAATACTACTTCCAGTACTTCCTACTCACAAGTCAGGAA 97448

Query 328837 CGAAGTACCTAGCCCTTCCTACACAACTTCAGTGTTGAACAAAACCACGACTCACTACAC 328896

||||||||||||||||||||||||||||||||||||||||||||||||||||||||||||

Sbjct 97449 CGAAGTACCTAGCCCTTCCTACACAACTTCAGTGTTGAACAAAACCACGACTCACTACAC 97508

Query 328897 TGAATATTCAGCATGAACAGTACGAAATAGGTATAAGCAATGTATAGCGCCATGAAGGGC 328956

||||||||||||||||||||||||||||||||||||||||||||||||||||||||||||

Sbjct 97509 TGAATATTCAGCATGAACAGTACGAAATAGGTATAAGCAATGTATAGCGCCATGAAGGGC 97568

Query 328957 AGACAATGAAGAATGAAAGAATGAATGAAAAGCACCCTCACAACTATTAGAATCTAGTGA 329016

||||||||||||||||||||||||||||||||||||||||||||||||||||||||||||

Sbjct 97569 AGACAATGAAGAATGAAAGAATGAATGAAAAGCACCCTCACAACTATTAGAATCTAGTGA 97628

Query 329017 TCATATGAATCTTTATGGTGAAGAAGTAGTTGTCAAAAGTCAAAGAGCTAGGCATTGCAA 329076

||||||||||||||||||||||||||||||||||||||||||||||||||||||||||||

Sbjct 97629 TCATATGAATCTTTATGGTGAAGAAGTAGTTGTCAAAAGTCAAAGAGCTAGGCATTGCAA 97688

Query 329077 AACTAGGCTTTATGTAGCCAACATATCAAAGTCTAAAAAGAGCTAGCTAGGCATAAGGTA 329136

||||||||||||||||||||||||||||||||||||||||||||||||||||||||||||

Sbjct 97689 AACTAGGCTTTATGTAGCCAACATATCAAAGTCTAAAAAGAGCTAGCTAGGCATAAGGTA 97748

Query 329137 TAGGGGTAGGATAGCCGCCAAGTTCAATGACATCGAAGTATAGGGCTAGGGAGGGGTTGT 329196

||||||||||||||||||||||||||||||||||||||||||||||||||||||||||||

Sbjct 97749 TAGGGGTAGGATAGCCGCCAAGTTCAATGACATCGAAGTATAGGGCTAGGGAGGGGTTGT 97808

Query 329197 GTGATAGGCAATGGAATAGGCATTGTTCAGGTATATGGACTTATGCATTCTTTTATTTTC 329256

||||||||||||||||||||||||||||||||||||||||||||||||||||||||||||

Sbjct 97809 GTGATAGGCAATGGAATAGGCATTGTTCAGGTATATGGACTTATGCATTCTTTTATTTTC 97868

Query 329257 ATGAAAGATCTTATTCACCAATAGGAACTTTGCGAGGTAGCTTGGTCTTGGGTATGCCTA 329316

||||||||||||||||||||||||||||||||||||||||||||||||||||||||||||

Sbjct 97869 ATGAAAGATCTTATTCACCAATAGGAACTTTGCGAGGTAGCTTGGTCTTGGGTATGCCTA 97928

Query 329317 ACGTTTAGAGCTAGCAAGATGTAAAAGATCATAAGGTCCTAAATAGACCTTTGTGAATAG 329376

||||||||||||||||||||||||||||||||||||||||||||||||||||||||||||

Sbjct 97929 ACGTTTAGAGCTAGCAAGATGTAAAAGATCATAAGGTCCTAAATAGACCTTTGTGAATAG 97988

Query 329377 GCCAGAGATGGGTAAGCTGCTGCTGGGAATTCCTACCATTAGTTCAGTCTAGTTTTCTTG 329436

||||||||||||||||||||||||||||||||||||||||||||||||||||||||||||

Sbjct 97989 GCCAGAGATGGGTAAGCTGCTGCTGGGAATTCCTACCATTAGTTCAGTCTAGTTTTCTTG 98048

Query 329437 GCGAGAGATGGTTTGAAGCTGTAAATGCCTTTCCAGAGTCATCGTACTCTTTGATATCAA 329496

||||||||||||||||||||||||||||||||||||||||||||||||||||||||||||

Sbjct 98049 GCGAGAGATGGTTTGAAGCTGTAAATGCCTTTCCAGAGTCATCGTACTCTTTGATATCAA 98108

Query 329497 AGTAATAGGCATAAGAGTACTGCTACTCGGGCAGCTGATCGATTCCAAAATAGCCTTAGC 329556

||||||||||||||||||||||||||||||||||||||||||||||||||||||||||||

Sbjct 98109 AGTAATAGGCATAAGAGTACTGCTACTCGGGCAGCTGATCGATTCCAAAATAGCCTTAGC 98168

Query 329557 TAGGAGCGGGAGGATGAAGTTGCTGAAACTTGCAGTAATGCTCCAAGAGACTGGATAAAC 329616

||||||||||||||||||||||||||||||||||||||||||||||||||||||||||||

Sbjct 98169 TAGGAGCGGGAGGATGAAGTTGCTGAAACTTGCAGTAATGCTCCAAGAGACTGGATAAAC 98228

Query 329617 TATGAGCCAAGGTGGATTAGTCGCCAAGCATGGAGAGAGTACTGTCTGGTGTGGGCTCGA 329676

||||||||||||||||||||||||||||||||||||||||||||||||||||||||||||

Sbjct 98229 TATGAGCCAAGGTGGATTAGTCGCCAAGCATGGAGAGAGTACTGTCTGGTGTGGGCTCGA 98288

Query 329677 AGGGCAATCCTACAATGAATTGGTCTCATTGGTTTACAACTTGTTGTTGAGTCGGGCGGG 329736

||||||||||||||||||||||||||||||||||||||||||||||||||||||||||||

Sbjct 98289 AGGGCAATCCTACAATGAATTGGTCTCATTGGTTTACAACTTGTTGTTGAGTCGGGCGGG 98348

Query 329737 CAGGAGGAAGAAATCTCATATTATCATAAATAGAGTCGAGtttttcgtgctttcaaactt 329796

||||||||||||||||||||||||||||||||||||||||||||||||||||||||||||

Sbjct 98349 CAGGAGGAAGAAATCTCATATTATCATAAATAGAGTCGAGTTTTTCGTGCTTTCAAACTT 98408

Query 329797 ctttcttttctttccttttccaagaaataacactcgcttctttctcttACTGGGAATGAA 329856

||||||||||||||||||||||||||||||||||||||||||||||||||||||||||||

Sbjct 98409 CTTTCTTTTCTTTCCTTTTCCAAGAAATAACACTCGCTTCTTTCTCTTACTGGGAATGAA 98468

Query 329857 CCAAGCTCAGACTCTGCTGATACGTGATTACAACAATTGCATAGAAACCCCTTCTGCTTA 329916

||||||||||||||||||||||||||||||||||||||||||||||||||||||||||||

Sbjct 98469 CCAAGCTCAGACTCTGCTGATACGTGATTACAACAATTGCATAGAAACCCCTTCTGCTTA 98528

Query 329917 TGTGAGTTTGACCCGCCTTTGACTCTGCTTGCTTCTGACTCCCTGCCATTTTACGACCTT 329976

||||||||||||||||||||||||||||||||||||||||||||||||||||||||||||

Sbjct 98529 TGTGAGTTTGACCCGCCTTTGACTCTGCTTGCTTCTGACTCCCTGCCATTTTACGACCTT 98588

Query 329977 ACtttttctttttttCAGAATATCTTATTATATAGTGTGTGTAGAAAGCTCTTTTGCTTC 330036

||||||||||||||||||||||||||||||||||||||||||||||||||||||||||||

Sbjct 98589 ACTTTTTCTTTTTTTCAGAATATCTTATTATATAGTGTGTGTAGAAAGCTCTTTTGCTTC 98648

Query 330037 TAGGATAAAAAGCTTTTCTATGAGGATCGTGAGTTTGCAATAAAAGGAGGAAAGAAAAGG 330096

|||||||||||||||||||||||||||||||||||||||| |||||||||||||||||||

Sbjct 98649 TAGGATAAAAAGCTTTTCTATGAGGATCGTGAGTTTGCAAGAAAAGGAGGAAAGAAAAGG 98708

Query 330097 AATCAACTCAGAAATCAGTATGTGTGTAGCAAATCAAAAGCGTGaaaaaaaaaGGGGTTT 330156

||||||||||||||||||||||||||||||||||||||||||||||||||||||||||||

Sbjct 98709 AATCAACTCAGAAATCAGTATGTGTGTAGCAAATCAAAAGCGTGAAAAAAAAAGGGGTTT 98768

Query 330157 TGTTATGTCGGaaaaaaaGATAAAAGAAATGCTTCTTCGGCAGCTTTCCTTCTTTCCTGG 330216

||||||||||||||||||||||||||||||||||||||||||||||||||||||||||||

Sbjct 98769 TGTTATGTCGGAAAAAAAGATAAAAGAAATGCTTCTTCGGCAGCTTTCCTTCTTTCCTGG 98828

Query 330217 CTTAGAGAAGAACATAGATTGTTTGCTCAAGGTGCTCACGCCTGGCGATATATCTGAGAT 330276

||||||||||||||||||||||||||||||||||||||||||||||||||||||||||||

Sbjct 98829 CTTAGAGAAGAACATAGATTGTTTGCTCAAGGTGCTCACGCCTGGCGATATATCTGAGAT 98888

Query 330277 AGCTTGCACTTTTCCTAGAGAAGAGTGGGTTTTCGAAGTATTTTCTCCACGTGAGCTGAA 330336

||||||||||||||||||||||||||||||||||||||||||||||||||||||||||||

Sbjct 98889 AGCTTGCACTTTTCCTAGAGAAGAGTGGGTTTTCGAAGTATTTTCTCCACGTGAGCTGAA 98948

Query 330337 GGAGCTCTTGTCAAAGATCCATGCCGCCAACTCTAAGGATAAGCGTTGACCTTAGAAGAG 330396

|||||||||||||||||||||||||||||||||||||||| |||||||||||||||||||

Sbjct 98949 GGAGCTCTTGTCAAAGATCCATGCCGCCAACTCTAAGGATCAGCGTTGACCTTAGAAGAG 99008

Query 330397 CTCCTATTTGTGTTTTCTAATCCTTAGTTTTTTGAGAAAAGGTCCCTTCCTTCAAAATCA 330456

|||||||||||||||| |||||||||||||||||||||||||||||||||||||||||||

Sbjct 99009 CTCCTATTTGTGTTTTATAATCCTTAGTTTTTTGAGAAAAGGTCCCTTCCTTCAAAATCA 99068

Query 330457 TGATTGGGTCGACCAGGTAAGGCGGCGCGGCCCGATCTAGAGTGAATAGAAAAGAGAAAA 330516

||||||||||||||||||||||||||||||||||||||||||||||||||||||||||||

Sbjct 99069 TGATTGGGTCGACCAGGTAAGGCGGCGCGGCCCGATCTAGAGTGAATAGAAAAGAGAAAA 99128

Query 330517 TGTACATTGCTGTTCCAGCGGAAATACTTTGTTTAATTATACCACTTTTACTAGGAGTAG 330576

||||||||||||||||||||||||||||||||||||||||||||||||||||||||||||

Sbjct 99129 TGTACATTGCTGTTCCAGCGGAAATACTTTGTTTAATTATACCACTTTTACTAGGAGTAG 99188

Query 330577 CCTTTTTAGTGCTAGCTGAACGTAAAGTAATGGCTTTTGTGCAGCGTCGAAAGGGTCCTG 330636

||||||||||||||||||||||||||||||||||||||||||||||||||||||||||||

Sbjct 99189 CCTTTTTAGTGCTAGCTGAACGTAAAGTAATGGCTTTTGTGCAGCGTCGAAAGGGTCCTG 99248

Query 330637 ATGTAGTGGGAGCGTTCGGATTGTTACAACCTATAGCAGATGGTTTGAAATTGATTCTAA 330696

||||||||||||||||||||||||||||||||||||||||||||||||||||||||||||

Sbjct 99249 ATGTAGTGGGAGCGTTCGGATTGTTACAACCTATAGCAGATGGTTTGAAATTGATTCTAA 99308

Query 330697 AAGAACCTATTTCACCAAGTAGTGCGTCTTTCTTCCTTTTTAGAATGGCTCCAGTGGCTA 330756

||||||||||||||||||||||||||||||||||||||||||||||||||||||||||||

Sbjct 99309 AAGAACCTATTTCACCAAGTAGTGCGTCTTTCTTCCTTTTTAGAATGGCTCCAGTGGCTA 99368

Query 330757 CTTTTATGTTAAGTCTGGTCGCTTGGGCCGTTATACCTTTTGATTATGGTATGGTATTGT 330816

||||||||||||||||||||||||||||||||||||||||||||||||||||||||||||

Sbjct 99369 CTTTTATGTTAAGTCTGGTCGCTTGGGCCGTTATACCTTTTGATTATGGTATGGTATTGT 99428

Query 330817 CCGATTTGAACATAGGGCTACTTTATTTGTTTGCCATATCTTCGCTAGGTGTTTATGGAA 330876

||||||||||||||||||||||||||||||||||||||||||||||||||||||||||||

Sbjct 99429 CCGATTTGAACATAGGGCTACTTTATTTGTTTGCCATATCTTCGCTAGGTGTTTATGGAA 99488

Query 330877 TTATTATAGCAGGTTGGTCTAGTAAGACGGGGGGCGGCCGTTCGATCGCCTATGATAGAC 330936

||||||||||||||||||||||||||||||||||||||||||||||||||||||||||||

Sbjct 99489 TTATTATAGCAGGTTGGTCTAGTAAGACGGGGGGCGGCCGTTCGATCGCCTATGATAGAC 99548

Query 330937 GGACCAATTGGTCAAAAATGGGTTTGTGCCGCGGGTGTTGAACGAGCTACTCTACACAGG 330996

||||||||||||||||||||||||||||||||||||||||||||||||||||||||||||

Sbjct 99549 GGACCAATTGGTCAAAAATGGGTTTGTGCCGCGGGTGTTGAACGAGCTACTCTACACAGG 99608

Query 330997 TGTGGGCTTACAGGGCTAGGGCTCATCAACCCTTTCTTTCATTCATTCAAAGAGGTCGGT 331056

||||||||||||||||||||||||||||||||||||||||||||||||||||||||||||

Sbjct 99609 TGTGGGCTTACAGGGCTAGGGCTCATCAACCCTTTCTTTCATTCATTCAAAGAGGTCGGT 99668

Query 331057 CACGTTTCCGTAGGGATCGATAAGTTTAAGTCATAAAAAAGAGATGTTTATCTTCGCACC 331116

|||||||||||||||||||| |||||||||||||||||||||||||||||||||||||||

Sbjct 99669 CACGTTTCCGTAGGGATCGAGAAGTTTAAGTCATAAAAAAGAGATGTTTATCTTCGCACC 99728

Query 331117 TCAGATCCAGAGTCAAGGTAGCATGTCTCGTCTAACTCCAATTAGAGCTTTCTTTGGACC 331176

||||||||||||||||||||||||||||||||||||||||||||||||||||||||||||

Sbjct 99729 TCAGATCCAGAGTCAAGGTAGCATGTCTCGTCTAACTCCAATTAGAGCTTTCTTTGGACC 99788

Query 331177 GGCTGTTCTTCTTTGTCAACGCTACTATTAAACGTTTGACCTGACTGACTGACGGCCTTG 331236

||||||||||||||||||||||||||||||||||||||||||||||||||||||||||||

Sbjct 99789 GGCTGTTCTTCTTTGTCAACGCTACTATTAAACGTTTGACCTGACTGACTGACGGCCTTG 99848

Query 331237 ATGAATGAAAGAATTTCAAGGAAAAGGCAAGCAAGGCCCACTACAGTACAGTCGAAGTAA 331296

||||||||||||||||||||||||||||||||||||||||||||||||||||||||||||

Sbjct 99849 ATGAATGAAAGAATTTCAAGGAAAAGGCAAGCAAGGCCCACTACAGTACAGTCGAAGTAA 99908

Query 331297 GCGGCTGCCTGCCTCGCCTACTAAAAAGAAATAAATAGTAGGCGAGCGAGTTAGCGACAA 331356

||||||||||||||||||||||||||||||||||||||||||||||||||||||||||||

Sbjct 99909 GCGGCTGCCTGCCTCGCCTACTAAAAAGAAATAAATAGTAGGCGAGCGAGTTAGCGACAA 99968

Query 331357 AAAGGCTGAGGGCGTAGCGAGAGCGTCAGTACGTAGCCTTCATTCTACTACGTTAAGTAA 331416

||||||||||||||||||||||||||||||||||||||||||||||||||||||||||||

Sbjct 99969 AAAGGCTGAGGGCGTAGCGAGAGCGTCAGTACGTAGCCTTCATTCTACTACGTTAAGTAA 100028

Query 331417 AGTAACAACACTCGACGTTCAGAGAGTAAGTAAAGGGCCCCAAGAAACCGCTGTTCACAA 331476

||||||||||||||||||||||||||||||||||||||||||||||||||||||||||||

Sbjct 100029 AGTAACAACACTCGACGTTCAGAGAGTAAGTAAAGGGCCCCAAGAAACCGCTGTTCACAA 100088

Query 331477 GGTCTAATCTTTCGCTCCCCGGCCGCTACtttttttCGCAAGCTTTTTTCATTGTAAGCC 331536

||||||||||||||||||||||||||||||||||||||||||||||||||||||||||||

Sbjct 100089 GGTCTAATCTTTCGCTCCCCGGCCGCTACTTTTTTTCGCAAGCTTTTTTCATTGTAAGCC 100148

Query 331537 AACGACCCTCCCTGGGAATCCGTAAATCTGAGAGCATGCCGCaaaaaaaaGGATGGTTCC 331596

||||||||||||||||||||||||||||||||||||||||||||||||||||||||||||

Sbjct 100149 AACGACCCTCCCTGGGAATCCGTAAATCTGAGAGCATGCCGCAAAAAAAAGGATGGTTCC 100208

Query 331597 CTATGCATTGAATTCTTTCCGTTCCGGAAAGAATTCAAGTACCTTACCCCCCATCATGGT 331656

||||||||||||||||||||| ||||||||||||||||||||||||||||||||||||

Sbjct 100209 CTATGCATTGAATTCTTTCCGGAACGGAAAGAATTCAAGTACCTTACCCCCCATCATGGT 100268

Query 331657 GAACCTCTCCTTGTGATCGGGATGAGGTAGATGCCTCCTCCCAGCCgggggggCGGATCG 331716

||||||||||||||||||||||||||||||||||||||||||||||||||||||||||||

Sbjct 100269 GAACCTCTCCTTGTGATCGGGATGAGGTAGATGCCTCCTCCCAGCCGGGGGGGCGGATCG 100328

Query 331717 AATCAGAGTTTCCTTAGGTAGCCACCGACCTACAGTTATCCTTAAACTTCTGCGCTTGGT 331776

||||||||||||||||||||||||||||||||||||||||||||||||||||||||||||

Sbjct 100329 AATCAGAGTTTCCTTAGGTAGCCACCGACCTACAGTTATCCTTAAACTTCTGCGCTTGGT 100388

Query 331777 GGAAAAGAAGCGAACAAAGGTACGCTCGCTTGCTGTCTTGTTCTCTGCCGCGGACTGGGA 331836

||||||||||||||||||||||||||||||||||||||||||||||||||||||||||||

Sbjct 100389 GGAAAAGAAGCGAACAAAGGTACGCTCGCTTGCTGTCTTGTTCTCTGCCGCGGACTGGGA 100448

Query 331837 TCGCTCGCCAGCTAGGCCCTCTAGAACCAATCAAGTTGGAGCAAGATTGTATGAGAACAT 331896

||||||||||||||||||||||||||||||||||||||||||||||||||||||||||||

Sbjct 100449 TCGCTCGCCAGCTAGGCCCTCTAGAACCAATCAAGTTGGAGCAAGATTGTATGAGAACAT 100508

Query 331897 ATTACCCATTTTCGGGGACAAGGGGCGGAACGACCTCTCGATCTACTTACTGCAACCCAG 331956

||||||||||||||||||||||||||||||||||||||||||||||||||||||||||||

Sbjct 100509 ATTACCCATTTTCGGGGACAAGGGGCGGAACGACCTCTCGATCTACTTACTGCAACCCAG 100568

Query 331957 TACGAGCGTCGTCTAGGCGTGACCTCTTGTTCTGATATCCCCTACGCCTAGGGCGTTGTC 332016

||||||||||||||||||||||||||||||||||||||||||||||||||||||||||||

Sbjct 100569 TACGAGCGTCGTCTAGGCGTGACCTCTTGTTCTGATATCCCCTACGCCTAGGGCGTTGTC 100628

Query 332017 TGGGCCAAGAGCCATAGTGAATTGCTGTTTCTATTTAGTTTTTCTTTCTCGTTGTTGATA 332076

||||||||||||||||||||||||||||||||||||||||||||||||||||||||||||

Sbjct 100629 TGGGCCAAGAGCCATAGTGAATTGCTGTTTCTATTTAGTTTTTCTTTCTCGTTGTTGATA 100688

Query 332077 CCGGCAAGACCCAGCCAGATGATGATGTATGCTGGTTGGTAGTGAGAGGACTCTTAGTAC 332136

||||||||||||||||||||||||||||||||||||||||||||||||||||||||||||

Sbjct 100689 CCGGCAAGACCCAGCCAGATGATGATGTATGCTGGTTGGTAGTGAGAGGACTCTTAGTAC 100748

Query 332137 CCGAGGGCACTGGTaaaaaaaaaGAAGATGATTAAGTTTCTTGCTCCCCCTTAGCAGCGG 332196

||||||||||||||||||||||||||||||||||||||||||||||||||||||||||||

Sbjct 100749 CCGAGGGCACTGGTAAAAAAAAAGAAGATGATTAAGTTTCTTGCTCCCCCTTAGCAGCGG 100808

Query 332197 GAAAGGAGTCTATCTATCTGCCTAGCTTTGGTAGATTTCCCCCAACGC--aaaaaaaaaa 332254

|||||||||||||||||||||||||||||||||||||||||||||||| ||||||||||

Sbjct 100809 GAAAGGAGTCTATCTATCTGCCTAGCTTTGGTAGATTTCCCCCAACGCAAAAAAAAAAAA 100868

Query 332255 GAAATTCCAGCTAGCGCCCTGAGGTGGATAAGTCCGACGACTCAGCAGCAGTGCGGAATG 332314

||||||||||||||||||||||||||||||||||||||||||||||||||||||||||||

Sbjct 100869 GAAATTCCAGCTAGCGCCCTGAGGTGGATAAGTCCGACGACTCAGCAGCAGTGCGGAATG 100928

Query 332315 GGCTTTCTTCTCGTCCGGTGGATCTGATCTATAGTGAGGGGGCAATACATACCAAAAGGT 332374

||||||||||||||||||||||||||||||||||||||||||||||||||||||||||||

Sbjct 100929 GGCTTTCTTCTCGTCCGGTGGATCTGATCTATAGTGAGGGGGCAATACATACCAAAAGGT 100988

Query 332375 TCGAAGATGGAAGGCTTTTCTAAGAAGCTATAATAAATATGAAAATTTGAAGTCGAGAAA 332434

|||||||||||||||||||||||||||||||||||||||||||||||| |||||||||||

Sbjct 100989 TCGAAGATGGAAGGCTTTTCTAAGAAGCTATAATAAATATGAAAATTTTAAGTCGAGAAA 101048

Query 332435 AAAGAATAGGATGAGGTTACCTTGCTCAGATGAGGAGATATGGTAAAGGGATGCTGGGCT 332494

||||||||||||||||||||||||||||||||||||||||||||||||||||||||||||

Sbjct 101049 AAAGAATAGGATGAGGTTACCTTGCTCAGATGAGGAGATATGGTAAAGGGATGCTGGGCT 101108

Query 332495 GAACCATATTTCACTATTTGAGGCAAAGCTAATGGAAAGGGCGCTAAGCTCTGTTCCTTC 332554

||||||||||||||||||||||||||||||||||||||||||||||||||||||||||||

Sbjct 101109 GAACCATATTTCACTATTTGAGGCAAAGCTAATGGAAAGGGCGCTAAGCTCTGTTCCTTC 101168

Query 332555 ACGAGCTGCAACACCCTCCTCATTAGTCTAGTAGTCTCATCAGGCAGCTTGGGTGAGCCG 332614

||||||||||||||||||||||||||||||||||||||||||||||||||||||||||||

Sbjct 101169 ACGAGCTGCAACACCCTCCTCATTAGTCTAGTAGTCTCATCAGGCAGCTTGGGTGAGCCG 101228

Query 332615 CAAGAACGTTCGGTAGAACTCTGATTGTTGTAAGTGCCGGTACCTGCTGGTAAAGTTTCA 332674

||||||||||||||||||||||||||||||||||||||||||||||||||||||||||||

Sbjct 101229 CAAGAACGTTCGGTAGAACTCTGATTGTTGTAAGTGCCGGTACCTGCTGGTAAAGTTTCA 101288

Query 332675 TCTTTTGCACCGGCtaaaaaaatatatatttttagaaataatatataataataatataat 332734

||||||||||||||||||||||||||||||||||||||||||||||||||||||||||||

Sbjct 101289 TCTTTTGCACCGGCTAAAAAAATATATATTTTTAGAAATAATATATAATAATAATATAAT 101348

Query 332735 atTAACAGCTTTCTCTCTGttttttttttCTTCTAGAGCAGTCAGTTTGTTGGTTAACAT 332794

||||||||||||||||||| |||||||||||||||||||||||||||||||||||||||

Sbjct 101349 ATTAACAGCTTTCTCTCTG--TTTTTTTTCTTCTAGAGCAGTCAGTTTGTTGGTTAACAT 101406

Query 332795 AATATAGCAGGAAGTACTGCAAATTGAGTATTCTTTTACTAGTTCTTAACCAACCCACCA 332854

||||||||||||||||||||||||||||||||||||||||||||||||||||||||||||

Sbjct 101407 AATATAGCAGGAAGTACTGCAAATTGAGTATTCTTTTACTAGTTCTTAACCAACCCACCA 101466

Query 332855 AATCAAACAGCAAAAAATTGTGTCAAAAGTTGGACCAGAGGAGTAAACTGTGTACATACC 332914

||||||||||||||||||||||||||||||||||||||||||||||||||||||||||||

Sbjct 101467 AATCAAACAGCAAAAAATTGTGTCAAAAGTTGGACCAGAGGAGTAAACTGTGTACATACC 101526

Query 332915 GTTAATAAATAAAGGTCTTGAGAGAGAGACGTTTCTTATCTGGTGGATTCttttttttAT 332974

||||||||||||||||||||||||||||||||||||||||||||||||||||||||||||

Sbjct 101527 GTTAATAAATAAAGGTCTTGAGAGAGAGACGTTTCTTATCTGGTGGATTCTTTTTTTTAT 101586

Query 332975 TAAAGCAAGCAATTTccccccccAACTGTGCAGCGTTGAGCTAGTATAAAAGAAGGGATT 333034

||||||||||||||||||||||||||||||||||||||||||||||||||||||||||||

Sbjct 101587 TAAAGCAAGCAATTTCCCCCCCCAACTGTGCAGCGTTGAGCTAGTATAAAAGAAGGGATT 101646

Query 333035 ATATATCTGTACCCCTCCTCAAATCCCCCAGTCAAATCCCCCAGTTTGACCAGCACCCTA 333094

||||||||||||||||||||||||||||||||||||||||||||||||||||||||||||

Sbjct 101647 ATATATCTGTACCCCTCCTCAAATCCCCCAGTCAAATCCCCCAGTTTGACCAGCACCCTA 101706

Query 333095 ACCTCCCCTGAAACGAAAGTAATAATACCTACTATATACTAACCAATAATACTTAACGAA 333154

||||||||||||||||||||||||||||||||||||||||||||||||||||||||||||

Sbjct 101707 ACCTCCCCTGAAACGAAAGTAATAATACCTACTATATACTAACCAATAATACTTAACGAA 101766

Query 333155 CAAAGTCATTATATTCATATGATAAAGTACAAATACATGTTACATGGTAATTCAATGAAT 333214

||||||||||||||||||||||||||||||||||||||||||||||||||||||||||||

Sbjct 101767 CAAAGTCATTATATTCATATGATAAAGTACAAATACATGTTACATGGTAATTCAATGAAT 101826

Query 333215 ATATTATATTAAGGGTCTCTATTATTTTGGTACTAAGAAACTAAAATAATAATAGTTaaa 333274

|||||||||||||||| |||||||||||||||||||||||||||||||||||||||||||

Sbjct 101827 ATATTATATTAAGGGTATCTATTATTTTGGTACTAAGAAACTAAAATAATAATAGTTAAA 101886

Query 333275 aaaaaaTATTTAATATTTAGGATTCATAATGTCAATAGTATGCAGATTTTGAGCTCCACG 333334

||||||||||||||||||||||||||||||||||||||||||||||||||||||||||||

Sbjct 101887 AAAAAATATTTAATATTTAGGATTCATAATGTCAATAGTATGCAGATTTTGAGCTCCACG 101946

Query 333335 AAAATTCTTGTATGAGTGTAACATTCTAATAATACAAATGACAATAATATTTAATACATA 333394

||||||||||||||||||||||||||||||||||||||||||||||||||||||||||||

Sbjct 101947 AAAATTCTTGTATGAGTGTAACATTCTAATAATACAAATGACAATAATATTTAATACATA 102006

Query 333395 ATAGAAAAATACGTCATTATCAAAATAAGTAAAAATAGTTCCAACACACGAGTAATAATA 333454

||||||||||||||||||||||||||||||||||||||||||||||||||||||||||||

Sbjct 102007 ATAGAAAAATACGTCATTATCAAAATAAGTAAAAATAGTTCCAACACACGAGTAATAATA 102066

Query 333455 CTTACTATCAACAACCAGCAATACAACTTCCAAAATAAATTATCTCCTAAAGGATACTAG 333514

||||||||||||||||||||||||||||||||||||||||||||||||||||||||||||

Sbjct 102067 CTTACTATCAACAACCAGCAATACAACTTCCAAAATAAATTATCTCCTAAAGGATACTAG 102126

Query 333515 TTTTGCTTAAACAACAAAGCATTTATTCATACATAAAAAATCATTTTTATTGCATTGTCT 333574

||||||||||||||||||||||||||||||||||||||||||||||||||||||||||||

Sbjct 102127 TTTTGCTTAAACAACAAAGCATTTATTCATACATAAAAAATCATTTTTATTGCATTGTCT 102186

Query 333575 AACTAACTATAGTCAATCTCCTTCACCCTCACATTCATGTCGGCCCGATTAGTAAGAATC 333634

||||||||||||||||||||||||||||||||||||||||||||||||||||||||||||

Sbjct 102187 AACTAACTATAGTCAATCTCCTTCACCCTCACATTCATGTCGGCCCGATTAGTAAGAATC 102246

Query 333635 TTGAAAGCAACGAAGGTGCGCTCCTTTGTAATATCATCCTACATATAATTAATATACACT 333694

||||||||||||||||||||||||||||||||||||||||||||||||||||||||||||

Sbjct 102247 TTGAAAGCAACGAAGGTGCGCTCCTTTGTAATATCATCCTACATATAATTAATATACACT 102306

Query 333695 GATCaaatacataataaagaaagtacggatactaaaaatacacaagcaaaatatataata 333754

||||||||||||||||||||||||||||||||||||||||||||||||||||||||||||

Sbjct 102307 GATCAAATACATAATAAAGAAAGTACGGATACTAAAAATACACAAGCAAAATATATAATA 102366

Query 333755 aaatGTATTAACACTTTTTGTACAAAGGCTTAATATATAATTAAGAAAATATCAATGCCT 333814

||||||||||||||||||||||||||||||||||||||||||||||||||||||||||||

Sbjct 102367 AAATGTATTAACACTTTTTGTACAAAGGCTTAATATATAATTAAGAAAATATCAATGCCT 102426

Query 333815 AGCAAACAAAAGTTAAATACGTAATTAAGAAAGAATTATTACCTAACACACGTAGTAATT 333874

|||||||||||||| |||||||||||||||||||||||||||||||||||||||||||||

Sbjct 102427 AGCAAACAAAAGTTCAATACGTAATTAAGAAAGAATTATTACCTAACACACGTAGTAATT 102486

Query 333875 AAGAAAGTATTAATACCTAATAGAAACAATATATTTACAATATAGAAAAGTTTCAATTTA 333934

||||||||||||||||||||||||||||||||||||||||||||||||||||||||||||

Sbjct 102487 AAGAAAGTATTAATACCTAATAGAAACAATATATTTACAATATAGAAAAGTTTCAATTTA 102546

Query 333935 TCAAAATAGTATAAAACAGTATTAATACCTAACATAAACAATATATGTACAATATAGAAA 333994

|||||||||||||||||||||||||||||||||||||||||||||||||||||||| |||

Sbjct 102547 TCAAAATAGTATAAAACAGTATTAATACCTAACATAAACAATATATGTACAATATATAAA 102606

Query 333995 CTTTAAACTAACTCAGAAACTAATAATACTTATGACACTAACATATAATACATATCAAAC 334054

||||||||||||||||||||||||||||||||||||||||||||||||||||||||||||

Sbjct 102607 CTTTAAACTAACTCAGAAACTAATAATACTTATGACACTAACATATAATACATATCAAAC 102666

Query 334055 AAACATCAAATATATATAATGCAAATTTCTATAGGGCGCCACCGACCACAATAAATTAAG 334114

||||||||||||||||||||||||||||||||||||||||||||||||||||||||||||

Sbjct 102667 AAACATCAAATATATATAATGCAAATTTCTATAGGGCGCCACCGACCACAATAAATTAAG 102726

Query 334115 AAACATACTTCAAAGTTAAATCTCCCAGTCAAATCCTCCAATTTTGACCAACACCTTAAC 334174

||||||||||||||||||||||||||||||||||||||||||||||||||||||||||||

Sbjct 102727 AAACATACTTCAAAGTTAAATCTCCCAGTCAAATCCTCCAATTTTGACCAACACCTTAAC 102786

Query 334175 CTCCCATATTTAGAAGTAATAATACCTACTACTTTATAAACACACTAACTAATAATACAT 334234

||||||||||||||||||||||||||||||||||||||||||||||||||||||||||||

Sbjct 102787 CTCCCATATTTAGAAGTAATAATACCTACTACTTTATAAACACACTAACTAATAATACAT 102846

Query 334235 AACGAACAAAATAACTATATTTATATAATAAAGTGGAAATACATATTTCATTTCAACGTA 334294

||||||||||||||||||||||||||||||||||||||||||||||||||||||||||||

Sbjct 102847 AACGAACAAAATAACTATATTTATATAATAAAGTGGAAATACATATTTCATTTCAACGTA 102906

Query 334295 atttttctatatatataatatttaggatatattatattttggcatatctattaatgtaat 334354

|||||||||||||| |||||||||||||||||||||||||||||||||||||||||||||

Sbjct 102907 ATTTTTCTATATATCTAATATTTAGGATATATTATATTTTGGCATATCTATTAATGTAAT 102966

Query 334355 tttttttGCGTATCTATAATTCAAATGAAAATAATTATCGATACCTATTTTATCTTGTAA 334414

||||||||||||||||||||||||||||||||||||||||||||||||||||||||||||

Sbjct 102967 TTTTTTTGCGTATCTATAATTCAAATGAAAATAATTATCGATACCTATTTTATCTTGTAA 103026

Query 334415 TCAAATTAACTCATAATACTTAGCAATAAAAGTCACGATACAAAAATTAGGAACAATTAA 334474

||||||||||||||||||||||||||||||||||||||||||||||||| ||||||||||

Sbjct 103027 TCAAATTAACTCATAATACTTAGCAATAAAAGTCACGATACAAAAATTATGAACAATTAA 103086

Query 334475 TTTTTCTGATAGATCCTATTTGAACAACAATTCAAAATATCAAGTTTTAAAAGCATTCGT 334534

||||||||||||||||||||||||||||||||||||||||||||||||||||||||||||

Sbjct 103087 TTTTTCTGATAGATCCTATTTGAACAACAATTCAAAATATCAAGTTTTAAAAGCATTCGT 103146

Query 334535 CGAACAAATGTACACAATATTTCTATAAGCATCTTTAATCAAATCCACCTCTTGCATTTA 334594

||||||||||||||||||||||||||||||||||||||||||||||||||||||||||||

Sbjct 103147 CGAACAAATGTACACAATATTTCTATAAGCATCTTTAATCAAATCCACCTCTTGCATTTA 103206

Query 334595 CCAAATCAACCACAATAATGTAATTTCTAAATAATAAATATGATATGATAACTATAATCG 334654

||||||||||||||||||||||||| |||||||||||||||||||||||||||||||||

Sbjct 103207 CCAAATCAACCACAATAATGTAATTAATAAATAATAAATATGATATGATAACTATAATCG 103266

Query 334655 AGATTTCAAGTAAGAAGTAAATGCATTTTCTAATCGAGTTTTTACTTGAGCCTTGTACTC 334714

||||||||||||||||||||||||||||||||||||||||||||||||||||||||||||

Sbjct 103267 AGATTTCAAGTAAGAAGTAAATGCATTTTCTAATCGAGTTTTTACTTGAGCCTTGTACTC 103326

Query 334715 AACTTCTATTCATCCGTAAGAATCTTGTAAGCACTGGCGGTTCGTTCCTCAATAACTTGC 334774

||||||||||||||||||||||||||||||||||||||||||||||||||||||||||||

Sbjct 103327 AACTTCTATTCATCCGTAAGAATCTTGTAAGCACTGGCGGTTCGTTCCTCAATAACTTGC 103386

Query 334775 CCTACAATAATTAACAAAATAATTGTTAAAAATCCTTATTGAAGACAAAGTACCAATACA 334834

||||||||||||||||||||||||||||||||||||||||||||||||||||||||||||

Sbjct 103387 CCTACAATAATTAACAAAATAATTGTTAAAAATCCTTATTGAAGACAAAGTACCAATACA 103446

Query 334835 AGATATTAATACCATGCACACATATGCCAAATACACATAACTAAAAAGTACTAATATCTA 334894

||||||||||||||||||||||||||||||||||||||||||||||||||||||||||||

Sbjct 103447 AGATATTAATACCATGCACACATATGCCAAATACACATAACTAAAAAGTACTAATATCTA 103506

Query 334895 ACATATACATGAAAATATATAATTACAGAAGTACTAATACCTTGCAGCCATAGTAAAACA 334954

||||||||||||||||||||||||||||||||||||||||||||||||||||||||||||

Sbjct 103507 ACATATACATGAAAATATATAATTACAGAAGTACTAATACCTTGCAGCCATAGTAAAACA 103566

Query 334955 CATAATTAAGAAACTAATAATACCTATCATATACAGGTCAAAGACATAAATAGAAAAATA 335014

||||||||||||||||||||||||||||||||||||||||||||||||||||||||||||

Sbjct 103567 CATAATTAAGAAACTAATAATACCTATCATATACAGGTCAAAGACATAAATAGAAAAATA 103626

Query 335015 TCAATACCAATCACAAGGTCTTAATAAAATAGAAAATCCTAAATTAAACTAATCCAAGAT 335074

||||||||||||||||||||||||||||||||||||||||||||||||||||||||||||

Sbjct 103627 TCAATACCAATCACAAGGTCTTAATAAAATAGAAAATCCTAAATTAAACTAATCCAAGAT 103686

Query 335075 ACAATCAGAGTTTTAATGTTTGCAAAACACAAAACCAACCAATTAATCGAGAAAAATGGT 335134

||||||||||||||||||||||||||||||||||||||||||||||||||||||||||||

Sbjct 103687 ACAATCAGAGTTTTAATGTTTGCAAAACACAAAACCAACCAATTAATCGAGAAAAATGGT 103746

Query 335135 ATGCTTCTCCATCAATACAGCTACTACTGCCTAGTAGaaaaaaaaaaaTACATTTGCAAG 335194

||||||||||||||||||||||||||||||||||||| ||||||||||||||||||||

Sbjct 103747 ATGCTTCTCCATCAATACAGCTACTACTGCCTAGTAG---AAAAAAAATACATTTGCAAG 103803

Query 335195 TATCGATACCAGCTTAAACATATCATAACACAAATATATGAAAATGCAAATAGTGTGCGA 335254

||||||||||||||||||||||||||||||||||||||||||||||||||||||||||||

Sbjct 103804 TATCGATACCAGCTTAAACATATCATAACACAAATATATGAAAATGCAAATAGTGTGCGA 103863

Query 335255 AATGTGTAATAATACCACGATAACAGTAAATGACACTTCATAATATAAATCACCAATATA 335314

||||||||||||||||||||||||||||||||||||||||||||||||||||||||||||

Sbjct 103864 AATGTGTAATAATACCACGATAACAGTAAATGACACTTCATAATATAAATCACCAATATA 103923

Query 335315 TGTTTAAACAAGTCATAAGACAGAGTATACGAATAGTTAATACTTTCTGATACAGTATGT 335374

||||||||||||||||||||||||||||||||||||||||||||||||||||||||||||

Sbjct 103924 TGTTTAAACAAGTCATAAGACAGAGTATACGAATAGTTAATACTTTCTGATACAGTATGT 103983

Query 335375 AAATATATTTATACATATTTTAGAATCTTATTTAAAAGAGTACACAACATTTCACCTTGT 335434

||||||||||||||||||||||||||||||||||||||||||||||||||||||||||||

Sbjct 103984 AAATATATTTATACATATTTTAGAATCTTATTTAAAAGAGTACACAACATTTCACCTTGT 104043

Query 335435 ATTGAAACTTAGAGTAGATGTACCTTTAACCTCTTATTGTACCATTCTAACAATTTGTCG 335494

||||||||||||||||||||||||||||||||||||||||||||||||||||||||||||

Sbjct 104044 ATTGAAACTTAGAGTAGATGTACCTTTAACCTCTTATTGTACCATTCTAACAATTTGTCG 104103

Query 335495 GTATATATGATGCACCTAATAACTTGTATTGCAATTTCTAGGAATGAAGAAGAGAAGAAA 335554

||||||||||||||||||||||||||||||||||||||||||||||||||||||||||||

Sbjct 104104 GTATATATGATGCACCTAATAACTTGTATTGCAATTTCTAGGAATGAAGAAGAGAAGAAA 104163

Query 335555 TACCTTGCAGTGAGGCGTCAAGATCCTTGATTTCTAAGGAAACATCATCACGTGCCTACT 335614

|||||||||||||||| |||||||||||||||||||||||||||||||||||||||||||

Sbjct 104164 TACCTTGCAGTGAGGCATCAAGATCCTTGATTTCTAAGGAAACATCATCACGTGCCTACT 104223

Query 335615 AATCATGTCAAGCTTTCTATGAGAGAACAGGTAGCTTTCAATACAAATCTGTTAAAGAGA 335674

||||||||||||||||||||||||||||||||||||||||||||||||||||||||||||

Sbjct 104224 AATCATGTCAAGCTTTCTATGAGAGAACAGGTAGCTTTCAATACAAATCTGTTAAAGAGA 104283

Query 335675 GTATACCATTATATCCTATAGATATTAGAGCAGAACAATTCAAGCAAAGGTTGAACCCAA 335734

||||||||||||||||| ||||||||||||||||||||||||||||||||||||||||||

Sbjct 104284 GTATACCATTATATCCTCTAGATATTAGAGCAGAACAATTCAAGCAAAGGTTGAACCCAA 104343

Query 335735 AGTGTCAGTAAGCATGCAGGGACATGTTGAATGAAGTACAAGATTAGACAAATGAATCAA 335794

||||||||||||||||||||||||||||||||||||||||||||||||||||||||||||

Sbjct 104344 AGTGTCAGTAAGCATGCAGGGACATGTTGAATGAAGTACAAGATTAGACAAATGAATCAA 104403

Query 335795 GTAAATACATTAAATTGCAATAGCAACaaatagaaataaaagaaaagaagaatgttagaa 335854

||||||||||||||||||||||||||||||||||||||||||||||||||||||||||||

Sbjct 104404 GTAAATACATTAAATTGCAATAGCAACAAATAGAAATAAAAGAAAAGAAGAATGTTAGAA 104463

Query 335855 gaccagaaaaaaaaTTATGAACAATTAAACCAATAGACAGTCAGGATAGTAAAAAGATCA 335914

||||||||||||||||||||||||||||||||||||||||||||||||||||||||||||

Sbjct 104464 GACCAGAAAAAAAATTATGAACAATTAAACCAATAGACAGTCAGGATAGTAAAAAGATCA 104523

Query 335915 GTTGAATCAGAGAAAGAGTAATTAATCAAAGAGGAAGAGCAAACCCGGCAAAGATCGGAG 335974

||||||||||||||||||||||||||||||||||||||||||||||||||||||||||||

Sbjct 104524 GTTGAATCAGAGAAAGAGTAATTAATCAAAGAGGAAGAGCAAACCCGGCAAAGATCGGAG 104583

Query 335975 GAGTAAAGAGTATTCACTTAGACTATCTATTTACTTTACATTTCGATCCATCTTTCAAAT 336034

||||||||||||||||||||||||||||||||||||||||||||||||||||||||||||

Sbjct 104584 GAGTAAAGAGTATTCACTTAGACTATCTATTTACTTTACATTTCGATCCATCTTTCAAAT 104643

Query 336035 AT-TAGATTTAACATATGATCCAAAAGAGAACCGATCTATATATCTCTACATCCACAAAC 336093

|| |||||||||||||||||||||||||||||||||||||||||||||||||||||||

Sbjct 104644 ATCGCGATTTAACATATGATCCAAAAGAGAACCGATCTATATATCTCTACATCCACAAAC 104703

Query 336094 AAAACCAGACTAAATACAAAACAATCAAGTACCAAAACAACGGATAAGAAGCATCAAAAG 336153

||||||||||||||||||||||||||||||||||||||||||||||||||||||||||||

Sbjct 104704 AAAACCAGACTAAATACAAAACAATCAAGTACCAAAACAACGGATAAGAAGCATCAAAAG 104763

Query 336154 ATAGCTTAGCTGTGAGTTACATCATTGCATCTACCATCAAGAATTTCATGCTCAGTTGAT 336213

||||||||||||||||||||||||||||||||||||||||||||||||||||||||||||

Sbjct 104764 ATAGCTTAGCTGTGAGTTACATCATTGCATCTACCATCAAGAATTTCATGCTCAGTTGAT 104823

Query 336214 TTTAGTACATCACTGTCCAAATCATGCTGTGACACTCCAAATAGGTTATAAAAGATTTCA 336273

||||||||||||||||||||||||||||||||||||||||||||||||||||||||||||

Sbjct 104824 TTTAGTACATCACTGTCCAAATCATGCTGTGACACTCCAAATAGGTTATAAAAGATTTCA 104883

Query 336274 GAACAAGGAAGTACCAGTTAAAATTAGTTGTCACAAGGGGGCAATAATTGAAATTAATGG 336333

||||||||||||||||||| ||||||||||||||||||||||||||||||||||||||||

Sbjct 104884 GAACAAGGAAGTACCAGTTCAAATTAGTTGTCACAAGGGGGCAATAATTGAAATTAATGG 104943

Query 336334 AGTTGGAGCACTTACAGCTTCTCTACAGTTCCTGTAATTGGTGGACGAGATACAGGTGGC 336393

||||||||||||||||||||||||||||||||||||||||||||||||||||||||||||

Sbjct 104944 AGTTGGAGCACTTACAGCTTCTCTACAGTTCCTGTAATTGGTGGACGAGATACAGGTGGC 105003

Query 336394 GGATATGCTGAGAAAGGGGCGGAGATCTTAGACTGAGATGGCTAATCGGGCATGAATTAT 336453

||||||||||||||||||||||||||||||||||||||||||||||||||||||||||||

Sbjct 105004 GGATATGCTGAGAAAGGGGCGGAGATCTTAGACTGAGATGGCTAATCGGGCATGAATTAT 105063

Query 336454 GACATCAGTAGATGACTGATACAGTATAGCGAGAGGGATAAAAATTCCGCGGAAATGCTG 336513

||||||||||||||||||||||||||||||||||||||||||||||||||||||||||||

Sbjct 105064 GACATCAGTAGATGACTGATACAGTATAGCGAGAGGGATAAAAATTCCGCGGAAATGCTG 105123

Query 336514 CTATCGGAGAAGCCCCTTGCCTCCTCCTCCATTAACATATTCAGCACAATCGGCTCCTTG 336573

||||||||||||||||||||||||||||||||||||||||||||||||||||||||||||

Sbjct 105124 CTATCGGAGAAGCCCCTTGCCTCCTCCTCCATTAACATATTCAGCACAATCGGCTCCTTG 105183

Query 336574 CCGCGGAGAACTGGAGATCATCGGAGAACCGCTTGGTGAACTATGAATACCTGCTAAACA 336633

||||||||||||||||||||||||||||||||||||||||||||||||||||||||||||

Sbjct 105184 CCGCGGAGAACTGGAGATCATCGGAGAACCGCTTGGTGAACTATGAATACCTGCTAAACA 105243

Query 336634 AAGTTTAAGTATCTATACAGTATTTTATTAATACTTGCATAAACAAGATATAGAATAATC 336693

||||||||||||||||||||||||||||||||||||||||||||||||||||||||||||

Sbjct 105244 AAGTTTAAGTATCTATACAGTATTTTATTAATACTTGCATAAACAAGATATAGAATAATC 105303

Query 336694 TAATAATATAGCCCCCTTTAAAATTGTTTTATATCATATTAATGAACAAATATAACGAGC 336753

||||||||||| ||||||||||||||||||||||||||||||||||||||||||||||||

Sbjct 105304 TAATAATATAG-CCCCTTTAAAATTGTTTTATATCATATTAATGAACAAATATAACGAGC 105362

Query 336754 TAGCAAAATGAAAGAACATTAGTTTGAGAAAAGCTTTGTTCTATCACATAGATAACTCAG 336813

||||||||| ||||||||||||||||||||||||||||||||||||||||||||||||||

Sbjct 105363 TAGCAAAATTAAAGAACATTAGTTTGAGAAAAGCTTTGTTCTATCACATAGATAACTCAG 105422

Query 336814 AAATCTACATTAAAATTAGAAACGGAAATTCTAGTAAATAGTAAAAAGGATTAGATCAAA 336873

||||||||||||||||||||||||||||||||||||||||||||||||||||||||||||

Sbjct 105423 AAATCTACATTAAAATTAGAAACGGAAATTCTAGTAAATAGTAAAAAGGATTAGATCAAA 105482

Query 336874 CATTATAATACCTTTATCATTTTGAAAATAAGAGCATCCGGTAGCAAACCTTGATTTCTT 336933

||||||||||||||||||||||| |||||||||||||||||||||||||||||||||||

Sbjct 105483 CATTATAATACCTTTATCATTTTTCAAATAAGAGCATCCGGTAGCAAACCTTGATTTCTT 105542

Query 336934 CATCTCCCTTCTCAAGCTACAGCATCCCTATGGACCCTATCTCGATTTCCATAATCTATA 336993

||||||||||||||||||||||||||||||||||||||||||||||||||||||||||||

Sbjct 105543 CATCTCCCTTCTCAAGCTACAGCATCCCTATGGACCCTATCTCGATTTCCATAATCTATA 105602

Query 336994 TTGCCTCTAATCCGCATTCAGTTTGATTATTTTGTTCATCGTCAAAGTCTGAAGATATTA 337053

||||||||||||||||||||||||||||||||||||||||||||||||||||||||||||

Sbjct 105603 TTGCCTCTAATCCGCATTCAGTTTGATTATTTTGTTCATCGTCAAAGTCTGAAGATATTA 105662

Query 337054 TGAAGTAAAAATTGTAGTGAATAGGAAGCAAAAAAGCACATCAAAATAACCTTAATTAGG 337113

||||||||||||||||||||||||||||||||||||||||||||||||||||||||||||

Sbjct 105663 TGAAGTAAAAATTGTAGTGAATAGGAAGCAAAAAAGCACATCAAAATAACCTTAATTAGG 105722

Query 337114 TTTATCTCCGATTAGGAAAAGTGAGGTACGAGAGGGAGAAGGATTGACCGAAAAGTACAT 337173

||||||||||||||| ||||||||||||||||||||||||||||||||||||||||||||

Sbjct 105723 TTTATCTCCGATTAGTAAAAGTGAGGTACGAGAGGGAGAAGGATTGACCGAAAAGTACAT 105782

Query 337174 AAGAAAGAGTCGCCATTAGAATGAGAGTTGCGAGATCGCCGAGAGCATCGACGTCGGTTC 337233

||||||||||||||||||||||||||||||||||||||||||||||||||||||||||||

Sbjct 105783 AAGAAAGAGTCGCCATTAGAATGAGAGTTGCGAGATCGCCGAGAGCATCGACGTCGGTTC 105842

Query 337234 GAGAGCCATCAGTGCGGAGTAGAGCATTAGATCGATATGTGAGATCGCCGGAGTAGAGGG 337293

||||||||||||||||||||||||||||||||||||||||||||||||||||||||||||

Sbjct 105843 GAGAGCCATCAGTGCGGAGTAGAGCATTAGATCGATATGTGAGATCGCCGGAGTAGAGGG 105902

Query 337294 TCGACATCGGTCCGACAGCTACCACTGCCGGAGTAGAGCGTCATCGTATATGACCTCGAT 337353

||||||||||||||||||||||||||||||||||||||||||||||||||||||||||||

Sbjct 105903 TCGACATCGGTCCGACAGCTACCACTGCCGGAGTAGAGCGTCATCGTATATGACCTCGAT 105962

Query 337354 CTACATCTCCCCATCGGTACTTGGAGTTCCCCTCTGACTCCATGGAAGAAATAAATCGCA 337413

||||||||||||||||||||||||||||||||||||||||||||||||||||||||||||

Sbjct 105963 CTACATCTCCCCATCGGTACTTGGAGTTCCCCTCTGACTCCATGGAAGAAATAAATCGCA 106022

Query 337414 CAGAAAAGAAGAGAAATGGATAGAAGCGTTGGAGAAGAGAAGGTAAAAATGTAATGGTAA 337473

||||||||||||||||||||||||||||||||||||||||||||||||||||||||||||

Sbjct 106023 CAGAAAAGAAGAGAAATGGATAGAAGCGTTGGAGAAGAGAAGGTAAAAATGTAATGGTAA 106082

Query 337474 TTGTAAGTGGGCCCAATGGCCCATAATAAGACCTTGATGGATTGATTTGGGGATTGGTTT 337533

||||||||||||||||||||||||||||||||||||||||||||||||||||||||||||

Sbjct 106083 TTGTAAGTGGGCCCAATGGCCCATAATAAGACCTTGATGGATTGATTTGGGGATTGGTTT 106142

Query 337534 AGAAAGAAGCGATGGTTGTGGACAGTTTATCGGGATAGTTGAGCCGTTGGGGTACGATAG 337593

||||||||||||||||||||||||||||||||||||||||||||||||||||||||||||

Sbjct 106143 AGAAAGAAGCGATGGTTGTGGACAGTTTATCGGGATAGTTGAGCCGTTGGGGTACGATAG 106202

Query 337594 CGGGGGATTGGAGTCCGGGGGATCTTACAATTACTGATAAAAGAATCCTCTGACCTATCA 337653

||||||||||||||||||||||||||||||||||||||||||||||||||||||||||||

Sbjct 106203 CGGGGGATTGGAGTCCGGGGGATCTTACAATTACTGATAAAAGAATCCTCTGACCTATCA 106262

Query 337654 CTCGTATAAGGCTTACTTCAATGGGAAAATACCTCATGGTAACATAAATCAAATACTAAA 337713

||||||||||||||||||||||||||||||||||||||||||||||||||||||||||||

Sbjct 106263 CTCGTATAAGGCTTACTTCAATGGGAAAATACCTCATGGTAACATAAATCAAATACTAAA 106322

Query 337714 ACTGCTTGGCAAACCGCTGACGATCAATGTAAAGACTTGACTAGAGAAACTGAATGTTCC 337773

||||||||||||||||||||||||||||||||||||||||||||||||||||||||||||

Sbjct 106323 ACTGCTTGGCAAACCGCTGACGATCAATGTAAAGACTTGACTAGAGAAACTGAATGTTCC 106382

Query 337774 CCAATTTaagaatagaaaagcgtgtgaaaagaagaaagaagagaaaTTGGGCTTTCGCGA 337833

|||||||||||||| |||||||||||||||||||||||||||||||||||||||||||||

Sbjct 106383 CCAATTTAAGAATATAAAAGCGTGTGAAAAGAAGAAAGAAGAGAAATTGGGCTTTCGCGA 106442

Query 337834 GAGAGACTGCCATCTCTCAGGTCTTATACTTCTAGTCGACTGCTCTATGACGGTCTGACC 337893

||||||||||||||||||||||||||||||||||||||||||||||||||||||||||||

Sbjct 106443 GAGAGACTGCCATCTCTCAGGTCTTATACTTCTAGTCGACTGCTCTATGACGGTCTGACC 106502

Query 337894 TCGTTACTGGGCTCTGCTCGCTCATCTACGCTCCGACCAAGGAACGATGTTTCTTTTCaa 337953

|||||||||||||||||||||||||||||||| |||||||||||||||||||||||||||

Sbjct 106503 TCGTTACTGGGCTCTGCTCGCTCATCTACGCTGCGACCAAGGAACGATGTTTCTTTTCAA 106562

Query 337954 aaagttcaaaaagtatagtagtaaaaaaaaaaGTGCACTTTTTTCAGATATTTATATTGT 338013

||||||||||||||||||||||||||||||||||||||||||||||||||||||||||||

Sbjct 106563 AAAGTTCAAAAAGTATAGTAGTAAAAAAAAAAGTGCACTTTTTTCAGATATTTATATTGT 106622

Query 338014 TGGGGGGAGGGATCCCTATCTTTATTTCTATCCAAATAGCCTTTTATATAGATCTCTATA 338073

||||||||||||||||||||||||||||||||||||||||||||||||||||||||||||

Sbjct 106623 TGGGGGGAGGGATCCCTATCTTTATTTCTATCCAAATAGCCTTTTATATAGATCTCTATA 106682

Query 338074 GATCTATCTATATACTATCCTCCTCCATCCGGATAGAGATGTCCCTATGAGCGACTATGC 338133

||||||||||||||||||||||||||||||||||||||||||||||||||||||||||||

Sbjct 106683 GATCTATCTATATACTATCCTCCTCCATCCGGATAGAGATGTCCCTATGAGCGACTATGC 106742

Query 338134 CTGTCTTTTCTTATATGGGTTGACCACGTCCGTTTCCGCTCCAAAGAAGAAGGTTGATAT 338193

||||||||||||||||||||||||||||||||||||||||||||||||||||||||||||

Sbjct 106743 CTGTCTTTTCTTATATGGGTTGACCACGTCCGTTTCCGCTCCAAAGAAGAAGGTTGATAT 106802

Query 338194 CTTTGAATTTTATGTCGTGAGGGATGTGACCGATGTTAGGTCCATAAAATACCACAGCTT 338253

||||||||||||||||||||||||||||||||||||||||||||||||||||||||||||

Sbjct 106803 CTTTGAATTTTATGTCGTGAGGGATGTGACCGATGTTAGGTCCATAAAATACCACAGCTT 106862

Query 338254 TTAGTGTTCTATGATTCACCTCCGAATAATGAGTAGGTAGTTGGATCCTTTTTATTCTTT 338313

||||||||||||||||||||||||||||||||||||||||||||||||||||||||||||

Sbjct 106863 TTAGTGTTCTATGATTCACCTCCGAATAATGAGTAGGTAGTTGGATCCTTTTTATTCTTT 106922

Query 338314 TTAGTAGTTCGATCCTTTTTATTCTGATCTTCCTAGTAAAAgggggggATCCATAGCAAT 338373

||||||||||||||||||||||||||||||||||||||||||||||||||||||||||||

Sbjct 106923 TTAGTAGTTCGATCCTTTTTATTCTGATCTTCCTAGTAAAAGGGGGGGATCCATAGCAAT 106982

Query 338374 ACTTATATaaagaagaaaaagtacttcttcttataaaaggacttcttcttctaaaaggac 338433

||||||||||||||||||||||||||||||||||||||||||||||||||||||||||||

Sbjct 106983 ACTTATATAAAGAAGAAAAAGTACTTCTTCTTATAAAAGGACTTCTTCTTCTAAAAGGAC 107042

Query 338434 ttctttttcttttaaaaggcagatttttcAATAAGAAGTGGTATTTTAAATGaaaaaaaT 338493

||||||||||||||||||||||||||||||||||||||||||||||||||||||||||||

Sbjct 107043 TTCTTTTTCTTTTAAAAGGCAGATTTTTCAATAAGAAGTGGTATTTTAAATGAAAAAAAT 107102

Query 338494 TCCTTATAATTCTATTGTGCTCAGCGAAGTAACTTCCTAAGCATACTTTTACGGATCCAA 338553

||||||||||||||||||||||||||||||||||||||||||||||||||||||||||||

Sbjct 107103 TCCTTATAATTCTATTGTGCTCAGCGAAGTAACTTCCTAAGCATACTTTTACGGATCCAA 107162

Query 338554 ACAACTTCTTTGTTCTTTCTAAGTCTTCtttttttATAGAAGAGCGCAACTCTTTCAAAA 338613

||||||||||||||||||||||||||||||||||||||||||||||||||||||||||||

Sbjct 107163 ACAACTTCTTTGTTCTTTCTAAGTCTTCTTTTTTTATAGAAGAGCGCAACTCTTTCAAAA 107222

Query 338614 AGCGGGATTTTAGTAGTAGCCGACACTTCCtttttttttttAGTAAGCGGAACCATCCTA 338673

||||||||||||||||||||||||||||||||||||||||||||||||||||||||||||

Sbjct 107223 AGCGGGATTTTAGTAGTAGCCGACACTTCCTTTTTTTTTTTAGTAAGCGGAACCATCCTA 107282

Query 338674 TTTTGGTTCTTCTCCACCATCTGACCGGGCGGGATTTTCTTATTATTTTTCCAACTAATA 338733

|||||||||||||||||||||||||||| |||||||||||||||||||||||||||||||

Sbjct 107283 TTTTGGTTCTTCTCCACCATCTGACCGGACGGGATTTTCTTATTATTTTTCCAACTAATA 107342

Query 338734 TGTCAATATAGAAAGATCTCCTTATTTCTTCACTGCGGGTTCTCACCTCATTTTCTTGAA 338793

||||||||||||||||||||||||||||||||||||||||||||||||||||||||||||

Sbjct 107343 TGTCAATATAGAAAGATCTCCTTATTTCTTCACTGCGGGTTCTCACCTCATTTTCTTGAA 107402

Query 338794 AAGATATGAGATCACCATGGGAAACTTTCCAATGAGTAATGCTTACCAGTCTATTATTCA 338853

||||||||||||||||||||||||||||||||||||||||||||||||||||||||||||

Sbjct 107403 AAGATATGAGATCACCATGGGAAACTTTCCAATGAGTAATGCTTACCAGTCTATTATTCA 107462

Query 338854 CACAAACCTTTCGATGACTTATCAGCTGCCTTGCTTGAGGAAGAGTTTTACTAAAATGGA 338913

||||||||||||||||||||||||||||||||||||||||||||||||||||||||||||

Sbjct 107463 CACAAACCTTTCGATGACTTATCAGCTGCCTTGCTTGAGGAAGAGTTTTACTAAAATGGA 107522

Query 338914 GACGAACTAAAATCACGTCTAATCTTGTTTCTTGATTGAGTAAAAAAGGGATATATGAAG 338973

||||||||||||||||||||||||||||||||||||||||||||||||||||||||||||

Sbjct 107523 GACGAACTAAAATCACGTCTAATCTTGTTTCTTGATTGAGTAAAAAAGGGATATATGAAG 107582

Query 338974 TTCGTTCTCTTCCTCTGTGCATCTCCCTTATGGGTAAACCCCCATAAAAAAGGGACAACT 339033

||||||||||||||||||||||||||||||||||||||||||||||||||||||||||||

Sbjct 107583 TTCGTTCTCTTCCTCTGTGCATCTCCCTTATGGGTAAACCCCCATAAAAAAGGGACAACT 107642

Query 339034 TTCGTGTAGTTTGTAATTTGATGTTACTGTTTAGATTTTCTCTCATAGAAAGATTTCTTT 339093

||||||||||||||||||||||||||||||||||||||||||||||||||||||||||||

Sbjct 107643 TTCGTGTAGTTTGTAATTTGATGTTACTGTTTAGATTTTCTCTCATAGAAAGATTTCTTT 107702

Query 339094 TAATGGATTTCCTCTTTTTCCTCAATCTTCGGAGAATGCGGCGTTGTATTATAGAAAGTT 339153

||||||||||||||||||||||||||||||||||||||||||||||||||||||||||||

Sbjct 107703 TAATGGATTTCCTCTTTTTCCTCAATCTTCGGAGAATGCGGCGTTGTATTATAGAAAGTT 107762

Query 339154 CTCTGTTCCAAACATTTCCTGGAAGTAGACGACACGTTTTAAATCTTAATGCAGGCATGG 339213

||||||||||||||||||||||||||||||||||||||||||||||||||||||||||||

Sbjct 107763 CTCTGTTCCAAACATTTCCTGGAAGTAGACGACACGTTTTAAATCTTAATGCAGGCATGG 107822

Query 339214 CATATCTCGAACAATCAGTCTTTTTTGCCACATCGCGTAGATAGAAAGGGAATCAATCAA 339273

||||||||||||||||||||||||||||||||||||||||||||||||||||||||||||

Sbjct 107823 CATATCTCGAACAATCAGTCTTTTTTGCCACATCGCGTAGATAGAAAGGGAATCAATCAA 107882

Query 339274 AGCCAACTCTTCCACGAACCCCTCACGAATGGTACTCCCTTAACTCAATGAACTATGAGA 339333

||||||||||||||||||||||||||||||||||||||||||||||||||||||||||||

Sbjct 107883 AGCCAACTCTTCCACGAACCCCTCACGAATGGTACTCCCTTAACTCAATGAACTATGAGA 107942

Query 339334 ATTCCTTTGCCTAGTGTTTTCAGTAATCCGCTCCGCGCCTTACTAGCTGAAACTCAATAC 339393

||||||||||||||||||||||||||||||||||||||||||||||||||||||||||||

Sbjct 107943 ATTCCTTTGCCTAGTGTTTTCAGTAATCCGCTCCGCGCCTTACTAGCTGAAACTCAATAC 108002

Query 339394 TTGACCGCTAACCAAACACTACTTACGAGTTTCTGACCAAACACTATATACGAGTTTCAC 339453

||||||||||||||||||||||||||||||||||||||||||||||||||||||||||||

Sbjct 108003 TTGACCGCTAACCAAACACTACTTACGAGTTTCTGACCAAACACTATATACGAGTTTCAC 108062

Query 339454 CAAAGCaaaaaaaGAAGCTTTTTCGCAGTGCGCCGTCGGGTGCTAAGAGCACAGCAGACA 339513

||||||||||||||||||||||||||||||||||||||||||||||||||||||||||||

Sbjct 108063 CAAAGCAAAAAAAGAAGCTTTTTCGCAGTGCGCCGTCGGGTGCTAAGAGCACAGCAGACA 108122

Query 339514 TCAAGGTGCGAAGGGAGAGAACGGAGTGAAGATGGAGTAAGGGGATCACCGTGGGTTCAA 339573

||||||||||||||||||||||||||||||||||||||||||||||||||||||||||||

Sbjct 108123 TCAAGGTGCGAAGGGAGAGAACGGAGTGAAGATGGAGTAAGGGGATCACCGTGGGTTCAA 108182

Query 339574 GGAGAATGGAAGCTCTGCTACGCTAGATCCACGAGAGGATGCTGGGCTTAAGATATAAGG 339633

||||||||||||||||||||||||||||||||||||||||||||||||||||||||||||

Sbjct 108183 GGAGAATGGAAGCTCTGCTACGCTAGATCCACGAGAGGATGCTGGGCTTAAGATATAAGG 108242

Query 339634 CCCTCTTCTCTTTTTCGCTACGCGACCAAGATTCTCATGAGATGGGATTCCTCCTCCTGC 339693

||||||||||||||||||||||||||||||||||||||||||||||||||||||||||||

Sbjct 108243 CCCTCTTCTCTTTTTCGCTACGCGACCAAGATTCTCATGAGATGGGATTCCTCCTCCTGC 108302

Query 339694 TCGGGGTTCAGCCAGCGAATAAGTTAGCTAAGCTGCCTAGTAGGCGAGTCTGATAAGTGT 339753

||||||||||||||||||||||||||||||||||||||||||||||||||||||||||||

Sbjct 108303 TCGGGGTTCAGCCAGCGAATAAGTTAGCTAAGCTGCCTAGTAGGCGAGTCTGATAAGTGT 108362

Query 339754 CAAACAAAAGTTCCAGGCACCGACGGATTCTCAGGTGAACCTTAGAGAAGTGGACTGGGA 339813

||||||||||||||||||||||||||||||||||||||||||||||||||||||||||||

Sbjct 108363 CAAACAAAAGTTCCAGGCACCGACGGATTCTCAGGTGAACCTTAGAGAAGTGGACTGGGA 108422

Query 339814 TGGTAGTAGAGGGCCCGCCCGAGGGCAGAAGGTGAAAGGCCTAGAGTAGAGGGGTAGGTG 339873

|||||||||||| ||||||||||||||||||||||||||||||||||||||||||||

Sbjct 108423 TGGTAGTAGAGG----GCCCGAGGGCAGAAGGTGAAAGGCCTAGAGTAGAGGGGTAGGTG 108478

Query 339874 ACTAGGCTTATCTATAGAATGACTTTCTCAAGTGACATCCTTTCCATTCTTTTGGAGAGA 339933

||||||||||||||||||||||||||||||||||||||||||||||||||||||||||||

Sbjct 108479 ACTAGGCTTATCTATAGAATGACTTTCTCAAGTGACATCCTTTCCATTCTTTTGGAGAGA 108538

Query 339934 ACTCGACTACTAGAAGGCACTAGACTCTGGGCTCCTCGTTCATCGGGAAATGGGCCTGAA 339993

||||||||||||||||||||||||||||||||||||||||||||||||||||||||||||

Sbjct 108539 ACTCGACTACTAGAAGGCACTAGACTCTGGGCTCCTCGTTCATCGGGAAATGGGCCTGAA 108598

Query 339994 ACCGCGCTGCATATCTTCGGCTTCGCCTAAGGCGCGGAGCGGTTTGTTAAGTTAGTTCTA 340053

||||||||||||||||||||||||||||||||||||||||||||||||||||||||||||

Sbjct 108599 ACCGCGCTGCATATCTTCGGCTTCGCCTAAGGCGCGGAGCGGTTTGTTAAGTTAGTTCTA 108658

Query 340054 TCAGTTCAAAACAAGTTGACAAGTAATCGCATCACCCTGCGGCCTTTGTCTGCTTGGTGG 340113

||||||||||||||||||||||||||||||||||||||||||||||||||||||||||||

Sbjct 108659 TCAGTTCAAAACAAGTTGACAAGTAATCGCATCACCCTGCGGCCTTTGTCTGCTTGGTGG 108718

Query 340114 ACGACTTCTCACCGCACTGGTTGGCTAGCATACCTGTACCCATTTGAACTTTTTTGTATC 340173

||||||||||||||||||||||||||||||||||||||||||||||||||||||||||||

Sbjct 108719 ACGACTTCTCACCGCACTGGTTGGCTAGCATACCTGTACCCATTTGAACTTTTTTGTATC 108778

Query 340174 GACTTCATATCCCCAACAAAAGACTTTACAGGCGCTACGCGTTGACTCTACACGCCTGCG 340233

||||||||||||||||||||||||||||||||||||||||||||||||||||||||||||

Sbjct 108779 GACTTCATATCCCCAACAAAAGACTTTACAGGCGCTACGCGTTGACTCTACACGCCTGCG 108838

Query 340234 GGCTTGGTTTACATTTTCTTCTTATGAAGAGATTGGCAAAGATGTTTTTTGGCCTCCAGG 340293

||||||||||||||||||||||||||||||||||||||||||||||||||||||||||||

Sbjct 108839 GGCTTGGTTTACATTTTCTTCTTATGAAGAGATTGGCAAAGATGTTTTTTGGCCTCCAGG 108898

Query 340294 AGCGAAGCTACAAAGCGTTTCCATCCTGTGCCCTTTCTCTTGAAATCAAAAGTACCTCTC 340353

||||||||||||||||||||||||||||||||||||||||||||||||||||||||||||

Sbjct 108899 AGCGAAGCTACAAAGCGTTTCCATCCTGTGCCCTTTCTCTTGAAATCAAAAGTACCTCTC 108958

Query 340354 CTTTTCTTGTGGACCTCCTGTATAAGCCTGCTTGTTGCTTGGATTTGAGGGAGCGCGGGC 340413

||||||||||||||||||||||||||||||||||||||||||||||||||||||||||||

Sbjct 108959 CTTTTCTTGTGGACCTCCTGTATAAGCCTGCTTGTTGCTTGGATTTGAGGGAGCGCGGGC 109018

Query 340414 TCAGTTGGTTGGAGGCCCGACTTGATTGAATACCTATGGAGGGCGGAGCTTTTTATTGAG 340473

||||||||||||||||||||||||||||||||||||||||||||||||||||||||||||

Sbjct 109019 TCAGTTGGTTGGAGGCCCGACTTGATTGAATACCTATGGAGGGCGGAGCTTTTTATTGAG 109078

Query 340474 AATGTGATCACTTGGTTTCTTCTTGTCATGGTGAATGGCTTTGTCTTCTTTTATCAAGCA 340533

||||||||||||||||||||||||||||||||||||||||||||||||||||||||||||

Sbjct 109079 AATGTGATCACTTGGTTTCTTCTTGTCATGGTGAATGGCTTTGTCTTCTTTTATCAAGCA 109138

Query 340534 AGGTTATGTTCTCTTGAGTAGTAGATGGAACGGTATCTTTTGTAGCAGGAATGCTATCTC 340593

||||||||||||||||||||||||||||||||||||||||||||||||||||||||||||

Sbjct 109139 AGGTTATGTTCTCTTGAGTAGTAGATGGAACGGTATCTTTTGTAGCAGGAATGCTATCTC 109198

Query 340594 TCGTAGTTGGTTTTTGAGAGAAGTTAGATGATTCATCATTTTATTTAAAGTGGTCATCAT 340653

||||||||||||||||||||||||||||||||||||||||||||||||||||||||||||

Sbjct 109199 TCGTAGTTGGTTTTTGAGAGAAGTTAGATGATTCATCATTTTATTTAAAGTGGTCATCAT 109258

Query 340654 GTTGTAGATTCATATTAACTTGTTCTTTCTGTTGTGTGTTGTTTACTTGACCCCTTCCTT 340713

||||||||||||||||||||||||||||||||||||||||||||||||||||||||||||

Sbjct 109259 GTTGTAGATTCATATTAACTTGTTCTTTCTGTTGTGTGTTGTTTACTTGACCCCTTCCTT 109318

Query 340714 CAGCCTTTCTCCCTTTCTTCTTTCTGAGGGCTATTTTGCAAGTACATACTCAATGCTCtt 340773

|||||||||||||||||||||||||| |||||||||||||||||||||||||||||||||

Sbjct 109319 CAGCCTTTCTCCCTTTCTTCTTTCTGGGGGCTATTTTGCAAGTACATACTCAATGCTCTT 109378

Query 340774 ttgctttttcttttttCTTATTCTTAGTAGGCAAGAGCCAGTTGCCGAGGGCTCCTTAAG 340833

||||||||||||||||||||||||||||||||||||||||||||||||||||||||||||

Sbjct 109379 TTGCTTTTTCTTTTTTCTTATTCTTAGTAGGCAAGAGCCAGTTGCCGAGGGCTCCTTAAG 109438

Query 340834 AAGAAGGAAAAGAGGGGGAAGGCGGATGGAATAAGAGGGGTAGTGGTTTTTATTCCGAAA 340893

||||||||||||||||||||||||||||||||||||||||||||||||||||||||||||

Sbjct 109439 AAGAAGGAAAAGAGGGGGAAGGCGGATGGAATAAGAGGGGTAGTGGTTTTTATTCCGAAA 109498

Query 340894 TGCCCTTTTATGACaaaaaaaCAAGGGGAATCCTACGTAGAACTACTTGAACTGCTGCAG 340953

||||||||||||||||||||||||||||||||||||||||||||||||||||||||||||

Sbjct 109499 TGCCCTTTTATGACAAAAAAACAAGGGGAATCCTACGTAGAACTACTTGAACTGCTGCAG 109558

Query 340954 TCAAAGCATAAGCAATCCAATCTGCAGCATAGCCCGCCCTTCTTAGGTGCCCTCTTTCAC 341013

||||||||||||||||||||||||||||||||||||||||||||||||||||||||||||

Sbjct 109559 TCAAAGCATAAGCAATCCAATCTGCAGCATAGCCCGCCCTTCTTAGGTGCCCTCTTTCAC 109618

Query 341014 TCAAGCTTGAAGCAAGAAAACTACTGATGTGGATGAGCTCAAATAGCTAATATTATATGA 341073

||||||||||||||||||||||||||||||||||||||||||||||||||||||||||||

Sbjct 109619 TCAAGCTTGAAGCAAGAAAACTACTGATGTGGATGAGCTCAAATAGCTAATATTATATGA 109678

Query 341074 TCTACCCAGGCTACATTGGCTTTGAGTGTGAAGGAAAGCCACTAGGCCACGTATGTTAGT 341133

||||||||||||||||||||||||||||||||||||||||||||||||||||||||||||

Sbjct 109679 TCTACCCAGGCTACATTGGCTTTGAGTGTGAAGGAAAGCCACTAGGCCACGTATGTTAGT 109738

Query 341134 GATTTGAGAAAGCGTAAGCCGCCCATAGGGATTGAAGTCGATAGAGCCCTGGAAGGAAGA 341193

||||||||||||||||||||| ||||||||||||||||||||||||||||||||||||||

Sbjct 109739 GATTTGAGAAAGCGTAAGCCGTCCATAGGGATTGAAGTCGATAGAGCCCTGGAAGGAAGA 109798

Query 341194 ATGAGGCAGAACCATAAACCGTGAAATGTGACTAAGAAATGCTTTGGACCCTTCCTCTTT 341253

||||||||||||||||||||||||||||||||||||||||||||||||||||||||||||

Sbjct 109799 ATGAGGCAGAACCATAAACCGTGAAATGTGACTAAGAAATGCTTTGGACCCTTCCTCTTT 109858

Query 341254 CCAAAATAGGCATCTTTGGGCCCTTCCTGCTTCAATAAGTATTCGGGGAATCCTTCTTTC 341313

||||||||||||||||||||||||||||||||||||||||||||||||||||||||||||

Sbjct 109859 CCAAAATAGGCATCTTTGGGCCCTTCCTGCTTCAATAAGTATTCGGGGAATCCTTCTTTC 109918

Query 341314 TCCGCTGCCTGCCCCTATACTAGCTAGCTATACCGGGTGGATACTTGACTCAACCTACTG 341373

|| |||||||||||||||||||||||||||||||||||||||||||||||||||||||||

Sbjct 109919 TCTGCTGCCTGCCCCTATACTAGCTAGCTATACCGGGTGGATACTTGACTCAACCTACTG 109978

Query 341374 GTGCATTGATGAATGGCTAGAAAGATGTTCAGTGACCCAGGAGAG----TTTTTTAAGCT 341429

||||||||||||||||||||||||||||||||||||||||||||| |||||||||||

Sbjct 109979 GTGCATTGATGAATGGCTAGAAAGATGTTCAGTGACCCAGGAGAGTTTTTTTTTTAAGCT 110038

Query 341430 GGTATTAGAGCCCCTTCTTTTTCCTTTATGTACTTATTGTAAGAATGCCTCCCGCTCTTC 341489

|||||||||||||||||||||||||||||||||||||||| |||||||||||||||||||

Sbjct 110039 GGTATTAGAGCCCCTTCTTTTTCCTTTATGTACTTATTGTCAGAATGCCTCCCGCTCTTC 110098

Query 341490 CCGGCGATAAAGAAGGCCCAGGATTGGAATGAGCAATGCCAATAAGCCTTTGAGAAAATC 341549

||||||||||||||||||||||||||||||||||||||||||||||||||||||||||||

Sbjct 110099 CCGGCGATAAAGAAGGCCCAGGATTGGAATGAGCAATGCCAATAAGCCTTTGAGAAAATC 110158

Query 341550 CAAGAGTACCTCACCTATCATGAGCAGGAAAGCCCCCAACTGGATTCTCTATTTCACCAT 341609

||||||||||||||||||||||||||||||||||||||||||||||||||||||||||||

Sbjct 110159 CAAGAGTACCTCACCTATCATGAGCAGGAAAGCCCCCAACTGGATTCTCTATTTCACCAT 110218

Query 341610 TTAAGCAAGCGAGAAGGCAGTCAGCTAAGGGTAAGAGGCAAGGGCAAAAGAGACCTGTGT 341669

||||||||||||||||||||||||||||||||||||||||||||||||||||||||||||

Sbjct 110219 TTAAGCAAGCGAGAAGGCAGTCAGCTAAGGGTAAGAGGCAAGGGCAAAAGAGACCTGTGT 110278

Query 341670 ATCTTGTGTCTAAGGTGCTCCGAGATGCGTAGACTAGATACCCGCCCTTATAAAAAGCAG 341729

|||||||||||||||||||||||||||||||||||| |||||||||||||||||||||||

Sbjct 110279 ATCTTGTGTCTAAGGTGCTCCGAGATGCGTAGACTATATACCCGCCCTTATAAAAAGCAG 110338

Query 341730 TATTCAAAGTGGTAAATTGCGAGCATACCAAATTGCGACCATACTTTCAAGCCCAATCTC 341789

||||||||||||||||||||||||||||||||||||||||||||||||||||||||||||

Sbjct 110339 TATTCAAAGTGGTAAATTGCGAGCATACCAAATTGCGACCATACTTTCAAGCCCAATCTC 110398

Query 341790 AGTGATGACTCACCTACCGTGGAAAAAGACTTTGGAGCACCTAGATGTCTACGTCTGTCG 341849

||||||||||||||||||||||||||||||||||||||||||||||||||||||||||||

Sbjct 110399 AGTGATGACTCACCTACCGTGGAAAAAGACTTTGGAGCACCTAGATGTCTACGTCTGTCG 110458

Query 341850 AACTTTTCCAATAAAATGTTCCCTTTCATCCACGGAACGCCCAGGTCCTTACAGGCTTCG 341909

||||||||||||||||||||||||||||||||||||||||||||||||||||||||||||

Sbjct 110459 AACTTTTCCAATAAAATGTTCCCTTTCATCCACGGAACGCCCAGGTCCTTACAGGCTTCG 110518

Query 341910 TTGTAGAATGTATGAAGTTTAGGACCAGACTGGTTGATATATGTGGACGGGGCTAGGGGG 341969

||||||||||||||||||||||||||||||||||||||||||||||||||||||||||||

Sbjct 110519 TTGTAGAATGTATGAAGTTTAGGACCAGACTGGTTGATATATGTGGACGGGGCTAGGGGG 110578

Query 341970 GTCCCGGAGCAGGGGTTACCATGGTAGGTACGAAAGGAGAGGTGTTGATTTATGCGACCT 342029

||||||||||||||||||||||||||||||||||||||||||||||||||||||||||||

Sbjct 110579 GTCCCGGAGCAGGGGTTACCATGGTAGGTACGAAAGGAGAGGTGTTGATTTATGCGACCT 110638

Query 342030 TCCGAACATCGAAGAACGCAGCAGAGAGGTACTTTGATTTTTATCATACCTTTGTCTACT 342089

||||||||||||||||||||||||||||||||||||||||||||||||||||||||||||

Sbjct 110639 TCCGAACATCGAAGAACGCAGCAGAGAGGTACTTTGATTTTTATCATACCTTTGTCTACT 110698

Query 342090 CAAATTGACAAGTGTAGAACTTGTACTATGTGCTATCATTAGTACCATTTATTGCTCCAA 342149

||||||||||||||||||||||||||||||||||||||||||||||||||||||||||||

Sbjct 110699 CAAATTGACAAGTGTAGAACTTGTACTATGTGCTATCATTAGTACCATTTATTGCTCCAA 110758

Query 342150 GACCCATTTCTGATTGAATTCAGactttttatggcttttttgacttgtttttcttttagg 342209

||||||||||||||||||||||||||||||||||||||||||||||||||||||||||||

Sbjct 110759 GACCCATTTCTGATTGAATTCAGACTTTTTATGGCTTTTTTGACTTGTTTTTCTTTTAGG 110818

Query 342210 gtactttgcctagggttactccttttttaTTGGAAAAATTACTTGGTGGTGTACATGCAG 342269

||||||||||||||||||||||||||||||||||||||||||||||||||||||||||||

Sbjct 110819 GTACTTTGCCTAGGGTTACTCCTTTTTTATTGGAAAAATTACTTGGTGGTGTACATGCAG 110878

Query 342270 ATATGCCAGCAATATGAGTTTGATACAACCAAGAAGAGATGGTCGACGTCGAGCTAGTGG 342329

||||||||||||||||||||||||||||||||||||||||||||||||||||||||||||

Sbjct 110879 ATATGCCAGCAATATGAGTTTGATACAACCAAGAAGAGATGGTCGACGTCGAGCTAGTGG 110938

Query 342330 GATAGGTTCATACTTAATCTTCTAGGACGAGTTCAAATGGCACGAACGAACGGGTGAGTC 342389

||||||||||||||||||||||||||||||||||||||||||||||||||||||||||||

Sbjct 110939 GATAGGTTCATACTTAATCTTCTAGGACGAGTTCAAATGGCACGAACGAACGGGTGAGTC 110998

Query 342390 AAGCACGAACCCTCAGATCCTGCCATCCGTTCACTAATTTTATTGCTCTACCTCTGATTG 342449

||||||||||||||||||||||||||||||||||||||||||||||||||||||||||||

Sbjct 110999 AAGCACGAACCCTCAGATCCTGCCATCCGTTCACTAATTTTATTGCTCTACCTCTGATTG 111058

Query 342450 CTCAGAGAAATGAAAACTTTATAAAGGTAGGTTTTGCAACTTTGCCACAGCTCTGCGTTT 342509

||||||||||||||||||||||||||||||||||||||||||||||||||||||||||||

Sbjct 111059 CTCAGAGAAATGAAAACTTTATAAAGGTAGGTTTTGCAACTTTGCCACAGCTCTGCGTTT 111118

Query 342510 CCTATGATGCCCAACAACGTTACAGCTGTATTCTATTTTGGAAAGGGGAATTCTTTTTCT 342569

||||||||||||||||||||||||||||||||||||||||||||||||||||||||||||

Sbjct 111119 CCTATGATGCCCAACAACGTTACAGCTGTATTCTATTTTGGAAAGGGGAATTCTTTTTCT 111178

Query 342570 TTATACAGCGTCATTTGTGCATAAGTACCTTCTATATTACCCCAAGCACACCAAGGGGAA 342629

||||||||||||||||||||||||||||||||||||||||||||||||||||||||||||

Sbjct 111179 TTATACAGCGTCATTTGTGCATAAGTACCTTCTATATTACCCCAAGCACACCAAGGGGAA 111238

Query 342630 CCTTAGCCACATCTAGATCTGGGTCGCCCACCTAACGAAGTTCAACCCATAAAGTAAACT 342689

||||||||||||||||||||||||||||||||||||||||||||||||||||||||||||

Sbjct 111239 CCTTAGCCACATCTAGATCTGGGTCGCCCACCTAACGAAGTTCAACCCATAAAGTAAACT 111298

Query 342690 TTCAGTAAGCATGATAGTTGTTGACTCATAAGCACTTATTCAAATTCTTTTTCATACATA 342749

||||||||||||||||||||||||||||||||||||||||||||||||||||||||||||

Sbjct 111299 TTCAGTAAGCATGATAGTTGTTGACTCATAAGCACTTATTCAAATTCTTTTTCATACATA 111358

Query 342750 AAGTATTTGATCTTTCTTTCGTTCTAGAAATAGCATAAGAGAATTTCTTATTATCCCTTC 342809

||||||||||||||||||||||||||||||||||||||||||||||||||||||||||||

Sbjct 111359 AAGTATTTGATCTTTCTTTCGTTCTAGAAATAGCATAAGAGAATTTCTTATTATCCCTTC 111418

Query 342810 AATGCTATATTAGGGATACCTTCTCTTAACAAGCTGGGGGCTTTCATATCAACCGCACAT 342869

||||||||||||||||||||||||||||||||||||||||||||||||||||||||||||

Sbjct 111419 AATGCTATATTAGGGATACCTTCTCTTAACAAGCTGGGGGCTTTCATATCAACCGCACAT 111478

Query 342870 CTAGCCATGAAGCTTCCGAGCCCCGGAGGAAAAGTGTGAACAATCTGGGTAGAGAAATAC 342929

||||||||||||||||||||||||||||||||||||||||||||||||||||||||||||

Sbjct 111479 CTAGCCATGAAGCTTCCGAGCCCCGGAGGAAAAGTGTGAACAATCTGGGTAGAGAAATAC 111538

Query 342930 GGAGCTAGGCACTGCTACACGACCTGTCTGAGGCAGACAAGTAATCCGGGTGGTTGATGT 342989

||||||||||||||||||||||||||||||||||||||||||||||||||||||||||||

Sbjct 111539 GGAGCTAGGCACTGCTACACGACCTGTCTGAGGCAGACAAGTAATCCGGGTGGTTGATGT 111598

Query 342990 TCCTATTGAGCCCGCAAGCGAGCACACGCCTTTCATCAGAGGTGCTCCTCTCGAAACCAA 343049

||||||||||||||||||||||||||||||||||||||||||||||||||||||||||||

Sbjct 111599 TCCTATTGAGCCCGCAAGCGAGCACACGCCTTTCATCAGAGGTGCTCCTCTCGAAACCAA 111658

Query 343050 AACGACCCAGCTGATAGAAGGAGGCCCATCCAAGATAACCAAGATTGGCGGCAGTCTGAC 343109

||||||||||||||||||||||||||||||||||||||||||||||||||||||||||||

Sbjct 111659 AACGACCCAGCTGATAGAAGGAGGCCCATCCAAGATAACCAAGATTGGCGGCAGTCTGAC 111718

Query 343110 AGAGCAAGAAGAGCTCGATCTGATAGCGGCGCTTGAAGGGCAATGTGGACATCTTTGCAT 343169

||||||||||||||||||||||||||||||||||||||||||||||||||||||||||||

Sbjct 111719 AGAGCAAGAAGAGCTCGATCTGATAGCGGCGCTTGAAGGGCAATGTGGACATCTTTGCAT 111778

Query 343170 GGAGTGCGAGCGATATGCCTGGCATTGATCCCAAGGTGATGAAACACAAGCTAGCAACAG 343229

||||||||||||||||||||||||||||||||||||||||||||||||||||||||||||

Sbjct 111779 GGAGTGCGAGCGATATGCCTGGCATTGATCCCAAGGTGATGAAACACAAGCTAGCAACAG 111838

Query 343230 ACCCTTCTGTCAGACCAGTCCAACAAAGACGGAGGAAGTTCGCTCCTGATAGATTGGATG 343289

||||||||||||||||||||||||||||||||||||||||||||||||||||||||||||

Sbjct 111839 ACCCTTCTGTCAGACCAGTCCAACAAAGACGGAGGAAGTTCGCTCCTGATAGATTGGATG 111898

Query 343290 CTATCAAAGAAGAGATGAAAAGGAATCCAGAAAAGTGTGTGTTCGGAGCAGTTTATGGAA 343349

||||||||||||||||||||||||||||||||||||||||||||||||||||||||||||

Sbjct 111899 CTATCAAAGAAGAGATGAAAAGGAATCCAGAAAAGTGTGTGTTCGGAGCAGTTTATGGAA 111958

Query 343350 AGTTTCCAGAAAGGGAGCTTCGAATTTATCCAATGGAAATCATGGATCGAAGGTGGGTCA 343409

||||||||||||||||||||||||||||||||||||||||||||||||||||||||||||

Sbjct 111959 AGTTTCCAGAAAGGGAGCTTCGAATTTATCCAATGGAAATCATGGATCGAAGGTGGGTCA 112018

Query 343410 AGAGTTTTCTTGAAAGAAGGCCTTCATCACTCTCGGGTTCTTAAGCTGTAGCTAAATAGC 343469

||||||||||||||||||||||||||||||||||||||||||||||||||||||||||||

Sbjct 112019 AGAGTTTTCTTGAAAGAAGGCCTTCATCACTCTCGGGTTCTTAAGCTGTAGCTAAATAGC 112078

Query 343470 TGCTTCACAAAGAGGCCGGCCCCAACGAACTACTTACTTCTTTTTCAATCAAAGGTCCGT 343529

||||||||||||||||||||||||||||||||||||||||||||||||||||||||||||

Sbjct 112079 TGCTTCACAAAGAGGCCGGCCCCAACGAACTACTTACTTCTTTTTCAATCAAAGGTCCGT 112138

Query 343530 CCAATGAGAATGAGTAGTGGCTTACATGCAAGTGAGAAGAGTAAAGATCGATCAGCTGAC 343589

||||||||||||||||||||||||||||||||||||||||||||||||||||||||||||

Sbjct 112139 CCAATGAGAATGAGTAGTGGCTTACATGCAAGTGAGAAGAGTAAAGATCGATCAGCTGAC 112198

Query 343590 TGGACATGAATTATTAAGGCGGACACTCCCGTTTGTAGACGCCTTCATGCCAGGGCTCTT 343649

||||||||||||||||||||||||||||||||||||||||||||||||||||||||||||

Sbjct 112199 TGGACATGAATTATTAAGGCGGACACTCCCGTTTGTAGACGCCTTCATGCCAGGGCTCTT 112258

Query 343650 CCTGCTGAGTGAGGATGCGCTTCCCCTGATCTGTAAGGTCTTTCCCATTCGTTCAGCCCT 343709

||||||||||||||||||||||||||||||||||||||||||||||||||||||||||||

Sbjct 112259 CCTGCTGAGTGAGGATGCGCTTCCCCTGATCTGTAAGGTCTTTCCCATTCGTTCAGCCCT 112318

Query 343710 ATTAATTCTAGGAAAATCTATGGAGTGAAATATCAAGTATGCCGGCCCGCCCTAGAAAGA 343769

||||||||||||||||||||||||||||||||||||||||||||||||||||||||||||

Sbjct 112319 ATTAATTCTAGGAAAATCTATGGAGTGAAATATCAAGTATGCCGGCCCGCCCTAGAAAGA 112378

Query 343770 TCATTGCATTTCTTTCTTCAGGCGCAAGCAGGCCGGTGTGTATGCATCCTACATGTTGTG 343829

||||||||||||||||||||||||||||||||||||||||||||||||||||||||||||

Sbjct 112379 TCATTGCATTTCTTTCTTCAGGCGCAAGCAGGCCGGTGTGTATGCATCCTACATGTTGTG 112438

Query 343830 GCCCCCAGGAAAGCGGCTCAAGGTACACCTAACTATGCGCATCTTTCTTTCTCACCCAAA 343889

||||||||||||||||||||||||||||||||||||||||||||||||||||||||||||

Sbjct 112439 GCCCCCAGGAAAGCGGCTCAAGGTACACCTAACTATGCGCATCTTTCTTTCTCACCCAAA 112498

Query 343890 ACCTAAACTATTAATTAGGAGAAATTCCTGTGAGCTTAGCATTAGTGAAACCATATATGG 343949

||||||||||||||||||||||||||||||||||||||||||||||||||||||||||||

Sbjct 112499 ACCTAAACTATTAATTAGGAGAAATTCCTGTGAGCTTAGCATTAGTGAAACCATATATGG 112558

Query 343950 TACTTCTATCGGGCACGAGATATCACCGCCGCCGCTTTTCAGTGACGGGACTATACTGGG 344009

||||||||||||||||||||||||||||||||||||||||||||||||||||||||||||

Sbjct 112559 TACTTCTATCGGGCACGAGATATCACCGCCGCCGCTTTTCAGTGACGGGACTATACTGGG 112618

Query 344010 GGAGACTCAATGGAGCTGGCTGGAGAATGAATTGAATGGACCTCAATTCGGGATCACCTT 344069

|||||||||||||||||||||||||||||||||||||||||||||||||| |||||||||

Sbjct 112619 GGAGACTCAATGGAGCTGGCTGGAGAATGAATTGAATGGACCTCAATTCGAGATCACCTT 112678

Query 344070 CATTGCTTCTTCTATTCGGAAACACACCGCCCTTTTTGGCTGGCAAGATTTTATTTCTCA 344129

||||||||||||||||||||||||||||||||||||||||||||||||||||||||||||

Sbjct 112679 CATTGCTTCTTCTATTCGGAAACACACCGCCCTTTTTGGCTGGCAAGATTTTATTTCTCA 112738

Query 344130 TAGCAAGATTCGAGAGTATAGGGCGTACGTAGCCCATTGTAGATCCAGCCAAGAAGAATC 344189

||||||||||||||||||||||||||||||||||||||||||||||||||||||||||||

Sbjct 112739 TAGCAAGATTCGAGAGTATAGGGCGTACGTAGCCCATTGTAGATCCAGCCAAGAAGAATC 112798

Query 344190 AACAGGCAAAGACGACCATGTTACTATCGCGTCTGTGTCTTATCATTTTGAAAAAGTGGG 344249

||||||||||||||||||||||||||||||||||||||||||||||||||||||||||||

Sbjct 112799 AACAGGCAAAGACGACCATGTTACTATCGCGTCTGTGTCTTATCATTTTGAAAAAGTGGG 112858

Query 344250 AATCCAACCCGGCTTCCTACCCTCGGGGAAGAAATCTTTGGAATACTAAGAGCCCTCCTC 344309

||||||||||||||||||||||||||||||||||||||||||||||||||||||||||||

Sbjct 112859 AATCCAACCCGGCTTCCTACCCTCGGGGAAGAAATCTTTGGAATACTAAGAGCCCTCCTC 112918

Query 344310 AAGGGTGTCTATAGGTTTTGGCCTATTCTCGCCTACAGGTTGCTGACGGATAATGAGAAT 344369

||||||||||||||||||||||||||||||||||||||||||||||||||||||||||||

Sbjct 112919 AAGGGTGTCTATAGGTTTTGGCCTATTCTCGCCTACAGGTTGCTGACGGATAATGAGAAT 112978

Query 344370 GCCTACAAATATGCGATGTGAAGAATTGAGATGACAATACTTTGCATATATAATATGCAT 344429

||||||||||||||||||||||||||||||||||||||||||||||||||||||||||||

Sbjct 112979 GCCTACAAATATGCGATGTGAAGAATTGAGATGACAATACTTTGCATATATAATATGCAT 113038

Query 344430 TTCCTATGTTGTAATTAGCACTTGTGTTATGTTTTGTACCACTGTGTATCTAGTAGTATA 344489

||||||||||||||||||||||||||||||||||||||||||||||||||||||||||||

Sbjct 113039 TTCCTATGTTGTAATTAGCACTTGTGTTATGTTTTGTACCACTGTGTATCTAGTAGTATA 113098

Query 344490 CTTACTTGGCTCACTAACCAACTACACAGGATCAAAAAAGCCCGACCTGGCGGCCTTTGT 344549

||||||||||||||||||||||||||||||||||||||||||||||||||||||||||||

Sbjct 113099 CTTACTTGGCTCACTAACCAACTACACAGGATCAAAAAAGCCCGACCTGGCGGCCTTTGT 113158

Query 344550 CAGCAGCAAATCAAGTTCCTACCGCGCCGCGATTTCCACTTAAAGTCAGGTCACAAAGAA 344609

||||||||||||||||||||||||||||||||||||||||||||||||||||||||||||

Sbjct 113159 CAGCAGCAAATCAAGTTCCTACCGCGCCGCGATTTCCACTTAAAGTCAGGTCACAAAGAA 113218

Query 344610 CTAGCTATGAAAAGCTATTTTGAATCATTGTCTTATTCATTGGAACATGGCAATCTATCT 344669

||||||||||||||||||||||||||||||||||||||||||||||||||||||||||||

Sbjct 113219 CTAGCTATGAAAAGCTATTTTGAATCATTGTCTTATTCATTGGAACATGGCAATCTATCT 113278

Query 344670 TACTTGCCCATACATACTAGTGCTTTAATATTCTGCTATATCAAGATCCAGGGAAAGTAT 344729

||||||||||||||||||||||||||||||||||||||||||||||||||||||||||||

Sbjct 113279 TACTTGCCCATACATACTAGTGCTTTAATATTCTGCTATATCAAGATCCAGGGAAAGTAT 113338

Query 344730 ACTTTTGAGAAAAGAGGCTCGCCGGTAGAAGATAAAGAATCACtttttttAGCTCCGCAT 344789

||||||||||||||||||||||||||||||||||||||||||||||||||||||||||||

Sbjct 113339 ACTTTTGAGAAAAGAGGCTCGCCGGTAGAAGATAAAGAATCACTTTTTTTAGCTCCGCAT 113398

Query 344790 ----TAACTACTTATTGACTGCATGCATGGAGTTCCGTCGCATTGTTGTGAGTGCTGCCG 344845

||||||||||||||||||||||||||||||||||||||||||||||||||||||||

Sbjct 113399 ATACTAACTACTTATTGACTGCATGCATGGAGTTCCGTCGCATTGTTGTGAGTGCTGCCG 113458

Query 344846 GAGGAGAAAGACCACCTGGTACGTACACCAGTTCGGGTTGTGGATCAGTAACAGCAGCTC 344905

||||||||||||||||||||||||||||||||||||||||||||||||||||||||||||

Sbjct 113459 GAGGAGAAAGACCACCTGGTACGTACACCAGTTCGGGTTGTGGATCAGTAACAGCAGCTC 113518

Query 344906 CCGTCTTCTCTGTCACAACGTTGTTTTTTATTGCAATTGTTTTTAATAAATATAAGATAA 344965

||||||||||||||||||||||||||||||||||||||||||||||||||||||||||||

Sbjct 113519 CCGTCTTCTCTGTCACAACGTTGTTTTTTATTGCAATTGTTTTTAATAAATATAAGATAA 113578

Query 344966 AGAAAGAGCTTTATTTATAGTTCAGTATTCTCTTTTAAGTATGTTCCAGACAGTAAAGTT 345025

|||||||||||||||||||||| |||||||||||||||||||||||||||||||||||||

Sbjct 113579 AGAAAGAGCTTTATTTATAGTTAAGTATTCTCTTTTAAGTATGTTCCAGACAGTAAAGTT 113638

Query 345026 CTTTTTAAGTGTATTGATTTAAGCATTTGCACTTTCATTTAGCATTGATTGAATTCTGGT 345085

||||||||||||||||||||||||||||||||||||||||||||||||||||||||||||

Sbjct 113639 CTTTTTAAGTGTATTGATTTAAGCATTTGCACTTTCATTTAGCATTGATTGAATTCTGGT 113698

Query 345086 AGGGATGGTAAAGCAAACTGAAATAGGGCGAGCGAGGCGCGAAGCGTATAGGTTTCTTGT 345145

||||||||||||||||||||||||||||||||||||||||||||||||||||||||||||

Sbjct 113699 AGGGATGGTAAAGCAAACTGAAATAGGGCGAGCGAGGCGCGAAGCGTATAGGTTTCTTGT 113758

Query 345146 AATTTTAGTTACTTGCCAGTAAGTAAATAAGCtttttttACTAGTCATCAAAGAGTTCCA 345205

||||||||||||||||||||||||||||||||||||||||||||||||||||||||||||

Sbjct 113759 AATTTTAGTTACTTGCCAGTAAGTAAATAAGCTTTTTTTACTAGTCATCAAAGAGTTCCA 113818

Query 345206 CATAAAAGTTATTAAGGAACAGTATAGGCTTAAATAAAGTAGTTTGAACCACTTGACCGT 345265

||||||||||||||||||||||||||||||||||||||||||||||||||||||||||||

Sbjct 113819 CATAAAAGTTATTAAGGAACAGTATAGGCTTAAATAAAGTAGTTTGAACCACTTGACCGT 113878

Query 345266 CTTGATATGAAGGGGATCTCTTTGATTGATGCCATTAAGTTCTTATTCTGGTGCGCAAGT 345325

||||||||||||||||||||||||||||||||||||||||||||||||||||||||||||

Sbjct 113879 CTTGATATGAAGGGGATCTCTTTGATTGATGCCATTAAGTTCTTATTCTGGTGCGCAAGT 113938

Query 345326 TTTGGTTTCATGGGTATATATATCTTCCTCTAAGTAATTATTGTGTGTTCTTGGTAGCGT 345385

||||||||||||||||||||||||||||||||||||||||||||||||||||||||||||

Sbjct 113939 TTTGGTTTCATGGGTATATATATCTTCCTCTAAGTAATTATTGTGTGTTCTTGGTAGCGT 113998

Query 345386 TGGAAAGTAAGTAGACTCACCAAGCTTTCTaaaaaaaaGTATAATGCTTTGGGTGGGGGT 345445

||||||||||||||||||||||||||||||||||||||||||||||||||||||||||||

Sbjct 113999 TGGAAAGTAAGTAGACTCACCAAGCTTTCTAAAAAAAAGTATAATGCTTTGGGTGGGGGT 114058

Query 345446 GTTATGGAAAGAGACAGTGATAAAACGGCCTTAGAAAAAGAGATTACTTTCAACAGAAGA 345505

||||||||||||||||||||||||||||||||||||||||||||||||||||||||||||

Sbjct 114059 GTTATGGAAAGAGACAGTGATAAAACGGCCTTAGAAAAAGAGATTACTTTCAACAGAAGA 114118

Query 345506 ATTTGGGCACGAGGGTAGAGAACACCAAAAAAGGTAAACAACAACGAATTGATATAAACA 345565

||||||||||||||||||||||||||||||||||||||||||||||||||||||||||||

Sbjct 114119 ATTTGGGCACGAGGGTAGAGAACACCAAAAAAGGTAAACAACAACGAATTGATATAAACA 114178

Query 345566 AAACCGTGAAAAGAATAAATAGAAGTCTATTTCATTCATTTACCTTGGCCATAAATACTC 345625

||||||||||||||||||||||||||||||||||||||||||||||||||||||||||||

Sbjct 114179 AAACCGTGAAAAGAATAAATAGAAGTCTATTTCATTCATTTACCTTGGCCATAAATACTC 114238

Query 345626 TTTCTTAGTGGTTCACCAAAAGTTAAATAAATGGGAGAGTAAGGTTATTAGACATAAAAT 345685

||||||||||||||||||||||||||||||||||||||||||||||||||||||||||||

Sbjct 114239 TTTCTTAGTGGTTCACCAAAAGTTAAATAAATGGGAGAGTAAGGTTATTAGACATAAAAT 114298

Query 345686 ATATTTCTTAGAAATCCCTATAATCTTGAACGATTTCACTACTAGACTAGCTATGATTTT 345745

||||||||||||||||||||||||||||||||||||||||||||||||||||||||||||

Sbjct 114299 ATATTTCTTAGAAATCCCTATAATCTTGAACGATTTCACTACTAGACTAGCTATGATTTT 114358

Query 345746 TCTAATGAAATTTCACTTAATTCAATGTGTTAATTGCTATAACTGATACTAGAAAGAGAA 345805

||||||||||||||||||||||||||||||||||||||||||||||||||||||||||||

Sbjct 114359 TCTAATGAAATTTCACTTAATTCAATGTGTTAATTGCTATAACTGATACTAGAAAGAGAA 114418

Query 345806 TTGCAACACCGGGACCTTGACTGGTTCTTGGAGGAGATGGTCCAGCCACTTCATTAGCTG 345865

||||||||||||||||||||||||||||||||||||||||||||||||||||||||||||

Sbjct 114419 TTGCAACACCGGGACCTTGACTGGTTCTTGGAGGAGATGGTCCAGCCACTTCATTAGCTG 114478

Query 345866 TACAAAATTAATAAAGCACAAAAAACCATTTATTGATAAATAAATCAGCGATGCTTTGTT 345925

||||||||||||||||||||||||||||||||||||||||||||||||||||||||||||

Sbjct 114479 TACAAAATTAATAAAGCACAAAAAACCATTTATTGATAAATAAATCAGCGATGCTTTGTT 114538

Query 345926 TTAATATGAAATAGAGTTGTGGTACCCTTAGCTTTAGTTTTCTTCCTAGGAGATGGTCGA 345985

||||||||||||||||||||||||||||||||||||||||||||||||||||||||||||

Sbjct 114539 TTAATATGAAATAGAGTTGTGGTACCCTTAGCTTTAGTTTTCTTCCTAGGAGATGGTCGA 114598

Query 345986 GGGATGTCAGGACCAGTCTAGTCTTGCTCCTTTGTACAAGGGGAGTAACAACAATAATTA 346045

||||||||||||||||||||||||||||||||||||||||||||||||||||||||||||

Sbjct 114599 GGGATGTCAGGACCAGTCTAGTCTTGCTCCTTTGTACAAGGGGAGTAACAACAATAATTA 114658

Query 346046 GAAGCAGTGTTTTAATGCAGAAGATAGAGCAAAAATCCCATTAAACTGAAAAAACTGGTA 346105

||||||||||||||||||||||||||||||||||||||||||||||||||||||||||||

Sbjct 114659 GAAGCAGTGTTTTAATGCAGAAGATAGAGCAAAAATCCCATTAAACTGAAAAAACTGGTA 114718

Query 346106 TAATGCCACTTTACTTACCCTATTCCCCCCTTTTCAAACGGAGACACACATTGAACAGCT 346165

||||||||||||||||||||||||||||||||||||||||||||||||||||||||||||

Sbjct 114719 TAATGCCACTTTACTTACCCTATTCCCCCCTTTTCAAACGGAGACACACATTGAACAGCT 114778

Query 346166 GTTGAATGAATCTTTGGAGATCGGTAGGCAGCCAACGTAGACTTGTGCCCAAGTATCTCC 346225

||||||||||||||||||||||||||||||||||||||||||||||||||||||||||||

Sbjct 114779 GTTGAATGAATCTTTGGAGATCGGTAGGCAGCCAACGTAGACTTGTGCCCAAGTATCTCC 114838

Query 346226 CTCATAGCTCTGGCTGATCTCTTTCATCCATGTTGGAACTAGCTCAGTAACGGCCATCAA 346285

||||||||||||||||||||||||||||||||||||||||||||||||||||||||||||

Sbjct 114839 CTCATAGCTCTGGCTGATCTCTTTCATCCATGTTGGAACTAGCTCAGTAACGGCCATCAA 114898

Query 346286 CTTTCCTCTTTTCTCCTCATTCCTTCTAGACAAGGCATCGTCAATCCACCTGATATATAT 346345

||||||||||||||||||||||||||||||||||||||||||||||||||||||||||||

Sbjct 114899 CTTTCCTCTTTTCTCCTCATTCCTTCTAGACAAGGCATCGTCAATCCACCTGATATATAT 114958

Query 346346 AAAAGAGATTGTTCGCTTACGCTTTCTCCTTCATTCGTAAAATACGCTTGTCAATTCTTG 346405

||||||||||||||||||||||||||||||||||||||||||||||||||||||||||||

Sbjct 114959 AAAAGAGATTGTTCGCTTACGCTTTCTCCTTCATTCGTAAAATACGCTTGTCAATTCTTG 115018

Query 346406 GTGTTTTTTCTCACTCGTCAATTTTTTGTGATTTCATTTTGAACTTCTCAGGTAGGTGTG 346465

||||||||||||||||||||||||||||||||||||||||||||||||||||||||||||

Sbjct 115019 GTGTTTTTTCTCACTCGTCAATTTTTTGTGATTTCATTTTGAACTTCTCAGGTAGGTGTG 115078

Query 346466 ATCAGTCTCATTAATCCGTATAAGAATTTGGAATTCCTCTTCCAACTCGTCCAAGAATTG 346525

||||||||||||||||||||||||||||||||||||||||||||||||||||||||||||

Sbjct 115079 ATCAGTCTCATTAATCCGTATAAGAATTTGGAATTCCTCTTCCAACTCGTCCAAGAATTG 115138

Query 346526 GCGTTATGGCAAAGAACAAGAAGAAAAATACAGAAGAAATTTGTCCAATAGTAACAAAAG 346585

||||||||||||||||||||||||||||||||||||||||||||||||||||||||||||

Sbjct 115139 GCGTTATGGCAAAGAACAAGAAGAAAAATACAGAAGAAATTTGTCCAATAGTAACAAAAG 115198

Query 346586 GTGCCTCTACAGGTTGACATCCGATCCAACCTAGTAGTAAGCAATCTGCCAAAAGCAACC 346645

||||||||||||||||||||||||||||||||||||||||||||||||||||||||||||

Sbjct 115199 GTGCCTCTACAGGTTGACATCCGATCCAACCTAGTAGTAAGCAATCTGCCAAAAGCAACC 115258

Query 346646 AAAATATTCCTTGGTAAATGGGTCGAAAACTTGAACTACGCACATACATATTTTTaaaaa 346705

||||||||||||||||||||||||||||||||||||||||||||||||||||||||||||

Sbjct 115259 AAAATATTCCTTGGTAAATGGGTCGAAAACTTGAACTACGCACATACATATTTTTAAAAA 115318

Query 346706 aaGGTAAAGCCAAGAGAGATATAAAAACTAGTGCTATTGCCGCTACACCTCCCGCTTTGT 346765

||||||||||||||||||||||||||||||||||||||||||||||||||||||||||||

Sbjct 115319 AAGGTAAAGCCAAGAGAGATATAAAAACTAGTGCTATTGCCGCTACACCTCCCGCTTTGT 115378

Query 346766 CAGGTATACTGCGAAGAATGGCATAGATCGGTAGGAAATACCATTCCGGCACAATATGAG 346825

||||||||||||||||||||||||||||||||||||||||||||||||||||||||||||

Sbjct 115379 CAGGTATACTGCGAAGAATGGCATAGATCGGTAGGAAATACCATTCCGGCACAATATGAG 115438

Query 346826 GCGGGGTGGACATCGGATTAGCAGGTATATAATTGTCGGGATGCCCCAAAACATTAGGAG 346885

||||||||||||||||||||||||||||||||||||||||||||||||||||||||||||

Sbjct 115439 GCGGGGTGGACATCGGATTAGCAGGTATATAATTGTCGGGATGCCCCAAAACATTAGGAG 115498

Query 346886 CATaaaaaatgaaaatggaaaaaaaGATAGCAAAAGCTACCCAACCTACTAGATCTTTGA 346945

||||||||||||||||||||||||||||||||||||||||||||||||||||||||||||

Sbjct 115499 CATAAAAAATGAAAATGGAAAAAAAGATAGCAAAAGCTACCCAACCTACTAGATCTTTGA 115558

Query 346946 CATAAAAATAAGGGTAAAAAGAAATTTTATCCATCTCTGAATGTACACCTAATGGATTAT 347005

||||||||||||||||||||||||||||||||||||||||||||||||||||||||||||

Sbjct 115559 CATAAAAATAAGGGTAAAAAGAAATTTTATCCATCTCTGAATGTACACCTAATGGATTAT 115618

Query 347006 TTGATCCATATTGATGCAATGCGGCTAGATGAAGAAGACTGGCGCCTGCTAAAATAAAGG 347065

||||||||||||||||||||||||||||||||||||||||||||||||||||||||||||

Sbjct 115619 TTGATCCATATTGATGCAATGCGGCTAGATGAAGAAGACTGGCGCCTGCTAAAATAAAGG 115678

Query 347066 GTAGTAAATAATGAAGACTaaaaaaaCGATTTAAGGTAGCATTGTCTACGGAGAAACCAC 347125

||||||||||||||||||||||||||||||||||||||||||||||||||||||||||||

Sbjct 115679 GTAGTAAATAATGAAGACTAAAAAAACGATTTAAGGTAGCATTGTCTACGGAGAAACCAC 115738

Query 347126 CCCAAAGCCAAGTAACTATGGTATCTCCTACTACAGGTATGGCGCTAGCTAAGCTTGTAA 347185

||||||||||||||||||||||||||||||||||||||||||||||||||||||||||||

Sbjct 115739 CCCAAAGCCAAGTAACTATGGTATCTCCTACTACAGGTATGGCGCTAGCTAAGCTTGTAA 115798

Query 347186 TTACAGTAGCACCCCAAAAGCTCATCTGCCCCCAAGGTAGTACGTATCCTATAAAAGCTG 347245

||||||||||||||||||||||||||||||||||||||||||||||||||||||||||||

Sbjct 115799 TTACAGTAGCACCCCAAAAGCTCATCTGCCCCCAAGGTAGTACGTATCCTATAAAAGCTG 115858

Query 347246 TTACAATCATTAAGAGGAATATGACAACTCCAATACACCAAACAAATTCCCTAGGACTGC 347305

||||||||||||||||||||||||||||||||||||||||||||||||||||||||||||

Sbjct 115859 TTACAATCATTAAGAGGAATATGACAACTCCAATACACCAAACAAATTCCCTAGGACTGC 115918

Query 347306 TATAACTCGCATAATATAGACCACGAAAAATATGAAGGTAAACCACAATGAAAAACATAC 347365

||||||||||||||||||||||||||||||||||||||||||||||||||||||||||||

Sbjct 115919 TATAACTCGCATAATATAGACCACGAAAAATATGAAGGTAAACCACAATGAAAAACATAC 115978

Query 347366 TTGCCCCATTAGCATGCATATAACGTAGCAACCAGCCCCCTTCAACATCTCTCATTATGT 347425

||||||||||||||||||||||||||||||||||||||||||||||||||||||||||||

Sbjct 115979 TTGCCCCATTAGCATGCATATAACGTAGCAACCAGCCCCCTTCAACATCTCTCATTATGT 116038

Query 347426 GTTCTACGCTGTTAAAAGCTAAATCCACATGGGGCGTGTAATGCATAGCTaaaaaaaCGC 347485

||||||||||||||||||||||||||||||||||||||||||||||||||||||||||||

Sbjct 116039 GTTCTACGCTGTTAAAAGCTAAATCCACATGGGGCGTGTAATGCATAGCTAAAAAAACGC 116098

Query 347486 CAGTCGCTATCTGAATGACTAAACAAATACCAGCTAACGAACCGAACCCCCACCAATAAC 347545

||||||||||||||||||||||||||||||||||||||||||||||||||||||||||||

Sbjct 116099 CAGTCGCTATCTGAATGACTAAACAAATACCAGCTAACGAACCGAACCCCCACCAATAAC 116158

Query 347546 TCAGATTGCTCGGGGTTGGATAATCTATCAAATGCTGGTTCAGTGTGGAGGCTATAGGTT 347605

||||||||||||||||||||||||||||||||||||||||||||||||||||||||||||

Sbjct 116159 TCAGATTGCTCGGGGTTGGATAATCTATCAAATGCTGGTTCAGTGTGGAGGCTATAGGTT 116218

Query 347606 GTTTTAGAATAGAGAATCGTTGGTTCCTCATAGTTAGTAATTTCTCTGGTCGTCAAAGTA 347665

||||||||||||||||||||||||||||||||||||||||||||||||||||||||||||

Sbjct 116219 GTTTTAGAATAGAGAATCGTTGGTTCCTCATAGTTAGTAATTTCTCTGGTCGTCAAAGTA 116278

Query 347666 CTCAGTAGTCTCTCCGGAAAGACTCTCATGTTATGTTTCTCACTATCATAGAGTGAAAAA 347725

||||||||||||||||||||||||||||||||||||||||||||||||||||||||||||

Sbjct 116279 CTCAGTAGTCTCTCCGGAAAGACTCTCATGTTATGTTTCTCACTATCATAGAGTGAAAAA 116338

Query 347726 GAGATTGACGTGGGTTCTACCAACGAAACGAAGAACTTGATGTATGAAAGGCGCTCCGAC 347785

|||||||||||||||||||||||||||||||||||||||||||||||||||||| |||||

Sbjct 116339 GAGATTGACGTGGGTTCTACCAACGAAACGAAGAACTTGATGTATGAAAGGCGCGCCGAC 116398

Query 347786 TCGTTGTAAGAACATACACTATATAAGAGTTTCTGAGTCTAGTTTGACGAGACACTATAT 347845

|||| ||||||||||| |||||||||||||||| ||||||||||||||||||| |||||

Sbjct 116399 TCGTCGTAAGAACATAGACTATATAAGAGTTTCAGAGTCTAGTTTGACGAGACGTTATAT 116458

Query 347846 ATGAGAAAAACCAACAATTCAACAACGTTGCTTGCAAAGGGCAAGGGC 347893

| || |||| | |||||||||| |||||||||||||| ||||||||||

Sbjct 116459 ACGATAAAACCTAACAATTCAATAACGTTGCTTGCAATGGGCAAGGGC 116506

Range 3: 559901 to 581481

Score:39578 bits(21432), Expect:0.0,

Identities:21539/21590(99%), Gaps:9/21590(0%), Strand: Plus/Minus

Query 156532 AGCATTAGAGATACGAATTGATTGGATACTTCATAAAACATACATAGTCGCAGCCAGCCA 156591

||||||||||||||||||||||||||||||||||||||||||||||||||||||||||||

Sbjct 581481 AGCATTAGAGATACGAATTGATTGGATACTTCATAAAACATACATAGTCGCAGCCAGCCA 581422

Query 156592 GCAGTAGTCCCACCAAAAGATGAGATGAGAATATACATACCTTTCACAGTAATATTATTG 156651

|||||||||||||||| |||||||||||||||||||||||||||||||||||||||||||

Sbjct 581421 GCAGTAGTCCCACCAAGAGATGAGATGAGAATATACATACCTTTCACAGTAATATTATTG 581362

Query 156652 ACTTCTTACCCTTTCTTTACCAGCACCAGTGCCTGTATTGGAATGTATAGCGCAAATACC 156711

||||||||||||||||||||||||||||||||||||||||||||||||||||||||||||

Sbjct 581361 ACTTCTTACCCTTTCTTTACCAGCACCAGTGCCTGTATTGGAATGTATAGCGCAAATACC 581302

Query 156712 ACTTTGAGAAGAAAAGCCTTACCCAATCTCCAACAAACAACTCGAATACACCATAACCCT 156771

||||||||||||||||||||||||||||||||||||||||||||||||||||||||||||

Sbjct 581301 ACTTTGAGAAGAAAAGCCTTACCCAATCTCCAACAAACAACTCGAATACACCATAACCCT 581242

Query 156772 TTTCATAGTAGCAAGCTTATATCTGTCTTGTAATTCCAAATTCCTTTGTCTTAAATTTCC 156831

||||||||||||||||||||||||||||||||||||||||||||||||||||||||||||

Sbjct 581241 TTTCATAGTAGCAAGCTTATATCTGTCTTGTAATTCCAAATTCCTTTGTCTTAAATTTCC 581182

Query 156832 TAGTCGAATAGACGAATTTACTGAGAGGAGAGATTTCCCATTTGGAGTTGATTTAGTTTG 156891

||||||||||||||||||||||||||||||||||||||||||||||||||||||||||||

Sbjct 581181 TAGTCGAATAGACGAATTTACTGAGAGGAGAGATTTCCCATTTGGAGTTGATTTAGTTTG 581122

Query 156892 TAATTTCATATTTTGATGCCGCACTCTTTCTTGTCCGGAGGAAGAATTTCATGGAATAGA 156951

||||||||||||||||||||||||||||||||||||||||||||||||||||||||||||

Sbjct 581121 TAATTTCATATTTTGATGCCGCACTCTTTCTTGTCCGGAGGAAGAATTTCATGGAATAGA 581062

Query 156952 ATTTCTTGAATTAGCTAGCTATCATAGTTTTCTTTCTTTACCCCCCAACACTGACTGGGG 157011

||||||||||||||||||||||||||||||||||||||||||||||||||||||||||||

Sbjct 581061 ATTTCTTGAATTAGCTAGCTATCATAGTTTTCTTTCTTTACCCCCCAACACTGACTGGGG 581002

Query 157012 TTGTTCAATCTTAAGTGGAGTGAGTTACCAGACGGAAGAAAGAAAATTACCTAACGTGGA 157071

||||||||||||||||||||||||||||||||||||||||||||||||||||||||||||

Sbjct 581001 TTGTTCAATCTTAAGTGGAGTGAGTTACCAGACGGAAGAAAGAAAATTACCTAACGTGGA 580942

Query 157072 GCGAGTGCTCACTTGTTAATTGAATCAGAGTATTTCTGCTCTTTGTCATTTGAATTTCCC 157131

||||||||||||||||||||||||||||||||||||||||||||||||||||||||||||

Sbjct 580941 GCGAGTGCTCACTTGTTAATTGAATCAGAGTATTTCTGCTCTTTGTCATTTGAATTTCCC 580882

Query 157132 CCGCCCGTGTTTGTTAATTCCCCTCTCTTGTTTTTGAAGGAAGGCATTTCCAGACTAAGT 157191

||||||||||||||||||||||||||||||||||||||||||||||||||||||||||||

Sbjct 580881 CCGCCCGTGTTTGTTAATTCCCCTCTCTTGTTTTTGAAGGAAGGCATTTCCAGACTAAGT 580822

Query 157192 GAGTTAAATGACTTGGCTTGCTCAATTCCTTCCCGGTGTAATTTCATAAGAGGAATAGCA 157251

||||||||||||||||||||||||||||||||||||||||||||||||||||||||||||

Sbjct 580821 GAGTTAAATGACTTGGCTTGCTCAATTCCTTCCCGGTGTAATTTCATAAGAGGAATAGCA 580762

Query 157252 CTCGTTCTGCTTTCATTTCACCTTAGAATGAGCACTTTGACCAGACAAAGCTCTAGTGCG 157311

||||||||||||||||||||||||||||||||||||||||||||||||||||||||||||

Sbjct 580761 CTCGTTCTGCTTTCATTTCACCTTAGAATGAGCACTTTGACCAGACAAAGCTCTAGTGCG 580702

Query 157312 TGGAGTATCCTTTCCTACTAGTATTGATCTGTTCACTTTAATACCGGGGATTCCCCATAT 157371

||||||||||||||||||||||||||||||||||||||||||||||||||||||||||||

Sbjct 580701 TGGAGTATCCTTTCCTACTAGTATTGATCTGTTCACTTTAATACCGGGGATTCCCCATAT 580642

Query 157372 TTCCTCTACACCCCGAGTAGTGATTTCTACGAATGAATTGCCAGAGCATAAGATTGATTA 157431

||||||||||||||||||||||||||||||||||||||||||||||||||||||||||||

Sbjct 580641 TTCCTCTACACCCCGAGTAGTGATTTCTACGAATGAATTGCCAGAGCATAAGATTGATTA 580582

Query 157432 CCTCCTCGTAGTGAGTGGATTTGTGCAGATGTATTTTCCACACTCATTGTCATTCCTTTC 157491

||||||||||||||||||||||||||||||||||||||||||||||||||||||||||||

Sbjct 580581 CCTCCTCGTAGTGAGTGGATTTGTGCAGATGTATTTTCCACACTCATTGTCATTCCTTTC 580522

Query 157492 TTTGTTTAGCATTTCTTTGTCTAGAATATCCTAATTTAGTGTACTGAATTTACTAGCTTT 157551

|||||||||||||||||||||||||||||||||||| |||||||||||||| ||||||||

Sbjct 580521 TTTGTTTAGCATTTCTTTGTCTAGAATATCCTAATTGAGTGTACTGAATTTCCTAGCTTT 580462

Query 157552 TTAGCTAGTTTATTTGTTTGACATAATATCATATGTGATTTAAGTGTGGTACCTTTTCAC 157611

||||||||||||||||||||||||||||||||||||||||||||||||||||||||||||

Sbjct 580461 TTAGCTAGTTTATTTGTTTGACATAATATCATATGTGATTTAAGTGTGGTACCTTTTCAC 580402

Query 157612 GAATTCCACTTTAGAGATTTGAGCAGAAACTCTATGGAGTGTTTTCATTGATATACCAAT 157671

||||||||||||||||||||||||||||||||||||||||||||||||||||||||||||

Sbjct 580401 GAATTCCACTTTAGAGATTTGAGCAGAAACTCTATGGAGTGTTTTCATTGATATACCAAT 580342

Query 157672 ACCTCTTAATTTCTTGATTTACCAGAAAGATTGGACTGAAGAGTGGATTAAGAGTCTTGA 157731

||||||||||||||||||||||||||||||||||||||||||||||||||||||||||||

Sbjct 580341 ACCTCTTAATTTCTTGATTTACCAGAAAGATTGGACTGAAGAGTGGATTAAGAGTCTTGA 580282

Query 157732 TGGAGTTGTTGAGTTTTGGGTCTGAATTGGAACTTCGAGTAGTAAAAGTCCTGATAGAAA 157791

||||||||||||||||||||||||||||||||||||||||||||||||||||||||||||

Sbjct 580281 TGGAGTTGTTGAGTTTTGGGTCTGAATTGGAACTTCGAGTAGTAAAAGTCCTGATAGAAA 580222

Query 157792 TTTGAGTTTGAGGTTTATTAACCTTTGAATTATTATTTAAGCACATGTGATTTATATGCA 157851

||||||||||||||||||||||||||||||||||||||||||||||||||||||||||||

Sbjct 580221 TTTGAGTTTGAGGTTTATTAACCTTTGAATTATTATTTAAGCACATGTGATTTATATGCA 580162

Query 157852 TAGACTGGAGTTTACTGTATAAGATTTATGACTAATTTTGTTTTAATTCTTCTAATTGTT 157911

||||||||||||||||||||||||||||||||||||||||||||||||||||||||||||

Sbjct 580161 TAGACTGGAGTTTACTGTATAAGATTTATGACTAATTTTGTTTTAATTCTTCTAATTGTT 580102

Query 157912 TTATTAACAGTAATAGAGCAACTGCCTTTCTTCTTTTAGTTAAGTTATGCAAAGCACTGA 157971

||||||||||||||||||||||||||||||||||||||||||||||||||||||||||||

Sbjct 580101 TTATTAACAGTAATAGAGCAACTGCCTTTCTTCTTTTAGTTAAGTTATGCAAAGCACTGA 580042

Query 157972 CTGAGACGGCTATTTCACTTAATCCTCAAGGAATGAAGGTTTGTACTGACTTCTATTGTG 158031

||||||||||||||||||||||||||||||||||||||||||||||||||||||||||||

Sbjct 580041 CTGAGACGGCTATTTCACTTAATCCTCAAGGAATGAAGGTTTGTACTGACTTCTATTGTG 579982

Query 158032 GAAACTACAATAAATCAATAAAATTGTGGCAGACTTTTATGAGATTTCATACTAGCTAAT 158091

||||||||||||||||||||||||||||||||||||||||||||||||||||||||||||

Sbjct 579981 GAAACTACAATAAATCAATAAAATTGTGGCAGACTTTTATGAGATTTCATACTAGCTAAT 579922

Query 158092 AATCTATTATTCTTATTCTGGTGTATTTTGCACCTATTTGTACTGTGTTAAttttttttt 158151

||||||||||||||||||||||||||||||||||||||||||||||||||||||||||||

Sbjct 579921 AATCTATTATTCTTATTCTGGTGTATTTTGCACCTATTTGTACTGTGTTAATTTTTTTTT 579862

Query 158152 aggttttatctttctttttATGTGAAATTCCCTCTTTTGTACGAGTGAGGCTATGTCATG 158211

||||||| ||||||||||||||||||||||||||||||||||||||||||||||||||||

Sbjct 579861 AGGTTTTCTCTTTCTTTTTATGTGAAATTCCCTCTTTTGTACGAGTGAGGCTATGTCATG 579802

Query 158212 TTAATTAAATGTATTTCTTAAAAATATCGCATAGTAATTCAAAGTTTGTTTGTATTACCT 158271

||||||||||||||||||||||||||||||||||||||||||||||||||||||||||||

Sbjct 579801 TTAATTAAATGTATTTCTTAAAAATATCGCATAGTAATTCAAAGTTTGTTTGTATTACCT 579742

Query 158272 ATAAAACGGGGAACTGAAAGAGTATGCCTGCACATGTTGCCAATCTATGTTATATGGAAA 158331

||||||||||||||||||||||||||||||||||||| ||||||||||||||||||||||

Sbjct 579741 ATAAAACGGGGAACTGAAAGAGTATGCCTGCACATGTAGCCAATCTATGTTATATGGAAA 579682

Query 158332 CGTATGCGGTAATATTTCATATTTTCTACTAtttttttttGAAGTGCAATTATCTGTTGT 158391

||||||||||||||||||||||||||||||||||||||||||||||||||||||||||||

Sbjct 579681 CGTATGCGGTAATATTTCATATTTTCTACTATTTTTTTTTGAAGTGCAATTATCTGTTGT 579622

Query 158392 CAGAGAGTTTATGAGTTATTAGTTGTGGGACTGGGAGGCACTAATTTGTAGGTTCAGTTA 158451

|||||||||||||||||||||||||||||||||| |||||||||||||||||||||||||

Sbjct 579621 CAGAGAGTTTATGAGTTATTAGTTGTGGGACTGGAAGGCACTAATTTGTAGGTTCAGTTA 579562

Query 158452 TGGAGGTTATGGTTTTTGTAGAATTTCAATATGATAGCTGCCCAAGTGGTAGAGAAAGAT 158511

||||||||||||||||||||||||||||||||||||||||||||||||||||||||||||

Sbjct 579561 TGGAGGTTATGGTTTTTGTAGAATTTCAATATGATAGCTGCCCAAGTGGTAGAGAAAGAT 579502

Query 158512 GCCCGCAGTGTGGCGGTGACAGGTAGTTGTAGTCATCAAACTATTTTATATACAAAAACA 158571

||||||||||||||||||||||||||||||||||||||||||||||||||||||||||||

Sbjct 579501 GCCCGCAGTGTGGCGGTGACAGGTAGTTGTAGTCATCAAACTATTTTATATACAAAAACA 579442

Query 158572 ATAAAGTTTTTATTTATAGCAAAGAATGAAAAGGTGAGCCTAGCTTTTCATAAGAAGTAT 158631

||||||||||||||||||||||||||||||||||||||||||||||||||||||||||||

Sbjct 579441 ATAAAGTTTTTATTTATAGCAAAGAATGAAAAGGTGAGCCTAGCTTTTCATAAGAAGTAT 579382

Query 158632 GTAGTCAGATTTATCGGTTTCTTTTTTCTGAAATTACCTTCTTCAGTGAAGAAAGCTGGG 158691

||||||||||||||||||||||||||||||||||||||||||||||||||||||||||||

Sbjct 579381 GTAGTCAGATTTATCGGTTTCTTTTTTCTGAAATTACCTTCTTCAGTGAAGAAAGCTGGG 579322

Query 158692 TTCCAAGCCGAGCTTTTTCTATGATTAAATCtttttttGTTCATTTCATTTATTGGGACA 158751

||||||||||||||||||||||||||||||||||||||||||||||||||||||||||||

Sbjct 579321 TTCCAAGCCGAGCTTTTTCTATGATTAAATCTTTTTTTGTTCATTTCATTTATTGGGACA 579262

Query 158752 TCTTCATATCAAAGACTGATAAATCTGGGACTGGGTAATTGAATTATATGCCTTGAGACA 158811

||||||||||||||||||||||||||||||||||||||||||||||||||||||||||||

Sbjct 579261 TCTTCATATCAAAGACTGATAAATCTGGGACTGGGTAATTGAATTATATGCCTTGAGACA 579202

Query 158812 GAAACATGAGAATGCTTGATTCAGCCTGCAAAGTAATGGTTCTATACAAGATCCTTTATT 158871

||||||||||||||||||||||||||||||||||||||||||||||||||||||||||||

Sbjct 579201 GAAACATGAGAATGCTTGATTCAGCCTGCAAAGTAATGGTTCTATACAAGATCCTTTATT 579142

Query 158872 TGGAAAATGATGCCAATGAACTTGAATGTATAAAATGCAGCTAAAAATGATAATAAGACG 158931

||||||||||||||||||||||||||||||||||||||||||||||||||||||||||||

Sbjct 579141 TGGAAAATGATGCCAATGAACTTGAATGTATAAAATGCAGCTAAAAATGATAATAAGACG 579082

Query 158932 ATCTCTTTTTCCTGGGGACTATGAGTAAACAGTATTTTAAGTTACATAATTTTTTCTAAC 158991

||||||||||||||||||||||||||||||||||||||||||||||||||||||||||||

Sbjct 579081 ATCTCTTTTTCCTGGGGACTATGAGTAAACAGTATTTTAAGTTACATAATTTTTTCTAAC 579022

Query 158992 GTTGTTTCGGTTTTCTTAATGGAATCTTGTCAGTGACACTTTTTTCTTAACATTGGTTCG 159051

||||||||||||||||||||||||||||||||||||||||||||||||||||||||||||

Sbjct 579021 GTTGTTTCGGTTTTCTTAATGGAATCTTGTCAGTGACACTTTTTTCTTAACATTGGTTCG 578962

Query 159052 CTAGGCTTGTCACGCTCGGGTGTTGATGTGGCACTAATGCTGATCTGGCACTAATGCTGA 159111

||||||||||||||||||||||||||||||||||||||||||||||||||||||||||||

Sbjct 578961 CTAGGCTTGTCACGCTCGGGTGTTGATGTGGCACTAATGCTGATCTGGCACTAATGCTGA 578902

Query 159112 TCTGGATAAGTTCTAGCTTACACTGTTGCAAATTGGCATTGTGCGGGCTAACGTTATTGC 159171

||||||||||||||||||||||||||||||||||||||||||||||||||||||||||||

Sbjct 578901 TCTGGATAAGTTCTAGCTTACACTGTTGCAAATTGGCATTGTGCGGGCTAACGTTATTGC 578842

Query 159172 TGATCTGACACTTTGTCAGTAGTACGCATTGCTATTGATCTGGTATGATTATGTGATATA 159231

||||||||||||||||||||||||||||||||||||||||||||||||||||||||||||

Sbjct 578841 TGATCTGACACTTTGTCAGTAGTACGCATTGCTATTGATCTGGTATGATTATGTGATATA 578782

Query 159232 ATTAGTTGCGATATACTGATATATTGTGAATCGTTCGGCTTCTTTTTCCTTAATTCAAAT 159291

||||||||||||||||||||||||||||||||||||||||||||||||||||||||||||

Sbjct 578781 ATTAGTTGCGATATACTGATATATTGTGAATCGTTCGGCTTCTTTTTCCTTAATTCAAAT 578722

Query 159292 ATTGTGAGTATTTTGTTGTTTTGGTAATAATAATTAGCTTTTGACGTTTTAACAATATGT 159351

||||||||||||||||||||||||||||||||||||||||||||||||||||||||||||

Sbjct 578721 ATTGTGAGTATTTTGTTGTTTTGGTAATAATAATTAGCTTTTGACGTTTTAACAATATGT 578662

Query 159352 AAGAATCTACTCAGAATTTGTTTTCTTAAGTTAGTTATTTTAATATTTAATTACACAAGC 159411

||||||||||||||||||||||||||||||||||||||||||||||||||||||||||||

Sbjct 578661 AAGAATCTACTCAGAATTTGTTTTCTTAAGTTAGTTATTTTAATATTTAATTACACAAGC 578602

Query 159412 TCGACCACTAGAAACCTGCATTTACTACATGCCCTGGTATAGCAGAATCTTGGGAATGCA 159471

||||||||||||||||||||||||||||||||||||||||||||||||||||||||||||

Sbjct 578601 TCGACCACTAGAAACCTGCATTTACTACATGCCCTGGTATAGCAGAATCTTGGGAATGCA 578542

Query 159472 GAATTGTAGAATCACTAATCGCAAGGAGCTTGAGTGTTTGCAAGGGGATCATATGTAGAA 159531

||||||||||||||||||||||||||||||||||||||||||||||||||||||||||||

Sbjct 578541 GAATTGTAGAATCACTAATCGCAAGGAGCTTGAGTGTTTGCAAGGGGATCATATGTAGAA 578482

Query 159532 TCACTACATGCGATATTTTGTCTCTCTTCACAAGTAATTAATTTTCATTAGATCTTTTGC 159591

||||||||||||||||||||||||||||||||||||||||||||||||||||||||||||

Sbjct 578481 TCACTACATGCGATATTTTGTCTCTCTTCACAAGTAATTAATTTTCATTAGATCTTTTGC 578422

Query 159592 ATTGTCTTGCTCATAGTAAAGATGGCTTATTTCCTTTTAGTGCAGAATGGGTGCTCGAGA 159651

||||||||||||||||||||||||||||||||||||||||||||||||||||||||||||

Sbjct 578421 ATTGTCTTGCTCATAGTAAAGATGGCTTATTTCCTTTTAGTGCAGAATGGGTGCTCGAGA 578362

Query 159652 AACACCTGTAGCAAGCTCTTGACGGAGCCGACAATGCATATCATTGGCCTCGGTATTTTC 159711

||||||||||||||||||||||||||||||||||||||||||||||||||||||||||||

Sbjct 578361 AACACCTGTAGCAAGCTCTTGACGGAGCCGACAATGCATATCATTGGCCTCGGTATTTTC 578302

Query 159712 CAGTATATAGTGGGTAGAATTCTCTCTTCCTTTACCCTTGGGGATGTTTTGTAGCTTGAG 159771

||||||||||||||||||||||||||||||||||||||||||||||||||||||||||||

Sbjct 578301 CAGTATATAGTGGGTAGAATTCTCTCTTCCTTTACCCTTGGGGATGTTTTGTAGCTTGAG 578242

Query 159772 ACTACAAGCTCTAGGGGCTTCAGAGGTCTGTTGTGGAGGCCTCGACTCAGTTTGCATATC 159831

||||||||||||||||||||||||||||||||||||||||||||||||||||||||||||

Sbjct 578241 ACTACAAGCTCTAGGGGCTTCAGAGGTCTGTTGTGGAGGCCTCGACTCAGTTTGCATATC 578182

Query 159832 AGGTATAATCACTGCTGCCTCCATTTTCCTTTTTTCCCCTCGGGGGAGGTTCAATTGATG 159891

||||||||||||||||||||||||||||||||||||||||||||||||||||||||||||

Sbjct 578181 AGGTATAATCACTGCTGCCTCCATTTTCCTTTTTTCCCCTCGGGGGAGGTTCAATTGATG 578122

Query 159892 GTGATTATTGTAATTTGAATTGGATAACATGAAGGTTGAAGGGATATTCTCATTCAGGTC 159951

||||||||||||||||||||||||||||||||||||||||||||||||||||||||||||

Sbjct 578121 GTGATTATTGTAATTTGAATTGGATAACATGAAGGTTGAAGGGATATTCTCATTCAGGTC 578062

Query 159952 TGTTGTGCTCTCTATCCCTCAAAATTCAGTTTCAATTTCATTCCAGTTATGTGCTGATCT 160011

|||||||||||||||||||||||||| |||||||||||||||||||||||||||||||||

Sbjct 578061 TGTTGTGCTCTCTATCCCTCAAAATTAAGTTTCAATTTCATTCCAGTTATGTGCTGATCT 578002

Query 160012 ATGGCTTTAAAGTTTCAACCATTCCGACTGCTAACCACTAAGGACTCAGATCAATATTAC 160071

||||||||||||||||||||||||||||||||||||||||||||||||||||||||||||

Sbjct 578001 ATGGCTTTAAAGTTTCAACCATTCCGACTGCTAACCACTAAGGACTCAGATCAATATTAC 577942

Query 160072 GCTTTATTTACATACAACTCGAGACTCTTACGAGCGGCTGTGCGATTGATTCCCTTACTC 160131

||||||||||||||||||||||||||||||||||||||||||||||||||||||||||||

Sbjct 577941 GCTTTATTTACATACAACTCGAGACTCTTACGAGCGGCTGTGCGATTGATTCCCTTACTC 577882

Query 160132 AAATGCAAAGTTTTGGATTCATGATTAACTCTGATGTCTTAGGCTTTATAGAATAAAATA 160191

||||||||||||||||||||||||||||||||||||||||||||||||||||||||||||

Sbjct 577881 AAATGCAAAGTTTTGGATTCATGATTAACTCTGATGTCTTAGGCTTTATAGAATAAAATA 577822

Query 160192 TGACTGGGTTAGTTGATGCTGGACTACTGATGCCCCCTTATCTTTCTCGAGTGAATATCT 160251

||||||||||||||||||||||||||||||||||||||||||||||||||||||||||||

Sbjct 577821 TGACTGGGTTAGTTGATGCTGGACTACTGATGCCCCCTTATCTTTCTCGAGTGAATATCT 577762

Query 160252 CTCAGCTAGGTCTCTTCAGAGGTTTTTAAAGGATAATAAGTATGATAAGTATAGGTTTTC 160311

||||||||||||||||||||||||||||||||||||||||||||||||||||||||||||

Sbjct 577761 CTCAGCTAGGTCTCTTCAGAGGTTTTTAAAGGATAATAAGTATGATAAGTATAGGTTTTC 577702

Query 160312 AACGCTGTGGGTTGGaaaaaaaGTGTTCAACAAGCGCGATATGAGAGGTTTTTATTGGAA 160371

||||||||||||||||||||||||||||||||||||||||||||||||||||||||||||

Sbjct 577701 AACGCTGTGGGTTGGAAAAAAAGTGTTCAACAAGCGCGATATGAGAGGTTTTTATTGGAA 577642

Query 160372 CTGGCTAAGGCTTACGATGGGTATCAGTTATACTTCCCAGCATTCATTTATTTCAGAGGA 160431

||||||||||||||||||||||||||||||||||||||||||||||||||||||||||||

Sbjct 577641 CTGGCTAAGGCTTACGATGGGTATCAGTTATACTTCCCAGCATTCATTTATTTCAGAGGA 577582

Query 160432 AGAATCTACCAATCTGGTATTCTACACTTTCCCGAAAAGGATATAGTGAAAAGTATGATA 160491

||||||||||||||||||||||||||||||||||||||||||||||||||||||||||||

Sbjct 577581 AGAATCTACCAATCTGGTATTCTACACTTTCCCGAAAAGGATATAGTGAAAAGTATGATA 577522

Query 160492 TGCTTTTATCCTCCTAAGGACGCTGTTATTAATAATTATTAGGATGCATATCGTACTCTG 160551

||||||||||||||||||||||||||||||||||||||||||||||||||||||||||||

Sbjct 577521 TGCTTTTATCCTCCTAAGGACGCTGTTATTAATAATTATTAGGATGCATATCGTACTCTG 577462

Query 160552 TTAGAAGCCACTGCATATCACTATAAAACATTTAATAGCCGTAAAGAAGCTGTAGGAGGG 160611

||||||||||||||||||||||||||||||||||||||||||||||||||||||||||||

Sbjct 577461 TTAGAAGCCACTGCATATCACTATAAAACATTTAATAGCCGTAAAGAAGCTGTAGGAGGG 577402

Query 160612 TCTAAAGTTTAGTCGTTTGAATGCATTAGAAAGAAAGGACTTATATATGTACTGGTCTAC 160671

||||||||||||||||||||||||||||||||||||||||||||||||||||||||||||

Sbjct 577401 TCTAAAGTTTAGTCGTTTGAATGCATTAGAAAGAAAGGACTTATATATGTACTGGTCTAC 577342

Query 160672 tttttttCTTCATGTTCGCTTATGCTTGCTTCTTTCTTAGCTTGGGTACTGGTATACTTT 160731

||||||||||||||||||||||||||||||||||||||||||||||||||||||||||||

Sbjct 577341 TTTTTTTCTTCATGTTCGCTTATGCTTGCTTCTTTCTTAGCTTGGGTACTGGTATACTTT 577282

Query 160732 AATAAGTTTTTACTTTCGTGGATGCTCGTAGATAGATTGAtttttcctgttcttcttttc 160791

||||||||||||||||||||||||||||||||||||||||||||||||||||||||||||

Sbjct 577281 AATAAGTTTTTACTTTCGTGGATGCTCGTAGATAGATTGATTTTTCCTGTTCTTCTTTTC 577222

Query 160792 ttctctcttttctggttgtcttctCGTGGCTGAGTGCCGAGTGATATCGGCCTTTTCTAT 160851

||||||||||||||||||||||||||||||||||||||||||||||||||||||||||||

Sbjct 577221 TTCTCTCTTTTCTGGTTGTCTTCTCGTGGCTGAGTGCCGAGTGATATCGGCCTTTTCTAT 577162

Query 160852 CTCCTCGCAGGGTCAATCCATTAGGGGTGTCAATCTCTCTGCCATTGAGTGAGGGGGCCA 160911

||||||||||||||||||||||||||||||||||||||||||||||||||||||||||||

Sbjct 577161 CTCCTCGCAGGGTCAATCCATTAGGGGTGTCAATCTCTCTGCCATTGAGTGAGGGGGCCA 577102

Query 160912 ATCACATTCAGCAGTGACATCAAAATGTGCTACAAACCAGTGTTCGGCAAAGCCCGCCCG 160971

||||||||||||||||||||||||||||||||||||||||||||||||||||||||||||

Sbjct 577101 ATCACATTCAGCAGTGACATCAAAATGTGCTACAAACCAGTGTTCGGCAAAGCCCGCCCG 577042

Query 160972 CCAGGCCATTCAACAAAGTTTTGATTTGACTGGAAAGACGTTAAACACGAACCGCCAATG 161031

||||||||||||||||||||||||||||||||||||||||||||||||||||||||||||

Sbjct 577041 CCAGGCCATTCAACAAAGTTTTGATTTGACTGGAAAGACGTTAAACACGAACCGCCAATG 576982

Query 161032 CAGGAACCGCACCCCAGCACCGTTTGCCATTCATTCATGAACTCTCTTTTATCTACTTCC 161091

||||||||||||||||||||||||||||||||||||||||||||||||||||||||||||

Sbjct 576981 CAGGAACCGCACCCCAGCACCGTTTGCCATTCATTCATGAACTCTCTTTTATCTACTTCC 576922

Query 161092 AACGATTCCTACTTACTCTTATTTCAAACTTTCCGGGAATTTCCATAACCCCAGGACACT 161151

||||||||||||||||||||||||||||||||||||||||||||||||||||||||||||

Sbjct 576921 AACGATTCCTACTTACTCTTATTTCAAACTTTCCGGGAATTTCCATAACCCCAGGACACT 576862

Query 161152 AAATAAAGATCTATTCTTTTCTAATTGAAGGCACCCAAAGCTCATTGGGCTTTCATAGGC 161211

|||||||||||||||||||| |||||||||||||||||||||||||||||||||||||||

Sbjct 576861 AAATAAAGATCTATTCTTTTATAATTGAAGGCACCCAAAGCTCATTGGGCTTTCATAGGC 576802

Query 161212 ATAAGGGGAAACGTGTCATATGCCTTTTTTACAACTATTTCTCTCTCTGCTATACTTGAC 161271

||||||||||||||||||||||||||||||||||||||||||||||||||||||||||||

Sbjct 576801 ATAAGGGGAAACGTGTCATATGCCTTTTTTACAACTATTTCTCTCTCTGCTATACTTGAC 576742

Query 161272 TTAGAAGCTTCTCCTCAACCTACTCATTCGCATGCATAAACCTACTCGCTTCGCGCCTCA 161331

||||||||||||||||||||||||||||||||||||||||||||||||||||||||||||

Sbjct 576741 TTAGAAGCTTCTCCTCAACCTACTCATTCGCATGCATAAACCTACTCGCTTCGCGCCTCA 576682

Query 161332 AGCAACTACTTGACCTAATGCCTAtttttttGTTTACCTATCCATGACATGACCTTGGCT 161391

||||||||||||||||||||||||||||||||||||||||||||||||||||||||||||

Sbjct 576681 AGCAACTACTTGACCTAATGCCTATTTTTTTGTTTACCTATCCATGACATGACCTTGGCT 576622

Query 161392 AGCCATTCACAAATACGTGCATACTTCTTCCTAAACTACGTACGCTCCCTTGCTGCACTT 161451

||||||||||||||||||||||||||||||||||||||||||||||||||||||||||||

Sbjct 576621 AGCCATTCACAAATACGTGCATACTTCTTCCTAAACTACGTACGCTCCCTTGCTGCACTT 576562

Query 161452 ACTAACTAAGCCGCCTATAAATCATAAAAAAGATAGCTActtccctttctttctacttca 161511

||||||||||||||||||||||||||||||||||||||||||||||||||||||||||||

Sbjct 576561 ACTAACTAAGCCGCCTATAAATCATAAAAAAGATAGCTACTTCCCTTTCTTTCTACTTCA 576502

Query 161512 aattttctactttgaatgaattttcatttatcttttcttcttttcAATCCTTAAGCTTCT 161571

| ||||||||||||||||||||||||||||||||||||||||||||||||||||||||||

Sbjct 576501 ATTTTTCTACTTTGAATGAATTTTCATTTATCTTTTCTTCTTTTCAATCCTTAAGCTTCT 576442

Query 161572 TACAGTGAAGCGTCGTCTAAGGAAAAAGGCCTATTTTCAAAGCCAATAATTAAGCCAGCT 161631

||||||||||||||||||||||||||||||||||||||||||||||||||||||||||||

Sbjct 576441 TACAGTGAAGCGTCGTCTAAGGAAAAAGGCCTATTTTCAAAGCCAATAATTAAGCCAGCT 576382

Query 161632 TATCACACTTCTGAGCTCCTCTGAATATACCAGTATACTTTGTCTTGCTACGGGAGAGGG 161691

||||||||||||||||||||||||||||||||||||||||||||||||||||||||||||

Sbjct 576381 TATCACACTTCTGAGCTCCTCTGAATATACCAGTATACTTTGTCTTGCTACGGGAGAGGG 576322

Query 161692 ATGAAGAGCAGGATCTGATATCGCTCGGAGTCATGTCATATGACCTGAAAGTAAAACATC 161751

||||||||||||||||||||||||||||||||||||||||||||||||||||||||||||

Sbjct 576321 ATGAAGAGCAGGATCTGATATCGCTCGGAGTCATGTCATATGACCTGAAAGTAAAACATC 576262

Query 161752 GGTACGCAGCAAAAGTGGAGCAAAGCTTTCAACATCCACGGGTGTGAACATAAGATAGGC 161811

||||||||||||||||||||||||||||||||||||||||||||||||||||||||||||

Sbjct 576261 GGTACGCAGCAAAAGTGGAGCAAAGCTTTCAACATCCACGGGTGTGAACATAAGATAGGC 576202

Query 161812 TATTTTGGCTGTATCTTTGAGCGAACACTTATTTTTCTGTCCACCACCTTTTTCTTTAAG 161871

||||||||||||||||||||||||||||||||||||||||||||||||||||||||||||

Sbjct 576201 TATTTTGGCTGTATCTTTGAGCGAACACTTATTTTTCTGTCCACCACCTTTTTCTTTAAG 576142

Query 161872 ATCTGGAGGGTCGGCTAGCTACTGGGATAGCATTCAATGACTGGCTATTGAAGGGTTTTG 161931

||||||||||||||||||||||||||||||||||||||||||||||||||||||||||||

Sbjct 576141 ATCTGGAGGGTCGGCTAGCTACTGGGATAGCATTCAATGACTGGCTATTGAAGGGTTTTG 576082

Query 161932 AATAATTCTTAATAAGAATAACGTGGTCTAGAGGTAAGAAAGACTGAGGCTCGGCTAGAT 161991

||||||||||||||||||||||||||||||||||||||||||||||||||||||||||||

Sbjct 576081 AATAATTCTTAATAAGAATAACGTGGTCTAGAGGTAAGAAAGACTGAGGCTCGGCTAGAT 576022

Query 161992 GATGCACGTAGAGAGCGTAGACCGTAGGACGTAGATCGAGTAGGTGAAAACCGGACTGAT 162051

||||||||||||||||||||||||||||||||||||||||||||||||||||||||||||

Sbjct 576021 GATGCACGTAGAGAGCGTAGACCGTAGGACGTAGATCGAGTAGGTGAAAACCGGACTGAT 575962

Query 162052 GAAGAGCTTGATCCTTATCCTGTTCCAATTGAAAAAACAGTGGACTCAAGATTTTCCCCA 162111

||||||||||||||||||||||||||||||||||||||||||||||||||||||||||||

Sbjct 575961 GAAGAGCTTGATCCTTATCCTGTTCCAATTGAAAAAACAGTGGACTCAAGATTTTCCCCA 575902

Query 162112 CCTTGCTCGTATGCCGATGGTGCTAGTGTTTAAGCTTACTTTTTTCCCACTCCTGGCTGC 162171

||||||||||||||||||||||||||||||| ||||||||||||||||||||||||||||

Sbjct 575901 CCTTGCTCGTATGCCGATGGTGCTAGTGTTTCAGCTTACTTTTTTCCCACTCCTGGCTGC 575842

Query 162172 CAGGCTATGTCAATGCTGATTTACCCAAGTCCTTTGCTTGCTTTGGCACAGTATGAGGAT 162231

||||||||||||||||||||||||||||||||||||||||||||||||||||||||||||

Sbjct 575841 CAGGCTATGTCAATGCTGATTTACCCAAGTCCTTTGCTTGCTTTGGCACAGTATGAGGAT 575782

Query 162232 GAGGCTCACTTCTGGACTTCAAATCCCTCAAAGTATCCTTTTCAGAAGGAACAAGAGGTT 162291

||||||||||||||||||| ||||||||||||||||||||||||||||||||||||||||

Sbjct 575781 GAGGCTCACTTCTGGACTTAAAATCCCTCAAAGTATCCTTTTCAGAAGGAACAAGAGGTT 575722

Query 162292 CAATTCATTCAGCATCAGTTGATCAAGGGCTTTTATCTGTTTAATCCTATGCTCCGAACA 162351

||||||||||||||||||||||||||||||||||||||||||||||||||||||||||||

Sbjct 575721 CAATTCATTCAGCATCAGTTGATCAAGGGCTTTTATCTGTTTAATCCTATGCTCCGAACA 575662

Query 162352 TGGATACCAAAGAAAGACAAAGAACCCAAAGGAGCTTCGACCTATAACTAAACCTGCCGA 162411

||||||||||||||||||||||||||||||||||||||||||||||||||||||||||||

Sbjct 575661 TGGATACCAAAGAAAGACAAAGAACCCAAAGGAGCTTCGACCTATAACTAAACCTGCCGA 575602

Query 162412 AAAAGATCAACTGGTGCTAGATGCGATGACCGTTCTAATGACTGAGGCGATGGACCCTAT 162471

||||||||||||||||||||||||||||||||||||||||||||||||||||||||||||

Sbjct 575601 AAAAGATCAACTGGTGCTAGATGCGATGACCGTTCTAATGACTGAGGCGATGGACCCTAT 575542

Query 162472 CTTTCTGGATCATTCACACGGCTTCATAAAAAGAAGAGGAGCAAAAACCCTTTTTCCACG 162531

|||||||||||||||||||||||||| |||||||||||||||||||||||||||||||||

Sbjct 575541 CTTTCTGGATCATTCACACGGCTTCAGAAAAAGAAGAGGAGCAAAAACCCTTTTTCCACG 575482

Query 162532 ACCTAAGTAAGTGGCCTGATATGGATTGCATTATCAAGTGCGATGTTCGGCACTGTTTTG 162591

||||||||||||||||||||||||||||||||||||||||||||||||||||||||||||

Sbjct 575481 ACCTAAGTAAGTGGCCTGATATGGATTGCATTATCAAGTGCGATGTTCGGCACTGTTTTG 575422

Query 162592 AGAGTATTAGTCATGACATACTCCTTAAGACCCTTAGGGATCACTTAGGGCAAGAGAACA 162651

||||||||||||||||||||||||||||||||||||||||||||||||||||||||||||

Sbjct 575421 AGAGTATTAGTCATGACATACTCCTTAAGACCCTTAGGGATCACTTAGGGCAAGAGAACA 575362

Query 162652 AGGCTTTCATTCACCTAATAAAGCGCTTTCTCACAACCCAGATCCTCGACCGTAAAGGAA 162711

||||||||||||||||||||||||||||||||||||||||||||||||||||||||||||

Sbjct 575361 AGGCTTTCATTCACCTAATAAAGCGCTTTCTCACAACCCAGATCCTCGACCGTAAAGGAA 575302

Query 162712 ATGACTATGCTTGCTATGAAGTTGGTATCCCTCAAGGGAGCCCTGTTTCTCCTACCCTCA 162771

||||||||||||||||||||||||||||||||||||||||||||||||||||||||||||

Sbjct 575301 ATGACTATGCTTGCTATGAAGTTGGTATCCCTCAAGGGAGCCCTGTTTCTCCTACCCTCA 575242

Query 162772 TGAATTGCTTCTTACACTTATTGGATCAATCCTTTTCATCTCATTGTCAGTCACGACATC 162831

||||||||||||||||||||||||||||||||||||||||||||||||||||||||||||

Sbjct 575241 TGAATTGCTTCTTACACTTATTGGATCAATCCTTTTCATCTCATTGTCAGTCACGACATC 575182

Query 162832 AAATAGGAAAAAATATGCCATGATAGCTTGGAAAAAACTATGTATGCCTAAGGAATTGGG 162891

||||||||||||||||||||||||||||||||||||||||||||||||||||||||||||

Sbjct 575181 AAATAGGAAAAAATATGCCATGATAGCTTGGAAAAAACTATGTATGCCTAAGGAATTGGG 575122

Query 162892 AGGGATGGGCATTCTGGATCTACATAATATGAAAATTTCCCTTCTCCTCAAACGGCTGTG 162951

||||||||||||||||||||||||||||||||||||||||||||||||||||||||||||

Sbjct 575121 AGGGATGGGCATTCTGGATCTACATAATATGAAAATTTCCCTTCTCCTCAAACGGCTGTG 575062

Query 162952 GAGATTAAAAGATCCTCTTTACAATAGTGTCTGGAAAGAAACAAATGATTAATGCAAAAT 163011

||||||||||||||||||||||||||||||||||||||||||||||||||||||||||||

Sbjct 575061 GAGATTAAAAGATCCTCTTTACAATAGTGTCTGGAAAGAAACAAATGATTAATGCAAAAT 575002

Query 163012 AGAATTCTAATACACATCAACAAAATATGTCTCCCCTGTGGAAAGAAATTATGAAATTAA 163071

||||||||||||||||||||||||||||||||||||||||||||||||||||||||||||

Sbjct 575001 AGAATTCTAATACACATCAACAAAATATGTCTCCCCTGTGGAAAGAAATTATGAAATTAA 574942

Query 163072 ATACAATTGGCTCTATAGGATGTAAGTTTCTAGTTGGTAATGGTAGGTCTATAAAATTAT 163131

|||||||||||||||||||||||||||||||||||||||||||||||||||||||||| |

Sbjct 574941 ATACAATTGGCTCTATAGGATGTAAGTTTCTAGTTGGTAATGGTAGGTCTATAAAATTCT 574882

Query 163132 GGTCTGACATAAGGTTTCAAGAATGCCCACTTTCTTCATTTTTTCCTCAACTTTACAACA 163191

||||||||||||||||||||||||||||||||||||||||||||||||||||||||||||

Sbjct 574881 GGTCTGACATAAGGTTTCAAGAATGCCCACTTTCTTCATTTTTTCCTCAACTTTACAACA 574822

Query 163192 TTTTTCAGTCAAAAAATATTACTGTTTTTGATGCCATTTTATCTAAAGGGCAGGATCTGA 163251

||||||||||||||||||||||||||||||||||||||||||||||||||||||||||||

Sbjct 574821 TTTTTCAGTCAAAAAATATTACTGTTTTTGATGCCATTTTATCTAAAGGGCAGGATCTGA 574762

Query 163252 CATTTTCTAGACAATTATCAGGGGTATTATTAATTGAATTCAATGAATTGTGTACCATTA 163311

||||||||||||||||||||||||||||||||||||||||||||||||||||||||||||

Sbjct 574761 CATTTTCTAGACAATTATCAGGGGTATTATTAATTGAATTCAATGAATTGTGTACCATTA 574702

Query 163312 TTTCTTCTTGTCAGTTATCTTTAGCACATGATCAAATAGTGTGGAGATGGAGCAGCTCAG 163371

||||||||||||||||||||||||||||||||||||||||||||||||||||||||||||

Sbjct 574701 TTTCTTCTTGTCAGTTATCTTTAGCACATGATCAAATAGTGTGGAGATGGAGCAGCTCAG 574642

Query 163372 GTTCATTTACTTCACAATCAGCCTATGAATGGCTCTCATTTATTTAGAGGAGTGGTAGAT 163431

||||||||||||||||||||||||||||||||||||||||||||||||||||||||||||

Sbjct 574641 GTTCATTTACTTCACAATCAGCCTATGAATGGCTCTCATTTATTTAGAGGAGTGGTAGAT 574582

Query 163432 AAAGATAGCCAAATATGGTGGAATTTACATCTGCCTTTGAAAATAAAAGTGTTCATGTGG 163491

||||||||||||||||||||||||||||||||||||||||||||||||||||||||||||

Sbjct 574581 AAAGATAGCCAAATATGGTGGAATTTACATCTGCCTTTGAAAATAAAAGTGTTCATGTGG 574522

Query 163492 ATGGTTTTTAAACAAAGAATAACTACTAGGGACCAAATGATCAAAAGAGGGTGGCCAGGA 163551

||||||||||||||||||||||||||||||||||||||||||||||||||||||||||||

Sbjct 574521 ATGGTTTTTAAACAAAGAATAACTACTAGGGACCAAATGATCAAAAGAGGGTGGCCAGGA 574462

Query 163552 AATCCACAGTGCAGTTTCTGTTCTTTACCAGAATCAGTGGAACACATTTTTCTACATTGT 163611

||||||||||||||||||||||||||||||||||||||||||||||||||||||||||||

Sbjct 574461 AATCCACAGTGCAGTTTCTGTTCTTTACCAGAATCAGTGGAACACATTTTTCTACATTGT 574402

Query 163612 CAATTTTTTAAATTCTTATGGTTTTGGATGGGAGATTGCCAATTGCTATACCCTCACTGG 163671

|||||||| |||||||||||||||||||||||||||||||||||||||||||||||| ||

Sbjct 574401 CAATTTTTGAAATTCTTATGGTTTTGGATGGGAGATTGCCAATTGCTATACCCTCACAGG 574342

Query 163672 TCAAATATGAATGATATATTATGTTTTGCAAATTGTTTCCCAAAGAATGAAAAAGAAGCT 163731

||||||||||||||| |||||||||||||||||||||| |||||||||||||||||||||

Sbjct 574341 TCAAATATGAATGATCTATTATGTTTTGCAAATTGTTTACCAAAGAATGAAAAAGAAGCT 574282

Query 163732 TTCTTAATTGTGATCAGTGCACTATTCTGGGCTCTTTGGAAAATTAGAAATGATATATAT 163791

||||||||||||||||||||||||||||||||||||||||||||||||||||||||||||

Sbjct 574281 TTCTTAATTGTGATCAGTGCACTATTCTGGGCTCTTTGGAAAATTAGAAATGATATATAT 574222

Query 163792 TACTTTTAATGGTACAAATTTAATAACTGTCAGAAATGCTGTGTTACTGATCCTGTCTTG 163851

||||||||||||||||||||||||||||||||||||||||||||||||||||||||||||

Sbjct 574221 TACTTTTAATGGTACAAATTTAATAACTGTCAGAAATGCTGTGTTACTGATCCTGTCTTG 574162

Query 163852 GTTGAATTATTGGCTAGGGAATGTCCCAGGTGAGGTGAAGAGACATGTGCAAAGGTGGGT 163911

||||||||||||||||||||||||||||||||||||||||||||||||||||||||||||

Sbjct 574161 GTTGAATTATTGGCTAGGGAATGTCCCAGGTGAGGTGAAGAGACATGTGCAAAGGTGGGT 574102

Query 163912 GCCCAAGAGCTTGGAGGAGATACCTATCCAGATGAGGCCACCAGTTCTTGCAATTGCTGG 163971

||||||||||||||||||||||||||||||||||||||||||||||||||||||||||||

Sbjct 574101 GCCCAAGAGCTTGGAGGAGATACCTATCCAGATGAGGCCACCAGTTCTTGCAATTGCTGG 574042

Query 163972 TTGATGAAGATAAGAGATGATGTCATAGAACTTTGGTTCAAAAAGTGATAATGAAGTAGT 164031

||||||||||||||||||||||||||||||||||||||||||||||||||||||||||||

Sbjct 574041 TTGATGAAGATAAGAGATGATGTCATAGAACTTTGGTTCAAAAAGTGATAATGAAGTAGT 573982

Query 164032 TACATATGTAGCAGTTAATTGTCTCAGTTCAGCAGTTAAATTGTTTTTTGTTTAGTCTAT 164091

||||||||||||||||||||||||||||||||||||||||||||||||||||||||||||

Sbjct 573981 TACATATGTAGCAGTTAATTGTCTCAGTTCAGCAGTTAAATTGTTTTTTGTTTAGTCTAT 573922

Query 164092 GTTAGTGCTGAGTAGGAGAACTTGTTGATGTTTCTGGCCTGTGTGATTGGGCTGAAGTTT 164151

||||||||||||||||||||||||||||||||||||||||||||||||||||||||||||

Sbjct 573921 GTTAGTGCTGAGTAGGAGAACTTGTTGATGTTTCTGGCCTGTGTGATTGGGCTGAAGTTT 573862

Query 164152 GATTACGAGTAGGTTTAAGTGCTTTGAGATGGGCTTTTTAGCCGTAGGGATCATAAAACC 164211

||||||||||||||||||||||||||||||||||||||||||||||||||||||||||||

Sbjct 573861 GATTACGAGTAGGTTTAAGTGCTTTGAGATGGGCTTTTTAGCCGTAGGGATCATAAAACC 573802

Query 164212 TTGTAGTTCTGGTTTGTATTTAATGAATGGGCTCAATAGCCTTGTTTaaaaaaaaaaaaG 164271

|||||||||||||||||||||||||||||||||||||||||||||||||||||||||| |

Sbjct 573801 TTGTAGTTCTGGTTTGTATTTAATGAATGGGCTCAATAGCCTTGTTTAAAAAAAAAAA-G 573743

Query 164272 AATAAGCGTAGAAAGAAGAAACTCCTCTCGCAAGTTTCAAATACACTCAAAACGAGACTA 164331

||||||||||||||||||||||||||||||||||||||||||||||||||||||||||||

Sbjct 573742 AATAAGCGTAGAAAGAAGAAACTCCTCTCGCAAGTTTCAAATACACTCAAAACGAGACTA 573683

Query 164332 TGCATTTCGAAATGAAAAGCTATAGCTATCCTGAAAAGAAAGGCTCATTCTAATTTCCCC 164391

||||||||||||||||||||||||||||||||||||||||||||||||||||||||||||

Sbjct 573682 TGCATTTCGAAATGAAAAGCTATAGCTATCCTGAAAAGAAAGGCTCATTCTAATTTCCCC 573623

Query 164392 TGGTCATAAGACAGTAAACTACTATAGATAAGGTCTACCTACGGGAAAGGCTACAGCTTT 164451

||||||||||||||||||||||||||||||||||||||||||||||||||||||||||||

Sbjct 573622 TGGTCATAAGACAGTAAACTACTATAGATAAGGTCTACCTACGGGAAAGGCTACAGCTTT 573563

Query 164452 GGCTTGATGCGGGGAGATTCGCGGAGAGGTTTCAGGAGAGGGTCATTTTTTATCAATAGA 164511

||||||||||||||||||||||||||||||||||||||||||||||||||| ||||||||

Sbjct 573562 GGCTTGATGCGGGGAGATTCGCGGAGAGGTTTCAGGAGAGGGTCATTTTTTCTCAATAGA 573503

Query 164512 GAATGCCTCATGCGTGACCTACCTAGTATTGGAAAACATTCTTCATCTTTATGCAACCAT 164571

||||||||||||||||||||||||||||||||||||||||||||||||||||||||||||

Sbjct 573502 GAATGCCTCATGCGTGACCTACCTAGTATTGGAAAACATTCTTCATCTTTATGCAACCAT 573443

Query 164572 AATCCTCCCAACCACTCTATGGATCATTGCCACCGCTCTTATGTTTTCGCACGCAGCTCC 164631

||||||||||||||||||||||||||||||||||||||||||||||||||||||||||||

Sbjct 573442 AATCCTCCCAACCACTCTATGGATCATTGCCACCGCTCTTATGTTTTCGCACGCAGCTCC 573383

Query 164632 GCTCCCGGTACGCAAAAGCCTTTGTGACACTGTAATGGAACAAATCTGTAAGCTCCATCA 164691

||||||||||||||||||||||||||||||||||||||||||||||||||||||||||||

Sbjct 573382 GCTCCCGGTACGCAAAAGCCTTTGTGACACTGTAATGGAACAAATCTGTAAGCTCCATCA 573323

Query 164692 CGATCTTTTTCCTCAATATGGGCTTACCCCCTTAGCGCCCTTCGTATAGTTTGAAACTTA 164751

||||||||||||||||||||||||||||||||||||||||||||||||||||||||||||

Sbjct 573322 CGATCTTTTTCCTCAATATGGGCTTACCCCCTTAGCGCCCTTCGTATAGTTTGAAACTTA 573263

Query 164752 GCTAACGAGGTGACAGAGCCCGAACGGTTTAATGGGCTATGCAATATTTACCAGAGTTTT 164811

||||||||||||||||||||||||||||||||||||||||||||||||||||||||||||

Sbjct 573262 GCTAACGAGGTGACAGAGCCCGAACGGTTTAATGGGCTATGCAATATTTACCAGAGTTTT 573203

Query 164812 GTCGAAAATGGAATCGACCCATCGTGAGGGAATTTCTTCTTACTGGGGGTGGAGGGTTGT 164871

||||||||||||||||||||||||||||||||||||||||||||||||||||||||||||

Sbjct 573202 GTCGAAAATGGAATCGACCCATCGTGAGGGAATTTCTTCTTACTGGGGGTGGAGGGTTGT 573143

Query 164872 AGAAAGGGGAGTAACGGAGTAAGTGGGGAGAAAAGGGAGCTAGCTAGAGTACGAGGGGAA 164931

||||||||||||||||||||||||||||||||||||||||||||||||||||||||||||

Sbjct 573142 AGAAAGGGGAGTAACGGAGTAAGTGGGGAGAAAAGGGAGCTAGCTAGAGTACGAGGGGAA 573083

Query 164932 GTCACTGCCGTTGATGAAAGGATTCCTGATAAGTTGAATCGGGACTGTATCATTTTAAAA 164991

|||||||||||||||||||||||||||||||||||||||||||||||||||||||| |||

Sbjct 573082 GTCACTGCCGTTGATGAAAGGATTCCTGATAAGTTGAATCGGGACTGTATCATTTTCAAA 573023

Query 164992 AATAAGGGCGTAGCGGAGAAAAGAAAGATTGGATGATTCTAGTGAACTCAAACCTCTGGC 165051

||||||||||||||||||||||||||||||||||||||||||||||||||||||||||||

Sbjct 573022 AATAAGGGCGTAGCGGAGAAAAGAAAGATTGGATGATTCTAGTGAACTCAAACCTCTGGC 572963

Query 165052 ATTCCCTCTACTCAACCTTGCCTAGCTGCCGTAAGATACCTACCCGGGCTACCAAGCCTA 165111

||||||||||||||||||||||||||||||||||||||||||||||||||||||||||||

Sbjct 572962 ATTCCCTCTACTCAACCTTGCCTAGCTGCCGTAAGATACCTACCCGGGCTACCAAGCCTA 572903

Query 165112 ACAACTAAACAAGGAAAAGGCCAAACCATATTATCATCATAAATAGTATAAAGCCTACTC 165171

|||||||||||||||||||||||||||| |||||||||||||||||||||||||||||||

Sbjct 572902 ACAACTAAACAAGGAAAAGGCCAAACCAGATTATCATCATAAATAGTATAAAGCCTACTC 572843

Query 165172 TCTCCTTTCAGATTCAAAGGCACCGGTTCCCGATGTAGGTGCTTCAATCTGTCCAACTCT 165231

||||||||||||||||||||||||||||||||||||||||||||||||||||||||||||

Sbjct 572842 TCTCCTTTCAGATTCAAAGGCACCGGTTCCCGATGTAGGTGCTTCAATCTGTCCAACTCT 572783

Query 165232 TCTTTCTCCTTTTGCTTTTGCTTAAAAAAGACAACCAATCCAATCCCACTAttttttttt 165291

||||||||||||||||||||||||||||||||||||||||||||||||||||||||||||

Sbjct 572782 TCTTTCTCCTTTTGCTTTTGCTTAAAAAAGACAACCAATCCAATCCCACTATTTTTTTTT 572723

Query 165292 AGGCAAACAAATCCTCAGCCATTTCCGACACGACGTGAGTGAGATAATTACCACTCATTC 165351

||||||||||||||||||||||||||||||||||||||||||||||||||||||||||||

Sbjct 572722 AGGCAAACAAATCCTCAGCCATTTCCGACACGACGTGAGTGAGATAATTACCACTCATTC 572663

Query 165352 ATCGTTCTTCTCTTTAGaaaaaaaGTACTCGAGGGCTTGTTCAAAGCCCCTAGAGTATAA 165411

||||||||||||||||||||||||||||||||||||||||||||||||||||||||||||

Sbjct 572662 ATCGTTCTTCTCTTTAGAAAAAAAGTACTCGAGGGCTTGTTCAAAGCCCCTAGAGTATAA 572603

Query 165412 GAAGAAAAAATAAGAAAAGCCATGCATGATGTTTCCACTCCATTTTCATTACGAAGATAT 165471

||||||||||||||||||||||||||||||||||||||||||||||||||||||||||||

Sbjct 572602 GAAGAAAAAATAAGAAAAGCCATGCATGATGTTTCCACTCCATTTTCATTACGAAGATAT 572543

Query 165472 ATTACGTCAGGATCTCTTGCTCAAACTGAATTACGCCAATGTTATGGAAGTTCCTGGATC 165531

||||||||||||||||||||||||||||||||||||||||||||||||||||||||||||

Sbjct 572542 ATTACGTCAGGATCTCTTGCTCAAACTGAATTACGCCAATGTTATGGAAGTTCCTGGATC 572483

Query 165532 CTTTGAAATAAGATTAGTACCAAAAGCAGGAGGCTCTGATTTCAGAATCCTTTTTGGGAA 165591

||||||||||||||||||||||||||||||||||||||||||||||||||||||||||||

Sbjct 572482 CTTTGAAATAAGATTAGTACCAAAAGCAGGAGGCTCTGATTTCAGAATCCTTTTTGGGAA 572423

Query 165592 ATTGGCTACGGAGATTTTGTGCGGTCAGAAATTCATACAGaaaaaaaGGGGCCTTGATTT 165651

||||||||||||||||||||||||||||||||||||||||||||||||||||||||||||

Sbjct 572422 ATTGGCTACGGAGATTTTGTGCGGTCAGAAATTCATACAGAAAAAAAGGGGCCTTGATTT 572363

Query 165652 CAAAGCAGGAAAGTCCTTTCGACCCAATCCATTCTTGGGGTCCAAAAAAGACACTGGATC 165711

|||||||||||||||||||||| |||||||||||||||||||||||||||||||||||||

Sbjct 572362 CAAAGCAGGAAAGTCCTTTCGATCCAATCCATTCTTGGGGTCCAAAAAAGACACTGGATC 572303

Query 165712 TGTCAGTAACTTTTCACGACAAAGCGTTGTCCGAGGGCATGGAATGTTCAATTTTTTGGT 165771

||||||||||||||||||||||||||||||||||||||||||||||||||||||||||||

Sbjct 572302 TGTCAGTAACTTTTCACGACAAAGCGTTGTCCGAGGGCATGGAATGTTCAATTTTTTGGT 572243

Query 165772 CAGAATCTTGAAAATAATTTATATTTTAGATTATCCGGTCGAAATACGGGAAAATTCCAT 165831

||||||||||||||||||||||||||||||||||||||||||||||||||||||||||||

Sbjct 572242 CAGAATCTTGAAAATAATTTATATTTTAGATTATCCGGTCGAAATACGGGAAAATTCCAT 572183

Query 165832 TAAATTCTCTATGGAAACGGAATTTTTAGAATTCTACCCAGAACTGGAAGATCATTTCGA 165891

||||||||||||||||||||||||||||||||||||||||||||||||||||||||||||

Sbjct 572182 TAAATTCTCTATGGAAACGGAATTTTTAGAATTCTACCCAGAACTGGAAGATCATTTCGA 572123

Query 165892 GATATTCGAGCATATTCGAGGGTTCAATGTGACTATTTTCACTTCGGCCAACACAAAAGA 165951

||||||||||||||||||||||||||||||||||||||||||||||||||||||||||||

Sbjct 572122 GATATTCGAGCATATTCGAGGGTTCAATGTGACTATTTTCACTTCGGCCAACACAAAAGA 572063

Query 165952 TGAGACTTTACTACTGTGGAGCGGCTTTTTGCAAGATGAATGAGGTTTGCTAAAACTAAC 166011

||||||||||||||||||||||||||||||||||||||||||||||||||||||||||||

Sbjct 572062 TGAGACTTTACTACTGTGGAGCGGCTTTTTGCAAGATGAATGAGGTTTGCTAAAACTAAC 572003

Query 166012 TAAATAAAGTTGATGTCAGATAAGCGAAATATATACGAGATCACAAACGTAGATTGCTTG 166071

||||||||||||||||||||||||||||||||||||||||||||||||||||||||||||

Sbjct 572002 TAAATAAAGTTGATGTCAGATAAGCGAAATATATACGAGATCACAAACGTAGATTGCTTG 571943

Query 166072 CGGCCAAATATGATTTGAGACGAAAGCTTTATATTATAAAGAAAGCCCTTTTTCAAGATC 166131

||||||||||| |||||||||||||||||||||||||||||||||||||||||||||

Sbjct 571942 CGGCCAAATAT----TGAGACGAAAGCTTTATATTATAAAGAAAGCCCTTTTTCAAGATC 571887

Query 166132 CCGCCCTCCGTCTGATATGCCATCGTTCGTTGTTGTCCCAGTTGCCAAGAAAGAGTTCCT 166191

||||||||||||||||||||||||||||||||||||||||||||||||||||||||||||

Sbjct 571886 CCGCCCTCCGTCTGATATGCCATCGTTCGTTGTTGTCCCAGTTGCCAAGAAAGAGTTCCT 571827

Query 166192 TTGCACGAATAAGAAACCTATGTTATGTATTGACACGGGTCGCCTTCGTGGAGTATAGAT 166251

|||||||||||||||||||||||||||||||||||||||||||| |||||||||||||||

Sbjct 571826 TTGCACGAATAAGAAACCTATGTTATGTATTGACACGGGTCGCCCTCGTGGAGTATAGAT 571767

Query 166252 GACTTTCGCTTTTATCATATCGtttttttttCATGGAGGACGAGGTTATGGGTCTAAAGA 166311

||||||||||||||||||||||||||||||||||||||||||||||||||||||||||||

Sbjct 571766 GACTTTCGCTTTTATCATATCGTTTTTTTTTCATGGAGGACGAGGTTATGGGTCTAAAGA 571707

Query 166312 AATCGTCTTGGTAGGGCACCAAACCAATATAAAAACGGTGAGCTCTGCAGCTGGTCCACA 166371

||||||||||||||||||||||||||||||||||||||||||||||||||||||||||||

Sbjct 571706 AATCGTCTTGGTAGGGCACCAAACCAATATAAAAACGGTGAGCTCTGCAGCTGGTCCACA 571647

Query 166372 AGCAAGGGTTGTAGGTCTATTACCGTCCGGTTCCGGGCCGAAACGGAGGAGTCATCCCAA 166431

|||||||||||||||||||||||||||||||||||| |||||||||||||||||||||||

Sbjct 571646 AGCAAGGGTTGTAGGTCTATTACCGTCCGGTTCCGGACCGAAACGGAGGAGTCATCCCAA 571587

Query 166432 CTCTCCTATCGTAGGGACGGGAATCGAAGTGGGGGACCTCTCTACCTCCCCAGACATAGA 166491

||||||||||||||||||||||||||||||||||||||||||||||||||||||||||||

Sbjct 571586 CTCTCCTATCGTAGGGACGGGAATCGAAGTGGGGGACCTCTCTACCTCCCCAGACATAGA 571527

Query 166492 CTACGTACAGGGCCGCGTAAACAAGAGAAAAAGTGAAGAATTTCACTCGCCACTATCAGA 166551

||||||||||||||||||||||||||||||||||||||||||||||||||||||||||||

Sbjct 571526 CTACGTACAGGGCCGCGTAAACAAGAGAAAAAGTGAAGAATTTCACTCGCCACTATCAGA 571467

Query 166552 CATTCATTTTGGGAGAAAACTCACTCTTGTTCATGCTTAGCGCCCTGGAGTACTGGGGTT 166611

||||||||||||||||||||||||||||||||||||||||||||||||||||||||||||

Sbjct 571466 CATTCATTTTGGGAGAAAACTCACTCTTGTTCATGCTTAGCGCCCTGGAGTACTGGGGTT 571407

Query 166612 GTTCCCACCCaaaaagaaaaaaaaGTTTTTCGTTTCGTTGGTAGAACCCACGCCAATCTC 166671

||||||||||||||||||||||||||||||||||||||||||||||||||||||||||||

Sbjct 571406 GTTCCCACCCAAAAAGAAAAAAAAGTTTTTCGTTTCGTTGGTAGAACCCACGCCAATCTC 571347

Query 166672 TTTTTCACTCTATGATAGTGAAAAACATGAAATTCTCTCCTCGGAGAAAGGGGGAAGAGG 166731

||||||||||||||||||||||||||||||||||||||||||||||||||||||||||||

Sbjct 571346 TTTTTCACTCTATGATAGTGAAAAACATGAAATTCTCTCCTCGGAGAAAGGGGGAAGAGG 571287

Query 166732 AGTACGATAGAGAAGAGAAATTATGGACCTATATCGTAACGCGGCCGAACGTATCCTTGG 166791

||||||||||||||||||||||||||||||||||||||||||||||||||||||||||||

Sbjct 571286 AGTACGATAGAGAAGAGAAATTATGGACCTATATCGTAACGCGGCCGAACGTATCCTTGG 571227

Query 166792 GGAGAGCAAGAATATAGATATATTTTACCATGGGGAGGGCAAGGATATATTTTTTCTGGA 166851

||||||||||||||||||||||||||||||||||||||||||||||||||||||||||||

Sbjct 571226 GGAGAGCAAGAATATAGATATATTTTACCATGGGGAGGGCAAGGATATATTTTTTCTGGA 571167

Query 166852 AGAACAGTTGAATGATTTGAAAAGAAAGGGAGTAGAAAGTGGAGCCTATCAGGATGCTCT 166911

||||||||||||||||||||||||||||||||||||||||||||||||||||||||||||

Sbjct 571166 AGAACAGTTGAATGATTTGAAAAGAAAGGGAGTAGAAAGTGGAGCCTATCAGGATGCTCT 571107

Query 166912 AAGCATAACCCCCGGCACCGACAGCCCCCTTGATCAATTTGAAATCTTCCCTTTGCTTCC 166971

||||||||||||||||||||||||||||||||||||||||||||||||||||||||||||

Sbjct 571106 AAGCATAACCCCCGGCACCGACAGCCCCCTTGATCAATTTGAAATCTTCCCTTTGCTTCC 571047

Query 166972 TATGAAAATAGGAGACTTCTTTTTCTCATTCACAAATTCATCCTTGTTTATGCTGCTTAC 167031

||||||||||||||||||||||||||||||||||||||||||||||||||||||||||||

Sbjct 571046 TATGAAAATAGGAGACTTCTTTTTCTCATTCACAAATTCATCCTTGTTTATGCTGCTTAC 570987

Query 167032 TCTCGGTTTGGTCCTACTTCTGATTTTGCTTGTAACGAAAAAGGGAGGGGGAAAGTTAGT 167091

||||||||||||||||||||||||||||||||||||||||||||||||||||||||||||

Sbjct 570986 TCTCGGTTTGGTCCTACTTCTGATTTTGCTTGTAACGAAAAAGGGAGGGGGAAAGTTAGT 570927

Query 167092 GCCAAATGCTTGGCAATCCGTGGTAGAGCTTATTTATGATTTCGTGCTGAACCTGGTAAA 167151

||||||||||||||||||||||||||||||||||||||||||||||||||||||||||||

Sbjct 570926 GCCAAATGCTTGGCAATCCGTGGTAGAGCTTATTTATGATTTCGTGCTGAACCTGGTAAA 570867

Query 167152 CGAACAAATAGGTGGTCCTTCTGCAAATGTGAAACAAAAGTTTTTCCCTTGCATCCTGGT 167211

||||||||||||||||||||||||||||||||||||||||||||||||||||||||||||

Sbjct 570866 CGAACAAATAGGTGGTCCTTCTGCAAATGTGAAACAAAAGTTTTTCCCTTGCATCCTGGT 570807

Query 167212 TACTTTTACTTTTTTGTTATTTTGTAATCTTCAGGGTATGATACCCTTTAGCTTCACAGT 167271

||||||||||||||||||||||||||||||||||||||||||||||||||||||||||||

Sbjct 570806 TACTTTTACTTTTTTGTTATTTTGTAATCTTCAGGGTATGATACCCTTTAGCTTCACAGT 570747

Query 167272 GACAAGTCATTTTCTCATTACTTTGGCTCTCTCCTTTTCTCTTTTTATAGGCATTACTAT 167331

||||||||||||||||||||||||||||||||||||||||||||||||||||||||||||

Sbjct 570746 GACAAGTCATTTTCTCATTACTTTGGCTCTCTCCTTTTCTCTTTTTATAGGCATTACTAT 570687

Query 167332 AGTGGGATTTCAAAGACATGGGCTTCAtttttttAGCTTCTTATTACCTGCAGGAGTCCC 167391

||||||||||||||||||||||||||||||||||||||||||||||||||||||||||||

Sbjct 570686 AGTGGGATTTCAAAGACATGGGCTTCATTTTTTTAGCTTCTTATTACCTGCAGGAGTCCC 570627

Query 167392 ACTGCCGTTAGCACCTTTTTTAGTACTCCTAGAGCTAATCTCTCATTGTTTTCGTGCATT 167451

||||||||||||||||||||||||||||||||||||||||||||||||||||||||||||

Sbjct 570626 ACTGCCGTTAGCACCTTTTTTAGTACTCCTAGAGCTAATCTCTCATTGTTTTCGTGCATT 570567

Query 167452 AAGCTCAGGAATACGTCTATTTGCTAATATGATGGCCGGTCATAGTTTAGTAAAGATTTT 167511

||||||||||||||||||||||||||||||||||||||||||||||||||||||||||||

Sbjct 570566 AAGCTCAGGAATACGTCTATTTGCTAATATGATGGCCGGTCATAGTTTAGTAAAGATTTT 570507

Query 167512 AAGTGGGTTTGCTTGGACTATGCTCTTTATGAATAATATTTTTTATTTCATAGGAGATCT 167571

||||||||||||||||||||||||||||||||||||||||||||||||||||||||||||

Sbjct 570506 AAGTGGGTTTGCTTGGACTATGCTCTTTATGAATAATATTTTTTATTTCATAGGAGATCT 570447

Query 167572 TGGTCCTTTATTTATAGTTCTTGCATTAACGGGTCTGGAATTAGGTGTAGCTATATTACA 167631

||||||||||||||||||||||||||||||||||||||||||||||||||||||||||||

Sbjct 570446 TGGTCCTTTATTTATAGTTCTTGCATTAACGGGTCTGGAATTAGGTGTAGCTATATTACA 570387

Query 167632 AGCTTATGTTTTTACGATCTTAATCTGTATTTACTTGAATGATGCTATAAATCTCCATTA 167691

||||||||||||||||||||||||||||||||||||||||||||||||||||||||||||

Sbjct 570386 AGCTTATGTTTTTACGATCTTAATCTGTATTTACTTGAATGATGCTATAAATCTCCATTA 570327

Query 167692 AAGGCCGCAGGAAGAATTTCATAATTGAATAAAAAAGAGGTTTTCATATATAACCAACCT 167751

||||||||||||||||||||||||||||||||||||||||||||||||||||||||||||

Sbjct 570326 AAGGCCGCAGGAAGAATTTCATAATTGAATAAAAAAGAGGTTTTCATATATAACCAACCT 570267

Query 167752 TTTTCAAAACATTCCTAATAAATAGGTGGATTTGATCATAGTATTCCATTAAGCATTTTC 167811

||||||||||||||||||||||||||||||||||||||||||||||||||||||||||||

Sbjct 570266 TTTTCAAAACATTCCTAATAAATAGGTGGATTTGATCATAGTATTCCATTAAGCATTTTC 570207

Query 167812 ATTAAGAGTCGATTTGATATCATAGTCGTGGAATGGCGGGACTCTTGGATTGCAGgaaag 167871

||||||||||||||||||||||||||||||||||||||||||||||||||||||||||||

Sbjct 570206 ATTAAGAGTCGATTTGATATCATAGTCGTGGAATGGCGGGACTCTTGGATTGCAGGAAAG 570147

Query 167872 aaaaagggcgggcagaaaggggcaaaagtttcttagaagaaggaagatgaaaaaaaCAAC 167931

||||||||| |||||||||||||||||||||||||||||||||||||||||||||||

Sbjct 570146 AAAAAGGGC----AGAAAGGGGCAAAAGTTTCTTAGAAGAAGGAAGATGAAAAAAACAAC 570091

Query 167932 TAGTTATCTTGGTGCTAAAGAAACAAAAGAAAAACGGCTTTTTGATTAATGGAGCACGAT 167991

||||||||||||||||||||||||||||||||||||||||||||||||||||||||||||

Sbjct 570090 TAGTTATCTTGGTGCTAAAGAAACAAAAGAAAAACGGCTTTTTGATTAATGGAGCACGAT 570031

Query 167992 ATTATTCTAACAAATTAGAAACACACCCAAACGTCAGGGTGTATCTTTCGGATAATAACC 168051

||||||||||||||||||||||||||||||||||||||||||||||||||||||||||||

Sbjct 570030 ATTATTCTAACAAATTAGAAACACACCCAAACGTCAGGGTGTATCTTTCGGATAATAACC 569971

Query 168052 GTGGTATGGTTATAGCGAGAATGGAGTTTTATAGATCAGTGGACTTGGGTTCTTCAACTT 168111

||||||||||||||||||||||||||||||||||||||||||||||||||||||||||||

Sbjct 569970 GTGGTATGGTTATAGCGAGAATGGAGTTTTATAGATCAGTGGACTTGGGTTCTTCAACTT 569911

Query 168112 TTGTTTATTCTGAAATAATCAAAGTGGTTTTAATTCACTTTGTGCCATACATAAGCGGTT 168171

||||||||||||||||||||||||||||||||||||||||||||||||||||||||||||

Sbjct 569910 TTGTTTATTCTGAAATAATCAAAGTGGTTTTAATTCACTTTGTGCCATACATAAGCGGTT 569851

Query 168172 TGGCTAAAATGACAATAGTGCCATCAGTCAAGTCATCTACTGCTGGTTACACCTTTGACT 168231

||||||||||||||||||||||||||||||||||||||||||||||||||||||||||||

Sbjct 569850 TGGCTAAAATGACAATAGTGCCATCAGTCAAGTCATCTACTGCTGGTTACACCTTTGACT 569791

Query 168232 ATGCACTTGGGGATGTACGCCAATGAGATTGGTTTCGCACTCAGTGGATGTAGTATATAT 168291

||||||||||||||||||||||||||||||||||||||||||||||||||||||||||||

Sbjct 569790 ATGCACTTGGGGATGTACGCCAATGAGATTGGTTTCGCACTCAGTGGATGTAGTATATAT 569731

Query 168292 ATTCAATAAAATAAAAGAGCAGGTTATCGAACCAAGGGATTCGGAGATCAAGGTTGAGCG 168351

||||||||||||||||||||||||||||||||||||||||||||||||||||||||||||

Sbjct 569730 ATTCAATAAAATAAAAGAGCAGGTTATCGAACCAAGGGATTCGGAGATCAAGGTTGAGCG 569671

Query 168352 TACAGTGGAATTTCTAAAGAAAATCAAGCCGGTTAATCTAGCTCCTTTCTTGGTAGCCGA 168411

||||||||||||||||||||||||||||||||||||||||||||||||||||||||||||

Sbjct 569670 TACAGTGGAATTTCTAAAGAAAATCAAGCCGGTTAATCTAGCTCCTTTCTTGGTAGCCGA 569611

Query 168412 TATGGAAACCGTTTTGAATGAAGAAGCTATTCATGTTCCATACGCTGTAGGTGTTCTTAA 168471

||||||||||||||||||||||||||||||||||||||||||||||||||||||||||||

Sbjct 569610 TATGGAAACCGTTTTGAATGAAGAAGCTATTCATGTTCCATACGCTGTAGGTGTTCTTAA 569551

Query 168472 AGTTATCCCTGGTATGAGTCTTTCAAAGGAATCTATAATGTGCTGATTTAGTGAGGATTT 168531

|||| |||||||||||||||||||||||||||||||||||||||||||||||||||||||

Sbjct 569550 AGTTCTCCCTGGTATGAGTCTTTCAAAGGAATCTATAATGTGCTGATTTAGTGAGGATTT 569491

Query 168532 GACTTTGGTGCCTGATTTTTAGAGCCGAAGTCGTAAAATGTTGAATTACTTTCTTTACTA 168591

||||||||||||||||||| ||||||||||||||||||||||||||||||||||||||||

Sbjct 569490 GACTTTGGTGCCTGATTTTGAGAGCCGAAGTCGTAAAATGTTGAATTACTTTCTTTACTA 569431

Query 168592 TGTTGTTTCTTTAGCGAGAACTGAATCTAAAATACGGGTTATATACTTTCATAATTTCTC 168651

||||||||||||||||||||||||||||||||||||||||||||||||||||||||||||

Sbjct 569430 TGTTGTTTCTTTAGCGAGAACTGAATCTAAAATACGGGTTATATACTTTCATAATTTCTC 569371

Query 168652 TAGGTTTGAAGGTATTCTTATTATTCAACATATAATAGGTAATAGACCAGAATGGGGTCT 168711

||||||||||||||||||||||||||||||||||||||||||||||||||||||||||||

Sbjct 569370 TAGGTTTGAAGGTATTCTTATTATTCAACATATAATAGGTAATAGACCAGAATGGGGTCT 569311

Query 168712 TAAGCCATTAATGCGAAATGGCGAAGTATAGAAATTTGAAATAAAAATAGAAAAAAGGAT 168771

||||||||||||||||||||||||||||||||||||||||||||||||||||||||||||

Sbjct 569310 TAAGCCATTAATGCGAAATGGCGAAGTATAGAAATTTGAAATAAAAATAGAAAAAAGGAT 569251

Query 168772 TAAGTTGGTTTTTCGCGATTCCATGAAGCAAAGGGAACCCAGAAAGGATGGAGGTAGAAA 168831

||||||||||||||||||||||||||||||||||||||||||||||||||||||||||||

Sbjct 569250 TAAGTTGGTTTTTCGCGATTCCATGAAGCAAAGGGAACCCAGAAAGGATGGAGGTAGAAA 569191

Query 168832 GATAGTTAACCAGGCTTCCCTGCTCGTTCAAAGTACCAATACTTACTTTTTTCTATATGA 168891

||||||||||||||||||||||||||||||||||||||||||||||||||||||||||||

Sbjct 569190 GATAGTTAACCAGGCTTCCCTGCTCGTTCAAAGTACCAATACTTACTTTTTTCTATATGA 569131

Query 168892 TGATATGATAAAGACGGAAGTCGGCGAAGGGCAAGGACGCAATACATATTGGTAAGCGAG 168951

||||||||||||||||||||||||||||||||||||||||||||||||||||||||||||

Sbjct 569130 TGATATGATAAAGACGGAAGTCGGCGAAGGGCAAGGACGCAATACATATTGGTAAGCGAG 569071

Query 168952 TGGCTTATTATATAGTTAGATATCAGTTTGATACGGATATTTCCTTGTTTGTTGTATCTT 169011

|||||||||| |||||||||||||||||||||||||||||||||||||||||||||||||

Sbjct 569070 TGGCTTATTAGATAGTTAGATATCAGTTTGATACGGATATTTCCTTGTTTGTTGTATCTT 569011

Query 169012 CCGATCAAGCGGATTTAGTGGATCCCCCGCTTATATCCCCTGCTTATATAGTTATAGTTT 169071

||||||||||||||||||||||||||||||||||||||||||||||||||||||||||||

Sbjct 569010 CCGATCAAGCGGATTTAGTGGATCCCCCGCTTATATCCCCTGCTTATATAGTTATAGTTT 568951

Query 169072 AGCTTTCATTCAATTAGTTGTTGTGTGCTTTGAATTGAAGTTCCAATTCCACCAGTTGTG 169131

||||||||||||||||||||||||||||||||||||||||||||||||||||||||||||

Sbjct 568950 AGCTTTCATTCAATTAGTTGTTGTGTGCTTTGAATTGAAGTTCCAATTCCACCAGTTGTG 568891

Query 169132 GTTTCAGGACCAAAGGGAAGTATGCTATAGAAATAGTAGATACGATGGTAGGCCGAGGCC 169191

||||||||||||||||||||||||||||||||||||||||||||||||||||||||||||

Sbjct 568890 GTTTCAGGACCAAAGGGAAGTATGCTATAGAAATAGTAGATACGATGGTAGGCCGAGGCC 568831

Query 169192 TGAAAGTCTTTTTCTTACAAATCGTAGTGATGAAGGCCTTACTACTAAATATATGGCAGG 169251

||||||||||||||||||||||||||||||||||||||||||||||||||||||||||||

Sbjct 568830 TGAAAGTCTTTTTCTTACAAATCGTAGTGATGAAGGCCTTACTACTAAATATATGGCAGG 568771

Query 169252 AGAGTCGGCATGAACAGGTGTTCAAAAGGAAGTAGGATGAATGTTCGTAGGCAATCTTTT 169311

||||||||||||||||||||||||||||||||||||||||||||||||||||||||||||

Sbjct 568770 AGAGTCGGCATGAACAGGTGTTCAAAAGGAAGTAGGATGAATGTTCGTAGGCAATCTTTT 568711

Query 169312 TCTTTCTATCGGTCAAGACCTATCCTTTCTTTATACTCTGCAGTCCTGCCTCGAGAGAGG 169371

||||||||||||||||||||||||||||||||||||||||||||||||||||||||||||

Sbjct 568710 TCTTTCTATCGGTCAAGACCTATCCTTTCTTTATACTCTGCAGTCCTGCCTCGAGAGAGG 568651

Query 169372 TGGCGTGGCTCAGGTGAGATTGTAATAGGTCAATTAAGGCCTCAGACAGCGAACCAATCG 169431

||||||||||||||||||||||||||||||||||||||||||||||||||||||||||||

Sbjct 568650 TGGCGTGGCTCAGGTGAGATTGTAATAGGTCAATTAAGGCCTCAGACAGCGAACCAATCG 568591

Query 169432 TCCTATGTCCCGAGATATTATGACGAACCATACTTGGCATTCGGCTATTATCATATAATA 169491

|||||||||||||||||||||||||||||||||||||||||||||||||||||| |||||

Sbjct 568590 TCCTATGTCCCGAGATATTATGACGAACCATACTTGGCATTCGGCTATTATCATCTAATA 568531

Query 169492 AGTACGTAGTAGGGGCAGGATAATAATTGGGAAACTCCCAATTGCCAAGAACGGAGAATT 169551

||||||||||||||||||||||||||||||||||||||||||||||||||||||||||||

Sbjct 568530 AGTACGTAGTAGGGGCAGGATAATAATTGGGAAACTCCCAATTGCCAAGAACGGAGAATT 568471

Query 169552 GGCTTGAAGTTAGGAGGAAAGAAAGTAGTCCTTTCTTACTACTTTTATTTTAGATATTTT 169611

||||||||||||||||||||||||||||||||||||||||||||| |||||||||| ||

Sbjct 568470 GGCTTGAAGTTAGGAGGAAAGAAAGTAGTCCTTTCTTACTACTTTGATTTTAGATAGATT 568411

Query 169612 AAGAAATTAGGGACTGGAAGAGAAGACTTTGAAGCAGAAAGAGGGTAAGCAAGAAATAGG 169671

|||||||| |||||||||||||||||||||||||||||||||||||||||||||||||||

Sbjct 568410 AAGAAATTCGGGACTGGAAGAGAAGACTTTGAAGCAGAAAGAGGGTAAGCAAGAAATAGG 568351

Query 169672 AATAATAGTAGCTGGATAGGCtttttttATTTCCGAAGCAAGTAAGGTAGGTATAGCCTC 169731

||||||||||||||||||||||||||||||||||||||||||||||||||||||||||||

Sbjct 568350 AATAATAGTAGCTGGATAGGCTTTTTTTATTTCCGAAGCAAGTAAGGTAGGTATAGCCTC 568291

Query 169732 CTAGCTTGGTTTAATAGGGGCTTATGCGTAACGTTGTATAGAAAAAGGAGCGGAGTATTC 169791

||||||||||||||||||||||||||||||||||||||||||||||||||||||||||||

Sbjct 568290 CTAGCTTGGTTTAATAGGGGCTTATGCGTAACGTTGTATAGAAAAAGGAGCGGAGTATTC 568231

Query 169792 ATTGAATAGGTGTTTTAGGTATCCAAAAAAGGGAGAAGGTCATAGACAAGGGTAAACTGA 169851

||||||||||||||||||||||||||||||||||||||||||||||||||||||||||||

Sbjct 568230 ATTGAATAGGTGTTTTAGGTATCCAAAAAAGGGAGAAGGTCATAGACAAGGGTAAACTGA 568171

Query 169852 ATAACCAAGGTAAGCTACTAAGCTCACGGATAGTTGGTTTAGGGCTAGTTGGCACTGTCG 169911

||||||||||||||||||||||||||||||||||||||||||||||||||||||||||||

Sbjct 568170 ATAACCAAGGTAAGCTACTAAGCTCACGGATAGTTGGTTTAGGGCTAGTTGGCACTGTCG 568111

Query 169912 GGGCAAAGCATTCGATCGAAGTAGTAGTAGTTTCTAAATAGTCATTCAAGGTAGGAGTTG 169971

||||||||||||||||||||||||||||||||||||||||||||||||||||||||||||

Sbjct 568110 GGGCAAAGCATTCGATCGAAGTAGTAGTAGTTTCTAAATAGTCATTCAAGGTAGGAGTTG 568051

Query 169972 GAGAATCAAGCTCATAGGCATATGGATGATTGTTGGCACCGGTAAGCTTATAGGTAGGAG 170031

||||||||||||||||||||||||||||||||||||||||||||||||||||||||||||

Sbjct 568050 GAGAATCAAGCTCATAGGCATATGGATGATTGTTGGCACCGGTAAGCTTATAGGTAGGAG 567991

Query 170032 CTCGGAATCGGAGCTAGCTAGGACTAGCAAGTATAGGGACAAGGGACGTTGTAGTTGTTG 170091

||||||||||||||||||||||||||||||||||||||||||||||||||||||||||||

Sbjct 567990 CTCGGAATCGGAGCTAGCTAGGACTAGCAAGTATAGGGACAAGGGACGTTGTAGTTGTTG 567931

Query 170092 TGAGGAATAGGGAGAAAAGGTAAGCTTATAGAGCTAGCAAGGAAAACAAGTATGTTTACA 170151

||||||||||||||||||||||||||||||||||||||||||||||||||||||||||||

Sbjct 567930 TGAGGAATAGGGAGAAAAGGTAAGCTTATAGAGCTAGCAAGGAAAACAAGTATGTTTACA 567871

Query 170152 CGGACGGTATTTTTGAAAAGCAAATCAAGGCAAGTAGGCCAGAATAACCCAAATAGGAAG 170211

||||||||||||||||||||||||||||||||||||||||||||||||||||||||||||

Sbjct 567870 CGGACGGTATTTTTGAAAAGCAAATCAAGGCAAGTAGGCCAGAATAACCCAAATAGGAAG 567811

Query 170212 GTTTATATACCTCACCATGACTAGGCCTTACATAAGTTATTTTTTCAAATTACAGAGTGA 170271

||||||||||||||||||||||||||||||||||||||||||||||||||||||||||||

Sbjct 567810 GTTTATATACCTCACCATGACTAGGCCTTACATAAGTTATTTTTTCAAATTACAGAGTGA 567751

Query 170272 GTTTATGCATGCGCCGAAAGAGTCACATATGGATGCTGGTATTCAGATGGTAAGATAAGA 170331

||||||||||||||||||||||||||||||||||||||||||||||||||||||||||||

Sbjct 567750 GTTTATGCATGCGCCGAAAGAGTCACATATGGATGCTGGTATTCAGATGGTAAGATAAGA 567691

Query 170332 TATATATAAGGATGTCTGGACTAGGACTGATAAAGAAGACCGCAATTTAGGGCTTAGAAT 170391

||||||||||||||||||||||||||||||||| ||||||||||||||||||||||||||

Sbjct 567690 TATATATAAGGATGTCTGGACTAGGACTGATAATGAAGACCGCAATTTAGGGCTTAGAAT 567631

Query 170392 TTCAAAGATTCTTCGATTCGGATTAGGCTTCATGTCCAATGACAATCAAATCATTGACTG 170451

||||||||||||||||||||||||||||||||||||||||||||||||||||||||||||

Sbjct 567630 TTCAAAGATTCTTCGATTCGGATTAGGCTTCATGTCCAATGACAATCAAATCATTGACTG 567571

Query 170452 GAGTGTAATGCTGGGAGATTCTAGAATCTCTTGGAAATGTAAGAAAGAGTCAAATCCATC 170511

||||||||||||||||||||||||||||||||||||||||||||||||||||||||||||
[truncated: 794,092 more chars]
